# Stereodivergent propargylic alkylation of enals via cooperative NHC and copper catalysis

**DOI:** 10.1038/s41467-022-29059-0

**Published:** 2022-03-15

**Authors:** Yu-Hua Wen, Zi-Jing Zhang, Shuai Li, Jin Song, Liu-Zhu Gong

**Affiliations:** 1 Department of Chemistry, University of Science and Technology of China, 230026 Hefei, China; 2 Institutes of Physical Science and Information Technology, Anhui University, 230601 Hefei, China; 3 Center for Excellence in Molecular Synthesis of Chinese Academy of Sciences, 230026 Hefei, China

**Keywords:** Asymmetric catalysis, Synthetic chemistry methodology

## Abstract

Despite that asymmetric stereodivergent synthesis has experienced great success to provide unusual processes for the creation of chirality complexity, concepts appliable to asymmetric stereodivergent catalysis are still limited. The dependence on the unusual capacity of each catalyst to precisely control the reactive site planar in the region poses unparalleled constraints on this field. Here, we first demonstrate that the chiral Cu-allenylidene species can participate in the stereodivergent propargylic alkylation of enals, in concert with chiral N-heterocyclic carbenes (NHCs). Thus, all four stereoisomers were obtained with excellent enantioselectivity and diastereoselectivity (up to >99% e.e. and >95:5 d.r.) from the same starting materials by simply altering chiral Cu-Pybox complex and NHC combinations. The rich chemistry workable in the products enables the structurally diverse synthesis of chiral functional molecules and holds great potential in alkaloid synthesis, as showcased by the preparation of the key building block to access (-)-perophoramidine.

## Abstract (web summary)

The ability to construct multiple stereocenters in a modular fashion is an important goal of synthetic organic chemistry. Here the authors present a method to construct oxindoles in four stereoisomers with high enantioselectivity and diastereoselectivity from the same starting materials by using cooperative copper- and organocatalysis.

## Introduction

**Fig. 1:**
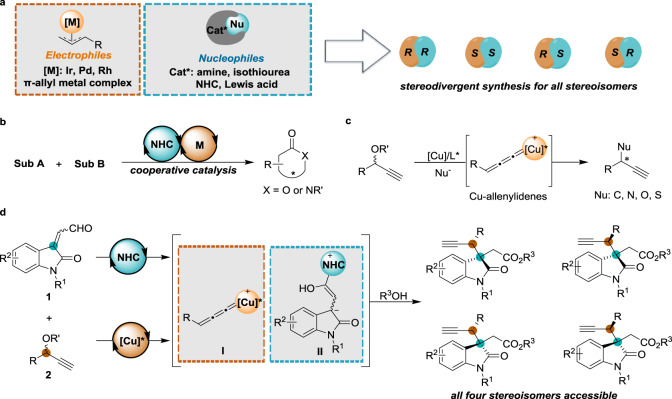
Stereodivergent synthesis via dual catalysis. **a** Representative stereodivergent synthesis via cooperative catalysis, chiral catalyst (Cat*). **b** NHC/transition metal cooperatively catalyzed enantioselective annulation reactions, substrate (sub). **c** Asymmetric propargylic substitution reactions mediated by chiral Cu-allenylidene complexes. **d** This work: the first stereodivergent propargylation of enals via cooperative NHC/copper catalysis, leading to all four stereoisomers with excellent optical purity.

Contiguous carbon stereogenic centers prevalently distribute in complex natural products and important bioactive compounds, and their absolute and relative configurations commonly exert some impact on the shape of the structurally complex molecules, which are closely related to the properties and biological activities^1^. Stereodivergent synthesis^2–7^, a direct access to all product stereoisomers incorporating vicinal stereogenic centers from the same set of starting materials, offers more opportunities for the investigation of structure−activity relationships to advance drug discovery and chemical biology study^1,8^. The recent decades have witnessed significant advances in cooperative catalysis, providing a preponderance of activation modes and stereochemical options to render asymmetric reactions^9–13^, and the robustness of each individual chiral catalyst allocates cooperative catalysis to enable stereodivergent synthesis. Carreira and coworkers first introduced and validated the concept by an asymmetric catalytic α-allylation of branched aldehydes^14^. All possible stereoisomers of the product were obtained by the orthogonal permutation of chiral amine and iridium complex of chiral phosphoramidite. Since this seminal work, substantial progress has been made on catalytic stereodivergent synthesis^15–33^ based on the cooperative action of different catalytic principles. In particular, the asymmetric coupling events of transient nucleophiles bonded with either chiral organocatalysts or Lewis acids with electrophilic π-allyl Ir^14–23,25–29^/Rh^24^/Pd^30–32^ intermediates have been intensively investigated and turn out to be the most general platforms to establish stereodivergent synthesis (Fig. 1a). While much has been accomplished, the exploration of chiral catalyst-coupled electrophiles beyond π-allyl metal complexes remains to be developed^33^ and is greatly desirable for broadening the domain of stereodivergent catalytic synthesis.

N-Heterocyclic carbene catalysis has seen appreciable research activity with the realization of abundant enantioselective transformations^34–37^. Recently, considerable efforts have been invested in the integration of NHC and transition metal catalysis^38–40^ to access new reactions^22,23,41–49^. However, stereoselective annulation reactions to produce lactones and lactams appear to be easily accessible and most successful, presumably due to the requirement for an intramolecular acyl transfer to a proximal nucleophile to facilitate the regeneration of the NHC catalyst (Fig. 1b). Over the past decades, copper-catalyzed asymmetric propargylic substitution reaction has proven to be a powerful method to assemble carbon-carbon and carbon-hetero bonds, wherein chiral ligands are employed to control the stereoselectivity (Fig. 1c)^50–54^. In this context, we anticipated that the coupling of the key Cu-allenylidene intermediate **I** and an NHC-bound nucleophile **II**^55^ using an external nucleophile as acyl acceptors^56^ might occur, leading to an NHC/copper cooperatively catalyzed asymmetric propargylation process (Fig. 1d). More significantly, the chiral Cu-allenylidene species **I** would be an ideal electrophile with high local stereocontrol^50–54^, and thus presumably enables access to stereodivergent catalytic synthesis, in concert with the chiral NHC-mediated nucleophilic event. However, such a stereodivergent propargylation reaction has not been described, yet. As a consequence, success in stereodivergent action of Cu-allenylidene species with NHC-bonded nucleophiles would add a new dimension to asymmetric catalysis. Herein, we report the first stereodivergent propargylic alkylation reaction of propargylic acetates with isatin-derived enals enabled by cooperative catalysis of chiral NHCs and copper complexes to deliver oxindole derivatives with excellent diastereoselectivity and enantioselectivity. The orthogonal alteration of enantiomers of NHCs and copper catalysts allows access to all four stereoisomers of the products.

## Results

### Optimization studies

To test the validity of our hypothesis, we began our investigation into the stereoselective propargylic alkylation reaction of isatin-derived enal **1a** with propargylic acetate **2a** under NHC/copper cooperative catalysis (Table 1 and Supplementary Tables 1–6). As expected, the desired propargylation oxindole product (*R*,*R*)-**3aa** was obtained in 70% yield with 93:7 diastereomeric ratio (d.r.) and 96% enantiomeric excess (e.e.) through the synergy of a chiral NHC catalyst generated in situ from **4a** and a copper complex with a chiral pyridine bis(oxazoline) (Pybox) ligand **L1**, by using methanol as the nucleophile required for the catalyst turnover (entry 1). Screening of chiral organocatalysts (entries 1–6, and Supplementary Table 1) revealed that the chiral NHC precatalyst **4a** appeared to be the most efficient Lewis base catalyst capable of delivering the highest diastereo- and enantioselectivities (entry 1). Copper salts had a considerable effect on the reaction performance and Cu(CH_3_CN)_4_PF_6_ was identified as the optimal metal catalyst precursor (Supplementary Table 2). The evaluation of Pybox **L2**-**L4** and other ligands (entries 7−9, and Supplementary Table 3) found that the combination of chiral NHC precatalyst **4a** with Pybox **L3** led to a significant enhancement in the stereoselectivity (entry 8). Either base or temperature also exerts impacts on the reaction (Supplementary Tables 4, 5). The variation of solvents indicated that the reaction gave the best results in tetrahydrofuran (entry 11) as compared to that in any other counterparts tested (entries 8, 10, 12 and Supplementary Table 6). Control experiments verified the necessity of each member of the combined catalyst system (entries 13−15), thus less than 5% yield was observed for the desired product in the absence of any of the NHC precatalyst, copper salt, and Pybox ligand.

**Table 1:** Reaction optimization.


Entry^a^	4	Ligand	Solvent	Yield (%)^b^	d.r.^c^	e.e. (%)^d^
1	**4a**	**L1**	Toluene	70	93:7	96
2	**4b**	**L1**	Toluene	20	87:13	94
3	**4c**	**L1**	Toluene	76	92:8	92
4	**4d**	**L1**	Toluene	74	67:33	29
5	**4e**	**L1**	Toluene	22	71:29	59
6	**4f**	**L1**	Toluene	83	50:50	18
7	**4a**	**L2**	Toluene	42	60:40	73
8	**4a**	**L3**	Toluene	51	>95:5	99
9	**4a**	**L4**	Toluene	39	81:19	87
10	**4a**	**L3**	DCM	47	>95:5	99
11	**4a**	**L3**	THF	79 (77)	>95:5	>99
12	**4a**	**L3**	MeOH	22	73:27	84
13	**–**	**L3**	THF	n.d.	–	–
14^e^	**4a**	**–**	THF	n.d.	–	–
15	**4a**	**–**	THF	<5	–	–

^a^Reaction conditions: Cu(CH_3_CN)_4_PF_6_ (5 mol%) and pyridine bis(oxazoline) ligand **L** (10 mol%) were stirred in solvent (0.5 mL) at 25 °C for 1 h, then NHC precatalyst **4** (5 mol%), **1a** (0.15 mmol), **2a** (0.1 mmol), MeOH (0.5 mmol), Na_2_CO_3_ (0.1 mmol) and solvent (0.5 mL) were added to the reaction mixture and stirred for 12 h under N_2._

^b^The yield was determined by ^1^H NMR spectroscopy (yield of isolated product given within parentheses).

^c^The diastereomeric ratio (d.r.) was determined by ^1^H NMR spectroscopy.

^d^The enantiomeric excess (e.e.) was determined by HPLC.

^e^In the absence of Cu(CH_3_CN)_4_PF_6_. Mes = mesityl, DCM = dichloromethane, THF = tetrahydrofuran, n.d. = not detected.

### Substrates scope with enals

**Fig. 2:**
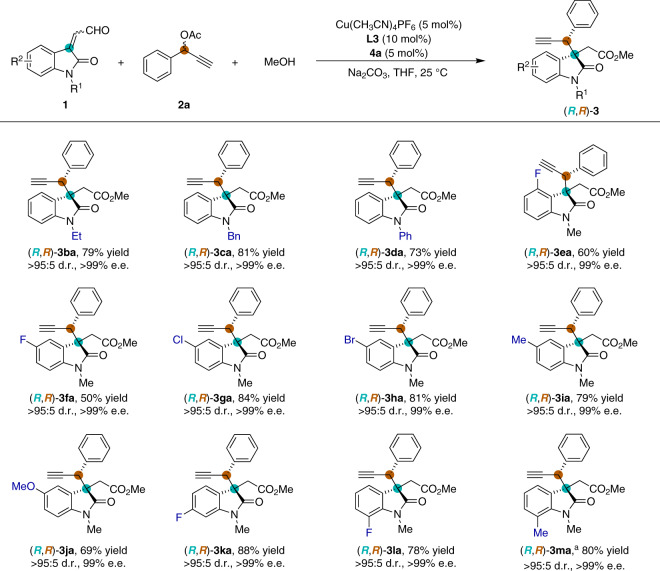
Substrate scope of isatin-derived enals. Reaction conditions: Cu(CH_3_CN)_4_PF_6_ (5 mol%) and pyridine bis(oxazoline) ligand **L3** (10 mol%) were stirred in THF (0.5 mL) at 25 °C for 1 h, then NHC precatalyst **4a** (5 mol%), **1** (0.15 mmol), **2a** (0.1 mmol), MeOH (0.5 mmol), Na_2_CO_3_ (0.1 mmol) and THF (0.5 mL) were added to the reaction mixture and stirred for 12 h under N_2_. Diastereomeric ratio (d.r.) was determined by ^1^H NMR spectroscopic analysis. Isolated yields. The enantiomeric excess (e.e.) was determined by HPLC. ^a^7.5 mol% of **4a** was used.

The substrate scope of the asymmetric propargylic substitution reaction with respect to enals was initially investigated under optimized conditions (Fig. 2). The isatin-derived enals with different nitrogen protecting groups were found to be suitable substrates and afforded corresponding products **3ba**–**3da** in high yields and with excellent levels of stereoselectivity (>95:5 d.r. and >99% e.e. for all). The presence of either electron-withdrawing or electron-donating substituents on the isatin ring was allowed to give corresponding products **3ea**–**3ma** in good yields and with excellent diastereo- and enantioselectivities (up to >95:5 d.r. and >99% e.e.).

### Substrates scope with propargylic acetates

**Fig. 3:**
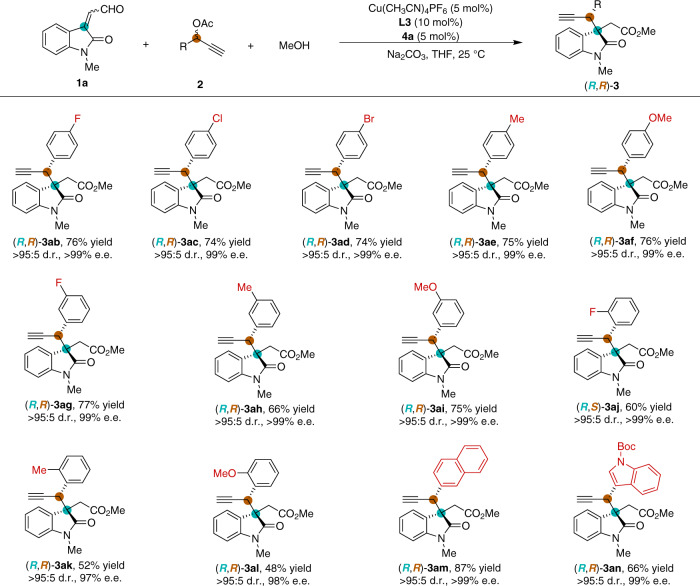
Substrate scope of propargylic acetates. Reaction conditions: Cu(CH_3_CN)_4_PF_6_ (5 mol%) and pyridine bis(oxazoline) ligand **L3** (10 mol%) were stirred in THF (0.5 mL) at 25 °C for 1 h, then NHC precatalyst **4a** (5 mol%), **1a** (0.15 mmol), **2** (0.1 mmol), MeOH (0.5 mmol), Na_2_CO_3_ (0.1 mmol) and THF (0.5 mL) were added to the reaction mixture and stirred for 12 h under N_2_. Diastereomeric ratio (d.r.) was determined by ^1^H NMR spectroscopic analysis. Isolated yields. The enantiomeric excess (e.e.) was determined by HPLC.

Substituted propargylic acetates were then examined under the optimized reaction conditions (Fig. 3). A broad range of propargylic acetates **2** bearing electron-withdrawing or electron-donating substituents on *para*- or *meta*-position of the benzene ring were well tolerated and gave the desired products **3ab**-**3ai** in high yields and excellent stereoselectivities. In addition, the presence of *ortho*-substituent at the aryl group of propargylic acetates also underwent an efficient and stereoselective coupling reaction (**3aj**-**3al**). Moreover, the asymmetric reaction of both 2-naphthyl and 3-indolyl propargyl acetates proceeded smoothly and furnished the corresponding products (**3am** and **3an**) with >95:5 d.r. and 99% e.e. Unfortunately, the other types of enals failed to undergo the desired reaction (See Supplementary Table 7 for some unsuccessful substrates).

### Diastereodivergent propargylic alkylations

**Fig. 4:**
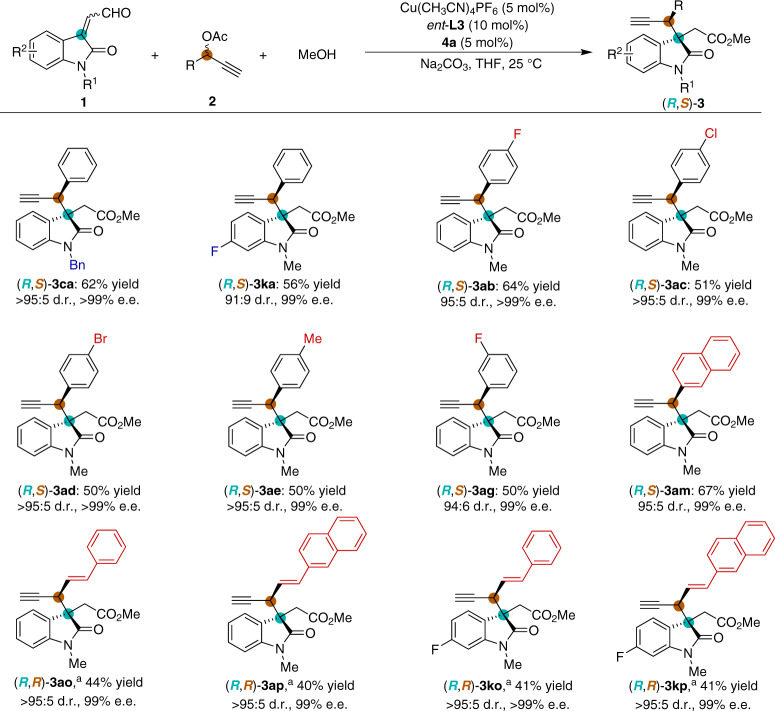
Demonstration of diastereodivergence. Reaction conditions: Cu(CH_3_CN)_4_PF_6_ (5 mol%) and pyridine bis(oxazoline) ligand *ent*-**L3** (10 mol%) were stirred in THF (0.5 mL) at 25 °C for 1 h, then NHC precatalyst **4a** (5 mol%), **1** (0.15 mmol), **2** (0.1 mmol), MeOH (0.5 mmol), Na_2_CO_3_ (0.1 mmol) and THF (0.5 mL) were added to the reaction mixture and stirred for 12 h under N_2_. Diastereomeric ratio (d.r.) was determined by ^1^H NMR spectroscopic analysis. Isolated yields. The enantiomeric excess (e.e.) was determined by HPLC. ^a^Cu(CH_3_CN)_4_PF_6_ (7.5 mol%), 2,6-bis((4 *R*,5 *S*)-4,5-diphenyl-4,5-dihydrooxazol-2-yl)pyridine **L5** (15 mol%), NHC precatalyst **4a** (7.5 mol%), **1** (0.15 mmol), styryl-substituted propargylic benzoates **2** (0.1 mmol), Na_2_CO_3_ (0.2 mmol), and DCM (1.0 mL) were used, at 15 °C.

The possibility to access diastereodivergent propargylic alkylation reaction of various isatin-derived enals **1** with propargylic acetates **2** was investigated by using a combination of chiral NHC precatalyst **4a** and the enantiomer of chiral pyridine bis(oxazoline) ligand (*ent*-**L3**) (Fig. 4). To our delight, diastereomers of **3ca**, **3ka**, **3ab**, **3ac**, **3ad**, **3ae**, **3ag**, and **3am** were all obtained in good yields and with excellent stereoselectivities under the optimal reaction conditions. Moreover, the styryl-substituted propargylic benzoates (**2o** and **2p**) were tolerated in our modified catalytic system (with Pybox ligand **L5**, see Supplementary Table 8 for details), and the alkylation products **3ao**, **3ap**, **3ko**, and **3kp** were obtained in moderate yields and with excellent stereoselectivities.

### Stereodivergent propargylic alkylation process

**Fig. 5:**
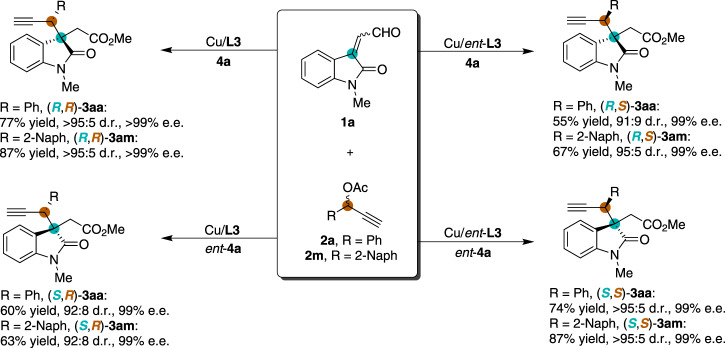
Stereodivergent synthesis of all four stereoisomers of oxindoles 3aa and 3am via cooperative NHC/Cu catalysis. Reaction conditions: Cu(CH_3_CN)_4_PF_6_ (5 mol%) and pyridine bis(oxazoline) ligand L3 or *ent*-L3 (10 mol%) were stirred in THF (0.5 mL) at 25 °C for 1 h, then NHC precatalyst 4a or *ent*-4a (5 mol%), 1a (0.15 mmol), 2 (0.1 mmol), MeOH (0.5 mmol), Na_2_CO_3_ (0.1 mmol) and THF (0.5 mL) were added to the reaction mixture and stirred for 12 h under N_2_. Diastereomeric ratio (d.r.) was determined by ^1^H NMR spectroscopic analysis. Isolated yields. The enantiomeric excess (e.e.) was determined by HPLC.

We then set out to explore the stereodivergence of the NHC/Cu cooperatively catalyzed propargylic alkylation process. As shown in Fig. 5, in the presence of the pairwise combination of NHC/Cu catalysts, both phenyl and 2-naphthyl propargylic acetates (**2a** and **2** **m**) reacted smoothly with isatin-derived enal **1a** to give all four stereoisomers of the corresponding products (**3aa** and **3am**) in good yields and with excellent diastereo- and enantioselectivities. The absolute configurations of (*R*,*R*)-**3aa** and (*R*,*S*)-**3aa** were determined by X-ray crystallography.

## Discussion

**Fig. 6:**
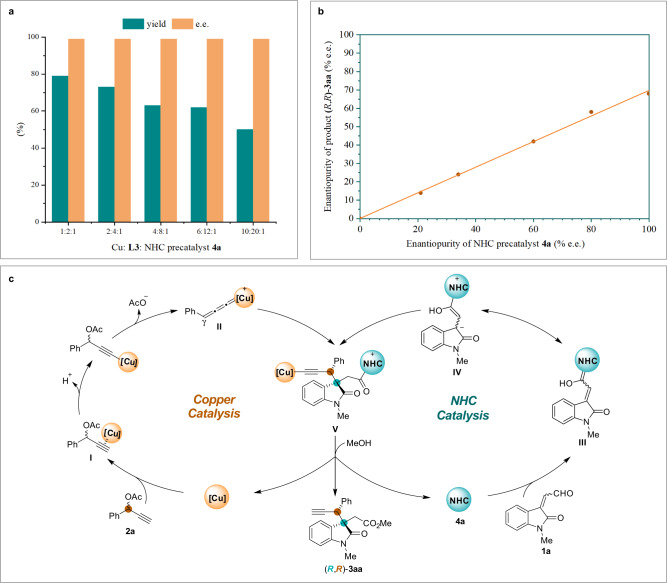
Mechanistic investigation and proposed catalytic cycles. **a** Reaction outcomes with different catalyst loadings. **b** Nonlinear experiments. **c** Proposed catalytic cycles.

To get insight into the reaction mechanism, a series of experiments on the propargylic alkylation reaction between isatin-derived enal **1a** and propargylic acetate **2a** were carried out with different catalyst ratios of Cu:Pybox **L3**:NHC **4a** ranging from 1:2:1 to 10:20:1 (Fig. 6a, see Supplementary Table 9 for details). The increase in the amount of copper catalyst did not affect the stereochemistry of the product (*R*,*R*)-**3aa** (>95:5 d.r., 99% e.e. for all), even when the amount of copper catalyst was tenfold that of the NHC catalyst (Cu:Pybox **L3**:NHC **4a** = 10:20:1). These results indicated that the catalytic performance of NHC **4a** involved in the organocatalytic cycle was not affected by the presence of excess amounts of the copper catalyst, even if the hybrid complex [Cu^I^(**L3**)(**4a**)] was formed (see Supplementary Information for ESI-MS analysis)^44,49,57,58^. Next, we searched for nonlinear effects using achiral Pybox ligand and chiral NHC precatalyst **4a** with different optical purities (see Supplementary Table 10 for details). As depicted in Fig. 6b, the linear relationship between the e.e. values of **4a** and those of the oxindole product (*R*,*R*)-**3aa** indicated that one molecule of NHC catalyst got involved in the stereochemical control events. We also carried out ^19^F NMR studies with a fluorine-substituted Pybox ligand **L6** (2,6-bis((*S*)-4-(4-fluorophenyl)-4,5-dihydrooxazol-2-yl)pyridine), Cu(CH_3_CN)_4_PF_6_, and NHC **4a** to identify the copper complex species existing in the catalytic system, and the Pybox-copper complex was the only fluorine-containing metal species detected (see Supplementary Fig. 1 for details). Aggregately, these results demonstrated that one molecule of NHC working as an organocatalyst was involved in the enantio-determining step, and the Pybox-copper complex is the reactive metal species in the catalytic system. The coordination event between NHC and copper center might exist, however, had little effect on the stereochemical control. Based on these experimental results, a plausible reaction pathway is proposed and summarized in Fig. 6c. In the copper catalytic cycle, the chiral copper complex^59–61^ interacts with the propargylic acetate **2a** and forms a copper(I) alkyne π-complex **I**. The subsequent deprotonation and elimination of an acetyl group deliver Cu−allenylidene complex **II**. Meanwhile, in the organocatalytic cycle, the addition of the NHC catalyst **4a** to the isatin-derived enal **1a** gives the Breslow intermediate **III**, which attacks at the Cγ atom of **II** and gives the corresponding Cu−acetylide complex **V**. The chiral NHC catalyst and the copper complex work cooperatively in the propargylic alkylation process and allow independent control of each chiral center, which is recognized as the key to success for achieving stereodivergent synthesis. Finally, the alcoholysis with MeOH completes the catalytic cycle to give the adduct **3aa** and regenerates both catalysts.

**Fig. 7:**
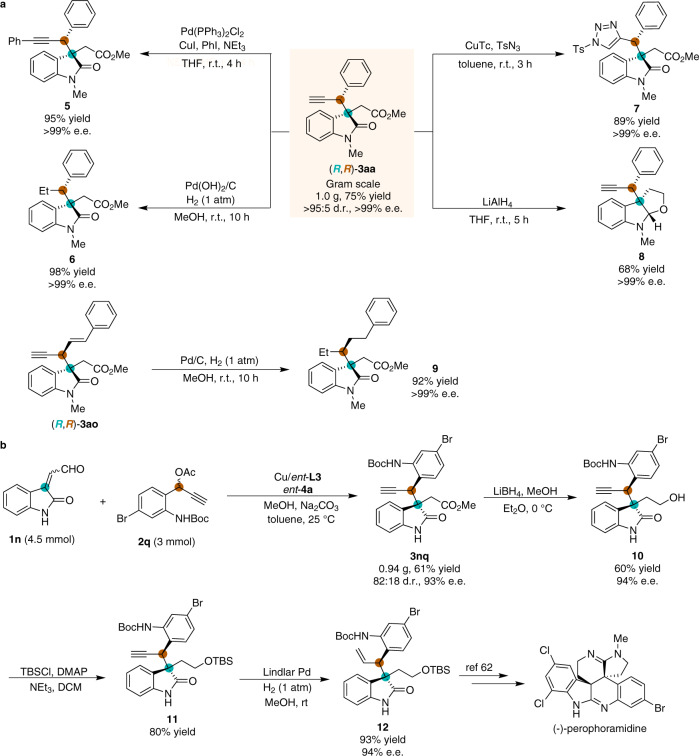
Scale-up reaction and synthetic transformations. **a** Gram-scale process and functional group transformations. **b** Preparation of synthetic precursors for (-)-perophoramidine. See Supplementary Information for experimental details.

The current process is highly reliable and scalable, and thus, a gram-scale reaction of **1a** and **2a** proceeds smoothly to generate (*R*,*R*)-**3aa** with maintained reaction efficiency and stereoselectivity in comparison with the small-scale process (Fig. 7a). The enantioenriched oxindole products are highly synthetically useful and can be elaborated to complex molecules via classical and easily operational transformations (Fig. 7a). Sonogashira coupling introduced an aryl group onto (*R*,*R*)-**3aa** to afford **5**. Hydrogenation of the alkyne group over Pd(OH)_2_/C catalyst led to an alkyl-substituted product **6** in 98% yield and > 99% e.e. Upon being treated with TsN_3_, 1,2,3-triazole substituted oxindole **7** was furnished in 89% yield and with maintained enantiopurity. Reduction of (*R*,*R*)-**3aa** with LiAlH_4_ in THF led to a furoindoline **8**. Hydrogenation of **3ao** in the presence of a catalytic amount of Pd/C led to the simultaneous reduction of the alkene and alkyne groups, providing multi alkyl-substituted product **9** in good yield and with no loss of enantiopurity. Due to the intriguing oxindole structural feature established through this method, we sought to explore its further synthetic applications toward the synthesis of related natural alkaloids (Fig. 7b). Starting from the enal **1n** and propargylic acetate **2q**, the oxindole **3nq** was obtained in good yield and stereoselectivity. Reduction of the ester group of **3nq** with LiBH_4_ furnished the corresponding primary alcohol **10** in 60% yield. The hydroxyl group of **10** was then protected with the TBS group to afford **11**. The following Lindlar reduction of **11** delivered compound **12** in 93% yield and 94% e.e., which could be applied in the total synthesis of (-)-perophoramidine^62^.

In summary, we have demonstrated that the synergistic catalysis of chiral copper complexes and NHCs can enable highly efficient stereodivergent synthesis, leading to the first stereodivergent propargylic alkylation of isatin-derived enals and propargylic acetates that provides a diverse set of oxindole derivatives bearing chiral quaternary stereocenters with a high level of enantiocontrol. All four possible stereoisomers of the resulting products containing two contiguous stereocenters are accessible by simple permutations of the enantiomers of the optimal copper complexes and NHC catalysts. The structural modulation of products works well to allow the structurally diverse synthesis of chiral functional molecules and key chiral intermediate to access (-)-perophoramidine. More importantly, the stereodivergent nucleophilic addition of chiral N-heterocyclic carbene-activated intermediates to the electrophilic Cu-allenylidenes would offer new opportunities to the field of asymmetric cooperative catalysis.

## Methods

### Materials

Unless otherwise noted, materials were purchased from commercial suppliers and used without further purification. All the solvents were treated according to general methods. Flash column chromatography was performed using 200–300 mesh silica gel. See Supplementary Methods for experimental details.

### General procedure for the synthesis of 3

To a flame-dried and N_2_-purged Schlenk tube were added Cu(CH_3_CN)_4_PF_6_ (0.005 mmol, 5 mol%) and pyridine bisoxazoline ligand **L3** (or *ent*-**L3**) (0.01 mmol, 10 mol%). The vial was sealed, purged and backfilled with N_2_ three times before adding THF (0.5 mL) at 25 °C. The resulting solution was stirred at 25 °C for 1 h. Then, isatin-derived enal **1** (0.15 mmol), NHC precatalyst **4a** (or *ent*-**4a**) (0.005 mmol, 5 mol%), Na_2_CO_3_ (0.1 mmol), MeOH (0.5 mmol) and a solution of propargylic acetate **2** (0.1 mmol) in THF (0.5 mL) were added. The resulting solution was stirred at 25 °C for 12 h and then quenched with saturated NH_4_Cl aqueous solution (5.0 mL). The resulting solution was extracted with ethyl acetate (5.0 mL × 3). The combined organic layers were dried over Na_2_SO_4_, filtered and concentrated in vacuo. The diastereomeric ratio was determined by ^1^H NMR analysis of the crude reaction mixture. The residue was purified by column chromatography on silica gel (petroleum ether: ethyl acetate = 5:1–2:1) to afford the desired product **3**. Full experimental details and characterization of new compounds can be found in the Supplementary Methods.

## Supplementary information

Supplementary Information

## Data Availability

All data generated or analyzed during this study are included in the published Article and Supplementary Information. Crystallographic data for the structures reported in this Article have been deposited at the Cambridge Crystallographic Data Centre, under deposition numbers CCDC 2094612 ((*R*,*R*)-**3aa**) and 2094609 ((*R*,*S*)-**3aa**). Copies of the data can be obtained free of charge via https://www.ccdc.cam.ac.uk/structures/.
