# Supplementary material for: Stereodivergent propargylic alkylation of enals via cooperative NHC and copper catalysis
Source: Nat Commun. 2022 Mar 15;13:1344. doi: 10.1038/s41467-022-29059-0 (PMC8924209; doi:10.1038/s41467-022-29059-0)
Supplement: Supplementary file 1 — Supplementary Information [file 41467_2022_29059_MOESM1_ESM.pdf]

Supplementary Information

Stereodivergent Propargylic Alkylation of Enals via Cooperative NHC and Copper Catalysis

Yu-Hua Wen, Zi-Jing Zhang, Shuai Li, Jin Song★ and Liu-Zhu Gong★

★*Email: gonglz@ustc.edu.cn; jill@ahu.edu.cn.*

Table of Contents

1.Supplementary Notes…………………………………………………………..…1

2. Supplementary Discussion…………………………………………………..…...2

3. Supplementary Methods……………………………..…………………………..11

4. Supplementary References…………………………………………….……...163

1. Supplementary Notes

General data:

NMR spectra were recorded on Bruker-400 MHz spectrometer or Bruker-500 MHz spectrometer. Chemical shifts (δ) are given in ppm relative to TMS. The residual solvent signals were used as references and the chemical shifts converted to the TMS scale ($CDCl_3$: δH = 7.26 ppm, δC = 77.16 ppm).

Enantiomeric excesses were measured on Waters-Alliance (2998. Photodiode Array Detector, UV detection monitored at220 nm, 230 nm, 254 nm or 270 nm). Chiralpak IA, IC, IE and IG columns were purchased from Daicel Chemical Industries, LTD.

Melting points were determined with a SWG X-4 melting apparatus. The high resolution mass spectra were recorded on a Thermo LTQ Orbitrap XL (ESI+) or a P-SIMS-Gly of Bruker DaltonicsInc (EI+). Infrared spectra were recorded on a Nicolet MX-1E FT-IR spectrometer. Optical rotations were measured at 589 nm (sodium D line) by using a Perkin-Elmer 343 polarimeter.

Materials:

All starting materials, reagents and solvents were purchased from commercial suppliers (Aldrich, Alfa, TCI, Daicel, etc.) and used as supplied unless otherwise stated. The isatin-derived enals **1**[1,2], propargylic acetates **2**[3,4], NHC precatalysts **4**[5,6], and Pybox ligand **L6**[7] were synthesized in accordance with the procedures in literatures. Tetrahydrofuran and toluene were dried over Na and distilled prior to use.

2. Supplementary Discussion

Supplementary Table 1. Effect of NHC precatalyst on the reaction[a]

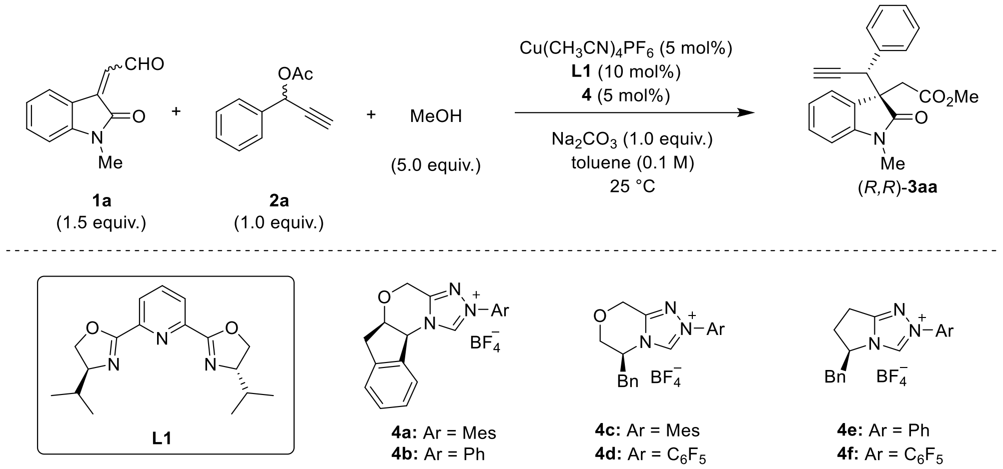

entry	4	yield (%)	d.r.	e.e. (%)
1	**4a**	70	93:7	96
2	**4b**	20	87:13	94
3	**4c**	76	92:8	92
4	**4d**	74	67:33	29
5	**4e**	22	71:29	59
6	**4f**	83	50:50	18

[a]Unless noted, reaction conditions: $Cu(CH_3CN)_4PF_6$ (5 mol%) and pyridine bisoxazoline ligand **L1** (10 mol%) were stirred in toluene (0.5 mL) at 25 ℃ for 1 h under N_2, then NHC precatalyst **4** (5 mol%), **1a** (0.15 mmol), **2a** (0.1 mmol), MeOH (0.5 mmol), Na_2CO_3 (0.1 mmol) and toluene (0.5 mL) were added to the reaction mixture and stirred at 25 ℃ for 12 h under N_2. The yield and diastereomeric ratio (d.r.) were determined by ^1H NMR spectroscopy. The enantiomeric excess (e.e.) was determined by HPLC.

Supplementary Table 2. Effect of copper catalyst on the reaction[a]

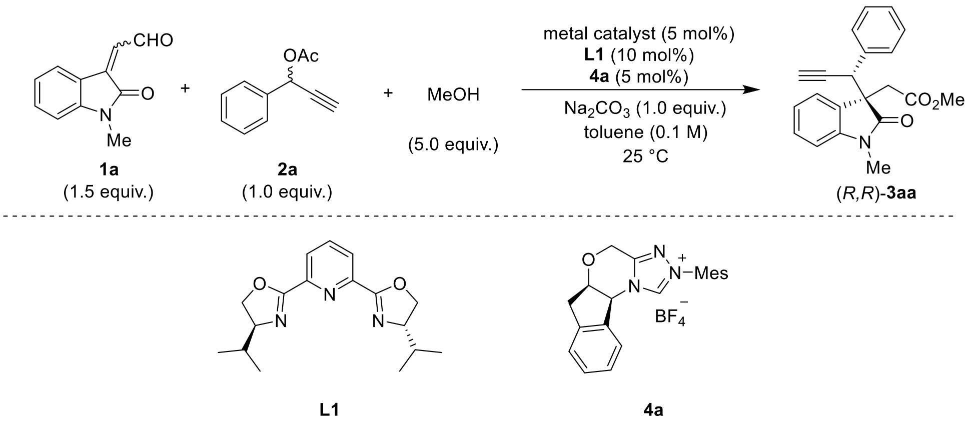

entry	metal catalyst	yield (%)	d.r.	e.e.(%)
1	$Cu(CH_3CN)_4PF_6$	70	93:7	96
2	$Cu(CH_3CN)_4BF_4$	32	75:25	88
3	CuI	<5	-	-
4	$Cu(OTf)_2$	52	88:12	94
5	$Cu(OAc)_2$	43	67:33	88

[a]Unless noted, reaction conditions: copper catalyst (5 mol%) and pyridine bisoxazoline ligand **L1** (10 mol%) were stirred in toluene (0.5 mL) at 25 ℃ for 1 h under N_2, then NHC precatalyst **4a**(5 mol%), **1a** (0.15 mmol), **2a** (0.1 mmol), MeOH (0.5 mmol), Na_2CO_3 (0.1 mmol) and toluene (0.5 mL) were added to the reaction mixture and stirred at 25 ℃ for 12 h under N_2. The yield and diastereomeric ratio (d.r.) were determined by ^1H NMR spectroscopy. The enantiomeric excess (e.e.) was determined by HPLC. n.d. = not detected.

Supplementary Table 3. Effect of ligand on the reaction[a]

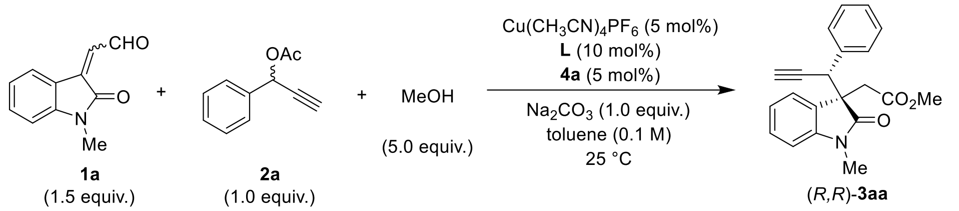

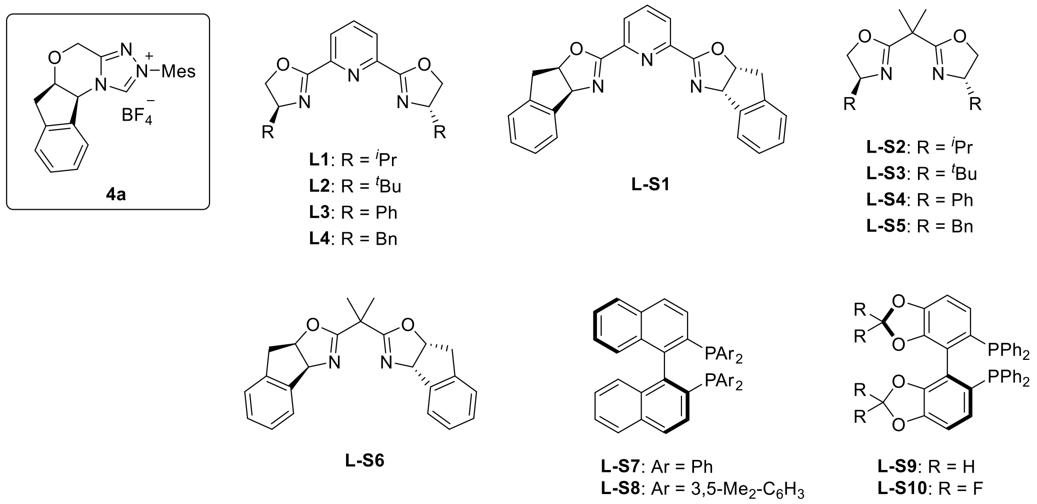

entry	**L**	yield (%)	d.r.	e.e. (%)
1	**L1**	70	93:7	96
2	**L2**	42	60:40	73
3	**L3**	51	>95:5	99
4	**L4**	39	81:19	87
5	**L-S1**	16	78:22	-
6	**L-S2**	<5	-	-
7	**L-S3**	<5	-	-
8	**L-S4**	15	78:22	90
9	**L-S5**	41	85:15	97
10	**L-S6**	15	84:16	94
11	**L-S7**	<5	-	-
12	**L-S8**	<5	-	-
13	**L-S9**	<5	-	-
14	**L-S10**	<5	-	-

[a]Unless noted, reaction conditions: $Cu(CH_3CN)_4PF_6$ (5 mol%) and ligand **L** (10 mol%) were stirred in toluene (0.5 mL) at 25 ℃ for 1 h under N_2, then NHC precatalyst **4a** (5 mol%), **1a** (0.15 mmol), **2a** (0.1 mmol), MeOH (0.5 mmol), Na_2CO_3 (0.1 mmol) and toluene (0.5 mL) were added to the reaction mixture and stirred at 25 ℃ for 12 h under N_2. The yield and diastereomeric ratio (d.r.) were determined by ^1H NMR spectroscopy. The enantiomeric excess (e.e.) was determined by HPLC.

Supplementary Table 4. Effect of base on the reaction[a]

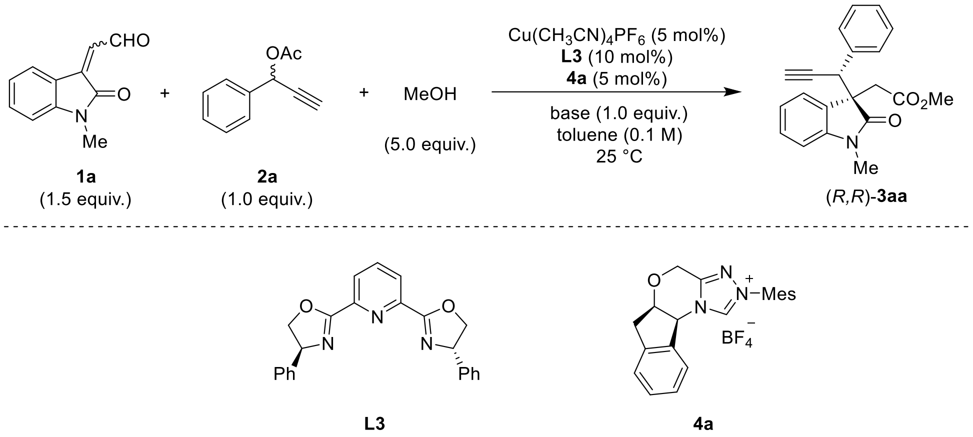

entry	base	yield (%)	d.r.	e.e. (%)
1	Na_2CO_3	51	>95:5	99
2	K_2CO_3	<5	-	-
3	Cs_2CO_3	n.d.	-	-
4	NaOAc	11	92:8	-
5	$NaHCO_3$	9	95:5	-
6	$^i Pr_2NEt$	51	93:7	96
7	NEt_3	60	95:5	98
8	DMAP	16	73:27	-
9	DABCO	30	80:20	98
10	TMEDA	44	86:14	95

[a]Unless noted, reaction conditions: $Cu(CH_3CN)_4PF_6$ (5 mol%) and pyridine bisoxazoline ligand **L3** (10 mol%) were stirred in toluene (0.5 mL) at 25 ℃ for 1 h under N_2, then NHC precatalyst **4a** (5 mol%), **1a** (0.15 mmol), **2a** (0.1 mmol), MeOH (0.5 mmol), base (0.1 mmol) and toluene (0.5 mL) were added to the reaction mixture and stirred at 25 ℃ for 12 h under N_2. The yield and diastereomeric ratio (d.r.) were determined by ^1H NMR spectroscopy. The enantiomeric excess (e.e.) was determined by HPLC. DMAP = 4-dimethylaminopyridine; DABCO = triethylene diamine; TMEDA = *N*,*N*,*N*',*N*'-tetramethylethylenediamine.

Supplementary Table 5. Effect of temperature on the reaction[a]

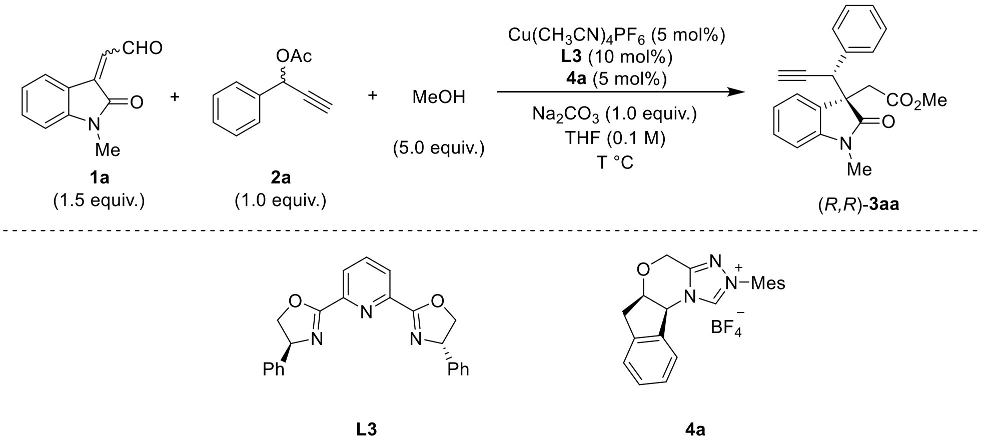

entry	T (℃)	yield (%)	d.r.	e.e. (%)
1	40	65	>95:5	99
2	30	66	>95:5	99
3	25	79	>95:5	>99
4[b]	10	64	>95:5	99
5[b]	0	32	>95:5	99

[a]Unless noted, reaction conditions: $Cu(CH_3CN)_4PF_6$ (5 mol%) and pyridine bisoxazoline ligand **L3** (10 mol%) were stirred in THF (0.5 mL) at 25 ℃ for 1 h under N_2, then NHC precatalyst **4a**(5 mol%), **1a** (0.15 mmol), **2a** (0.1 mmol), MeOH (0.5 mmol), Na_2CO_3 (0.1 mmol) and THF (0.5 mL) were added to the reaction mixture and stirred at T ℃ for 12 h under N_2. The yield and diastereomeric ratio (d.r.) were determined by ^1H NMR spectroscopy. The enantiomeric excess (e.e.) was determined by HPLC. [b]For 3 days.

Supplementary Table 6. Effect of solvent on the reaction[a]

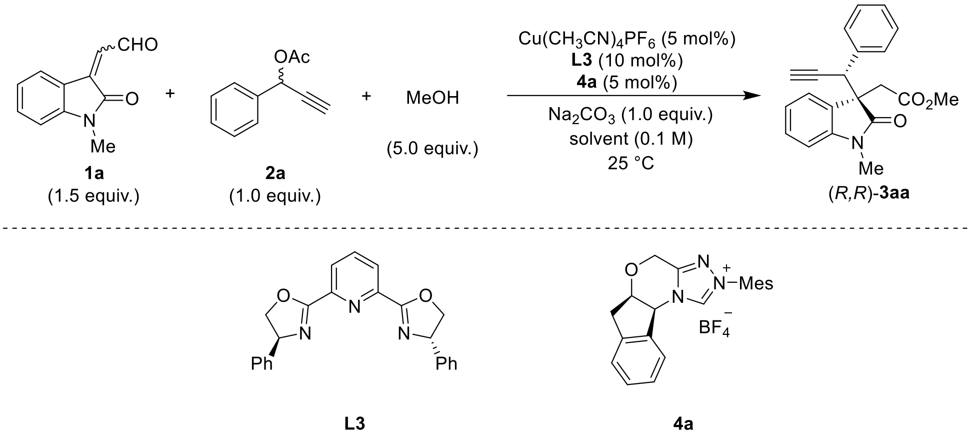

entry	solvent	yield (%)	d.r.	e.e. (%)
1	toluene	51	>95:5	99
2	DCM	47	>95:5	99
3	THF	79	>95:5	>99
4	DCE	45	>95:5	98
5	DMSO	n.d.	-	-
6	MeOH	22	73:27	84
7	EtOAc	40	>95:5	99
8	CH_3CN	53	80:20	48
9	1,4-dioxane	57	>95:5	99

[a]Unless noted, reaction conditions: $Cu(CH_3CN)_4PF_6$ (5 mol%) and pyridine bisoxazoline ligand **L3** (10 mol%) were stirred in solvent (0.5 mL) at 25 ℃ for 1 h under N_2, then NHC precatalyst **4a**(5 mol%), **1a** (0.15 mmol), **2a** (0.1 mmol), MeOH (0.5 mmol), Na_2CO_3 (0.1 mmol) and solvent (0.5 mL) were added to the reaction mixture and stirred at 25 ℃ for 12 h under N_2. The yield and diastereomeric ratio (d.r.) were determined by ^{1}H NMR spectroscopy. The enantiomeric excess (e.e.) was determined by HPLC.DCM = dichloromethane; DCE = 1,2-dichloroethane; THF =tetrahydrofuran; DMSO = dimethyl sulfoxide.

Supplementary Table 7. Unsuccessful substrates

Incompatible substratesare shown below:

Unsuccessful Electrophiles:

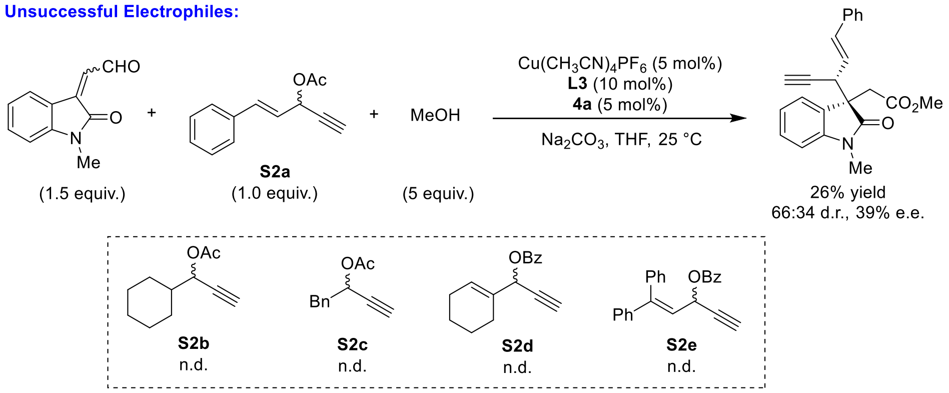

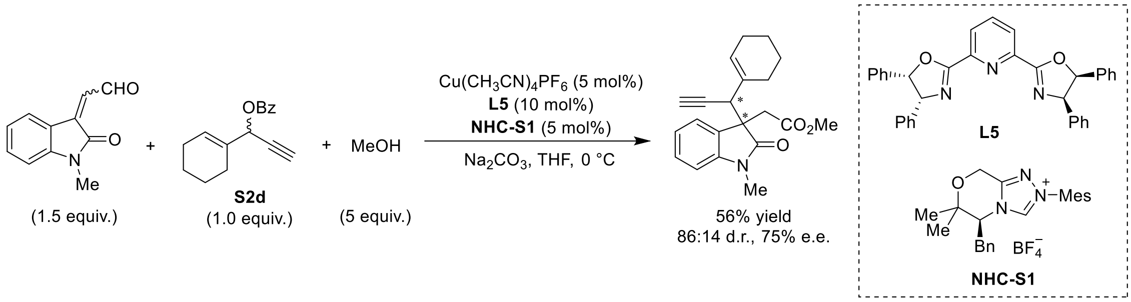

Unsuccessful Nucleophiles:

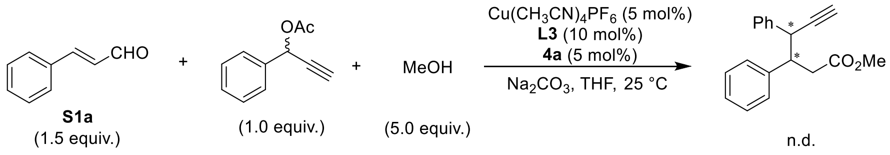

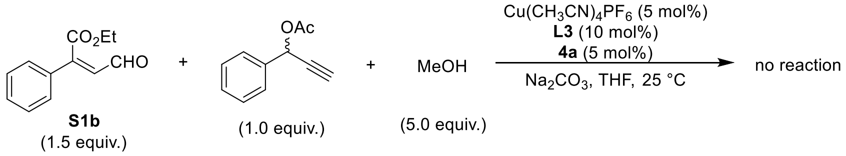

Failed to synthesize:

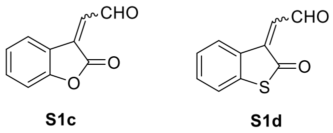

Supplementary Table 8. Condition optimization for styryl-substituted propargylic substrates[a]

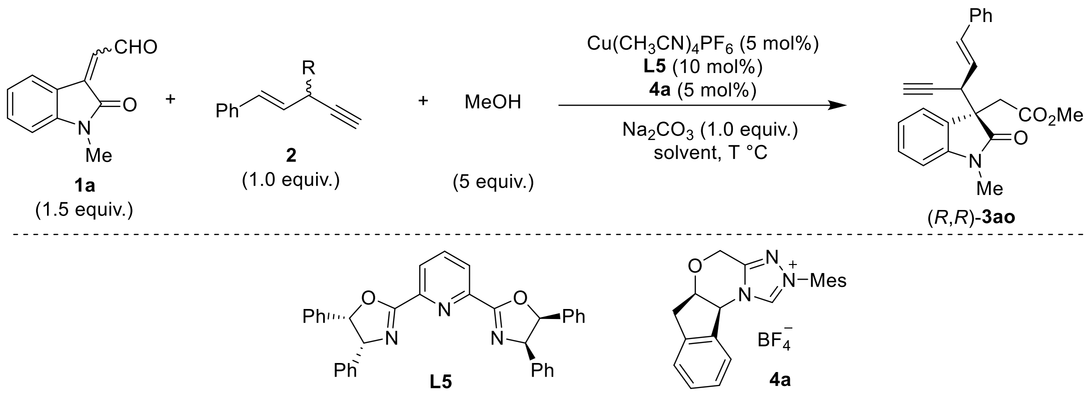

entry	R	solvent	T	yield (%)	d.r.	e.e. (%)
1	OBz	THF	25	40	86:14	85
2	OPiv	THF	25	26	68:32	64
3	$OC(O)C_6F_5$	THF	25	8	66:34	n.d.
4[b]	OBz	THF	25	32	86:14	89
5[b]	OBz	THF	35	35	81:19	89
6[b]	OBz	THF	15	34	89:11	96
7[b]	OBz	toluene	15	26	>95:5	>99
8[b]	OBz	DCM	15	32	>95:5	>99
9[c]	OBz	DCM	15	44	>95:5	99

[a]Unless noted, reaction conditions: $Cu(CH_3CN)_4PF_6$ (5 mol%) and pyridine bisoxazoline ligand **L5** (10 mol%) were stirred in solvent (0.5 mL) at 25 °C for 1 h under N_2, then NHC precatalyst **4a** (5 mol%), **1a** (0.15 mmol), **2** (0.1 mmol), MeOH (0.5 mmol), Na_2CO_3 (0.1 mmol) and solvent (0.5 mL) were added to the reaction mixture and stirred at T °C for 12 h under N_2. The yield and diastereomeric ratio (d.r.) were determined by ^1H NMR spectroscopy. The enantiomeric excess (e.e.) was determined by HPLC. DCM = dichloromethane; THF = tetrahydrofuran. [b]with **4a** (7.5 mol%). [c]with $Cu(CH_3CN)_4PF_6$ (7.5 mol%), **L5** (15 mol%), **4a** (7.5 mol%), and Na_2CO_3 (0.2 mmol).

Supplementary Table 9. Effect of Cu/Ligand/NHC ratio on the reaction[a]

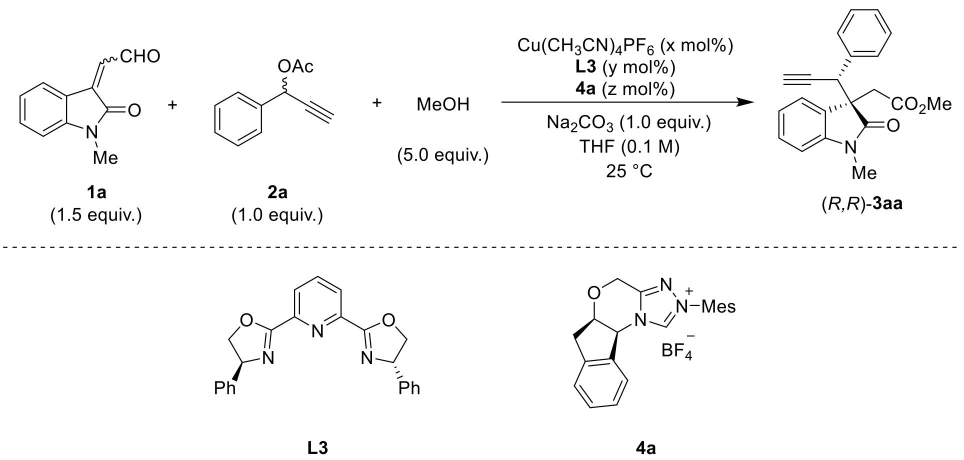

entry	x	y	z	yield (%)	d.r.	e.e. (%)
1	5	5	5	64	>95:5	>99
2	5	10	5	79	>95:5	>99
3	10	20	5	73	>95:5	>99
4	20	40	5	63	>95:5	>99
5	30	60	5	62	>95:5	>99
6	50	100	5	50	>95:5	>99

[a]Unless noted, reaction conditions: $Cu(CH_3CN)_4PF_6$ (x mol%) and pyridine bisoxazoline ligand **L3** (y mol%) were stirred in THF (0.5 mL) at 25 ℃ for 1 h under N_2, then NHC precatalyst **4a** (z mol%), **1a** (0.15 mmol), **2a** (0.1 mmol), MeOH (0.5 mmol), Na_2CO_3 (0.1 mmol) and THF (0.5 mL) were added to the reaction mixture and stirred at 25 ℃ for 12 h under N_2. The yield and diastereomeric ratio (d.r.) were determined by ^1H NMR spectroscopy. The enantiomeric excess (e.e.) was determined by HPLC.

3. Supplementary Methods

Gram Scale Reaction

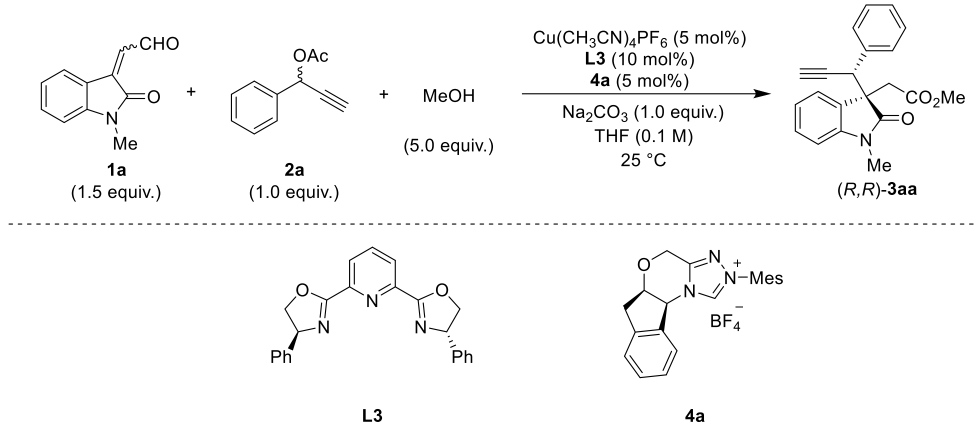

To a flame-dried and N_2-purged Schlenk tube were added $Cu(CH_3CN)_4PF_6$ (0.2 mmol, 74.5 mg) and pyridine bisoxazoline ligand **L3** (0.4 mmol, 147.8 mg). The vial was sealed, purged and backfilled with N_2 three times before adding THF (20.0 mL) at 25°C. The resulting solution was stirred at 25°C for 1 hour. Then, isatin-derived enal **1a** (6.0 mmol, 1123.2 mg), NHC precatalyst **4a** (0.2 mmol, 83.8 mg), Na_2CO_3 (4.0 mmol, 424.0 mg), MeOH (20.0 mmol, 800 μL) and a solution of propargylic acetate **2a** (4.0 mmol, 696.8 mg) in THF (20.0 mL) were added. The resulting solution was stirred at 25°C for 12 hours and then quenched with saturated NH_4Cl aqueous solution (10.0 mL). The resulting solution was extracted with ethyl acetate (15.0 mL x 3). The combined organic layers were dried over Na_2SO_4, filtered and concentrated in vacuo. The diastereomeric ratio was determined by ^{1}H NMR analysis of the crude reaction mixture. The residue was purified by column chromatography on silica gel (petroleum ether: ethyl acetate= 5:1-2:1) to afford the desired product (*R*,*R*)-**3aa** (1.0 g, 75% yield, > 95:5 d.r., >99% e.e.).

General Procedure

To a flame-dried and N_2-purged Schlenk tube were added $Cu(CH_3CN)_4PF_6$ (0.005 mmol, 5 mol%) and pyridine bisoxazoline ligand **L3** (or *ent*-**L3**) (0.01 mmol, 10 mol%). The vial was sealed, purged and backfilled with N_2 three times before adding THF (0.5 mL) at 25 °C. The resulting solution was stirred at 25 °C for 1 hour. Then, isatin-derived enal **1** (0.15 mmol), NHC precatalyst **4a** (or *ent*-**4a**) (0.005 mmol, 5 mol%), Na_2CO_3 (0.1 mmol), MeOH (0.5 mmol) and a solution of propargylic acetate **2** (0.1 mmol) in THF (0.5 mL) were added. The resulting solution was stirred at 25 °C for 12 hours and then quenched with saturated NH_4Cl aqueous solution (5.0 mL). The resulting solution was extracted with ethyl acetate (5.0 mL x 3). The combined organic layers were dried over Na_2SO_4, filtered and concentrated in vacuo. The diastereomeric ratio was determined by ^1H NMR analysis of the crude reaction mixture. The residue was purified by column chromatography on silica gel (petroleum ether: ethyl acetate = 5:1-2:1) to afford the desired product **3**.

Characterization Data of Products

Methyl 2-((*R*)-1-methyl-2-oxo-3-((*R*)-1-phenylprop-2-yn-1-yl)indolin-3-yl)acetate ((*R*,*R*)-3aa)

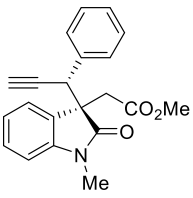

White solid, m.p. 73-75 °C; 77% yield; >95:5 d.r.; **^{1}H NMR (400 MHz, $CDCl_3$)** δ 7.30 – 7.18 (m, 4H), 7.15 – 7.11 (m, 2H), 6.97 (td, *J* = 7.5, 1.0 Hz, 1H), 6.85 (dd, *J* = 7.4, 1.3 Hz, 1H), 6.71 (d, *J* = 7.7 Hz, 1H), 4.16 (d, *J* = 2.6 Hz, 1H), 3.42 (s, 3H), 3.28 (d, *J* = 16.5 Hz, 1H), 3.12 (s, 3H), 2.89 (d, *J* = 16.5 Hz, 1H), 2.20 (d, *J* = 2.6 Hz, 1H). **^{13}C NMR (126 MHz, $CDCl_3$)** δ 177.4, 170.1, 145.1, 134.9, 129.9, 128.9, 128.2, 128.0, 127.9, 124.1, 121.8, 107.9, 81.0, 73.2, 53.1, 51.8, 45.0, 39.2, 26.3. **IR** (KBr, cm^{-1}) *γ* 3291, 3058, 3029, 2952, 2925, 2853, 1715, 1613, 1494, 1471, 1377, 1354, 1262, 1198, 1176, 1091, 1028, 752, 702, 543, 489. **HRMS** (ESI) m/z $(M+Na)^+$: calculated for $(C_{21}H_{19}NO_3Na)^+$: 356.1257, found: 356.1270; **$[\alpha]^{20}_D$** = +37.4 (*c* = 0.50, $CHCl_3$); The product was analyzed by HPLC to determine the enantiomeric excess: >99% e.e. (CHIRALPAK IG, hexane/*i*-PrOH = 70/30, flow rate: 1.0 mL/min, T = 30 ℃, 230 nm), t_R (major) = 11.26 min, t_R (minor) = 16.01 min.

Methyl 2-((*R*)-1-methyl-2-oxo-3-((*S*)-1-phenylprop-2-yn-1-yl)indolin-3-yl)acetate ((*R*,*S*)-3aa)

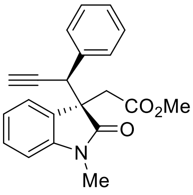

White solid, m.p. 66 °C; 55% yield; 91:9 d.r.; **^{1}H NMR (500 MHz, $CDCl_3$)** δ 7.64 (dd, *J* = 7.5, 1.3 Hz, 1H), 7.21 (td, *J* = 7.8, 1.3 Hz, 1H), 7.10 – 7.02 (m, 2H), 7.01 – 6.95 (m, 2H), 6.86 – 6.81 (m, 2H), 6.48 – 6.43 (m, 1H), 4.13 (d, *J* = 2.6 Hz, 1H), 3.58 (d, *J* = 16.6 Hz, 1H), 3.45 (s, 3H), 3.28 (d, *J* = 16.7 Hz, 1H), 2.77 (s, 3H), 2.51 (d, *J* = 2.6 Hz, 1H). **^{13}C NMR (126 MHz, $CDCl_3$)** δ 177.0, 170.3, 144.4, 134.5, 128.9, 128.8, 127.8, 127.7, 127.3, 123.9, 122.0, 107.7, 82.3, 74.2, 53.6, 51.8, 45.1, 39.7, 25.9. **IR** (KBr, cm^{-1}) *γ* 3289, 3058, 3031, 2924, 2853, 1742, 1713, 1613, 1494, 1471, 1377, 1355, 1255, 1199, 1177, 1092, 1028, 752, 699, 683, 543, 487. **HRMS** (ESI) m/z $(M+Na)^+$: calculated for $(C_{21}H_{19}NO_3Na)^+$: 356.1257, found: 356.1257; **$[\alpha]^{20}_D$** = -51.2 (*c* = 0.59, $CHCl_3$); The product was analyzed by HPLC to determine the enantiomeric excess: 99% e.e. (CHIRALPAK IG, hexane/*i*-PrOH = 70/30, flow rate: 1.0 mL/min, T = 30 ℃, 270

nm), t_R (major) = 8.74 min, t_R (minor) = 10.81 min.

Methyl 2-((*S*)-1-methyl-2-oxo-3-((*S*)-1-phenylprop-2-yn-1-yl)indolin-3-yl)acetate ((*S*,*S*)-3aa)

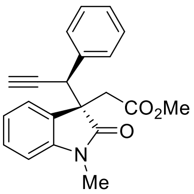

White solid; 74% yield; >95:5 d.r.; Spectral data were in agreement with those of the enantiomer reported above. **HRMS** (ESI) m/z $(M+Na)^+$: calculated for $(C_{21}H_{19}NO_3Na)^+$: 356.1257, found: 356.1255; $[\alpha]^{20}_D$ = -40.1 (*c* = 0.48, $CHCl_3$); The product was analyzed by HPLC to determine the enantiomeric excess: >99% e.e. (CHIRALPAK IG, hexane/*i*-PrOH = 70/30, flow rate: 1.0 mL/min, T = 30 ℃, 230 nm), t_R (minor) = 11.46 min, t_R (major) = 15.42 min.

Methyl 2-((*S*)-1-methyl-2-oxo-3-((*R*)-1-phenylprop-2-yn-1-yl)indolin-3-yl)acetate ((*S*,*R*)-3aa)

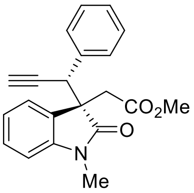

White solid; 60% yield; 92:8 d.r.; Spectral data were in agreement with those of the enantiomer reported above. **HRMS** (ESI) m/z $(M+Na)^+$: calculated for $(C_{21}H_{19}NO_3Na)^+$: 356.1257, found: 356.1263; $[\alpha]^{20}_D$ = +53.7 (*c* = 0.25, $CHCl_3$); The product was analyzed by HPLC to determine the enantiomeric excess: 99% e.e. (CHIRALPAK IG, hexane/*i*-PrOH = 70/30, flow rate: 1.0 mL/min, T = 30 ℃, 270 nm), t_R (minor) = 9.30 min, t_R (major) = 10.79 min.

Methyl 2-((*R*)-1-ethyl-2-oxo-3-((*R*)-1-phenylprop-2-yn-1-yl)indolin-3-yl)acetate ((*R*,*R*)-3ba)

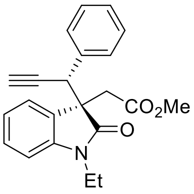

Yellow solid, m.p. 91-92 °C; 79% yield; >95:5 d.r.; **^{1}H NMR (500 MHz, $CDCl_3$)** δ 7.27 – 7.24 (m, 1H), 7.24 – 7.18 (m, 3H), 7.17 – 7.11 (m, 2H), 6.97 (td, *J* = 7.5, 1.0 Hz, 1H), 6.90 (dd, *J* = 7.5, 1.4 Hz, 1H), 6.73 (d, *J* = 7.8 Hz, 1H), 4.18 (d, *J* = 2.6 Hz, 1H), 3.78 (dq, *J* = 14.5, 7.3 Hz, 1H), 3.59 (dq, *J* = 14.3, 7.2 Hz, 1H), 3.40 (s, 3H), 3.27 (d, *J* = 16.4 Hz, 1H), 2.90 (d, *J* = 16.4 Hz, 1H), 2.21 (d, *J* = 2.6 Hz, 1H), 1.11 (t, *J* = 7.2 Hz, 3H). **^{13}C NMR (126 MHz, $CDCl_3$)** δ 176.8, 170.1, 144.2, 135.0, 129.9, 128.8, 128.5, 128.0, 127.9, 124.4, 121.6, 108.0, 81.1, 73.4, 52.7, 51.7, 44.9, 39.5, 34.7, 12.2. **IR** (KBr,

cm^{-1}) γ 3299, 2961, 2926, 2853, 1712, 1612, 1489, 1467, 1369, 1261, 1201, 1174, 1135, 1098, 1022, 799, 753, 700. **HRMS** (ESI) m/z (M+Na)$^+$: calculated for ($C_{22}H_{21}NO_3Na$)$^+$: 370.1414, found: 370.1417; **[α]^{20}$_D$** = +35.7 (*c* = 0.53, $CHCl_3$); The product was analyzed by HPLC to determine the enantiomeric excess: >99% e.e. (CHIRALPAK IG, hexane/*i*-PrOH = 70/30, flow rate: 1.0 mL/min, T = 30 ℃, 254 nm), t_R (major) = 10.57 min, t_R (minor) = 16.33 min.

Methyl 2-((*R*)-1-benzyl-2-oxo-3-((*R*)-1-phenylprop-2-yn-1-yl)indolin-3-yl)acetate ((*R*,*R*)-3ca)

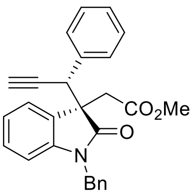

Yellow solid, m.p. 93 °C; 81% yield; >95:5 d.r.; **^{1}H NMR (500 MHz, $CDCl_3$)** δ 7.32 – 7.11 (m, 11H), 6.99 – 6.91 (m, 2H), 6.58 (d, *J* = 7.8 Hz, 1H), 4.88 (d, *J* = 15.8 Hz, 1H), 4.77 (d, *J* = 15.8 Hz, 1H), 4.25 (d, *J* = 2.7 Hz, 1H), 3.39 (s, 3H), 3.36 (d, *J* = 16.4 Hz, 1H), 2.96 (d, *J* = 16.4 Hz, 1H), 2.18 (d, *J* = 2.6 Hz, 1H). **^{13}C NMR (126 MHz, $CDCl_3$)** δ 177.4, 170.0, 144.4, 135.9, 135.0, 130.0, 128.8, 128.6, 128.3, 128.1, 128.0, 127.7, 127.5, 124.2, 121.9, 109.1, 81.2, 73.6, 52.9, 51.8, 44.9, 44.3, 39.9. **IR** (KBr, cm^{-1}) γ 3297, 3029, 2954, 2924, 1715, 1613, 1488, 1467, 1454, 1355, 1261, 1197, 1080, 1173, 1014, 800, 753, 699. **HRMS** (ESI) m/z (M+Na)$^+$: calculated for ($C_{27}H_{23}NO_3Na$)$^+$: 432.1570, found: 432.1577; **[α]^{20}$_D$** = +29.9 (*c* = 0.60, $CHCl_3$); The product was analyzed by HPLC to determine the enantiomeric excess: >99% e.e. (CHIRALPAK IG, hexane/*i*-PrOH = 70/30, flow rate: 1.0 mL/min, T = 30 ℃, 254 nm), t_R (major) = 18.93 min, t_R (minor) = 25.23 min.

Methyl 2-((*R*)-2-oxo-1-phenyl-3-((*R*)-1-phenylprop-2-yn-1-yl)indolin-3-yl)acetate ((*R*,*R*)-3da)

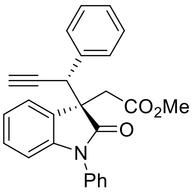

Yellow solid, m.p. 130 °C; 73% yield; >95:5 d.r.; **^{1}H NMR (500 MHz, $CDCl_3$)** δ 7.48 (t, *J* = 7.8 Hz, 2H), 7.41 – 7.36 (m, 1H), 7.32 – 7.22 (m, 5H), 7.21 – 7.14 (m, 3H), 7.01 (td, *J* = 7.5, 1.0 Hz, 1H), 6.93 (dd, *J* = 7.5, 1.3 Hz, 1H), 6.64 (d, *J* = 7.8 Hz, 1H), 4.24 (d, *J* = 2.6 Hz, 1H), 3.47 (s, 3H), 3.46 (d, *J* = 16.7 Hz, 1H), 2.98 (d, *J* = 16.6 Hz, 1H), 2.31 (d, *J* = 2.6 Hz, 1H). **^{13}C NMR (126 MHz, $CDCl_3$)** δ 176.7, 170.1, 145.3, 134.9, 134.7, 130.0, 129.6, 128.8, 128.1, 128.1, 128.0, 126.8, 124.2, 122.2, 109.1, 81.0, 77.4, 73.7,

53.0, 51.9, 45.3, 39.8. **IR** (KBr, cm^{-1}) γ 3301, 3011, 2954, 2923, 2853, 1723, 1611, 1595, 1503, 1465, 1377, 1261, 1200, 1174, 1114, 1027, 800, 752, 700, 594. **HRMS** (ESI) m/z $(M+Na)^+$: calculated for $(C_{26}H_{21}NO_3Na)^+$: 418.1414, found: 418.1425; $[\alpha]^{20}_D$ = +1.9 (*c* = 0.55, $CHCl_3$); The product was analyzed by HPLC to determine the enantiomeric excess: >99% e.e. (CHIRALPAK IE, hexane/*i*-PrOH = 70/30, flow rate: 1.0 mL/min, T = 30 ℃, 254 nm), t_R (major) = 16.91 min, t_R (minor) = 32.05 min.

Methyl 2-((*R*)-4-fluoro-1-methyl-2-oxo-3-((*R*)-1-phenylprop-2-yn-1-yl)indolin-3-yl)acetate ((*R,R*)-3ea)

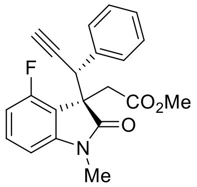

Yellow solid, m.p. 97 °C; 60% yield; >95:5 d.r.; **^{1}H NMR (400 MHz, $CDCl_3$)** δ 7.19 – 7.01 (m, 6H), 6.67 (ddd, *J* = 9.3, 8.4, 0.7 Hz, 1H), 6.30 (dd, *J* = 7.8, 0.7 Hz, 1H), 4.21 (d, *J* = 2.7 Hz, 1H), 3.74 (d, *J* = 17.0 Hz, 1H), 3.48 (s, 3H), 3.27 (d, *J* = 17.0 Hz, 1H), 2.94 (s, 3H), 2.45 (d, *J* = 2.6 Hz, 1H). **^{13}C NMR (101 MHz, $CDCl_3$)** δ 175.6, 170.5, 158.8 (d, *J* = 245.6 Hz), 146.5 (d, *J* = 9.6 Hz), 134.9, 130.7 (d, *J* = 8.8 Hz), 128.7, 127.9, 127.7, 114.3 (d, *J* = 18.8 Hz), 109.6 (d, *J* = 20.8 Hz), 104.0 (d, *J* = 2.9 Hz), 80. 6, 74.4, 53.3 (d, *J* = 3.0 Hz), 51.9, 43.8, 38.5, 26.4. **^{19}F NMR (376 MHz, $CDCl_3$)** δ -120.06. **IR** (KBr, cm^{-1}) γ 3293, 2921, 2851, 1723, 1629, 1477, 1455, 1358, 1260, 1235, 1200, 1147, 1050, 776, 701, 577, 542. **HRMS** (ESI) m/z $(M+Na)^+$: calculated for $(C_{21}H_{18}FNO_3Na)^+$: 374.1163, found: 374.1171; $[\alpha]^{20}_D$ = +70.7 (*c* = 0.39, $CHCl_3$); The product was analyzed by HPLC to determine the enantiomeric excess: 99% e.e. (CHIRALPAK IG, hexane/*i*-PrOH = 70/30, flow rate: 1.0 mL/min, T = 30 ℃, 254 nm), t_R (major) = 8.00 min, t_R (minor) = 9.23 min.

Methyl2-((*R*)-5-fluoro-1-methyl-2-oxo-3-((*R*)-1-phenylprop-2-yn-1-yl)indolin-3-yl)acetate ((*R,R*)-3fa)

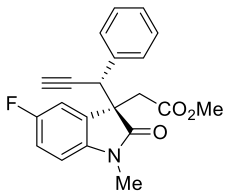

White solid, m.p. 84 °C; 50% yield; >95:5 d.r.; **^{1}H NMR (400 MHz, $CDCl_3$)** δ 7.32 – 7.23 (m, 3H), 7.19 – 7.13 (m, 2H), 6.97 (ddd, *J* = 9.3, 8.5, 2.6 Hz, 1H), 6.65 (dd, *J* = 8.5, 4.2 Hz, 1H), 6.61 (dd, *J* = 8.1, 2.6 Hz, 1H), 4.15 (d, *J* = 2.6 Hz, 1H), 3.45 (s, 3H), 3.27 (d, *J* = 16.9 Hz, 1H), 3.12 (s, 3H), 2.85 (d, *J* = 16.8 Hz, 1H), 2.20 (d, *J* = 2.6 Hz,

1H). **^{13}C NMR (126 MHz, $CDCl_3$)** δ 177.1, 170.0, 158.6 (d, *J* = 239.9 Hz), 141.2, 134.5, 129.9 (d, *J* = 8.3 Hz), 129.8, 128.3, 128.0, 115.4 (d, *J* = 23.3 Hz), 112.2 (d, *J* = 25.2 Hz), 108.2 (d, *J* = 8.2 Hz), 80.6, 73.4, 53.4 (d, *J* = 1.9 Hz), 52.0, 44.8, 39.0, 26.5. **^{19}F NMR (471 MHz, $CDCl_3$)** δ -121.05. **IR** (KBr, cm^{-1}) *γ* 3303, 3009, 2956, 2922, 2852, 1715, 1659, 1625, 1496, 1469, 1359, 1262, 1200, 1177, 1111, 1023, 811, 755, 701, 664, 561. **HRMS** (ESI) m/z $(M+Na)^+$: calculated for $(C_{21}H_{18}FNO_3Na)^+$: 374.1163, found: 374.1179; **$[\alpha]^{20}_D$** = +29.6 (*c* = 0.22, $CHCl_3$); The product was analyzed by HPLC to determine the enantiomeric excess: >99% e.e. (CHIRALPAK IG, hexane/*i*-PrOH = 70/30, flow rate: 1.0 mL/min, T = 30 ℃, 254 nm), t_R (major) = 9.99 min, t_R (minor) = 18.36 min.

Methyl 2-((*R*)-5-chloro-1-methyl-2-oxo-3-((*R*)-1-phenylprop-2-yn-1-yl)indolin-3-yl)acetate ((*R,R*)-3ga)

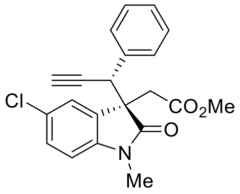

Yellow oil; 84% yield; >95:5 d.r.; **^{1}H NMR (500 MHz, $CDCl_3$)** δ 7.30 – 7.21 (m, 4H), 7.18 – 7.12 (m, 2H), 6.79 (d, *J* = 2.1 Hz, 1H), 6.65 (d, *J* = 8.3 Hz, 1H), 4.13 (d, *J* = 2.6 Hz, 1H), 3.46 (s, 3H), 3.28 (d, *J* = 16.9 Hz, 1H), 3.12 (s, 3H), 2.85 (d, *J* = 17.0 Hz, 1H), 2.21 (d, *J* = 2.6 Hz, 1H). **^{13}C NMR (126 MHz, $CDCl_3$)** δ 177.0, 169.9, 143.8, 134.4, 130.0, 129.8, 128.8, 128.3, 128.1, 127.0, 124.6, 108.7, 80.5, 73.5, 53.2, 52.0, 44.9, 39.0, 26.4. **IR** (KBr, cm^{-1}) *γ* 3296, 3029, 2957, 2925, 1717, 1610, 1492, 1454, 1358, 1261, 1201, 1177, 1100, 1023, 809, 750, 702. **HRMS** (ESI) m/z $(M+Na)^+$: calculated for $(C_{21}H_{18}ClNO_3Na)^+$: 390.0867, found: 390.0874; **$[\alpha]^{20}_D$** = +70.4 (*c* = 0.58, $CHCl_3$); The product was analyzed by HPLC to determine the enantiomeric excess: >99% e.e. (CHIRALPAK IG, hexane/*i*-PrOH = 70/30, flow rate: 1.0 mL/min, T = 30 ℃, 254nm), t_R (major) = 9.18 min, t_R (minor) = 15.87 min.

Methyl 2-((*R*)-5-bromo-1-methyl-2-oxo-3-((*R*)-1-phenylprop-2-yn-1-yl)indolin-3-yl)acetate ((*R,R*)-3ha)

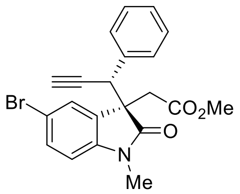

Yellow oil; 81% yield; >95:5 d.r.; **^{1}H NMR (500 MHz, $CDCl_3$)** δ 7.39 (dd, *J* = 8.2, 2.0 Hz, 1H), 7.30 – 7.24 (m, 3H), 7.17 – 7.11 (m, 2H), 6.91 (d, *J* = 2.0 Hz, 1H), 6.61 (d, *J* = 8.3 Hz, 1H), 4.12

(d, J = 2.6 Hz, 1H), 3.46 (s, 3H), 3.28 (d, J = 17.0 Hz, 1H), 3.11 (s, 3H), 2.84 (d, J = 17.0 Hz, 1H), 2.20 (d, J = 2.5 Hz, 1H). **^{13}C NMR (126 MHz, CDCl$_3$)** δ 176.9, 169.9, 144.3, 134.4, 131.7, 130.3, 129.8, 128.3, 128.1, 127.3, 114.3, 109.3, 80.5, 73.5, 53.1, 52.0, 44.9, 39.0, 26.4. **IR** (KBr, cm^{-1}) *γ* 3296, 3028, 2952, 2925, 2853, 1715, 1608, 1491, 1366, 1204, 1177, 1099, 1024, 809, 750, 702. **HRMS** (ESI) m/z (M+Na)$^+$: calculated for $(C_{21}H_{18}BrNO_3Na)^+$: 434.0362, found: 434.0368; **[α]$^{20}_{D}$** = +76.0 (*c* = 0.60, CHCl$_3$); The product was analyzed by HPLC to determine the enantiomeric excess: >99% e.e. (CHIRALPAK IG, hexane/*i*-PrOH = 70/30, flow rate: 1.0 mL/min, T = 30 ℃, 254 nm), t_R (major) = 9.52 min, t_R (minor) = 14.99 min.

Methyl 2-((*R*)-1,5-dimethyl-2-oxo-3-((*R*)-1-phenylprop-2-yn-1-yl)indolin-3-yl) acetate ((*R*,*R*)-3ia)

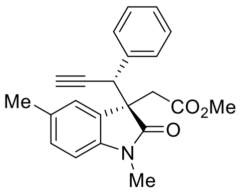

Yellow oil; 79% yield; >95:5 d.r.; **^{1}H NMR (500 MHz, CDCl$_3$)** δ 7.27 – 7.20 (m, 3H), 7.16 – 7.12 (m, 2H), 7.07 – 7.03 (m, 1H), 6.63 (d, J = 1.7 Hz, 1H), 6.61 (d, J = 7.9 Hz, 1H), 4.15 (d, J = 2.6 Hz, 1H), 3.43 (s, 3H), 3.27 (d, J = 16.7 Hz, 1H), 3.10 (s, 3H), 2.86 (d, J = 16.7 Hz, 1H), 2.28 (s, 3H), 2.19 (d, J = 2.6 Hz, 1H). **^{13}C NMR (126 MHz, CDCl$_3$)** δ 177.3, 170.1, 142.7, 134.9, 131.1, 129.9, 129.1, 128.1, 128.0, 127.8, 125.0, 107.5, 81.0, 73.1, 53.0, 51.8, 44.9, 39.1, 26.3, 21.3. **IR** (KBr, cm^{-1}) *γ* 3288, 3028, 2923, 1740, 1715, 1621, 1603, 1501, 1454, 1358, 1199, 1175, 1096, 1032, 809, 754, 702, 661. **HRMS** (ESI) m/z (M+Na)$^+$: calculated for $(C_{22}H_{21}NO_3Na)^+$: 370.1414, found: 370.1417; **[α]$^{20}_{D}$** = +69.5 (*c* = 0.52, CHCl$_3$); The product was analyzed by HPLC to determine the enantiomeric excess: 99% e.e. (CHIRALPAK IG, hexane/*i*-PrOH = 70/30, flow rate: 1.0 mL/min, T = 30 ℃, 254 nm), t_R (major) = 10.19 min, t_R (minor) = 16.93 min.

Methyl 2-((*R*)-5-methoxy-1-methyl-2-oxo-3-((*R*)-1-phenylprop-2-yn-1-yl)indolin-3-yl)acetate ((*R*,*R*)-3ja)

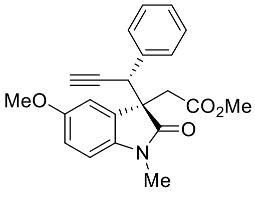

Yellow oil; 69% yield; >95:5 d.r.; **^{1}H NMR (500 MHz, CDCl$_3$)** δ 7.29 – 7.22 (m, 3H), 7.17 (dd, J = 6.3, 2.7 Hz, 2H), 6.78 (dd, J = 8.5, 2.5 Hz, 1H), 6.62 (d, J = 8.4 Hz, 1H), 6.45 (d, J = 2.5 Hz,

1H), 4.16 (d, J = 2.5 Hz, 1H), 3.72 (s, 3H), 3.43 (s, 3H), 3.25 (d, J = 16.6 Hz, 1H), 3.10 (s, 3H), 2.84 (d, J = 16.6 Hz, 1H), 2.19 (d, J = 2.5 Hz, 1H). **^{13}C NMR (126 MHz, $CDCl_3$)** δ 177.1, 170.1, 155.3, 138.8, 134.9, 130.0, 129.5, 128.1, 127.9, 113.1, 111.8, 108.1, 81.0, 73.1, 55.8, 53.4, 51.8, 44.9, 39.2, 26.4. **IR** (KBr, cm^{-1}) γ 3288, 3004, 2924, 2852, 1739, 1709, 1601, 1498, 1367, 1288, 1234, 1201, 1175, 1115, 1033, 888, 807, 753, 701. **HRMS** (ESI) m/z $(M+Na)^+$: calculated for $(C_{22}H_{21}NO_4Na)^+$: 386.1363, found: 386.1369; **$[\alpha]^{20}_D$** = +5.0 (c = 0.43, $CHCl_3$); The product was analyzed by HPLC to determine the enantiomeric excess: >99% e.e. (CHIRALPAK IA, hexane/i-PrOH = 85/15, flow rate: 1.0 mL/min, T = 30 ℃, 254 nm), t_R (minor) = 12.89 min, t_R (major) = 13.84 min.

Methyl 2-((*R*)-6-fluoro-1-methyl-2-oxo-3-((*R*)-1-phenylprop-2-yn-1-yl)indolin-3-yl)acetate ((*R*,*R*)-3ka)

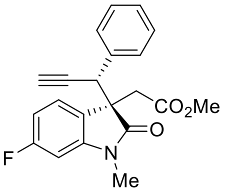

White solid; 88% yield; >95:5 d.r.; **^{1}H NMR (400 MHz, $CDCl_3$)** δ 7.29 – 7.22 (m, 3H), 7.16 – 7.11 (m, 2H), 6.74 (dd, J = 8.2, 5.4 Hz, 1H), 6.64 (ddd, J = 9.4, 8.2, 2.3 Hz, 1H), 6.47 (dd, J = 8.9, 2.3 Hz, 1H), 4.12 (d, J = 2.6 Hz, 1H), 3.43 (s, 3H), 3.26 (d, J = 16.7 Hz, 1H), 3.12 (s, 3H), 2.84 (d, J = 16.7 Hz, 1H), 2.20 (d, J = 2.6 Hz, 1H). **^{13}C NMR (101 MHz, $CDCl_3$)** δ 177.8, 170.0, 163.5 (d, J = 245.4 Hz), 146.7 (d, J = 11.7 Hz), 134.7, 129.8, 128.2, 128.0, 125.1 (d, J = 9.9 Hz), 123.4 (d, J = 2.9 Hz), 107.8 (d, J = 22.4 Hz), 96.8 (d, J = 27.5 Hz), 80.7, 73.3, 52.7, 51.9, 44.9, 39.1, 26.4. **^{19}F NMR (376 MHz, $CDCl_3$)** δ -111.19. **IR** (KBr, cm^{-1}) γ 3301, 3029, 2953, 2926, 2853, 1723, 1615, 1504, 1454, 1382, 1198, 1178, 1085, 837, 755, 702. **HRMS** (ESI) m/z $(M+Na)^+$: calculated for $(C_{21}H_{18}FNO_3Na)^+$: 374.1163, found: 374.1171; **$[\alpha]^{20}_D$** = +31.5 (c = 0.49, $CHCl_3$); The product was analyzed by HPLC to determine the enantiomeric excess: >99% e.e. (CHIRALPAK IG, hexane/i-PrOH = 70/30, flow rate: 1.0 mL/min, T = 30 ℃, 254 nm), t_R (major) = 9.35 min, t_R (minor) = 13.58 min.

Methyl 2-((*R*)-7-fluoro-1-methyl-2-oxo-3-((*R*)-1-phenylprop-2-yn-1-yl)indolin-3-yl)acetate ((*R*,*R*)-3la)

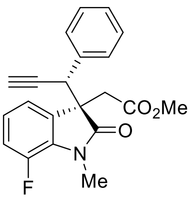

White solid, m.p. 127-129 °C; 78% yield; >95:5 d.r.; **^{1}H NMR (400 MHz, $CDCl_3$)** δ 7.31 – 7.20 (m, 3H), 7.14 – 7.08 (m, 2H), 6.98 (ddd, *J* = 11.5, 8.4, 1.1 Hz, 1H), 6.89 (ddd, *J* = 8.4, 7.4, 4.5 Hz, 1H), 6.60 (dd, *J* = 7.3, 1.1 Hz, 1H), 4.11 (d, *J* = 2.6 Hz, 1H), 3.44 (s, 3H), 3.33 (d, *J* = 2.7 Hz, 3H), 3.32 (d, *J* = 16.8 Hz, 1H), 2.86 (d, *J* = 16.8 Hz, 1H), 2.25 (d, *J* = 2.6 Hz, 1H). **^{13}C NMR (101 MHz, $CDCl_3$)** δ 177.1, 170.0, 147.6 (d, *J* = 243.3 Hz), 134.5, 131.7 (d, *J* = 8.2 Hz), 131.3 (d, *J* = 3.6 Hz), 129.8, 128.2, 128.0, 122.2 (d, *J* = 6.3 Hz), 119.8 (d, *J* = 3.2 Hz), 116.9 (d, *J* = 19.4 Hz), 80.6, 73.5, 53.4 (d, *J* = 2.0 Hz), 51.9, 45.1, 39.3, 28.7 (d, *J* = 5.9 Hz). **^{19}F NMR (376 MHz, $CDCl_3$)** δ -136.67. **IR** (KBr, cm^{-1}) *γ* 3300, 3030, 2924, 2853, 1720, 1631, 1598, 1484, 1454, 1437, 1374, 1240, 1200, 1116, 1057, 925, 778, 733, 702. **HRMS** (ESI) m/z $(M+Na)^+$: calculated for $(C_{21}H_{18}FNO_3Na)^+$: 374.1163, found: 374.1171; **$[\alpha]^{20}_D$** = +51.2 (*c* = 0.44, $CHCl_3$); The product was analyzed by HPLC to determine the enantiomeric excess: >99% e.e. (CHIRALPAK IG, hexane/*i*-PrOH = 70/30, flow rate: 1.0 mL/min, T = 30 ℃, 254 nm), t_R (major) = 8.38 min, t_R (minor) = 10.36 min.

Methyl 2-((*R*)-1,7-dimethyl-2-oxo-3-((*R*)-1-phenylprop-2-yn-1-yl)indolin-3-yl)acetate ((*R*,*R*)-3ma)

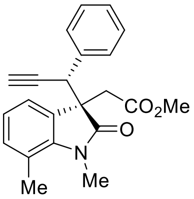

White solid, m.p. 109 °C; 80% yield; >95:5 d.r.; **^{1}H NMR (500 MHz, $CDCl_3$)** δ 7.25 – 7.18 (m, 3H), 7.11 – 7.05 (m, 2H), 6.97 (d, *J* = 7.2 Hz, 1H), 6.85 (t, *J* = 7.6 Hz, 1H), 6.67 (dd, *J* = 7.4, 1.3 Hz, 1H), 4.10 (d, *J* = 2.6 Hz, 1H), 3.44 (s, 3H), 3.36 (s, 3H), 3.33 (d, *J* = 16.7 Hz, 1H), 2.87 (d, *J* = 16.7 Hz, 1H), 2.45 (s, 3H), 2.25 (d, *J* = 2.6 Hz, 1H). **^{13}C NMR (126 MHz, $CDCl_3$)** δ 178.0, 170.2, 142.9, 134.8, 132.6, 129.8, 128.8, 128.0, 127.7, 121.8, 121.7, 119.4, 81.1, 73.3, 52.4, 51.8, 45.4, 39.3, 29.6, 19.2. **IR** (KBr, cm^{-1}) *γ* 3360, 3303, 2922, 2852, 1742, 1712, 1632, 1601, 1455, 1369, 1262, 1198, 1114, 1077, 747, 701, 666. **HRMS** (ESI) m/z $(M+Na)^+$: calculated for $(C_{21}H_{21}NO_3Na)^+$: 370.1414, found: 370.1421; **$[\alpha]^{20}_D$** = +9.7 (*c* = 0.09, $CHCl_3$); The product was analyzed by HPLC to determine the enantiomeric excess: >99% e.e. (CHIRALPAK IG, hexane/*i*-PrOH = 70/30, flow rate: 1.0 mL/min, T = 30 ℃, 230 nm), t_R (major) = 10.71 min, t_R (minor) = 14.86 min.

Methyl 2-((*R*)-3-((*R*)-1-(4-fluorophenyl)prop-2-yn-1-yl)-1-methyl-2-oxoindolin-3-yl)acetate ((*R*,*R*)-3ab)

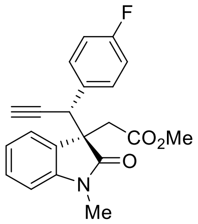

White solid, m.p. 55 °C; 76% yield; >95:5 d.r.; **^{1}H NMR (500 MHz, CDCl$_3$)** δ 7.29 – 7.22 (m, 1H), 7.10 – 7.03 (m, 2H), 6.99 (td, *J* = 7.5, 1.0 Hz, 1H), 6.92 (dd, *J* = 7.4, 1.3 Hz, 1H), 6.90 – 6.85 (m, 2H), 6.69 (d, *J* = 7.7 Hz, 1H), 4.16 (d, *J* = 2.6 Hz, 1H), 3.44 (s, 3H), 3.29 (d, *J* = 16.7 Hz, 1H), 3.08 (s, 3H), 2.93 (d, *J* = 16.6 Hz, 1H), 2.27 (d, *J* = 2.6 Hz, 1H). **^{13}C NMR (126 MHz, CDCl$_3$)** δ 176.9, 170.1, 162.5 (d, *J* = 247.0 Hz), 145.0, 131.3 (d, *J* = 8.1 Hz), 130.7 (d, *J* = 3.2 Hz), 129.0, 128.3, 123.8, 121.9, 114.7 (d, *J* = 21.6 Hz), 108.0, 80.8, 73.7, 53.0, 51.9, 44.2, 39.1, 26.2. **^{19}F NMR (471 MHz, CDCl$_3$)** δ -114.28. **IR** (KBr, cm^{-1}) *γ* 3299, 3010, 2953, 2925, 2853, 1715, 1612, 1508, 1494, 1470, 1377, 1353, 1224, 1176, 1160, 1091, 827, 753, 568, 543. **HRMS** (ESI) m/z (M+Na)$^{+}$: calculated for $(C_{21}H_{18}FNO_3Na)^{+}$: 374.1163, found: 374.1172; **$[\alpha]^{20}_{D}$** = +44.7 (*c* = 0.54, CHCl$_3$); The product was analyzed by HPLC to determine the enantiomeric excess: >99% e.e. (CHIRALPAK IG, hexane/*i*-PrOH = 70/30, flow rate: 1.0 mL/min, T = 30 °C, 254 nm), t_R (major) = 10.21 min, t_R (minor) = 12.80 min.

Methyl 2-((*R*)-3-((*R*)-1-(4-chlorophenyl)prop-2-yn-1-yl)-1-methyl-2-oxoindolin-3-yl)acetate ((*R*,*R*)-3ac)

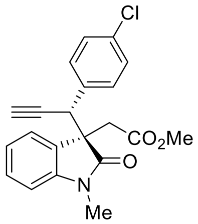

Yellow oil; 74% yield; >95:5 d.r.; **^{1}H NMR (400 MHz, CDCl$_3$)** δ 7.30 – 7.23 (m, 1H), 7.20 – 7.14 (m, 2H), 7.06 – 7.02 (m, 2H), 6.99 (td, *J* = 7.5, 1.0 Hz, 1H), 6.93 – 6.88 (m, 1H), 6.71 (dt, *J* = 7.8, 0.8 Hz, 1H), 4.15 (d, *J* = 2.6 Hz, 1H), 3.44 (s, 3H), 3.28 (d, *J* = 16.6 Hz, 1H), 3.09 (s, 3H), 2.91 (d, *J* = 16.5 Hz, 1H), 2.26 (d, *J* = 2.6 Hz, 1H). **^{13}C NMR (101 MHz, CDCl$_3$)** δ 176.9, 170.0, 144.9, 133.9, 133.5, 131.0, 129.1, 128.0, 128.0, 123.8, 122.0, 108.1, 80.5, 73.8, 52.9, 51.9, 44.2, 39.1, 26.3. **IR** (KBr, cm^{-1}) *γ* 3298, 3055, 3011, 2952, 2925, 2853, 1715, 1613, 1493, 1470, 1377, 1352, 1198, 1175, 1091, 1016, 819, 753, 651, 592, 543, 491. **HRMS** (ESI) m/z (M+Na)$^{+}$: calculated for $(C_{21}H_{18}ClNO_3Na)^{+}$: 390.0867, found: 390.0870; **$[\alpha]^{20}_{D}$** = +36.4 (*c* = 0.50, CHCl$_3$); The product was analyzed by HPLC to determine the enantiomeric excess: >99% e.e.

(CHIRALPAK IG, hexane/*i*-PrOH = 70/30, flow rate: 1.0 mL/min, T = 30 ℃, 230 nm), t_R (major) = 10.60 min, t_R (minor) = 13.93 min.

Methyl 2-((*R*)-3-((*R*)-1-(4-bromophenyl)prop-2-yn-1-yl)-1-methyl-2-oxoindolin-3-yl)acetate ((*R*,*R*)-3ad)

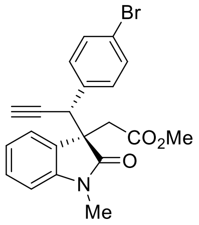

Yellow oil; 74% yield; >95:5 d.r.; **^{1}H NMR (400 MHz, CDCl$_3$)** δ 7.36 – 7.30 (m, 2H), 7.29 – 7.23 (m, 1H), 7.02 – 6.95 (m, 3H), 6.93 – 6.87 (m, 1H), 6.71 (dt, *J* = 7.8, 0.7 Hz, 1H), 4.14 (d, *J* = 2.6 Hz, 1H), 3.44 (s, 3H), 3.27 (d, *J* = 16.5 Hz, 1H), 3.09 (s, 3H), 2.91 (d, *J* = 16.6 Hz, 1H), 2.25 (d, *J* = 2.6 Hz, 1H). **^{13}C NMR (101 MHz, CDCl$_3$)** δ 176.9, 170.0, 144.9, 134.0, 131.4, 131.0, 129.1, 128.0, 123.9, 122.2, 122.0, 108.1, 80.4, 73.8, 52.8, 51.9, 44.3, 39.0, 26.3. **IR** (KBr, cm^{-1}) *γ* 3295, 3011, 2952, 2925, 2853, 1715, 1613, 1488, 1470, 1377, 1352, 1199, 1175, 1091, 1012, 816, 753, 643, 543. **HRMS** (ESI) m/z (M+Na)$^+$: calculated for $(C_{21}H_{18}BrNO_3Na)^+$: 434.0362, found: 434.0364; **[α]^{20}$_D$** = +28.3 (*c* = 0.54, CHCl$_3$); The product was analyzed by HPLC to determine the enantiomeric excess: >99% e.e. (CHIRALPAK IG, hexane/*i*-PrOH = 70/30, flow rate: 1.0 mL/min, T = 30 ℃, 230 nm), t_R (major) = 11.44 min, t_R (minor) = 13.93 min.

Methyl 2-((*R*)-1-methyl-2-oxo-3-((*R*)-1-(*p*-tolyl)prop-2-yn-1-yl)indolin-3-yl) acetate ((*R*,*R*)-3ae)

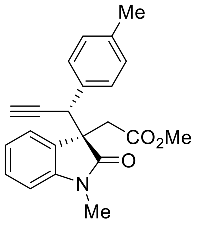

White solid, m.p. 52 °C; 75% yield; >95:5 d.r.; **^{1}H NMR (500 MHz, CDCl$_3$)** δ 7.29 – 7.24 (m, 1H), 7.04 (m, 4H), 6.96 (td, *J* = 7.5, 1.1 Hz, 1H), 6.82 (dd, *J* = 7.5, 1.2 Hz, 1H), 6.74 (d, *J* = 7.8 Hz, 1H), 4.12 (d, *J* = 2.6 Hz, 1H), 3.40 (s, 3H), 3.24 (d, *J* = 16.5 Hz, 1H), 3.15 (s, 3H), 2.86 (d, *J* = 16.6 Hz, 1H), 2.31 (s, 3H), 2.15 (d, *J* = 2.5 Hz, 1H). **^{13}C NMR (126 MHz, CDCl$_3$)** δ 177.6, 170.1, 145.2, 137.8, 131.9, 129.8, 128.8, 128.6, 128.3, 124.2, 121.7, 107.8, 81.2, 72.9, 53.1, 51.8, 44.6, 39.2, 26.3, 21.2. **IR** (KBr, cm^{-1}) *γ* 3290, 3009, 2952, 2924, 2854, 1715, 1613, 1496, 1470, 1377, 1352, 1261, 1197, 1175, 1091, 1022, 811, 753, 667, 642, 543. **HRMS** (ESI) m/z (M+Na)$^+$: calculated for $(C_{22}H_{21}NO_3Na)^+$: 370.1414, found: 370.1418; **[α]^{20}$_D$** = +16.1 (*c* = 0.50, CHCl$_3$); The product was analyzed by HPLC to determine the enantiomeric excess: 99% e.e. (CHIRALPAK IA,

hexane/*i*-PrOH = 70/30, flow rate: 1.0 mL/min, T = 30 ℃, 254 nm), t_R (minor) = 5.96 min, t_R (major) = 7.44 min.

Methyl 2-((*R*)-3-((*R*)-1-(4-methoxyphenyl)prop-2-yn-1-yl)-1-methyl-2-oxoindolin-3-yl)acetate ((*R,R*)-3af)

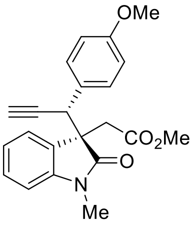

White solid, m.p. 85 ℃; 76% yield; >95:5 d.r.; **^{1}H NMR (500 MHz, $CDCl_3$)** δ 7.29 – 7.23 (m, 1H), 7.05 (d, *J* = 8.8 Hz, 2H), 6.97 (td, *J* = 7.5, 1.1 Hz, 1H), 6.85 (dd, *J* = 7.4, 1.3 Hz, 1H), 6.76 (d, *J* = 8.7 Hz, 2H), 6.73 (d, *J* = 7.8 Hz, 1H), 4.11 (d, *J* = 2.6 Hz, 1H), 3.78 (s, 3H), 3.41 (s, 3H), 3.25 (d, *J* = 16.5 Hz, 1H), 3.13 (s, 3H), 2.87 (d, *J* = 16.5 Hz, 1H), 2.18 (d, *J* = 2.6 Hz, 1H). **^{13}C NMR (126 MHz, $CDCl_3$)** δ 177.5, 170.1, 159.4, 145.2, 130.9, 128.8, 128.3, 126.9, 124.1, 121.7, 113.3, 107.9, 81.2, 73.0, 55.4, 53.2, 51.8, 44.2, 39.2, 26.3. **IR** (KBr, cm^{-1}) γ 3288, 3005, 2954, 2926, 2852, 1715, 1612, 1511, 1494, 1170, 1377, 1353, 1257, 1177, 1031, 825, 753, 667, 543. **HRMS** (ESI) m/z $(M+Na)^+$: calculated for $(C_{22}H_{21}NO_4Na)^+$: 386.1363, found: 386.1376; **$[\alpha]^{20}_D$** = +15.5 (*c* = 0.50, $CHCl_3$); The product was analyzed by HPLC to determine the enantiomeric excess: >99% e.e. (CHIRALPAK IG, hexane/*i*-PrOH = 70/30, flow rate: 1.0 mL/min, T = 30 ℃, 254 nm), t_R (major) = 16.39 min, t_R (minor) = 18.68 min.

Methyl 2-((*R*)-3-((*R*)-1-(3-fluorophenyl)prop-2-yn-1-yl)-1-methyl-2-oxoindolin-3-yl)acetate ((*R,R*)-3ag)

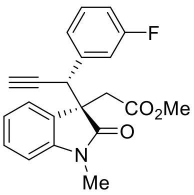

White solid, m.p. 50-60 ℃; 77% yield; >95:5 d.r.; **^{1}H NMR (500 MHz, $CDCl_3$)** δ 7.30 – 7.24 (m, 1H), 7.17 (td, *J* = 7.9, 6.1 Hz, 1H), 6.99 (t, *J* = 7.5 Hz, 1H), 6.97 – 6.93 (m, 1H), 6.91 (d, *J* = 7.7 Hz, 2H), 6.85 (dd, *J* = 10.0, 2.3 Hz, 1H), 6.71 (d, *J* = 7.8 Hz, 1H), 4.18 (d, *J* = 2.6 Hz, 1H), 3.44 (s, 3H), 3.27 (d, *J* = 16.5 Hz, 1H), 3.10 (s, 3H), 2.92 (d, *J* = 16.5 Hz, 1H), 2.25 (d, *J* = 2.4 Hz, 1H). **^{13}C NMR (126 MHz, $CDCl_3$)** δ 176.9, 170.0, 162.3 (d, *J* = 246.2 Hz), 145.0, 137.5 (d, *J* = 7.3 Hz), 129.2 (d, *J* = 8.2 Hz), 129.1, 128.1, 125.5 (d, *J* = 3.1 Hz), 123.9, 122.0, 116.8 (d, *J* = 22.5 Hz), 115.0 (d, *J* = 21.1 Hz), 108.0, 80.4, 73.8, 52.9, 51.9, 44.6 (d, *J* = 1.9 Hz), 39.1, 26.3. **^{19}F NMR (471 MHz, $CDCl_3$)** δ -113.14. **IR** (KBr, cm^{-1}) γ 3299, 3016, 2953, 2925, 2853, 1715, 1613,

1589, 1493, 1470, 1377, 1352, 1261, 1205, 1177, 1131, 1090, 1027, 764, 754, 693, 650, 542. **HRMS** (ESI) m/z $(M+Na)^+$: calculated for $(C_{21}H_{18}FNO_3Na)^+$: 374.1163, found: 374.1172; **$[\alpha]^{20}_D$** = +37.8 (*c* = 0.59, $CHCl_3$); The product was analyzed by HPLC to determine the enantiomeric excess: >99% e.e. (CHIRALPAK IG, hexane/*i*-PrOH = 70/30, flow rate: 1.0 mL/min, T = 30 ℃, 254 nm), t_R (major) = 9.87 min, t_R (minor) = 13.97 min.

Methyl 2-((*R*)-1-methyl-2-oxo-3-((*R*)-1-(*m*-tolyl)prop-2-yn-1-yl)indolin-3-yl) acetate ((*R*,*R*)-3ah)

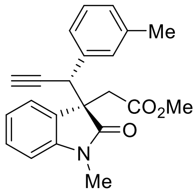

White solid, m.p. 98 °C; 66% yield; >95:5 d.r.; **^{1}H NMR (500 MHz, $CDCl_3$)** δ 7.29 – 7.23 (m, 1H), 7.12 (t, *J* = 7.5 Hz, 1H), 7.06 (d, *J* = 7.6 Hz, 1H), 6.99 – 6.91 (m, 3H), 6.83 (dd, *J* = 7.4, 1.3 Hz, 1H), 6.73 (d, *J* = 7.8 Hz, 1H), 4.12 (d, *J* = 2.5 Hz, 1H), 3.41 (s, 3H), 3.26 (d, *J* = 16.5 Hz, 1H), 3.13 (s, 3H), 2.87 (d, *J* = 16.5 Hz, 1H), 2.28 (s, 3H), 2.17 (d, *J* = 2.4 Hz, 1H). **^{13}C NMR (126 MHz, $CDCl_3$)** δ 177.5, 170.1, 145.2, 137.6, 134.8, 130.6, 128.8, 128.7, 128.3, 127.8, 127.0, 124.2, 121.6, 107.8, 81.1, 73.0, 53.1, 51.8, 44.9, 39.2, 26.3, 21.4. **IR** (KBr, cm^{-1}) *γ* 3289, 3021, 2952, 2924, 2853, 1715, 1613, 1494, 1470, 1377, 1353, 1261, 1203, 1176, 1091, 795, 755, 701, 543, 487. **HRMS** (ESI) m/z $(M+Na)^+$: calculated for $(C_{22}H_{21}NO_3Na)^+$: 370.1414, found: 370.1419; **$[\alpha]^{20}_D$** = +36.7 (*c* = 0.44, $CHCl_3$); The product was analyzed by HPLC to determine the enantiomeric excess: >99% e.e. (CHIRALPAK IG, hexane/*i*-PrOH = 85/15, flow rate: 1.0 mL/min, T = 30 ℃, 254 nm), t_R (major) = 17.39 min, t_R (minor) = 27.59 min.

Methyl 2-((*R*)-3-((*R*)-1-(3-methoxyphenyl)prop-2-yn-1-yl)-1-methyl-2-oxoindolin-3-yl)acetate ((*R*,*R*)-3ai)

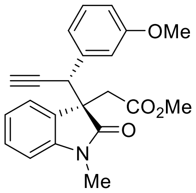

White solid, m.p. 58-64 °C; 75% yield; >95:5 d.r.; **^{1}H NMR (500 MHz, $CDCl_3$)** δ 7.29 – 7.23 (m, 1H), 7.15 (t, *J* = 7.9 Hz, 1H), 6.98 (t, *J* = 7.5 Hz, 1H), 6.89 (d, *J* = 7.4 Hz, 1H), 6.81 – 6.76 (m, 2H), 6.73 (d, *J* = 7.8 Hz, 1H), 6.63 (d, *J* = 2.1 Hz, 1H), 4.14 (d, *J* = 2.5 Hz, 1H), 3.69 (s, 3H), 3.42 (s, 3H), 3.25 (d, *J* = 16.5 Hz, 1H), 3.12 (s, 3H), 2.90 (d, *J* = 16.5 Hz, 1H), 2.19 (d, *J* = 2.5 Hz, 1H). **^{13}C NMR (126 MHz, $CDCl_3$)** δ 177.4, 170.1, 159.1,

145.2, 136.4, 128.9, 128.9, 128.3, 124.2, 122.3, 121.7, 115.2, 114.0, 107.9, 80.9, 73.2, 55.3, 53.1, 51.8, 45.0, 39.2, 26.3. **IR** (KBr, cm^{-1}) *γ* 3286, 2962, 2853, 1712, 1610, 1469, 1434, 1376, 1350, 1261, 1090, 1025, 865, 797, 754, 697, 542, 487. **HRMS** (ESI) m/z $(M+Na)^+$: calculated for $(C_{22}H_{21}NO_4Na)^+$: 386.1363, found: 386.1372; $[\alpha]^{20}_D$ = +34.7 (*c* = 0.58, $CHCl_3$); The product was analyzed by HPLC to determine the enantiomeric excess: >99% e.e. (CHIRALPAK IG, hexane/*i*-PrOH = 70/30, flow rate: 1.0 mL/min, T = 30 ℃, 254 nm), t_R (major) = 14.03 min, t_R (minor) = 20.86 min.

Methyl 2-((*R*)-3-((*S*)-1-(2-fluorophenyl)prop-2-yn-1-yl)-1-methyl-2-oxoindolin-3-yl)acetate ((*R*,*S*)-3aj)

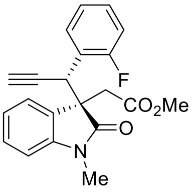

White solid, m.p. 105 °C; 60% yield; >95:5 d.r.; **^{1}H NMR (500 MHz, $CDCl_3$)** δ 7.35 (td, *J* = 7.6, 1.7 Hz, 1H), 7.27 (q, *J* = 5.9, 4.3 Hz, 2H), 7.10 (t, *J* = 7.6 Hz, 1H), 7.00 – 6.93 (m, 2H), 6.86 (d, *J* = 7.4 Hz, 1H), 6.76 (d, *J* = 7.8 Hz, 1H), 4.59 (d, *J* = 2.6 Hz, 1H), 3.37 (d, *J* = 17.5 Hz, 4H), 3.21 (s, 3H), 2.85 (d, *J* = 16.6 Hz, 1H), 2.12 (d, *J* = 2.5 Hz, 1H). **^{13}C NMR (126 MHz, $CDCl_3$)** δ 177.3, 170.1, 160.2 (d, *J* = 247.3 Hz), 145.1, 131.7 (d, *J* = 3.0 Hz), 130.0 (d, *J* = 8.3 Hz), 129.0, 127.8, 124.4, 123.9 (d, *J* = 3.7 Hz), 122.6 (d, *J* = 13.8 Hz), 121.7, 115.2 (d, *J* = 22.7 Hz), 107.8, 80.5, 72.5, 53.1, 51.8, 38.4 (d, *J* = 2.5 Hz), 36.9 (d, *J* = 2.8 Hz), 26.4. **^{19}F NMR (471 MHz, $CDCl_3$)** δ -115.00. **IR** (KBr, cm^{-1}) *γ* 3269, 3056, 3011, 2924, 2853, 1715, 1613, 1613, 1491, 1470, 1377, 1352, 1198, 1094, 1029, 802, 754, 677. **HRMS** (ESI) m/z $(M+Na)^+$: calculated for $(C_{21}H_{18}FNO_3Na)^+$: 374.1163, found: 374.1171; $[\alpha]^{20}_D$ = +8.2 (*c* = 0.33, $CHCl_3$); The product was analyzed by HPLC to determine the enantiomeric excess: >99% e.e. (CHIRALPAK IC, hexane/*i*-PrOH = 70/30, flow rate: 1.0 mL/min, T = 30 ℃, 254 nm), t_R (major) = 10.49 min, t_R (minor) = 13.34 min.

Methyl 2-((*R*)-1-methyl-2-oxo-3-((*R*)-1-(*o*-tolyl)prop-2-yn-1-yl)indolin-3-yl) acetate ((*R*,*R*)-3ak)

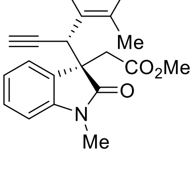

White solid, m.p. 130 °C; 52% yield; >95:5 d.r.; **^{1}H NMR (500 MHz, $CDCl_3$)** δ 7.39 – 7.34 (m, 1H), 7.30 – 7.24 (m, 1H), 7.18 – 7.14 (m, 2H), 7.11 – 7.07 (m, 1H), 7.02 (dd, *J* = 7.4, 1.5 Hz, 1H), 6.97 (dd, *J*

= 8.0, 6.8 Hz, 1H), 6.78 (d, *J* = 7.8 Hz, 1H), 4.46 (d, *J* = 2.6 Hz, 1H), 3.36 (s, 3H), 3.27 (d, *J* = 16.3 Hz, 1H), 3.21 (s, 3H), 2.85 (d, *J* = 16.2 Hz, 1H), 2.30 (s, 3H), 2.11 (d, *J* = 2.4 Hz, 1H). **^{13}C NMR (126 MHz, $CDCl_3$)** δ 177.8, 170.1, 145.1, 136.4, 133.9, 130.6, 130.5, 129.0, 128.3, 128.0, 125.7, 124.8, 121.5, 107.8, 82.1, 72.2, 53.7, 51.8, 40.1, 38.2, 26.4, 20.3. **IR** (KBr, cm^{-1}) *γ* 3289, 3054, 3020, 2925, 2853, 1715, 1612, 1493, 1470, 1436, 1377, 1351, 1262, 1197, 1175, 1090, 1028, 894, 753, 543, 488. **HRMS** (ESI) m/z $(M+Na)^+$: calculated for $(C_{22}H_{21}NO_3Na)^+$: 370.1414, found: 370.1419; **$[\alpha]^{20}_D$** = -7.2 (*c* = 0.34, $CHCl_3$); The product was analyzed by HPLC to determine the enantiomeric excess: 97% e.e. (CHIRALPAK IA, hexane/*i*-PrOH = 90/10, flow rate: 1.0 mL/min, T = 30 ℃, 254 nm), t_R (major) = 10.46 min, t_R (minor) = 11.57 min.

Methyl 2-((*R*)-3-((*R*)-1-(2-methoxyphenyl)prop-2-yn-1-yl)-1-methyl-2-oxoindolin-3-yl)acetate ((*R*,*R*)-3al)

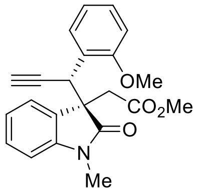

White solid, m.p. 141-146 °C; 48% yield; >95:5 d.r.; **^{1}H NMR (500 MHz, $CDCl_3$)** δ 7.30 – 7.23 (m, 3H), 6.93 (q, *J* = 7.6 Hz, 2H), 6.84 (d, *J* = 7.4 Hz, 1H), 6.79 (dd, *J* = 16.6, 8.0 Hz, 2H), 4.81 (d, *J* = 2.6 Hz, 1H), 3.79 (s, 3H), 3.40 (d, *J* = 16.7 Hz, 1H), 3.35 (s, 3H), 3.23 (s, 3H), 2.74 (d, *J* = 16.7 Hz, 1H), 2.02 (d, *J* = 2.6 Hz, 1H). **^{13}C NMR (126 MHz, $CDCl_3$)** δ 178.0, 170.5, 156.8, 145.3, 131.4, 129.4, 128.7, 128.3, 124.7, 123.8, 121.3, 120.2, 110.4, 107.6, 81.9, 71.5, 55.3, 53.5, 51.6, 38.3, 36.5, 26.4. **IR** (KBr, cm^{-1}) *γ* 3290, 2961, 2924, 2853, 1713, 1612, 1493, 1469, 1176, 1351, 1261, 1091, 1026, 799, 752, 665, 607, 542, 490. **HRMS** (ESI) m/z $(M+Na)^+$: calculated for $(C_{22}H_{21}NO_4Na)^+$: 386.1363, found: 386.1374; **$[\alpha]^{20}_D$** = -27.1 (*c* = 0.31, $CHCl_3$); The product was analyzed by HPLC to determine the enantiomeric excess: 98% e.e. (CHIRALPAK IA, hexane/*i*-PrOH = 70/30, flow rate: 1.0 mL/min, T = 30 ℃, 230 nm), t_R (major) = 4.94 min, t_R (minor) = 5.71 min.

Methyl 2-((*R*)-1-methyl-3-((*R*)-1-(naphthalen-2-yl)prop-2-yn-1-yl)-2-oxoindolin-3-yl)acetate ((*R*,*R*)-3am)

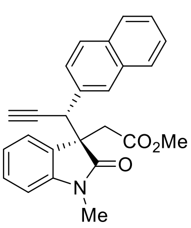

White solid, m.p. 150-153 °C; 87% yield; >95:5 d.r.; **^{1}H NMR (500 MHz, $CDCl_3$)** δ 7.82 – 7.78 (m, 1H), 7.77 – 7.73 (m, 1H), 7.71 (d, *J* = 8.5 Hz, 1H), 7.61 (d, *J* = 1.8 Hz, 1H), 7.49 – 7.43 (m, 2H), 7.32 – 7.21 (m, 2H), 6.97 (t, *J* = 7.5 Hz, 1H), 6.86 (d, *J* = 7.4 Hz, 1H), 6.69 (d, *J* = 7.8 Hz, 1H), 4.35 (d, *J* = 2.6 Hz, 1H), 3.41 (s, 3H), 3.34 (d, *J* = 16.5 Hz, 1H), 3.11 (s, 3H), 2.93 (d, *J* = 16.5 Hz, 1H), 2.25 (d, *J* = 2.5 Hz, 1H). **^{13}C NMR (126 MHz, $CDCl_3$)** δ 177.4, 170.1, 145.2, 133.0, 132.9, 132.5, 129.1, 128.9, 128.2, 128.1, 127.6, 127.6, 127.4, 126.4, 126.3, 124.2, 121.8, 107.9, 81.0, 73.4, 53.3, 51.8, 45.1, 39.3, 26.3. **IR** (KBr, cm^{-1}) *γ* 3292, 3056, 3012, 2952, 2925, 2853, 1715, 1613, 1494, 1470, 1436, 1376, 1352, 1201, 1176, 1090, 1018, 818, 751, 654, 543, 480. **HRMS** (ESI) m/z $(M+Na)^+$: calculated for $(C_{25}H_{21}NO_3Na)^+$: 406.1414, found: 406.1420; **$[\alpha]^{20}_D$** = +2.6 (*c* = 0.49, $CHCl_3$); The product was analyzed by HPLC to determine the enantiomeric excess: >99% e.e. (CHIRALPAK IG, hexane/*i*-PrOH = 70/30, flow rate: 1.0 mL/min, T = 30 ℃, 254 nm), t_R (major) = 15.75 min, t_R (minor) = 22.76 min.

Methyl 2-((*R*)-1-methyl-3-((*S*)-1-(naphthalen-2-yl)prop-2-yn-1-yl)-2-oxoindolin-3-yl)acetate ((*R,S*)-3am)

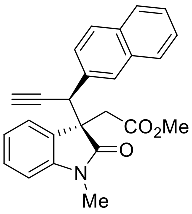

White solid, m.p. 145-151 °C; 67% yield; 95:5 d.r.; **^{1}H NMR (500 MHz, $CDCl_3$)** δ 7.71 (dd, *J* = 7.3, 1.3 Hz, 1H), 7.68 – 7.65 (m, 1H), 7.59 (dd, *J* = 7.1, 2.3 Hz, 1H), 7.44 (d, *J* = 8.6 Hz, 1H), 7.40 – 7.35 (m, 2H), 7.34 (d, *J* = 1.9 Hz, 1H), 7.17 (td, *J* = 7.7, 1.3 Hz, 1H), 7.09 (td, *J* = 7.5, 1.0 Hz, 1H), 6.94 (dd, *J* = 8.6, 1.9 Hz, 1H), 6.37 – 6.32 (m, 1H), 4.31 (d, *J* = 2.6 Hz, 1H), 3.63 (d, *J* = 16.7 Hz, 1H), 3.46 (s, 3H), 3.34 (d, *J* = 16.7 Hz, 1H), 2.66 (s, 3H), 2.57 (d, *J* = 2.6 Hz, 1H). **^{13}C NMR (126 MHz, $CDCl_3$)** δ 177.0, 170.3, 144.4, 132.7, 132.0, 129.0, 128.1, 128.0, 127.8, 127.4, 126.7, 126.5, 126.0, 125.9, 123.9, 122.1, 107.8, 82.3, 74.5, 53.6, 51.9, 45.1, 39.8, 25.9. **IR** (KBr, cm^{-1}) *γ* 3293, 3056, 3012, 2960, 2923, 2852, 1740, 1712, 1613, 1494, 1470, 1377, 1353, 1261, 1200, 1177, 1092, 1027, 859, 816, 752, 682, 543, 479. **HRMS** (ESI) m/z $(M+Na)^+$: calculated for $(C_{25}H_{21}NO_3Na)^+$: 406.1414, found: 406.1413; **$[\alpha]^{20}_D$** = +23.7 (*c* = 0.43, $CHCl_3$); The product was analyzed by HPLC to determine the enantiomeric excess: 99% e.e.

(CHIRALPAK IG, hexane/*i*-PrOH = 70/30, flow rate: 1.0 mL/min, T = 30 ℃, 254 nm), t_R (major) = 11.34 min, t_R (minor) = 13.08 min.

Methyl 2-((*S*)-1-methyl-3-((*S*)-1-(naphthalen-2-yl)prop-2-yn-1-yl)-2-oxoindolin-3-yl)acetate ((*S,S*)-3am)

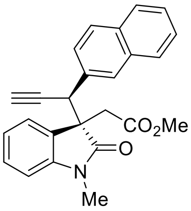

White solid, m.p. 150-153 °C; 87% yield; >95:5 d.r.; Spectral data were in agreement with those of the enantiomer reported above. **HRMS** (ESI) m/z (M+Na)$^+$: calculated for $(C_{25}H_{21}NO_3Na)^+$: 406.1414, found: 406.1418; **[α]^{20}$_D$** = -3.9 (c = 0.55, $CHCl_3$); The product was analyzed by HPLC to determine the enantiomeric excess: >99% e.e. (CHIRALPAK IG, hexane/*i*-PrOH = 70/30, flow rate: 1.0 mL/min, T = 30 ℃, 254 nm), t_R (minor) = 15.83 min, t_R (major) = 21.04 min.

Methyl 2-((*S*)-1-methyl-3-((*R*)-1-(naphthalen-2-yl)prop-2-yn-1-yl)-2-oxoindolin-3-yl)acetate ((*S,R*)-3am)

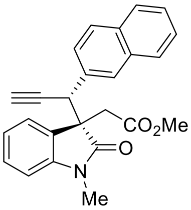

White solid, m.p. 145-151 °C; 63% yield; 95:5 d.r.; Spectral data were in agreement with those of the enantiomer reported above. **HRMS** (ESI) m/z (M+Na)$^+$: calculated for $(C_{25}H_{21}NO_3Na)^+$: 406.1414, found: 406.1422; **[α]^{20}$_D$** = -29.3 (*c* = 0.42, $CHCl_3$); The product was analyzed by HPLC to determine the enantiomeric excess: 99% e.e. (CHIRALPAK IG, hexane/*i*-PrOH = 70/30, flow rate: 1.0 mL/min, T = 30 ℃, 254 nm), t_R (minor) = 11.36 min, t_R (major) = 12.99 min.

***tert*-Butyl 3-((*R*)-1-((*R*)-3-(2-methoxy-2-oxoethyl)-1-methyl-2-oxoindolin-3-yl) prop-2-yn-1-yl)-1*H*-indole-1-carboxylate ((*R,R*)-3an)**

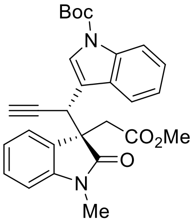

Yellow oil; 66% yield; >95:5 d.r.; **^{1}H NMR (500 MHz, $CDCl_3$)** δ 8.12 (d, *J* = 8.3 Hz, 1H), 7.55 (d, *J* = 7.9 Hz, 1H), 7.38 (s, 1H), 7.32 – 7.27 (m, 2H), 7.23 – 7.15 (m, 1H), 6.95 (q, *J* = 4.1, 3.5 Hz, 2H), 6.79 (d, *J* = 7.8 Hz, 1H), 4.44 (d, *J* = 2.7 Hz, 1H), 3.38 (s, 3H), 3.27 (d, *J* = 16.4 Hz, 1H), 3.18 (s, 3H), 2.93 (d, *J* = 16.4 Hz, 1H), 2.11 (d, *J* = 2.6 Hz, 1H), 1.67 (s, 9H). **^{13}C NMR (126 MHz, $CDCl_3$)** δ 177.7, 170.0, 149.5, 145.3, 135.2, 129.5, 129.0, 128.5, 126.5, 124.7, 124.2, 122.7, 121.8, 120.2, 115.3, 115.0, 107.9, 84.1, 80.5,

72.1, 53.3, 51.7, 39.1, 36.5, 28.3, 26.4. **IR** (KBr, cm^{-1}) *γ* 3304, 3054, 3008, 2929, 2854, 1732, 1613, 1564, 1494, 1470, 1452, 1359, 1308, 1255, 1156, 1084, 1020, 854, 750, 666, 543, 487. **HRMS** (ESI) m/z $(M+Na)^+$: calculated for $(C_{28}H_{28}N_2O_5Na)^+$: 495.1890, found: 495.1894; **$[\alpha]^{20}_D$** = +50.8 (*c* = 0.55, $CHCl_3$); The product was analyzed by HPLC to determine the enantiomeric excess: 99% e.e. (CHIRALPAK IG, hexane/*i*-PrOH = 70/30, flow rate: 1.0 mL/min, T = 30 ℃, 254 nm), t_R (major) = 9.31 min, t_R (minor) = 22.57 min.

Methyl 2-((*R*)-1-benzyl-2-oxo-3-((*S*)-1-phenylprop-2-yn-1-yl)indolin-3-yl)acetate ((*R*,*S*)-3ca)

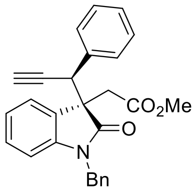

Yellow solid, m.p. 86 °C; 62% yield; >95:5 d.r.; **^{1}H NMR (400 MHz, $CDCl_3$)** δ 7.74 (dd, *J* = 7.1, 1.5 Hz, 1H), 7.21 – 7.11 (m, 4H), 7.11 – 7.01 (m, 4H), 6.93 – 6.89 (m, 2H), 6.67 (dd, *J* = 7.4, 2.1 Hz, 2H), 6.30 (dd, *J* = 7.4, 1.3 Hz, 1H), 4.65 (d, *J* = 16.1 Hz, 1H), 4.49 (d, *J* = 16.1 Hz, 1H), 4.21 (d, *J* = 2.6 Hz, 1H), 3.65 (d, *J* = 16.4 Hz, 1H), 3.42 (s, 3H), 3.33 (d, *J* = 16.4 Hz, 1H), 2.53 (d, *J* = 2.6 Hz, 1H). **^{13}C NMR (101 MHz, $CDCl_3$)** δ 177.2, 170.1, 144.0, 135.5, 134.9, 129.2, 128.9, 128.6, 127.9, 127.8, 127.7, 127.2, 126.8, 124.1, 122.2, 109.2, 82.6, 74.2, 53.4, 51.8, 45.0, 44.0, 40.7. **IR** (KBr, cm^{-1}) *γ* 3290, 3060, 3030, 2923, 2853, 1741, 1716, 1613, 1488, 1467, 1455, 1355, 1262, 1198, 1175, 1101, 1079, 1013, 800, 753, 697, 651, 552. **HRMS** (ESI) m/z $(M+Na)^+$: calculated for $(C_{27}H_{23}NO_3Na)^+$: 432.1570, found: 432.1574; **$[\alpha]^{20}_D$** = -11.1 (*c* = 0.53, $CHCl_3$); The product was analyzed by HPLC to determine the enantiomeric excess: >99% e.e. (CHIRALPAK IE, hexane/*i*-PrOH = 70/30, flow rate: 1.0 mL/min, T = 30 ℃, 254 nm), t_R (major) = 9.57 min, t_R (minor) = 12.03 min.

Methyl 2-((*R*)-6-fluoro-1-methyl-2-oxo-3-((*S*)-1-phenylprop-2-yn-1-yl)indolin-3-yl)acetate ((*R*,*S*)-3ka)

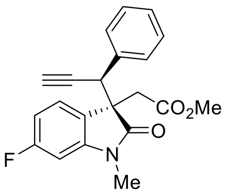

White solid, m.p. 55 °C; 56% yield; 91:9 d.r.; **^{1}H NMR (500 MHz, $CDCl_3$)** δ 7.57 (dd, *J* = 8.2, 5.4 Hz, 1H), 7.12 – 7.06 (m, 1H), 7.05 – 6.98 (m, 2H), 6.87 – 6.82 (m, 2H), 6.74 (ddd, *J* = 9.5, 8.2, 2.4 Hz, 1H), 6.21 (dd, *J* = 8.9, 2.3 Hz, 1H), 4.10 (d, *J* = 2.7 Hz, 1H),

3.56 (d, J = 16.8 Hz, 1H), 3.47 (s, 3H), 3.27 (d, J = 16.8 Hz, 1H), 2.75 (s, 3H), 2.52 (d, J = 2.6 Hz, 1H). **^{13}C NMR (126 MHz, $CDCl_3$)** δ 177.3, 170.2, 163.7 (d, J = 245.3 Hz), 146.0, (d, J = 11.8 Hz).134.2, 128.7, 127.8, 127.5, 124.9, 123.2 (d, J = 3.1 Hz), 108.2, 96.6 (d, J = 27.6 Hz), 82.1, 74.4, 53.3, 51.9, 45.0, 39.7, 26.0. **^{19}F NMR (471 MHz, $CDCl_3$)** δ -111.24. **IR** (KBr, cm^{-1}) γ 3297, 3063, 3031, 2956, 2923, 2853, 1721, 1615, 1504, 1437, 1380, 1261, 1246, 1198, 1177, 1086, 1024, 972, 936, 834, 797, 758, 699, 544. **HRMS** (ESI) m/z $(M+Na)^+$: calculated for $(C_{21}H_{18}FNO_3Na)^+$: 374.1163, found: 374.1172; **$[\alpha]^{20}_D$** = -46.8 (c = 0.44, $CHCl_3$); The product was analyzed by HPLC to determine the enantiomeric excess: 99% e.e. (CHIRALPAK IG, hexane/i-PrOH = 90/10, flow rate: 1.0 mL/min, T = 30 ℃, 254 nm), t_R (major) = 12.91 min, t_R (minor) = 14.82 min.

Methyl 2-((*R*)-3-((*S*)-1-(4-fluorophenyl)prop-2-yn-1-yl)-1-methyl-2-oxoindolin-3-yl)acetate ((*R,S*)-3ab)

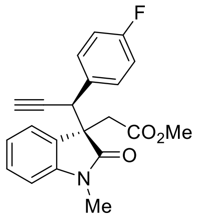

White solid, m.p. 86 °C; 64% yield; 95:5 d.r.; **^{1}H NMR (400 MHz, $CDCl_3$)** δ 7.63 (dd, J = 7.4, 1.3 Hz, 1H), 7.22 (td, J = 7.7, 1.3 Hz, 1H), 7.06 (td, J = 7.5, 1.0 Hz, 1H), 6.81 (dd, J = 8.7, 5.4 Hz, 2H), 6.67 (t, J = 8.7 Hz, 2H), 6.49 (d, J = 7.8 Hz, 1H), 4.12 (d, J = 2.7 Hz, 1H), 3.56 (d, J = 16.6 Hz, 1H), 3.44 (s, 3H), 3.26 (d, J = 16.7 Hz, 1H), 2.81 (s, 3H), 2.53 (d, J = 2.7 Hz, 1H). **^{13}C NMR (101 MHz, $CDCl_3$)** δ 176.8, 170.0, 162.2 (d, J = 246.4 Hz), 144.3, 130.3 (d, J = 8.1 Hz), 130.2 (d, J = 3.2 Hz), 129.0, 127.4, 123.7, 122.0, 114.1 (d, J = 21.4 Hz), 107.7, 81.9, 74.4, 53.4, 51.7, 44.1, 39.6, 25.9. **^{19}F NMR (376 MHz, $CDCl_3$)** δ -114.68. **IR** (KBr, cm^{-1}) γ 3299, 3012, 2922, 2852, 1741, 1710, 1612, 1508, 1469, 1376, 1354, 1224, 1177, 1160, 1093, 1028, 839, 753, 681, 567, 542. **HRMS** (ESI) m/z $(M+Na)^+$: calculated for $(C_{21}H_{18}FNO_3Na)^+$: 374.1163, found: 374.1168; **$[\alpha]^{20}_D$** = -52.7 (c = 0.38, $CHCl_3$); The product was analyzed by HPLC to determine the enantiomeric excess: >99% e.e. (CHIRALPAK IG, hexane/i-PrOH = 70/30, flow rate: 1.0 mL/min, T = 30 ℃, 254 nm), t_R (major) = 6.49 min, t_R (minor) = 7.17 min.

Methyl 2-((*R*)-3-((*S*)-1-(4-chlorophenyl)prop-2-yn-1-yl)-1-methyl-2-oxoindolin-3-

yl)acetate ((***R,S***)-3ac)

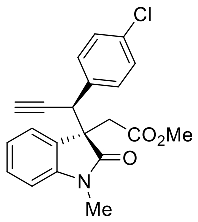

Yellow oil; 51% yield; >95:5 d.r.; **^{1}H NMR (500 MHz, $CDCl_3$)** δ 7.62 (d, *J* = 7.4 Hz, 1H), 7.23 (td, *J* = 7.7, 1.3 Hz, 1H), 7.06 (t, *J* = 7.5 Hz, 1H), 6.99 – 6.93 (m, 2H), 6.82 – 6.75 (m, 2H), 6.51 (d, *J* = 7.8 Hz, 1H), 4.11 (d, *J* = 2.6 Hz, 1H), 3.55 (d, *J* = 16.7 Hz, 1H), 3.44 (s, 3H), 3.26 (d, *J* = 16.7 Hz, 1H), 2.83 (s, 3H), 2.52 (d, *J* = 2.5 Hz, 1H). **^{13}C NMR (126 MHz, $CDCl_3$)** δ 176.84, 170.11, 144.37, 133.62, 133.15, 130.14, 129.15, 127.53, 127.44, 123.87, 122.18, 107.96, 81.82, 74.60, 53.47, 51.87, 44.35, 39.76, 26.02. **IR** (KBr, cm^{-1}) *γ* 3294, 3055, 2953, 2925, 2853, 1742, 1713, 1613, 1493, 1470, 1376, 1352, 1260, 1199, 1177, 1092, 1016, 832, 753, 678, 542, 468. **HRMS** (ESI) m/z $(M+Na)^+$: calculated for $(C_{21}H_{18}ClNO_3Na)^+$: 390.0867, found: 390.0875; **$[\alpha]^{20}_D$** = -20.9 (*c* = 0.34, $CHCl_3$); The product was analyzed by HPLC to determine the enantiomeric excess: 99% e.e. (CHIRALPAK IG, hexane/*i*-PrOH = 70/30, flow rate: 1.0 mL/min, T = 30 ℃, 230 nm), t_R (major) = 6.98 min, t_R (minor) = 7.85 min.

Methyl 2-((*R*)-3-((*S*)-1-(4-bromophenyl)prop-2-yn-1-yl)-1-methyl-2-oxoindolin-3-yl)acetate ((*R,S*)-3ad)

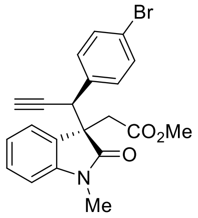

Yellow oil; 50% yield; >95:5 d.r.; **^{1}H NMR (500 MHz, $CDCl_3$)** δ 7.62 (d, *J* = 7.4 Hz, 1H), 7.23 (td, *J* = 7.8, 1.3 Hz, 1H), 7.11 (d, *J* = 8.5 Hz, 2H), 7.06 (t, *J* = 7.5 Hz, 1H), 6.74 – 6.69 (m, 2H), 6.52 (d, *J* = 7.8 Hz, 1H), 4.09 (d, *J* = 2.6 Hz, 1H), 3.55 (d, *J* = 16.6 Hz, 1H), 3.44 (s, 3H), 3.25 (d, *J* = 16.7 Hz, 1H), 2.83 (s, 3H), 2.52 (d, *J* = 2.5 Hz, 1H). **^{13}C NMR (126 MHz, $CDCl_3$)** δ 176.82, 170.09, 144.37, 133.68, 131.39, 130.49, 129.16, 127.41, 123.87, 122.19, 121.84, 107.99, 81.75, 74.61, 53.40, 51.87, 44.41, 39.77, 26.03. **IR** (KBr, cm^{-1}) *γ* 3293, 3055, 3009, 2953, 2924, 2853, 1742, 1712, 1613, 1489, 1470, 1376, 1352, 1260, 1200, 1177, 1192, 1073, 1012, 810, 753, 667, 542. **HRMS** (ESI) m/z $(M+Na)^+$: calculated for $(C_{21}H_{18}BrNO_3Na)^+$: 434.0362, found: 434.0367; **$[\alpha]^{20}_D$** = -9.5 (*c* = 0.40, $CHCl_3$); The product was analyzed by HPLC to determine the enantiomeric excess: >99% e.e. (CHIRALPAK IG, hexane/*i*-PrOH = 70/30, flow rate: 1.0 mL/min, T = 30 ℃, 230 nm), t_R (major) = 7.30 min, t_R (minor) = 8.49 min.

Methyl 2-((*R*)-1-methyl-2-oxo-3-((*S*)-1-(*p*-tolyl)prop-2-yn-1-yl)indolin-3-yl) acetate ((*R,S*)-3ae)

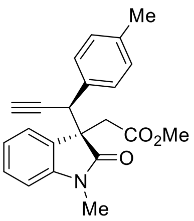

White solid, m.p. 50-55 °C; 50% yield; 92:8 d.r.; **^{1}H NMR (500 MHz, $CDCl_3$)** δ 7.63 (dd, *J* = 7.3, 1.2 Hz, 1H), 7.21 (td, *J* = 7.7, 1.3 Hz, 1H), 7.05 (td, *J* = 7.5, 1.0 Hz, 1H), 6.79 (d, *J* = 8.0 Hz, 2H), 6.74 – 6.69 (m, 2H), 6.48 (d, *J* = 7.7 Hz, 1H), 4.09 (d, *J* = 2.6 Hz, 1H), 3.56 (d, *J* = 16.7 Hz, 1H), 3.44 (s, 3H), 3.26 (d, *J* = 16.7 Hz, 1H), 2.80 (s, 3H), 2.49 (d, *J* = 2.6 Hz, 1H), 2.17 (s, 3H). **^{13}C NMR (126 MHz, $CDCl_3$)** δ 177.1, 170.3, 144.5, 137.3, 131.5, 128.8, 128.6, 128.1, 127.9, 123.9, 122.0, 107.7, 82.5, 74.0, 53.5, 51.8, 44.7, 39.8, 26.0, 21.1. **IR** (KBr, cm^{-1}) *γ* 3287, 3055, 3009, 2922, 2852, 1743, 1714, 1613, 1512, 1494, 1470, 1437, 1377, 1354, 1259, 1199, 1177, 1093, 1023, 811, 753, 682, 543. **HRMS** (ESI) m/z $(M+Na)^+$: calculated for $(C_{22}H_{21}NO_3Na)^+$: 370.1414, found: 370.1421; **$[\alpha]^{20}_D$** = -27.5 (*c* = 0.33, $CHCl_3$); The product was analyzed by HPLC to determine the enantiomeric excess: 99% e.e. (CHIRALPAK IG, hexane/*i*-PrOH = 70/30, flow rate: 1.0 mL/min, T = 30 ℃, 254 nm), t_R (major) = 7.30 min, t_R (minor) = 9.21 min.

Methyl 2-((*R*)-3-((*S*)-1-(3-fluorophenyl)prop-2-yn-1-yl)-1-methyl-2-oxoindolin-3-yl)acetate ((*R,S*)-3ag)

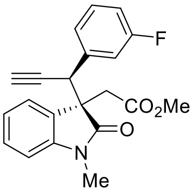

White solid, m.p. 68 °C; 50% yield; 94:6 d.r.; **^{1}H NMR (400 MHz, $CDCl_3$)** δ 7.62 (dd, *J* = 7.5, 1.3 Hz, 1H), 7.23 (td, *J* = 7.8, 1.3 Hz, 1H), 7.07 (td, *J* = 7.5, 1.0 Hz, 1H), 6.98 (td, *J* = 8.0, 6.0 Hz, 1H), 6.77 (tdd, *J* = 8.4, 2.6, 1.0 Hz, 1H), 6.70 (dt, *J* = 7.7, 1.3 Hz, 1H), 6.56 – 6.47 (m, 2H), 4.13 (d, *J* = 2.6 Hz, 1H), 3.56 (d, *J* = 16.6 Hz, 1H), 3.45 (s, 3H), 3.26 (d, *J* = 16.6 Hz, 1H), 2.82 (s, 3H), 2.54 (d, *J* = 2.6 Hz, 1H). **^{13}C NMR (101 MHz, $CDCl_3$)** δ 176.8, 170.1, 161.9 (d, *J* = 245.4 Hz), 144.4, 137.1 (d, *J* = 7.6 Hz), 129.2, 128.7 (d, *J* = 8.1 Hz), 127.5, 124.7 (d, *J* = 2.9 Hz), 123.8, 122.2, 115.7 (d, *J* = 22.7 Hz), 114.6 (d, *J* = 21.2 Hz), 107.8, 81.7, 74.7, 53.5, 51.9, 44.6 (d, *J* = 1.9 Hz), 39.7, 26.0. **^{19}F NMR (376 MHz, $CDCl_3$)** δ -113.89. **IR** (KBr, cm^{-1}) *γ* 3299, 3057, 3010, 2922, 2852, 1741, 1713, 1614, 1589, 1494, 1471, 1450, 1377, 1355, 1263, 1204, 1178, 1135, 1092, 872, 753, 691, 542, 522. **HRMS** (ESI) m/z $(M+Na)^+$: calculated for

($C_{21}H_{18}FNO_3Na$)$^+$: 374.1163, found: 374.1172; **[α]^{20}D** = -55.7 (*c* = 0.34, $CHCl_3$); The product was analyzed by HPLC to determine the enantiomeric excess: 99% e.e. (CHIRALPAK IG, hexane/*i*-PrOH = 70/30, flow rate: 1.0 mL/min, T = 30 ℃, 254 nm), t_R (major) = 7.09 min, t_R (minor) = 7.70 min.

Methyl 2-((*R*)-1-methyl-2-oxo-3-((*R*,*E*)-1-phenylpent-1-en-4-yn-3-yl)indolin-3-yl) acetate ((*R*,*R*)-3ao)

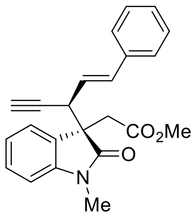

colorless liquid; 44% yield; >95:5 d.r.; **^{1}H NMR (400 MHz, $CDCl_3$)** δ 7.54 (dd, *J* = 7.4, 1.2 Hz, 1H), 7.30 (td, *J* = 7.8, 1.3 Hz, 1H), 7.25 – 7.12 (m, 3H), 7.18 – 7.02 (m, 3H), 6.79 (d, *J* = 7.8 Hz, 1H), 6.46 (d, *J* = 15.7 Hz, 1H), 5.49 (dd, *J* = 15.7, 8.1 Hz, 1H), 3.71 (ddd, *J* = 8.2, 2.6, 1.0 Hz, 1H), 3.44 (d, *J* = 16.5 Hz, 1H), 3.44 (s, 3H), 3.17 (d, *J* = 16.7 Hz, 1H), 3.15 (s, 3H), 2.47 (d, *J* = 2.5 Hz, 1H). **^{13}C NMR (101 MHz, $CDCl_3$)** δ 177.4, 170.2, 144.6, 136.5, 134.3, 129.0, 128.5, 128.2, 127.8, 126.5, 123.7, 122.8, 122.4, 108.0, 81.3, 74.5, 52.4, 51.8, 42.4, 39.7, 26.5. **IR** (KBr, cm^{-1}) *γ* 2929, 2387, 1959, 1713, 1613, 1494, 1470, 1435, 1377, 1350, 1176, 1091, 753, 697, 451, 434, 425. **HRMS** (ESI) m/z (M+Na)$^+$: calculated for ($C_{23}H_{21}NO_3Na$)$^+$: 382.1414, found: 382.1424; **[α]^{20}D** = -67.0 (*c* = 0.21, $CHCl_3$); The product was analyzed by HPLC to determine the enantiomeric excess: 99% e.e. (CHIRALPAK IG, hexane/*i*-PrOH = 70/30, flow rate: 1.0 mL/min, T = 30 ℃, 254 nm), t_R (major) = 10.68 min, t_R (minor) = 11.74 min.

Methyl 2-((*R*)-1-methyl-3-((*R*,*E*)-1-(naphthalen-2-yl)pent-1-en-4-yn-3-yl)-2-oxoindolin-3-yl)acetate ((*R*,*R*)-3ap)

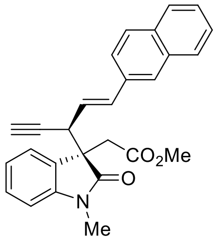

colorless liquid; 40% yield; >95:5 d.r.; **^{1}H NMR (500 MHz, $CDCl_3$)** δ 7.73 (td, *J* = 7.2, 2.5 Hz, 2H), 7.66 (d, *J* = 8.6 Hz, 1H), 7.58 (dd, *J* = 7.5, 1.2 Hz, 1H), 7.53 – 7.50 (m, 1H), 7.42 (tt, *J* = 6.9, 5.1 Hz, 2H), 7.30 (td, *J* = 7.7, 1.2 Hz, 1H), 7.23 (dd, *J* = 8.5, 1.8 Hz, 1H), 7.09 (td, *J* = 7.6, 1.0 Hz, 1H), 6.77 (d, *J* = 7.8 Hz, 1H), 6.61 (d, *J* = 15.6 Hz, 1H), 5.63 (dd, *J* = 15.7, 8.1 Hz, 1H), 3.77 (dt, *J* = 8.1, 1.6 Hz, 1H), 3.46 (d, *J* = 16.6 Hz, 1H), 3.46 (s, 3H), 3.20 (d, *J* = 16.6 Hz, 1H), 3.14 (s, 3H), 2.50 (d, *J* = 2.5 Hz, 1H). **^{13}C NMR (126 MHz, $CDCl_3$)** δ 177.4, 170.2, 144.6, 134.4, 133.9, 133.5, 133.1, 129.1,

128.2, 128.1, 127.7, 126.6, 126.4, 126.1, 123.8, 123.5, 123.1, 122.5, 108.0, 81.3, 74.5, 52.5, 51.9, 42.5, 39.7, 26.4. **IR** (KBr, cm^{-1}) *γ* 3461, 2924, 1959, 1713, 1613, 1494, 1469, 1378, 1261, 1203, 1179, 1092, 810, 754, 476. **HRMS** (ESI) m/z $(M+Na)^+$: calculated for $(C_{27}H_{23}NO_3Na)^+$: 432.1570, found: 432.1752; **$[α]^{20}_D$** = -11.0 (*c* = 0.26, $CHCl_3$); The product was analyzed by HPLC to determine the enantiomeric excess: >99% e.e. (CHIRALPAK IG, hexane/*i*-PrOH = 70/30, flow rate: 1.0 mL/min, T = 30 ℃, 254 nm), t_R (minor) = 16.43 min, t_R (minor) = 18.12 min.

Methyl 2-((*R*)-6-fluoro-1-methyl-2-oxo-3-((*R*,*E*)-1-phenylpent-1-en-4-yn-3-yl) indolin-3-yl)acetate ((*R*,*R*)-3ko)

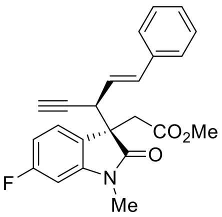

colorless liquid; 41% yield; >95:5 d.r.; **^{1}H NMR (400 MHz, $CDCl_3$)** δ 7.48 (dd, *J* = 8.2, 5.4 Hz, 1H), 7.25 – 7.16 (m, 3H), 7.13 – 7.06 (m, 2H), 6.74 (ddd, *J* = 9.6, 8.2, 2.4 Hz, 1H), 6.53 (dd, *J* = 8.9, 2.4 Hz, 1H), 6.46 (d, *J* = 15.7 Hz, 1H), 5.49 (dd, *J* = 15.7, 8.0 Hz, 1H), 3.68 (ddd, *J* = 8.1, 2.6, 1.0 Hz, 1H), 3.47 (s, 3H), 3.42 (d, *J* = 16.7 Hz, 1H), 3.15 (d, *J* = 16.7 Hz, 1H), 3.13 (s, 4H), 2.47 (d, *J* = 2.5 Hz, 1H). **^{13}C NMR (101 MHz, $CDCl_3$)** δ 177.7, 170.1, 163.7 (d, *J* = 245.5 Hz), 146.2 (d, *J* = 11.8 Hz), 136.4, 134.6, 128.6, 128.0, 126.5, 124.8 (d, *J* = 9.8 Hz), 123.5, 122.5, 108.5 (d, *J* = 22.3 Hz), 97.0 (d, *J* = 27.7 Hz), 81.1, 74.6, 52.1, 51.9, 42.4, 39.8, 26.6. **^{19}F NMR** (376 MHz, $CDCl_3$) δ -111.16. **IR** (KBr, cm^{-1}) *γ* 3467, 2962, 1959, 1720, 1618, 1503, 1467, 1382, 1261, 1180, 1089, 938, 802, 695, 545. **HRMS** (ESI) m/z $(M+Na)^+$: calculated for $(C_{23}H_{20}FNO_3Na)^+$: 400.1319, found: 400.1327; **$[α]^{20}_D$** = -55.0 (*c* = 0.20, $CHCl_3$); The product was analyzed by HPLC to determine the enantiomeric excess: >99% e.e. (CHIRALPAK IG, hexane/*i*-PrOH = 70/30, flow rate: 1.0 mL/min, T = 30 ℃, 254 nm), t_R (major) = 8.10 min, t_R (minor) = 9.19 min.

Methyl 2-((*R*)-6-fluoro-1-methyl-3-((*R*,*E*)-1-(naphthalen-2-yl)pent-1-en-4-yn-3-yl) -2-oxoindolin-3-yl)acetate ((*R*,*R*)-3kp)

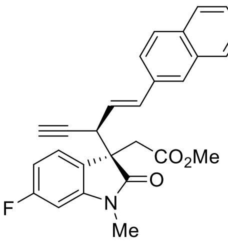

colorless liquid; 41% yield; >95:5 d.r.; **^{1}H NMR (400 MHz, $CDCl_3$)** δ 7.74 (dd, *J* = 8.4, 5.9 Hz, 2H), 7.68 (d, *J* = 8.6 Hz, 1H), 7.55 – 7.49 (m, 2H), 7.47 – 7.38 (m, 2H), 7.26 – 7.22 (m, 1H),

6.76 (ddd, *J* = 9.6, 8.2, 2.3 Hz, 1H), 6.61 (d, *J* = 15.7 Hz, 1H), 6.52 (dd, *J* = 8.9, 2.3 Hz, 1H), 5.62 (dd, *J* = 15.7, 8.1 Hz, 1H), 3.74 (ddd, *J* = 8.1, 2.6, 1.0 Hz, 1H), 3.48 (s, 3H), 3.45 (d, *J* = 16.6 Hz, 1H), 3.18 (d, *J* = 16.6 Hz, 1H), 3.12 (s, 3H), 2.50 (d, *J* = 2.5 Hz, 1H). **^{13}C NMR (101 MHz, $CDCl_3$)** δ 177.8, 170.1, 163.7 (d, *J* = 245.6 Hz), 146.2 (d, *J* = 11.7 Hz), 134.6, 133.7, 133.5, 133.2, 128.3, 128.1, 127.7, 126.7, 126.5, 126.2, 124.8 (d, *J* = 9.8 Hz), 123.5 (d, *J* = 3.0 Hz), 123.4, 122.7, 108.5 (d, *J* = 22.2 Hz), 97.0 (d, *J* = 27.5 Hz), 81.1, 74.7, 52.2, 51.9, 42.5, 39.7, 26.6. **^{19}F NMR** (376 MHz, $CDCl_3$) δ -111.08. **IR** (KBr, cm^{-1}) *γ* 3466, 2925, 1959, 1720, 1612, 1504, 1453, 1381, 1264, 1171, 1088, 938, 812, 757, 666, 576, 545, 478. **HRMS** (ESI) m/z $(M+Na)^+$: calculated for $(C_{27}H_{23}FNO_3Na)^+$: 450.1476, found: 450.1479; **$[\alpha]^{20}_D$** = -8.2 (*c* = 0.20, $CHCl_3$); The product was analyzed by HPLC to determine the enantiomeric excess: 99% e.e. (CHIRALPAK IG, hexane/*i*-PrOH = 70/30, flow rate: 1.0 mL/min, T = 30 ℃, 254 nm), t_R (minor) = 11.57 min, t_R (major) = 13.18 min.

Synthetic Applications

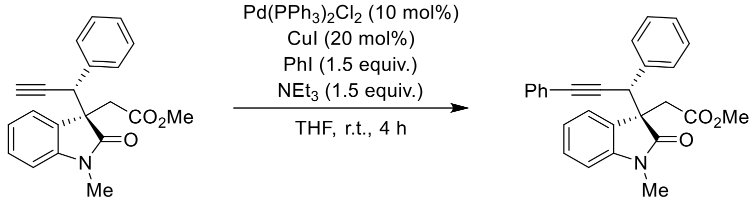

(*R*,*R*)-**3aa**
>99% e.e.

5
95% yield
>99% e.e.

To a flame-dried and N_2-purged Schlenk tube were added (*R*,*R*)-**3aa** (33.3 mg, 0.10 mmol, 1.0 equiv.), $Pd(PPh_3)_2Cl_2$ (7.0 mg, 0.01 mmol, 10 mol%), and CuI (3.8 mg, 0.02 mmol, 20 mol%). The vial was then sealed, purged and backfilled with N_2 three times before adding NEt_3 (21 µL, 0.15 mmol, 1.5 equiv.), PhI (17 µL, 0.15 mmol, 1.5 equiv.) and THF (1.0 mL) at room temperature. The resulting mixture was stirred at room temperature under nitrogen atmosphere for 4 hours. After concentrated under reduced pressure, the residue was purified by column chromatography on silica gel to give **5** (38.9 mg, 95% yield, >99% e.e.).

Methyl 2-((*R*)-3-((*S*)-1,3-diphenylprop-2-yn-1-yl)-1-methyl-2-oxoindolin-3-yl) acetate (5)

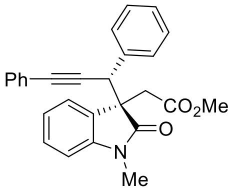

Colorless oil; 95% yield; **^{1}H NMR (500 MHz, $CDCl_3$)** δ 7.31 – 7.18 (m, 11H), 6.98 (t, *J* = 7.5 Hz, 1H), 6.84 – 6.81 (m, 1H), 6.77 (d, *J* = 7.8 Hz, 1H), 4.33 (s, 1H), 3.41 (s, 3H), 3.31 (d, *J* = 16.5 Hz, 1H), 3.15 (s, 3H), 2.88 (d, *J* = 16.5 Hz, 1H). **^{13}C NMR (126 MHz, $CDCl_3$)** δ 177.8, 170.1, 145.3, 135.5, 131.7, 130.1, 128.9, 128.4, 128.2, 128.1, 128.0, 128.0, 124.2, 123.1, 121.7, 107.8, 86.6, 85.2, 53.6, 51.8, 45.9, 39.1, 26.4. **IR** (KBr, cm^{-1}) *γ* 3058, 3029, 2954, 2926, 2854, 1741, 1716, 1613, 1492, 1470, 1377, 1353, 1261, 1198, 1175, 1081, 1028, 801, 756, 700, 605, 543. **HRMS** (ESI) m/z $(M+Na)^+$: calculated for $(C_{27}H_{23}NO_3Na)^+$: 432.1570, found: 432.1569; **$[\alpha]^{20}_D$** = +131.3 (*c* = 0.72, $CHCl_3$); The product was analyzed by HPLC to determine the enantiomeric excess: >99% e.e. (CHIRALPAK IA, hexane/*i*-PrOH = 70/30, flow rate: 1.0 mL/min, T = 30 ℃, 254 nm), t_R (minor) = 6.77 min, t_R (major) = 7.78 min.

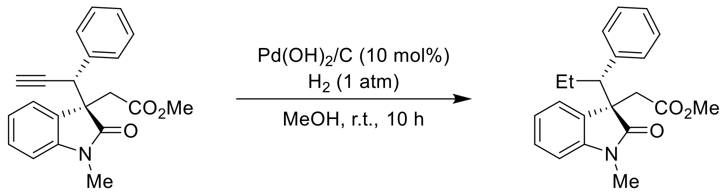

To a stirring solution of (*R*,*R*)-**3aa** (33.3 mg, 0.10 mmol, 1.0 equiv.) in MeOH (5.0 mL) was slowly added palladium hydroxide-onactivated charcoal (10%; 20.0 mg) at room temperature. The resulting mixture was stirred at room temperature in an atmosphere of hydrogen gas for 6 hours. The mixture was filtered and concentrated under reduced pressure. The residue was purified by column chromatography on silica gel to give **6** (33.0 mg, 98% yield, >99%e.e.).

Methyl 2-((*R*)-1-methyl-2-oxo-3-((*R*)-1-phenylpropyl)indolin-3-yl)acetate (6)

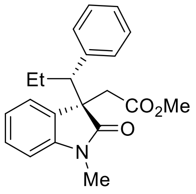

Colorless oil; 98% yield; **^{1}H NMR (500 MHz, CDCl$_3$)** δ 7.31 – 7.21 (m, 4H), 7.09 (d, *J* = 6.5 Hz, 2H), 6.96 (t, *J* = 7.5 Hz, 1H), 6.88 (dd, *J* = 7.3, 1.2 Hz, 1H), 6.77 (d, *J* = 7.8 Hz, 1H), 3.33 (s, 3H), 3.23 (s, 3H), 3.11 (d, *J* = 16.3 Hz, 1H), 3.01 (dd, *J* = 12.1, 3.6 Hz, 1H), 2.62 (d, *J* = 16.3 Hz, 1H), 1.55 (dqd, *J* = 14.6, 7.3, 3.5 Hz, 1H), 1.45 (ddq, *J* = 14.2, 12.1, 7.2 Hz, 1H), 0.59 (t, *J* = 7.3 Hz, 3H). **^{13}C NMR (126 MHz, CDCl$_3$)** δ 179.7, 170.4, 144.9, 138.2, 130.2, 129.5, 128.3, 128.0, 127.3, 124.5, 121.7, 107.8, 54.5, 53.5, 51.6, 40.7, 26.4, 21.2, 12.1. **IR** (KBr, cm^{-1}) *γ* 3057, 3027, 2962, 2932, 2875, 1743, 1712, 1612, 1493, 1469, 1377, 1350, 1252, 1197, 1176, 1126, 1094, 1027, 755, 702, 679, 603, 543, 488. **$[\alpha]^{20}_D$** = -11.2 (*c* = 0.73, CHCl$_3$); **HRMS** (ESI) m/z (M+Na)$^+$: calculated for ($C_{21}H_{23}NO_3Na$)$^+$: 360.1570, found: 360.1569; The product was analyzed by HPLC to determine the enantiomeric excess: >99% e.e. (CHIRALPAK IG, hexane/*i*-PrOH = 70/30, flow rate: 1.0 mL/min, T = 30 ℃, 254 nm), t_R (minor) = 7.32 min, t_R (major) = 9.08 min.

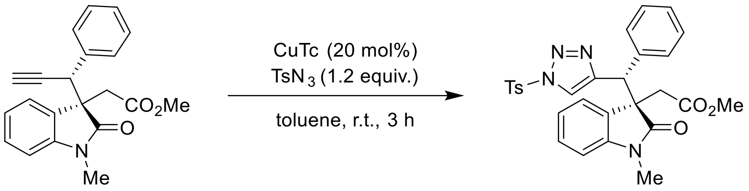

(*R,R*)-**3aa** >99% e.e. **7** 89% yield >99% e.e.

To a flame-dried and N_2-purged Schlenk tube were added (*R*,*R*)-**3aa** (33.3 mg, 0.10 mmol, 1.0 equiv.) and CuTc (3.8 mg, 0.02 mmol, 20 mol%). The vial was then sealed, purged and backfilled with N_2 three times before adding TsN_3 (23.7 mg, 0.12 mmol, 1.2 equiv.) and toluene (1.0 mL) at room temperature. The resulting mixture was stirred at room temperature under nitrogen atmosphere for 3 hours. After concentrated under reduced pressure, the residue was purified by column chromatography on silica gel to give **7** (47.2 mg, 89% yield, >99%e.e.).

Methyl 2-((*R*)-1-methyl-2-oxo-3-((*R*)-phenyl(1-tosyl-1*H*-1,2,3-triazol-4-yl)methyl) indolin-3-yl)acetate (7)

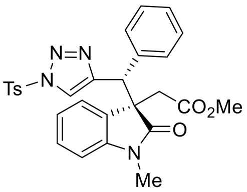

White solid, m.p. 85-93 °C; 89% yield; **^{1}H NMR (500 MHz, $CDCl_3$)** δ 8.29 (s, 1H), 7.96 (d, *J* = 8.5 Hz, 2H), 7.36 (d, *J* = 8.1 Hz, 2H), 7.22 (td, *J* = 7.7, 1.3 Hz, 1H), 7.17 (d, *J* = 7.3 Hz, 1H), 7.15 – 7.06 (m, 5H), 7.03 (t, *J* = 7.5 Hz, 1H), 6.53 (d, *J* = 7.8 Hz, 1H), 4.74 (s,1H), 3.34 (s, 3H), 2.99 (d, *J* = 16.4 Hz, 1H), 2.87 (s, 3H), 2.82 (d, *J* = 16.5 Hz, 1H), 2.43 (s, 3H). **^{13}C NMR (126 MHz, $CDCl_3$)** δ 177.7, 170.0, 147.3, 145.3, 144.4, 135.70, 133.2, 130.5, 129.7, 129.1, 129.0, 128.8, 128.0, 127.7, 123.8, 122.8, 122.3, 108.0, 52.9, 51.8, 50.8, 39.9, 26.0, 21.9. **IR** (KBr, cm^{-1}) γ 3012, 2926, 2854, 1741, 1707, 1613, 1496, 1471, 1381, 1356, 1202, 1174, 1122, 1033, 1009, 816, 753, 702, 682, 568, 490**.** **HRMS** (ESI) m/z $(M+H)^+$: calculated for $(C_{28}H_{27}N_4O_5S)^+$: 531.1697, found: 531.1707; **$[\alpha]^{20}_D$** = +77.8 (*c* = 0.73, $CHCl_3$); The product was analyzed by HPLC to determine the enantiomeric excess: >99% e.e. (CHIRALPAK IA, hexane/*i*-PrOH = 70/30, flow rate: 1.0 mL/min, T = 30 ℃, 230 nm), t_R (minor) = 9.92 min, t_R (major) = 11.81 min.

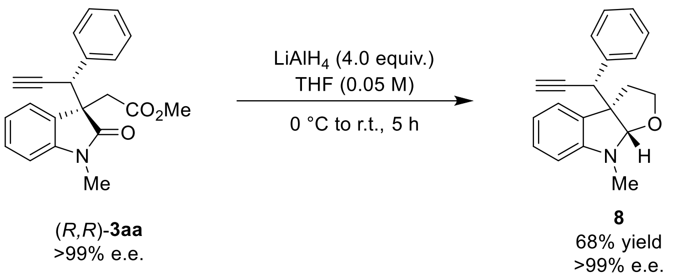

Under nitrogen atmosphere, a solution of $LiAlH_4$ (15.2 mg, 0.4 mmol, 4.0 equiv.) in anhydrous THF (1.0 ml) was added dropwise to a solution of (*R*,*R*)-**3aa** (33.3 mg, 0.10 mmol, 1.0 equiv.) in anhydrous THF (1.0 mL) at 0 °C. The resulting mixture was stirred at room temperature under nitrogen atmosphere for 5 hours. The reaction mixture was quenched with Na_2SO_4•$10H_2O$, filtered and extracted with ethyl acetate (10.0 mL x 3). The combined organic layers were dried over Na_2SO_4, filtered and concentrated in vacuo. The residue was purified by column chromatography on silica gel to give **8** (19.6 mg, 68% yield, >99% e.e.).

(*3aR,8aS*)-8-methyl-3a-((*R*)-1-phenylprop-2-yn-1-yl)-3,3a,8,8a-tetrahydro-2*H*-furo[2,3-*b*]indole (8)

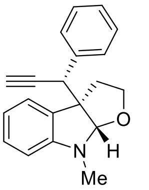

White solid, m.p. 96-100 °C; 68% yield; **^{1}H NMR (500 MHz, $CDCl_3$)** δ 7.21 (tt, *J* = 7.4, 3.1 Hz, 5H), 7.07 (td, *J* = 7.6, 1.3 Hz, 1H), 6.96 (dd, *J* = 7.3, 1.3 Hz, 1H), 6.63 (td, *J* = 7.4, 0.9 Hz, 1H), 6.23 (d, *J* = 7.8 Hz, 1H), 5.35 (s, 1H), 4.08 (d, *J* = 2.6 Hz, 1H), 3.96 (ddd, *J* = 8.5, 7.2, 1.2 Hz, 1H), 3.42 (ddd, *J* = 11.3, 8.5, 4.7 Hz, 1H), 2.78 (s, 3H), 2.65 (td, *J* = 11.5, 7.3 Hz, 1H), 2.34 (d, *J* = 2.5 Hz, 1H), 2.17 – 2.10 (m, 1H). **^{13}C NMR (126 MHz, $CDCl_3$)** δ 151.9, 137.0, 130.1, 129.2, 128.9, 127.8, 127.5, 124.3, 116.9, 105.3, 101.2, 83.5, 72.7, 67.5, 60.7, 44.4, 38.3, 31.1. **IR** (KBr, cm^{-1}) *γ* 3290, 3054, 3029, 2924, 2870, 1659, 1607, 1495, 1453, 1390, 1303, 1262, 1220, 1156, 1108, 1011, 931, 913, 799, 750, 702, 648, 601. **HRMS** (ESI) m/z $(M+H)^+$: calculated for $(C_{20}H_{20}NO)^+$: 290.1539, found: 290.1538; **$[\alpha]^{20}_D$**=-141.2 (*c* = 0.38, $CHCl_3$); The product was analyzed by HPLC to determine the enantiomeric excess: >99% e.e. (CHIRALPAK IG, hexane/*i*-PrOH = 80/20, flow rate: 1.0 mL/min, T = 30 ℃, 254 nm), t_R (major) = 4.38 min, t_R (major) = 4.74 min.

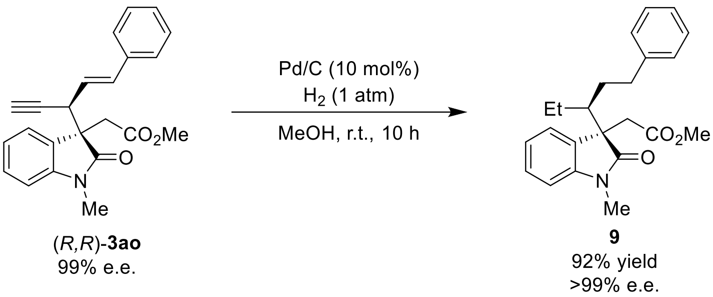

To a stirring solution of (*R*,*R*)-**3ao** (35.9 mg, 0.10 mmol, 1.0 equiv.) in MeOH (5.0 mL) was slowly added palladium onactivated charcoal (10%, 20.0 mg) at room temperature. The resulting mixture was stirred at room temperature in an atmosphere of hydrogen gas for 10 hours. The mixture was filtered and concentrated under reduced pressure. The residue was purified by column chromatography on silica gel to give **9** (33.6 mg, 92% yield, >99%e.e.).

Methyl 2-((*R*)-1-methyl-2-oxo-3-((*S*)-1-phenylpentan-3-yl)indolin-3-yl)acetate (9)

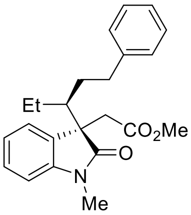

Colorless liquid; 92% yield; >95:5 d.r.; **^{1}H NMR (400 MHz, $CDCl_3$)** δ 7.33 – 7.21 (m, 3H), 7.20 – 7.12 (m, 2H), 7.10 – 7.04 (m, 2H), 7.01 (td, *J* = 7.5, 1.1 Hz, 1H), 6.89 – 6.80 (m, 1H), 3.39 (s, 3H), 3.25 (s, 3H), 3.08 (d, *J* = 16.0 Hz, 1H), 2.99 (d, *J* = 16.0 Hz, 1H), 2.47 (dd, *J* = 9.6, 7.4 Hz, 2H), 1.80 (ddd, *J* = 8.6, 6.7, 3.5 Hz, 1H), 1.71 – 1.54 (m, 2H), 1.46 – 1.34 (m, 1H), 1.28 (ddt, *J* = 14.4, 10.3, 7.3 Hz, 1H), 0.92 (t, *J* = 7.4 Hz, 3H). **^{13}C NMR (101 MHz, $CDCl_3$)** δ 179.5, 170.6, 144.9, 142.5, 130.8, 128.4, 128.4, 125.8, 123.3, 122.2, 108.0, 53.1, 51.7, 47.0, 40.2, 34.8, 31.8, 26.4, 23.1, 13.4. **IR** (KBr, cm^{-1}) *γ* 3470, 2929, 1959, 1709, 1611, 1492, 1452, 1343, 1260, 1168, 1091, 1026, 814, 755, 701, 544. **HRMS** (ESI) m/z $(M+Na)^+$: calculated for $(C_{23}H_{27}NO_3Na)^+$: 388.1883, found: 388.1892; **$[\alpha]^{20}_D$** = +10.9 (*c* = 0.61, $CHCl_3$); The product was analyzed by HPLC to determine the enantiomeric excess: >99% e.e. (CHIRALPAK IG, hexane/*i*-PrOH = 85/15, flow rate: 1.0 mL/min, T = 30 ℃, 254 nm), t_R(major) = 17.73 min, t_R (minor) = 19.15 min.

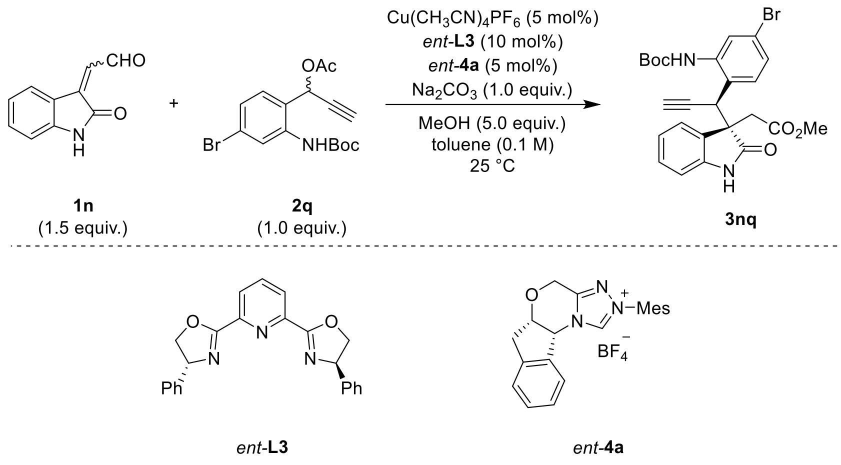

To a flame-dried and N_2-purged Schlenk tube were added $Cu(CH_3CN)_4PF_6$ (0.15 mmol, 55.9 mg) and pyridine bisoxazoline ligand *ent*-**L3** (0.3 mmol, 110.9 mg). The vial was sealed, purged and back filled with N_2 three times before adding toluene (15.0 mL) at 25 °C. The resulting solution was stirred at 25 °C for 1 hour. Then, isatin-derived enal **1n** (4.5 mmol, 779.3 mg), NHC precatalyst **4a** (0.15 mmol, 62.9 mg) Na_2CO_3 (3.0mmol, 318.0 mg), MeOH (15.0 mmol, 600 μL) and a solution of propargylic acetate **2q** (3.0 mmol, 1104.7 mg) in toluene (15.0 mL) were added. The resulting solution was stirred at 25 °C for 12 hours and then quenched with saturated NH_4Cl aqueous solution (10.0 mL). The resulting solution was extracted with ethyl acetate (15.0 mL x 3). The combined organic layers were dried over Na_2SO_4, filtered and concentrated in vacuo. The diastereomeric ratio was determined by ^1H NMR analysis of the crude reaction mixture. The residue was purified by column chromatography on silica gel (petroleum ether: ethyl acetate = 3:1-2:1) to afford desired product **3nq** (0.94 g, 82:18 d.r., 93% e.e.).

Methyl 2-((*S*)-3-((*S*)-1-(4-bromo-2-((*tert*-butoxycarbonyl)amino)phenyl)prop-2-yn-1-yl)-2-oxoindolin-3-yl)acetate (3nq)

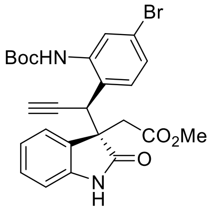

White solid, m.p. 90-96 °C; 61% yield; 82:18 d.r.; **^{1}H NMR (500 MHz, $CDCl_3$)** δ 8.35 (s, 1H), 7.78 (s, 1H), 7.20 (td, *J* = 7.7, 1.3 Hz, 1H), 7.09 (m, 3H), 6.98 (td, *J* = 7.5, 1.0 Hz, 2H), 6.80 (d, *J* = 7.8 Hz, 1H), 4.38 (d, *J* = 2.6 Hz, 1H), 3.44 (s, 3H), 3.28 (d, *J* = 16.5 Hz, 1H), 2.99 (d, *J* = 16.4 Hz, 1H), 2.38 (d, *J* = 2.6 Hz, 1H), 1.48 (s, 9H). **^{13}C NMR (126 MHz, $CDCl_3$)** δ 179.2, 170.1, 153.0, 141.9, 137.7, 133.0, 129.2, 128.5, 127.1, 124.3,

122.4, 122.1, 110.1, 80.9, 80.0, 74.5, 53.4, 51.8, 40.2, 38.7, 28.3. **IR** (KBr, cm^{-1}) *γ* 3299, 2964, 2928, 1716, 1621, 1575, 1506, 1472, 1438, 1367, 1261, 1235, 1158, 1051, 1024, 870, 804, 755, 666, 580, 492. **HRMS** (ESI) m/z $(M+Na)^+$: calculated for $(C_{25}H_{25}BrN_2O_5Na)^+$: 535.0833, found: 535.0844; **$[\alpha]^{20}_D$** = -31.2 (*c* = 0.87, $CHCl_3$); The product was analyzed by HPLC to determine the enantiomeric excess: 93% e.e. (CHIRALPAK IA, hexane/*i*-PrOH = 85/15, flow rate: 1.0 mL/min, T = 30 ℃, 254 nm), t_R (major) = 13.58 min, t_R (minor) = 16.16 min.

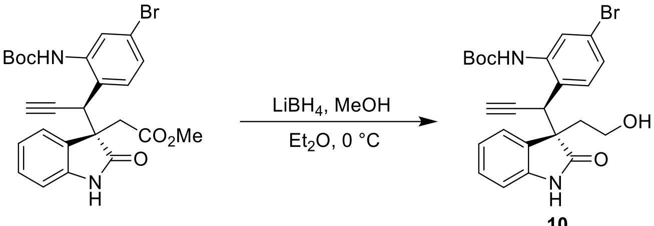

3nq
93% e.e.

10
60% yield
94% e.e.

A flame-dried Schlenk tube equipped with a magnetic stir bar was charged with **3nq** (51.3 mg, 93% e.e., 0.1 mmol, 1.0 equiv.), and 2.0 mL of Et_2O was added to the Schlenk tube under argon. After cooling down to 0 ℃, $LiBH_4$ (21.8mg, 1.0 mmol, 10.0 equiv.) and methanol (40 μL, 1.0 mmol, 10.0 equiv.) were added subsequently. The mixture was stirred at 0 ℃ until the starting materials were consumed (monitoring by TLC). The reaction mixture was quenched with water (5.0 mL). The resulting solution was extracted with ethyl acetate (5.0 mL x 3). The combined organic layers were dried over Na_2SO_4, filtered and concentrated in vacuo. The crude material was purified by column chromatography to afford compound **10** (29.1 mg, 60% yield, 94% e.e.).

***tert*-Butyl (5-bromo-2-((*S*)-1-((*S*)-3-(2-hydroxyethyl)-2-oxoindolin-3-yl)prop-2-yn -1-yl)phenyl)carbamate (10)**

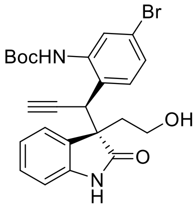

White solid, m.p. 109-111 °C; 60% yield; **^{1}H NMR (500 MHz, $CDCl_3$)** δ 8.84 (s, 1H), 7.75 (br, 1H), 7.24 – 7.05 (m, 3H), 6.97 (t, *J* = 7.6 Hz, 1H), 6.84 (d, *J* = 7.5 Hz, 1H), 6.76 (d, *J* = 7.8 Hz, 1H), 4.41 (s, 1H), 3.38 (m, *J* = 11.8, 7.1, 3.7 Hz, 1H), 3.13 (s, 1H), 2.54 (m, *J* = 7.2 Hz, 1H), 2.15 (s, 1H), 1.90 (m, *J* = 14.5, 4.5 Hz, 1H), 1.46 (s, 9H). **^{13}C NMR (101 MHz, $CDCl_3$)** δ 181.2, 142.1, 138.2, 133.3, 128.9, 128.3, 127.2, 125.4,

110.4, 81.2, 81.0, 59.1, 55.6, 40.5, 36.9, 28.4. **IR** (KBr, cm^{-1}) *γ* 3298, 2927, 2854, 1713, 1621, 1592, 1576, 1514, 1471, 1408, 1392, 1368, 1235, 1158, 1050, 1021, 870, 804, 754, 665, 575, 493. **HRMS** (ESI) m/z $(M+Na)^+$: calculated for $(C_{24}H_{25}BrN_2O_4Na)^+$: 504.0890, found: 507.0897; $[\alpha]^{20}_D$ = -6.7 (*c* = 0.65, $CHCl_3$); The product was analyzed by HPLC to determine the enantiomeric excess: 94% e.e. (CHIRALPAK IA, hexane/*i*-PrOH = 70/30, flow rate: 1.0 mL/min, T = 30 ℃, 254 nm), t_R (major) = 4.44 min, t_R (minor) = 5.90 min.

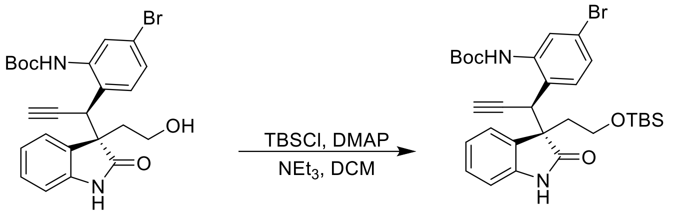

10 **11** 80% yield

To a flame-dried and N_2-purged Schlenk tube were added **10** (48.5 mg, 94% e.e., 0.10 mmol, 1.0 equiv.), DMAP (1.2 mg, 0.01 mmol, 10 mol%) and TBSCl (45.2 mg, 0.3 mmol, 3.0 equiv.). The vial was then sealed, purged and backfilled with N_2 three times before adding NEt_3 (42 μL, 0.3 mmol, 3.0 equiv.) and DCM (2.0 mL) at room temperature. The resulting mixture was stirred at room temperature under nitrogen atmosphere until the reaction completed (monitoring by TLC). After concentrated under reduced pressure, the residue was purified by column chromatography on silica gel to give **11** (48.0 mg, 80% yield).

***tert*-Butyl (5-bromo-2-((*S*)-1-((*S*)-3-(2-((tert-butyldimethylsilyl)oxy)ethyl)-2-oxoindolin-3-yl)prop-2-yn-1-yl)phenyl)carbamate (11)**

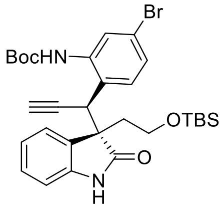

White solid, m.p. 90-97 °C; 80% yield; **^{1}H NMR (400 MHz, $CDCl_3$)** δ 8.46 (s, 1H), 7.89 (s, 1H), 7.21 (td, *J* = 7.7, 1.3 Hz, 1H), 7.16 – 7.01 (m, 2H), 6.98 (td, *J* = 7.5, 1.1 Hz, 1H), 6.91 (d, *J* = 7.4 Hz, 1H), 6.84 (dt, *J* = 7.7, 0.8 Hz, 1H), 4.26 (d, *J* = 2.7 Hz, 1H), 3.27 (qdd, *J* = 10.2, 7.7, 5.2 Hz, 2H), 2.50 (dt, *J* = 13.4, 7.8 Hz, 1H), 2.25 (d, *J* = 2.5 Hz, 1H), 2.00 (ddd, *J* = 13.4, 6.0, 4.3 Hz, 1H), 1.47 (s, 9H), 0.74 (s, 9H), -0.19 (s, 3H), -0.21 (s, 3H). **^{13}C NMR (101 MHz, $CDCl_3$)** δ 180.3, 152.9, 141.9, 138.0, 133.5, 128.9, 128.1, 126.7, 125.3, 122.4, 122.1, 110.1, 80.9, 80.6, 74.3, 59.4, 55.2, 37.0, 28.4,

25.9, 18.3, -5.7, -5.7. **IR** (KBr, cm^{-1}) γ 3303, 2955, 2928, 2856, 1712, 1619, 1592, 1577, 1509, 1473, 1408, 1382, 1368, 1340, 1250, 1158, 1110, 1051, 1022, 853, 754, 663, 491. **HRMS** (ESI) m/z $(M+Na)^+$: calculated for $(C_{30}H_{39}BrN_2O_4SiNa)^+$: 621.1755, found: 621.1763; $[\alpha]^{20}_D$ = -11.5 (c = 0.62, $CHCl_3$).

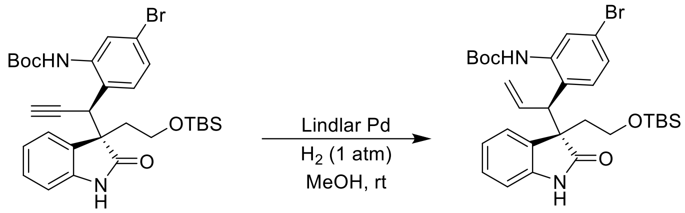

11

12
93% yield
94% e.e.

To a stirring solution of **11** (60.0 mg, 0.10 mmol, 1.0 equiv.) in MeOH (5.0 mL) was slowly added Lindlar Pd (5%, 20.0 mg) at room temperature. The resulting mixture was stirred at room temperature in an atmosphere of hydrogen gas until the reaction completed (monitoring by TLC). The mixture was filtered and concentrated under reduced pressure. The residue was purified by column chromatography on silica gel to give **12** (56.0 mg, 93% yield, 94% e.e.).

***tert*-Butyl (5-bromo-2-((*S*)-1-((*S*)-3-(2-((*tert*-butyldimethylsilyl)oxy)ethyl)-2-oxoindolin-3-yl)allyl)phenyl)carbamate(12)**

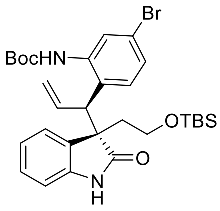

White solid; 93% yield; **^{1}H NMR (500 MHz, $CDCl_3$)** δ 7.87 (s, 1H), 7.69 (s, 1H), 7.18 (td, J = 7.5, 1.6 Hz, 1H), 7.11 (dd, J = 8.5, 2.2 Hz, 1H), 7.06 – 6.93 (m, 3H), 6.74 (d, J = 7.7 Hz, 1H), 6.31 (s, 1H), 6.16 (dt, J = 17.8, 9.6 Hz, 1H), 5.15 (dd, J = 16.8, 1.6 Hz, 1H), 5.09 (dd, J = 10.0, 1.5 Hz, 1H), 3.85 (d, J = 9.4 Hz, 1H), 3.32 – 3.18 (m, 2H), 2.44 (dt, J = 13.6, 7.9 Hz, 1H), 1.88 (ddd, J = 13.6, 6.2, 4.1 Hz, 1H), 1.53 (s, 9H), 0.74 (s, 9H), -0.18 (s, 3H), -0.22 (s, 3H). **^{13}C NMR (126 MHz, $CDCl_3$)** δ 180.7, 153.2, 141.4, 137.0, 135.0, 131.0, 129.4, 128.7, 127.4, 124.7, 122.0, 121.0, 118.8, 109.9, 81.0, 59.4, 55.2, 51.1, 37.8, 28.5, 25.9, 18.3, -5.7. **IR** (KBr, cm^{-1}) γ 2956, 2927, 2855, 1707, 1619, 1592, 1572, 1509, 1472, 1367, 1258, 1159, 1090, 1050, 1022, 923, 836, 806, 776, 755, 666, 492. $[\alpha]^{20}_D$ = +44.6 (c = 0.26, $CHCl_3$); **HRMS** (ESI) m/z $(M+Na)^+$: calculated for $(C_{30}H_{41}BrN_2O_4SiNa)^+$: 623.1911, found: 623.1927; The product was analyzed by HPLC to determine the enantiomeric excess: 94% e.e.

(CHIRALPAK IA, hexane/*i*-PrOH = 98/2, flow rate: 1.0 mL/min, T = 30 ℃, 220 nm), t_R (minor) = 23.47 min, t_R (major) = 32.21 min.

Nonlinear Effect Studies

The non-linear effect study were conducted by building a relationship between the e.e. value of the NHC precatalyst **4a** and that of the product (*R*,*R*)-**3aa**. The specified e.e. values of NHC precatalyst **4a** was made by mixing certain amounts of optically pure NHC precatalyst **4a** with optically pure NHC precatalyst *ent*-**4a**. Six reactions containing NHC precatalyst of racemic, 21%, 34%, 60%, 80%, and 99% optical purity were run in parallel.

To a flame-dried and N_2-purged Schlenk tube were added $Cu(CH_3CN)_4PF_6$ (0.005 mmol, 5 mol%) and pyridine bisoxazoline ligand **L-S11** (0.01 mmol, 10 mol%). The vial was sealed, purged and backfilled with N_2 three times before adding THF (0.5 mL) at 25 ℃. The resulting solution was stirred at 25 ℃ for 1 hour. Then, isatin-derived enal **1a** (0.15 mmol), NHC precatalyst **4a** with different e.e. values (0.005 mmol, 5 mol%), Na_2CO_3 (0.1mmol), MeOH (0.5 mmol) and propargylic acetate **2a** (0.1 mmol) in THF (0.5 mL) were added. The resulting solution was stirred at 25 ℃ for 12 hours and then quenched with saturated aqueous NH_4Cl solution (5.0 mL). The resulting solution was extracted with ethyl acetate (5.0 mL x 3). The combined organic layers were dried over Na_2SO_4, filtered and concentrated in vacuo. The diastereomeric ratio was determined by ^1H NMR analysis of the crude reaction mixture. The residue was purified by column chromatography on silica gel (petroleum ether: ethyl acetate = 5:1-2:1) to afford desired product (*R*,*R*)-**3aa**. The product was analyzed by HPLC (CHIRALPAK IG, hexane/*i*-PrOH = 70/30, flow rate: 1.0 mL/min, T = 30 ℃, 230 nm), t_R (major) = 11.26 min, t_R (minor) = 16.01 min. A graph of e.e. of product vs. e.e. of catalyst was then plotted.

Supplementary Table 10. Nonlinear experiments

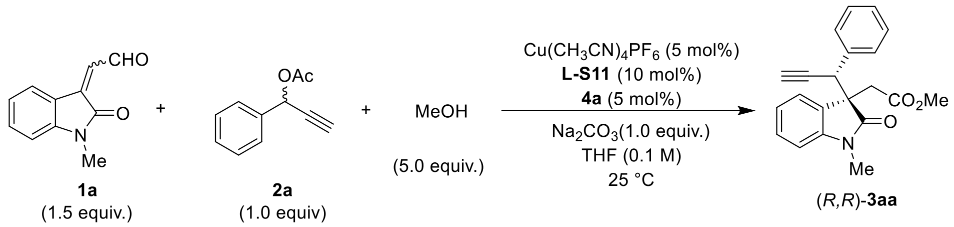

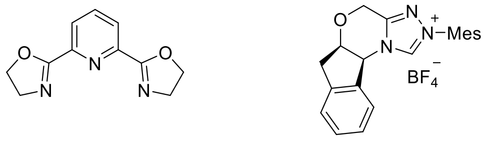

entry[a]	e.e. of NHC precatalyst **4a**(%)	yield (%)	d.r.	e.e. of (*R*,*R*)-**3aa**(%)
1	0	70	82:18	0
2	21	73	82:18	14
3	34	72	82:18	24
4	60	74	81:19	42
5	80	74	81:19	58
6	99	71	80:20	68

[a]Unless noted, reaction conditions: $Cu(CH_3CN)_4PF_6$ (5 mol%), pyridine bisoxazoline ligand **L-S11** (10 mol%) were stirred in THF (0.5 mL) at 25 ℃ for 1 h under N_2, then NHC precatalyst **4a**(5 mol%), **1a** (0.15 mmol), **2a** (0.1 mmol), MeOH (0.5 mmol), Na_2CO_3 (0.1 mmol) and THF (0.5 mL) were added to the reaction mixture and stirred at 25 ℃ for 12 h under N_2. The yield and diastereomeric ratio (d.r.) were determined by ^{1}H NMR spectroscopy. The enantiomeric excess (e.e.) was determined by HPLC.

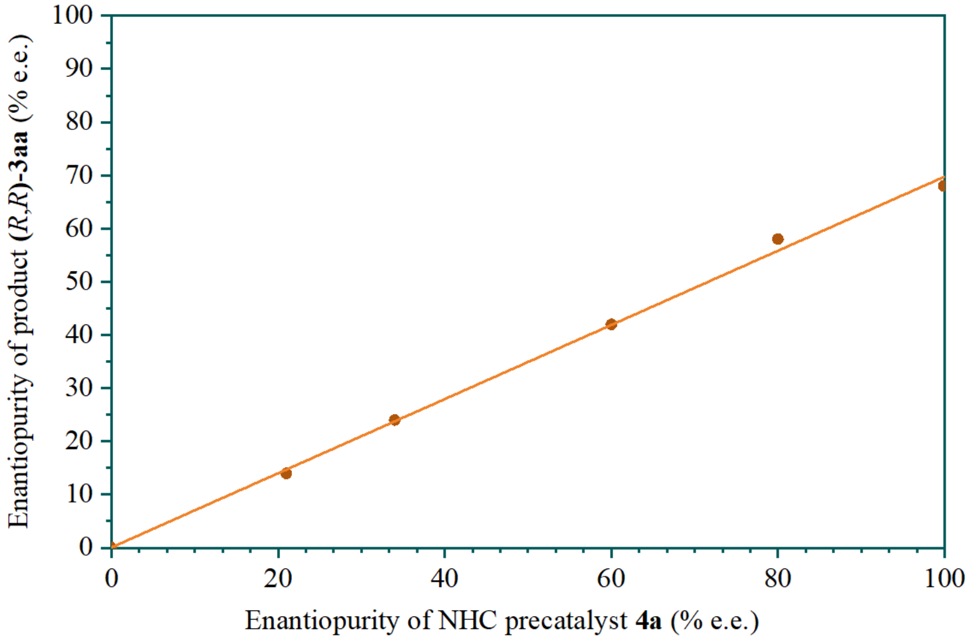

ESI-MS Analysis

HRMS analysis of Cu^{I}(**L3**)(**4a**)$^{+}$

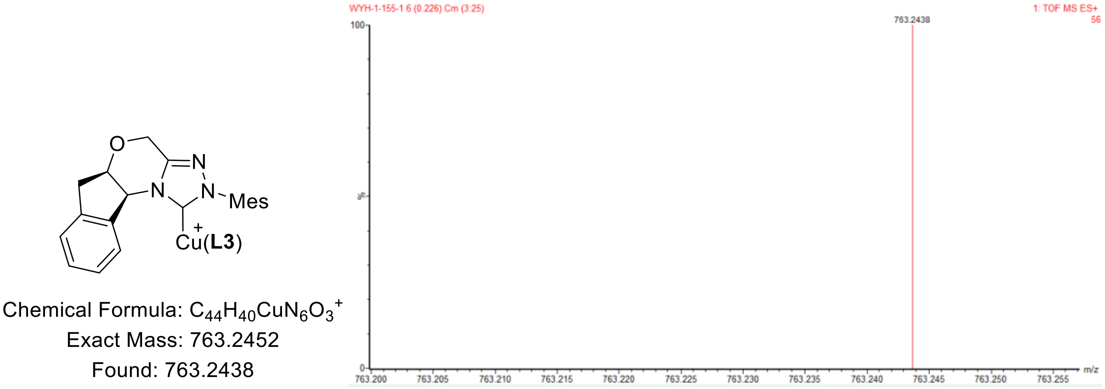

To a flame-dried and N_2-purged Schlenk tube were added $Cu(CH_3CN)_4PF_6$ (0.005 mmol, 5 mol%) and pyridine bisoxazoline ligand **L3** (0.01 mmol, 10 mol%). The vial was sealed, purged and backfilled with N_2 three times before adding THF (0.5 mL) at 25 °C. The resulting solution was stirred at 25 °C for 1 hour. Then, isatin-derived enal **1a** (0.15 mmol), NHC precatalyst **4a** (0.005 mmol, 5 mol%), Na_2CO_3 (0.1mmol), MeOH (0.5 mmol) and a solution of propargylic acetate **2a** (0.1 mmol) in THF (0.5 mL) were added. The resulting solution was stirred at 25 °C for 1 hour. HRMS analysis of the reaction mixture showed the presence of Cu^{I}(**L3**)(**4a**)$^{+}$.

NMR Studies

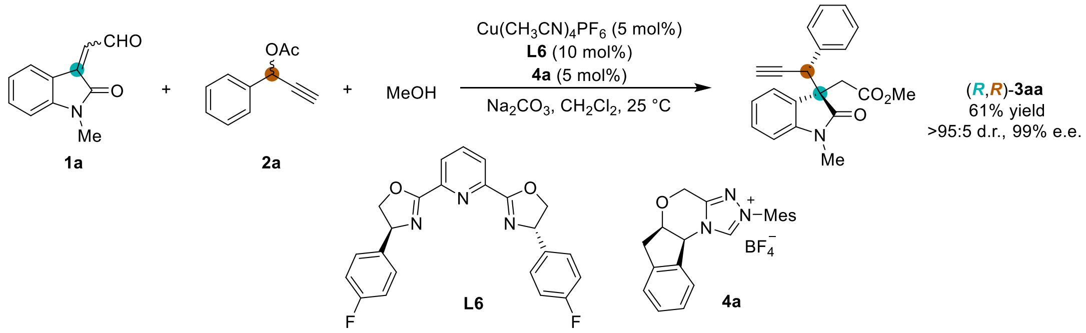

^{19}F NMR studies (in CD_2Cl_2) on the coordination effect of copper salt $Cu(CH_3CN)_4PF_6$, a fluorine-substituted Pybox ligand **L6** (2,6-bis((*S*)-4-(4-fluorophenyl)-4,5-dihydrooxazol-2-yl)pyridine),[7] and an NHC catalyst **4a** were carried out. The ^{19}F NMR spectra of Pybox **L6** and Pybox **L6**-copper complex were collected and are shown in (1) and (2) (Supplementary Figure 1). The ^{19}F NMR spectrum of the catalyst system of $Cu(CH_3CN)_4PF_6$, Pybox

L6, and NHC **4a** in a ratio of 1:2:1 found that a considerable amount of the Pybox **L6**-copper complex was formed (Supplementary Figure 1, (3)). The same result was observed under the standard reaction conditions (Supplementary Figure 1, (4)). We speculated that the copper complex including NHC-**4a** as a ligand cannot be detected in our catalytic system via NMR analysis, which may be due to the instability or extremely low concentration (can be detected via ESI-MS Analysis) of the complex.

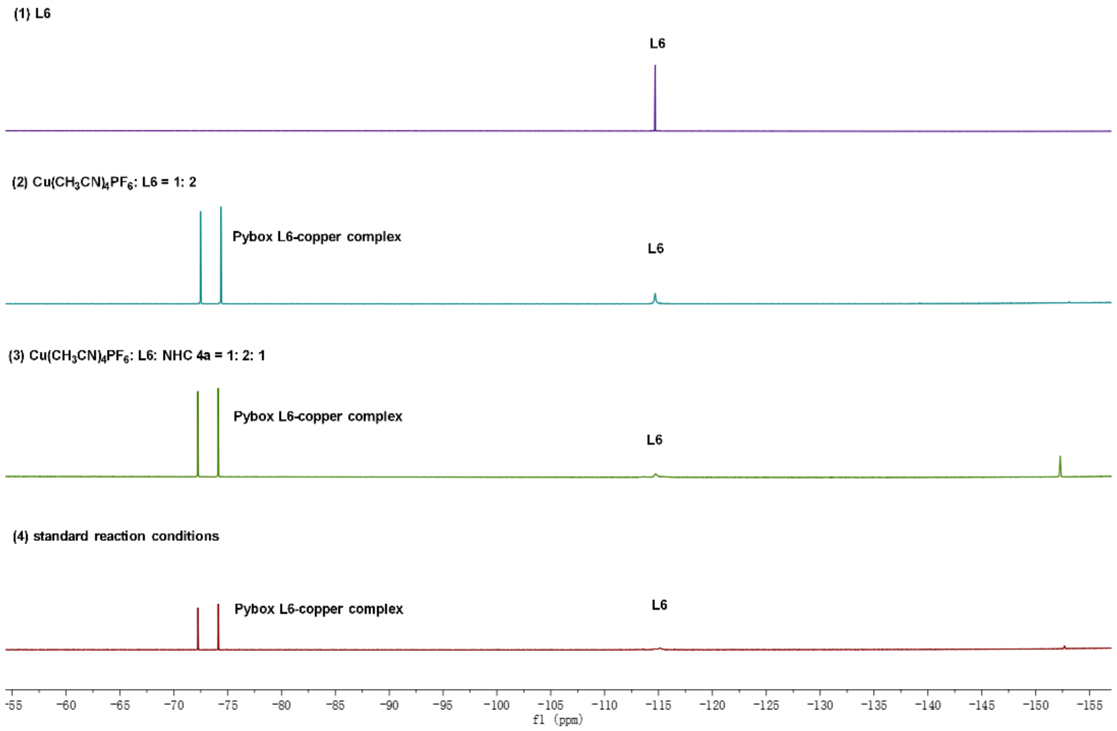

Supplementary Figure 1. ^{19}F NMR studies.

X-ray Single Crystal Data

(*R*,*R*)-**3aa**

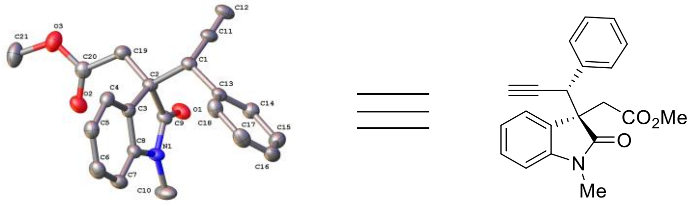

Empirical formula	$C_{21}H_{19}NO_3$
Formula weight	333.37
Temperature/K	192.99
Wavelength/Å	1.34139
Crystal system	Hexagonal
Space group	$P6_1$
a/Å	9.47499(10)
b/Å	9.47499(10)
c/Å	70.0389(7)
α/°	90
β/°	90
γ/°	120
Volume/Å^3	5445.37(13)
Z	12
ρ_{calc} Mg/m^3	1.220
μ/mm^{-1}	0.423
F(000)	2112
Crystal size/mm^3	0.08 x 0.05 x 0.03
2Θ range for data collection/°	3.294 to 54.891
Index ranges	-10<=h<=11, -11<=k<=9, -85<=l<=85
Reflections collected	79418
Independent reflections	6910 [R(int) = 0.0377]
Data/restraints/parameters	6910 / 1 / 455
Goodness-of-fit on F^2	1.031
Final R indexes [I>=2σ (I)]	R_1 = 0.0332, wR_2 = 0.0790
Final R indexes [all data]	R_1 = 0.0398, wR_2 = 0.0840
Largest diff. peak/hole / e Å^{-3}	0.187 and -0.200
Absolute structure parameter	0.13(6)

(*R*,*S*)-**3aa**

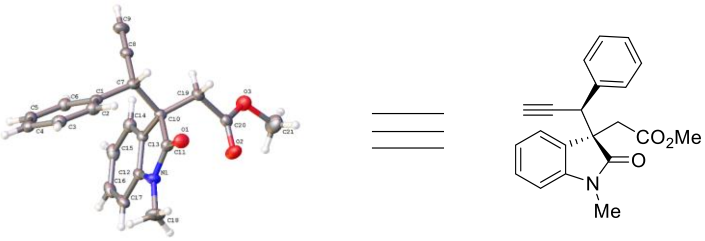

Empirical formula	$C_{21}H_{19}NO_3$
Formula weight	333.37
Temperature/K	173.0
Wavelength/Å	1.34139
Crystal system	Monoclinic
Space group	P1 21 1
a/Å	8.81790(10)
b/Å	9.00430(10)
c/Å	22.8050(3)
α/°	90
β/°	97.2030
γ/°	90
Volume/Å^3	1796.40(13)
Z	4
ρ_{calc} Mg/m^3	1.233
μ/mm^{-1}	0.427
F(000)	704
Crystal size/mm^3	0.07 x 0.06 x 0.05
2Θ range for data collection/°	3.399 to 54.939
Index ranges	-10<=h<=10, -10<=k<=10, -26<=l<=27
Reflections collected	27020
Independent reflections	6758 [R(int) = 0.0310]
Data/restraints/parameters	6758 / 1 / 455
Goodness-of-fit on F^2	1.084
Final R indexes [I>=2σ (I)]	R_1 = 0.0322, wR_2 = 0.0834
Final R indexes [all data]	R_1 = 0.0341, wR_2 = 0.0852
Largest diff. peak/hole / e Å^{-3}	0.181 and -0.131
Absolute structure parameter	0.04(6)

Supplementary Figures

NMR Spectra

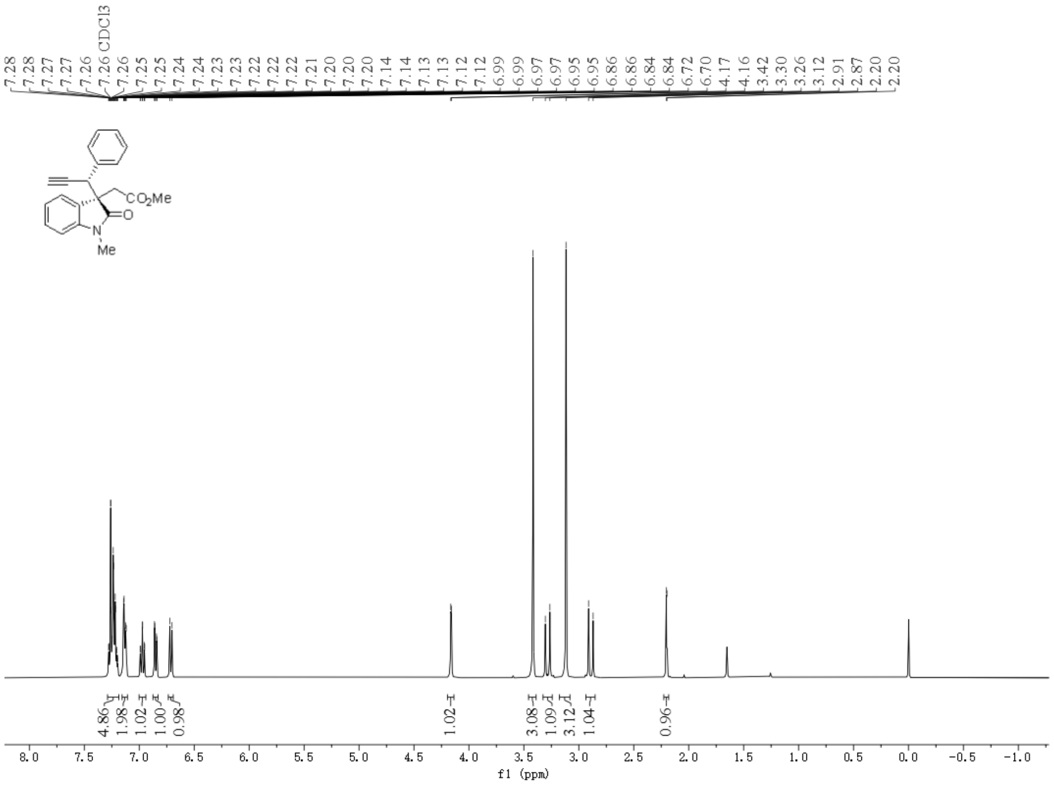

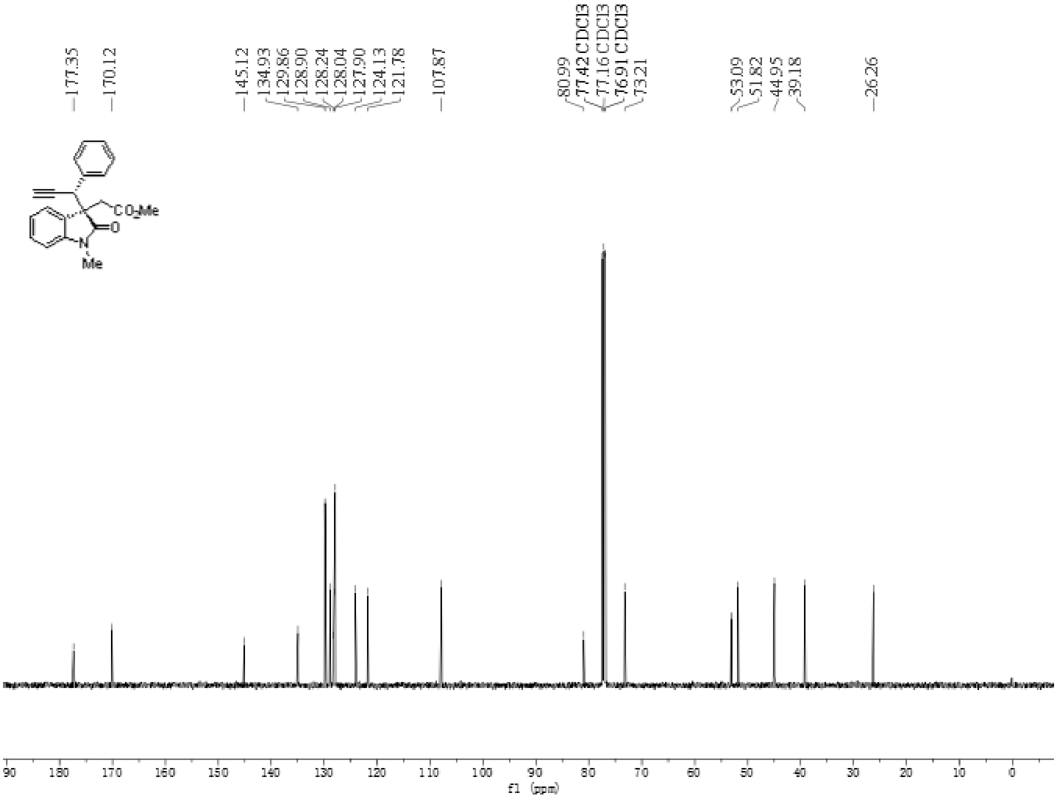

Supplementary Fig. 2. ^{1}H NMR & ^{13}C NMR spectra of compound (*R*,*R*)-**3aa**

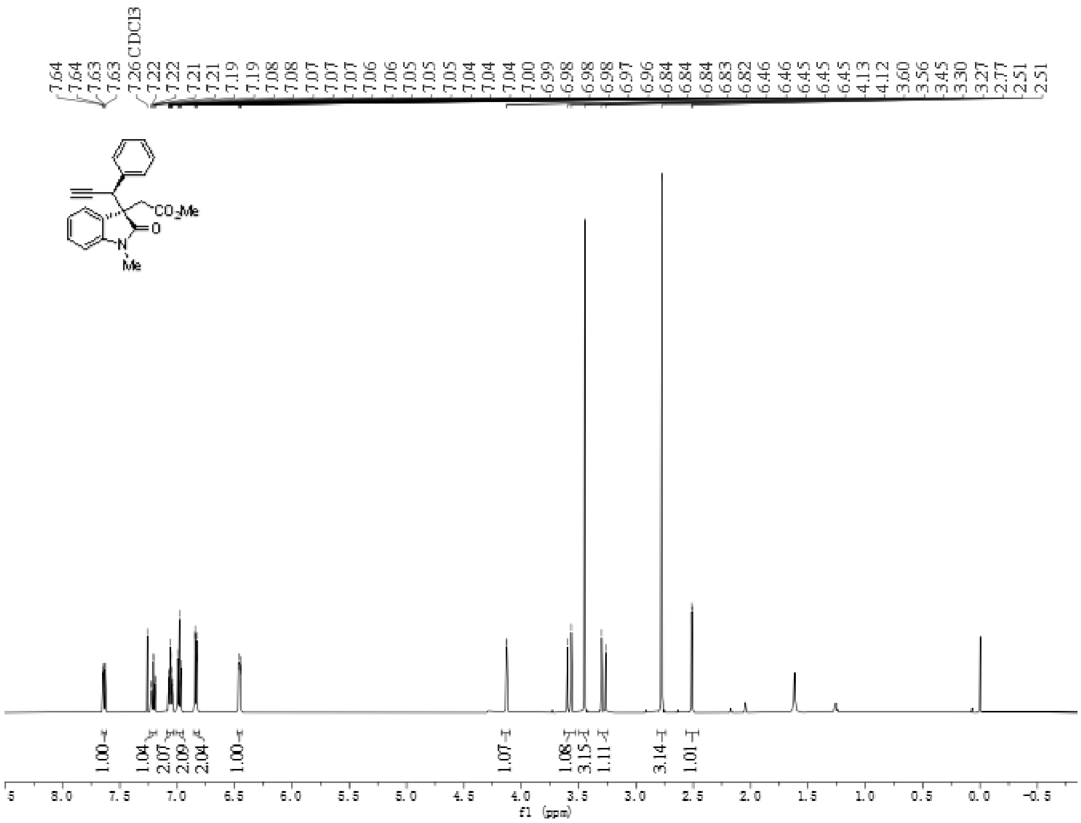

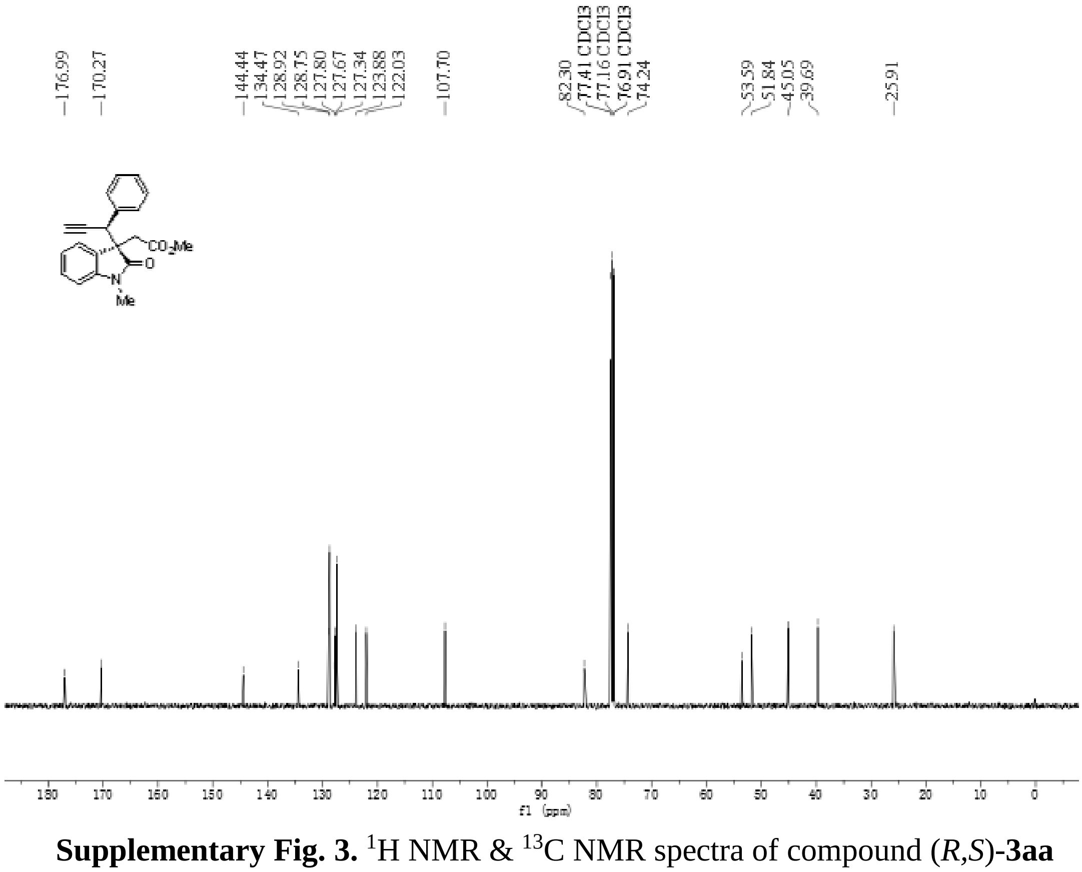

Supplementary Fig. 3. ^{1}H NMR & ^{13}C NMR spectra of compound (*R*,*S*)-**3aa**

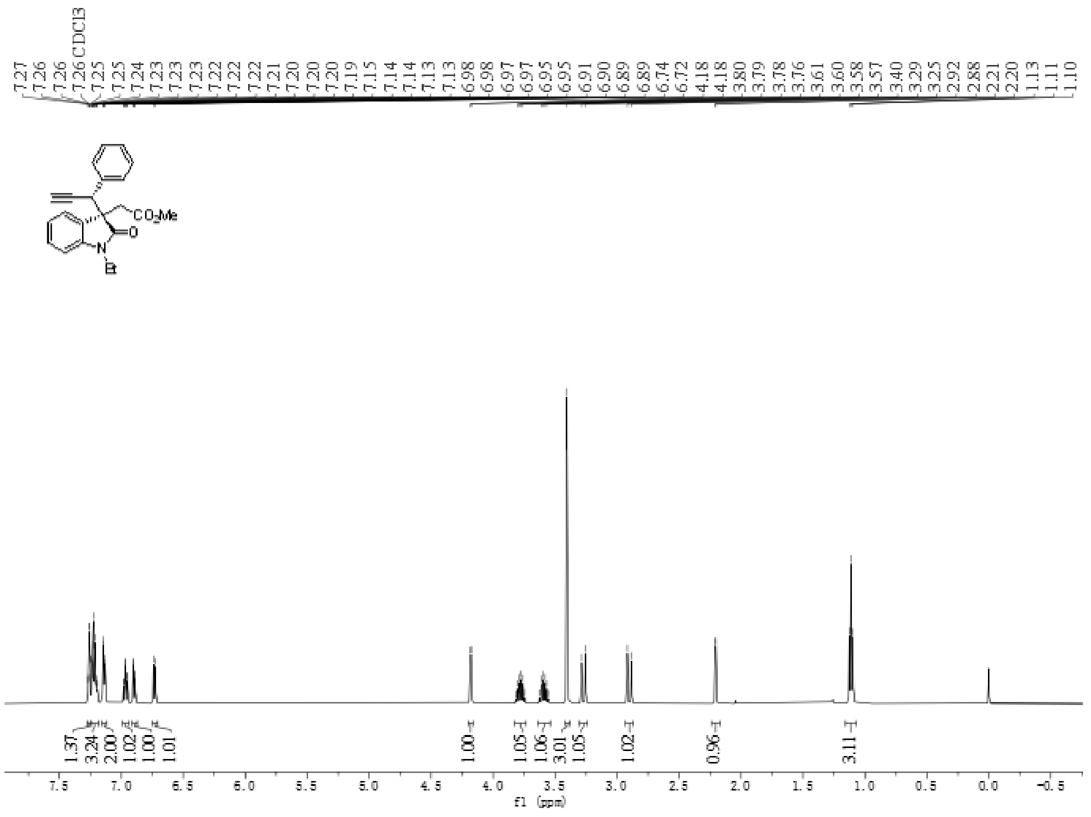

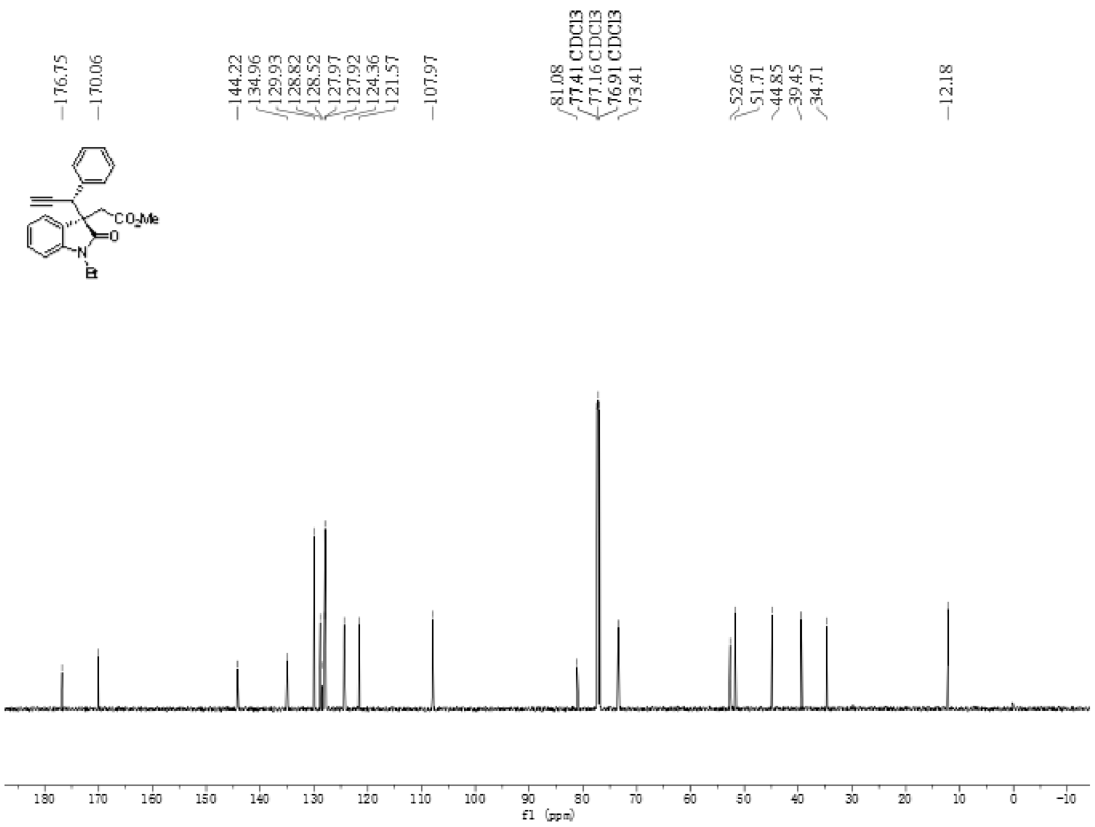

Supplementary Fig. 4. ^{1}H NMR & ^{13}C NMR spectra of compound (*R*,*R*)-**3ba**

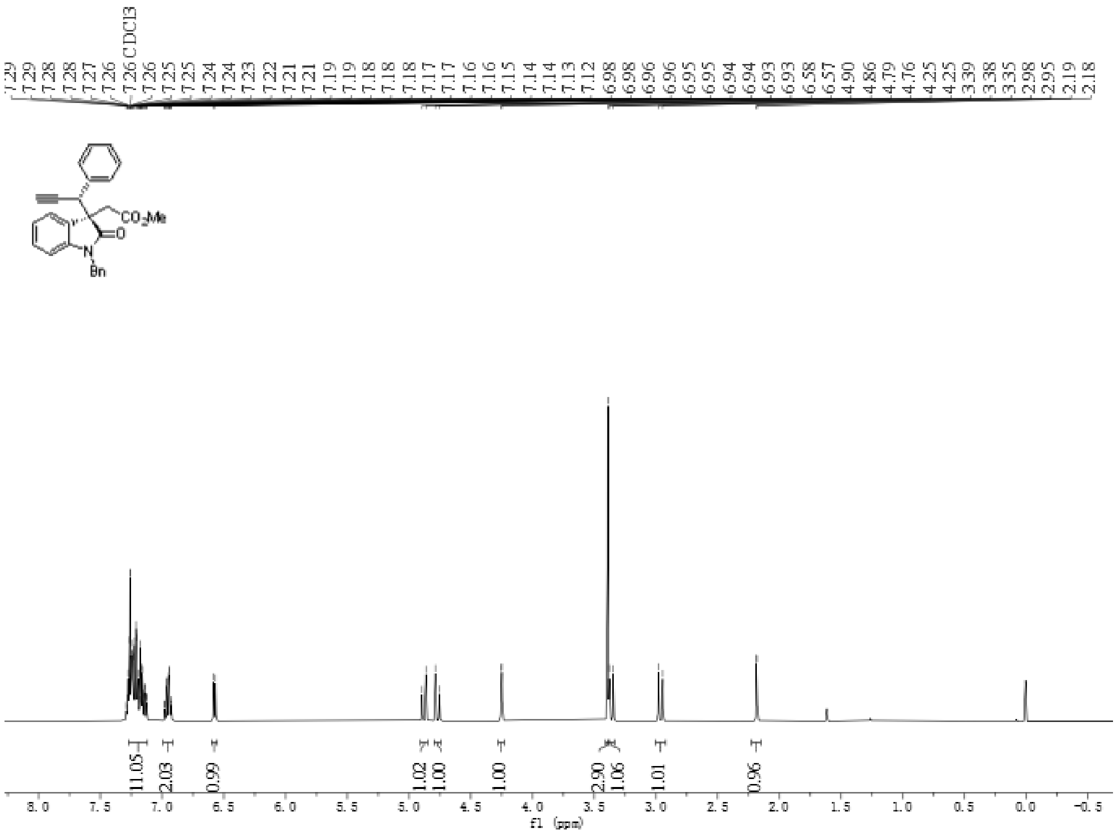

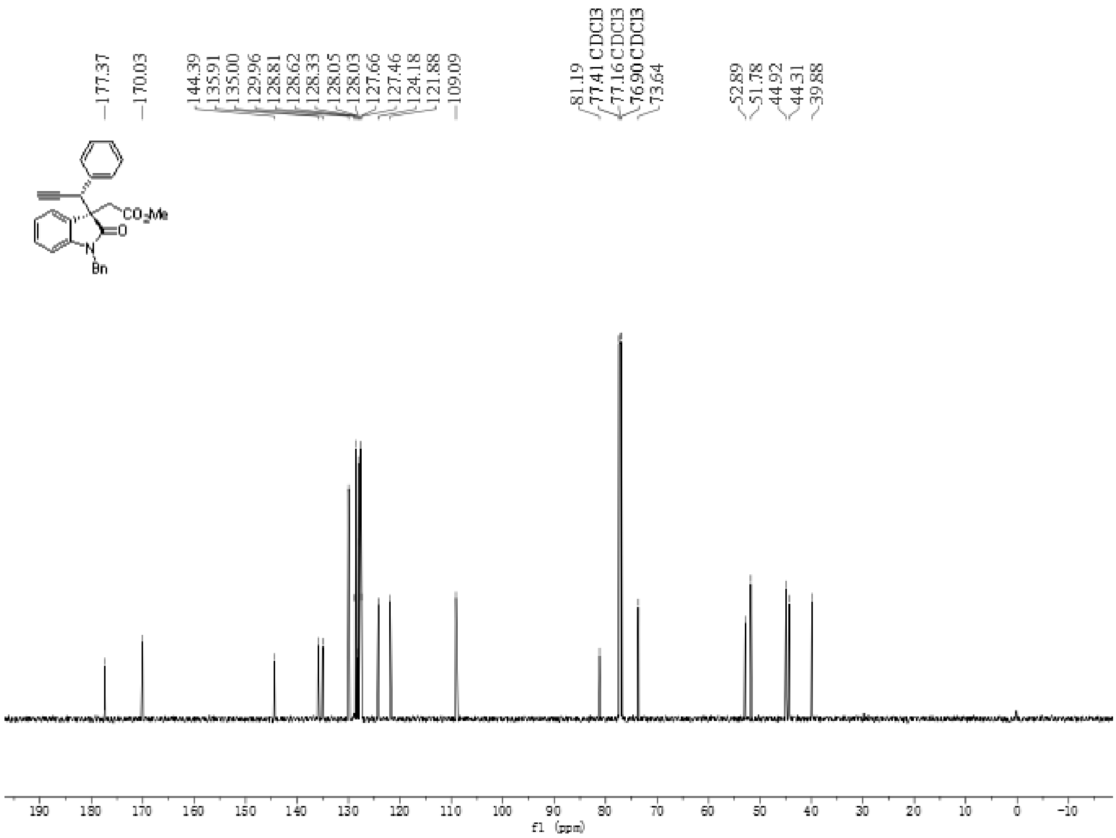

Supplementary Fig. 5. ^{1}H NMR & ^{13}C NMR spectra of compound (*R*,*R*)-**3ca**

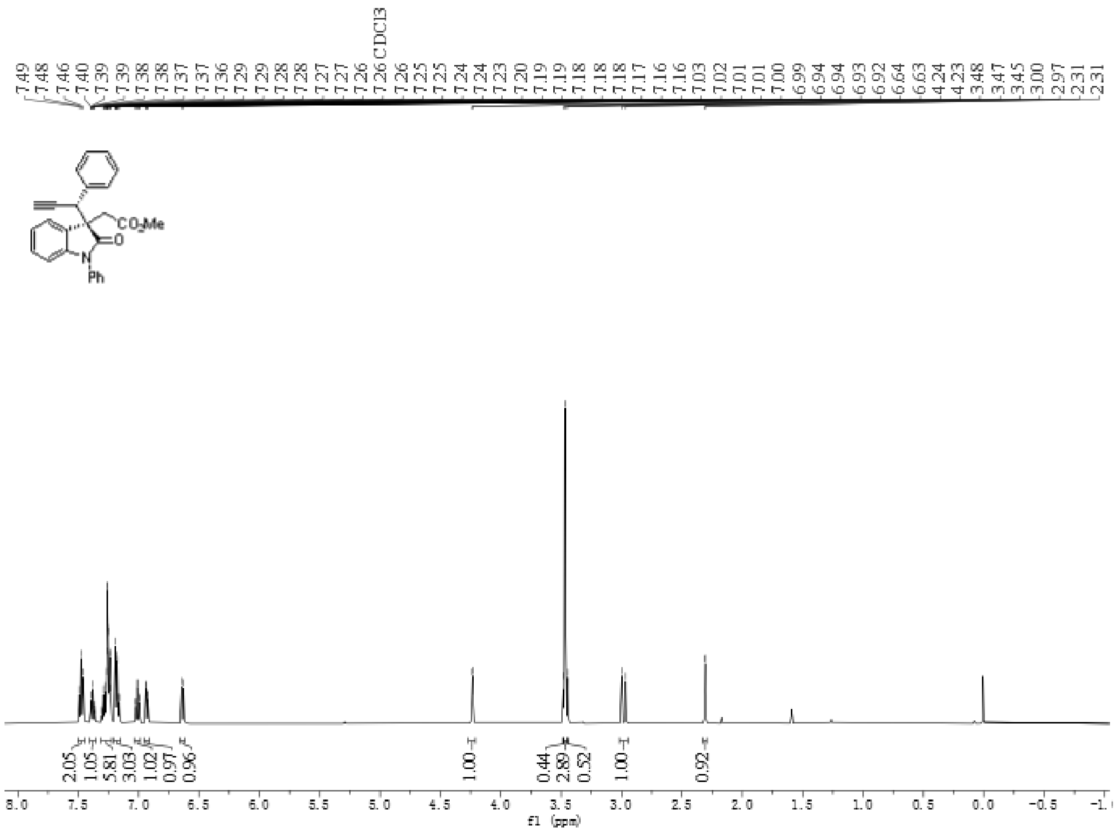

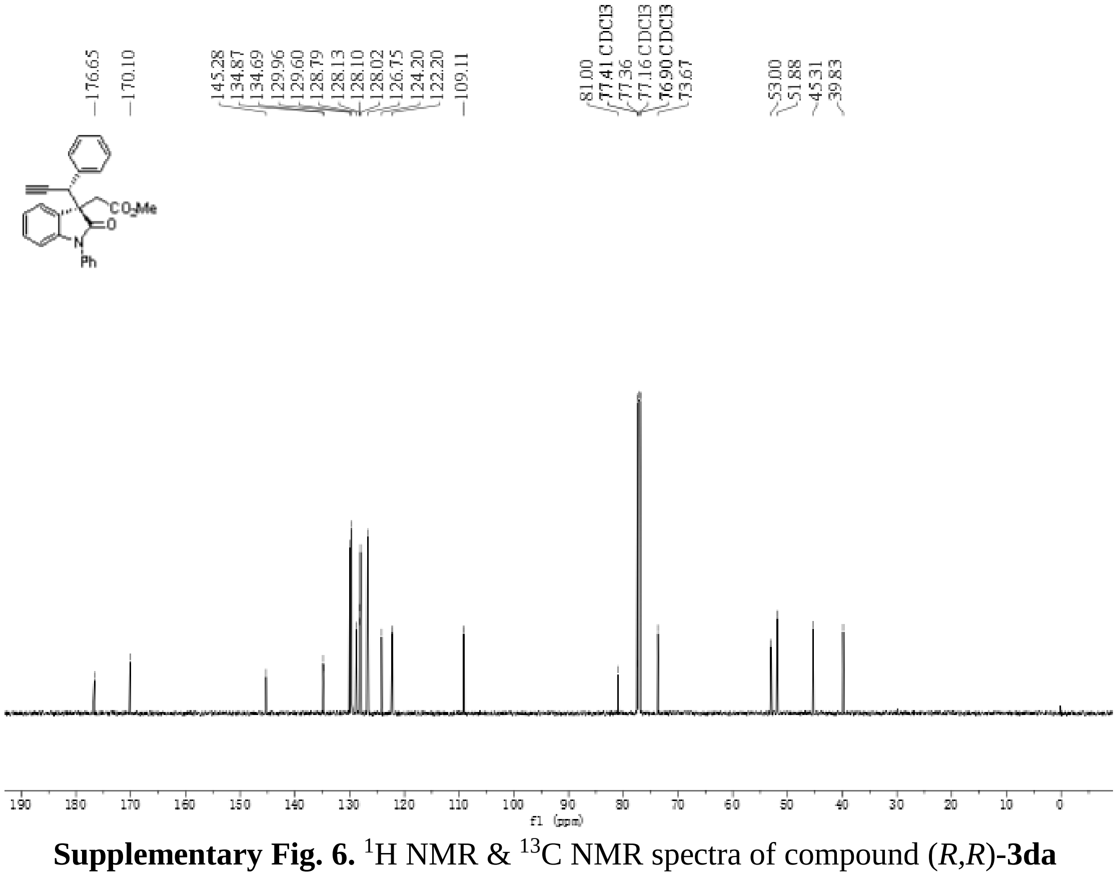

Supplementary Fig. 6. ^{1}H NMR & ^{13}C NMR spectra of compound (*R*,*R*)-**3da**

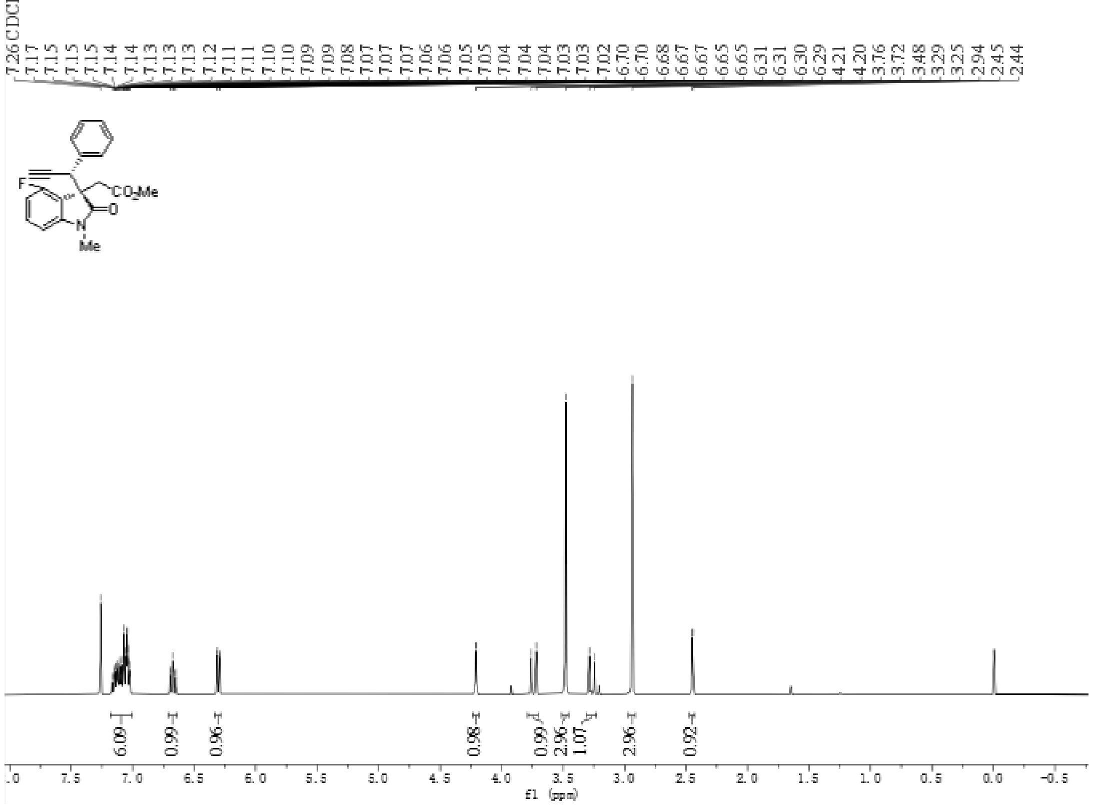
7.26 CDCl3
7.17
7.15
7.15
7.15
7.14
7.14
7.13
7.13
7.13
7.12
7.11
7.11
7.10
7.10
7.09
7.09
7.08
7.07
7.07
7.07
7.06
7.06
7.05
7.05
7.04
7.04
7.04
7.03
7.03
7.02
6.70
6.70
6.68
6.67
6.67
6.65
6.65
6.31
6.31
6.30
6.29
4.21
4.20
3.76
3.72
3.48
3.29
3.25
2.94
2.45
2.44
F
CO2Me
O
N
Me
6.09
0.99
0.96
0.98
0.99
2.96
1.07
2.96
0.92
f1 (ppm)

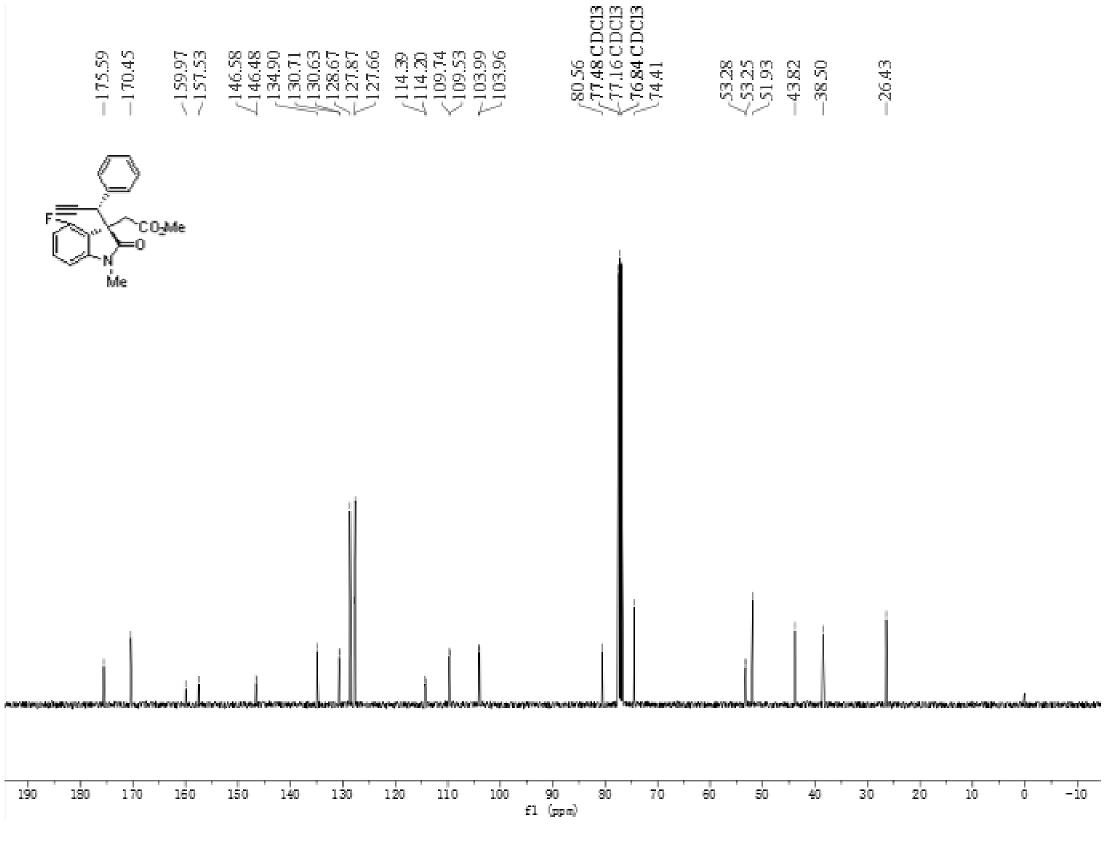
175.59
170.45
159.97
157.53
146.58
146.48
134.90
130.71
130.63
128.67
127.87
127.66
114.39
114.20
109.74
109.53
103.99
103.96
80.56
77.48 CDCl3
77.16 CDCl3
76.84 CDCl3
74.41
53.28
53.25
51.93
43.82
38.50
26.43
F
CO2Me
O
N
Me
f1 (ppm)

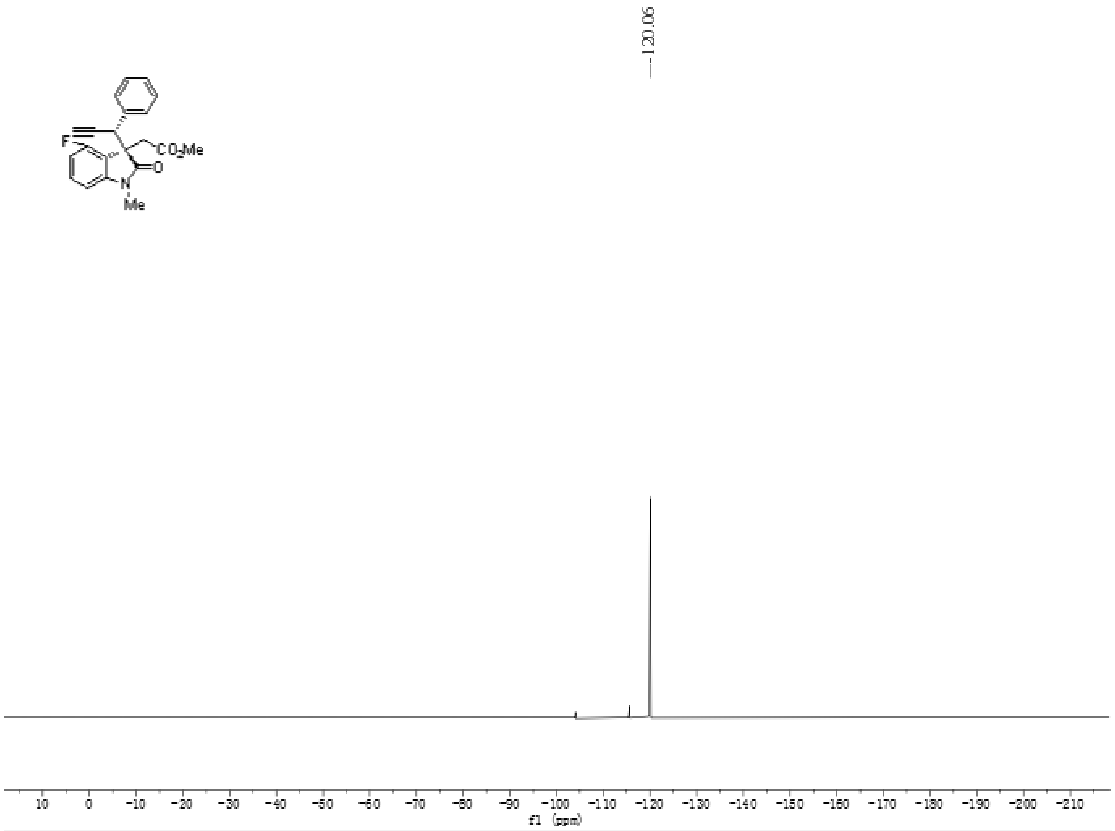

Supplementary Fig. 7. ^{1}H NMR ^{13}C NMR & ^{19}F NMR spectra of compound (*R*,*R*)-**3ea**

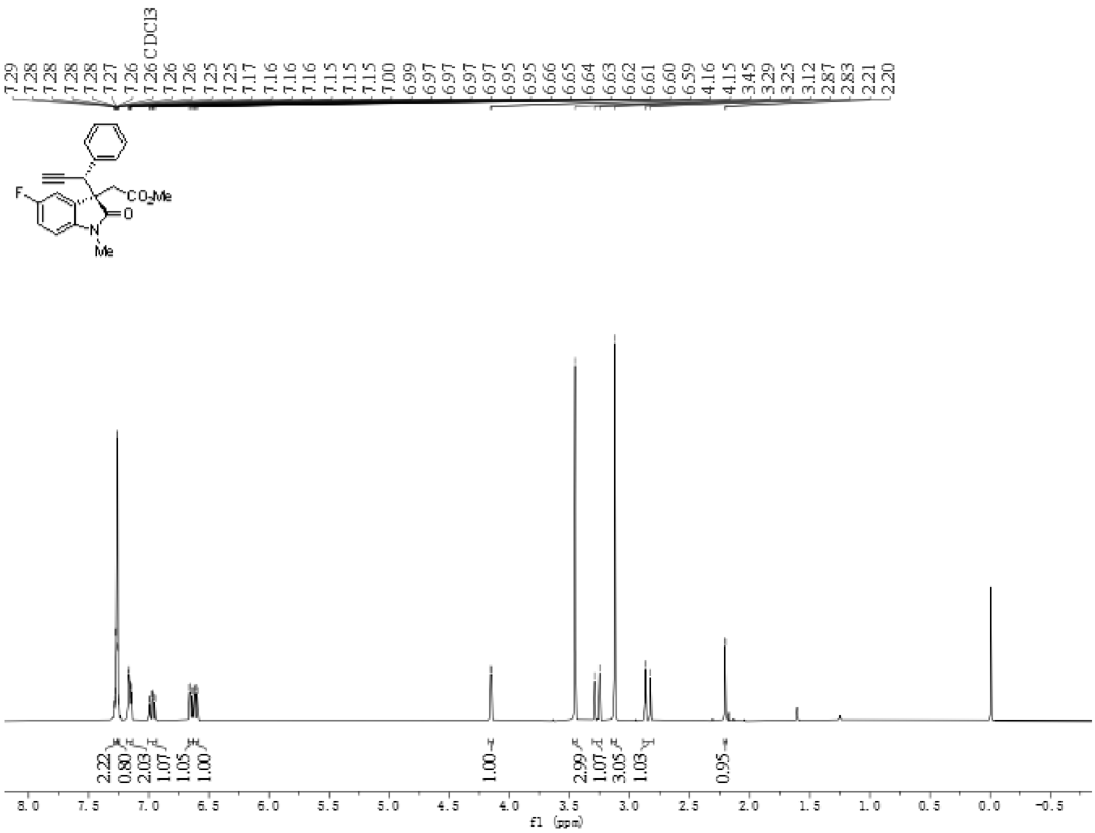
7.29 7.28 7.28 7.28 7.28 7.27 7.26 CDCl3 7.26 7.26 7.25 7.25 7.17 7.16 7.16 7.16 7.15 7.15 7.15 7.00 6.99 6.97 6.97 6.97 6.97 6.95 6.95 6.66 6.65 6.64 6.63 6.62 6.61 6.60 6.59 4.16 4.15 3.45 3.29 3.25 3.12 2.87 2.83 2.21 2.20
F
CO2Me
O
N
Me
2.22 0.80 2.03 1.07 1.05 1.00
1.00
2.99 1.07 3.05 1.03
0.95
8.0 7.5 7.0 6.5 6.0 5.5 5.0 4.5 4.0 3.5 3.0 2.5 2.0 1.5 1.0 0.5 0.0 -0.5
f1 (ppm)

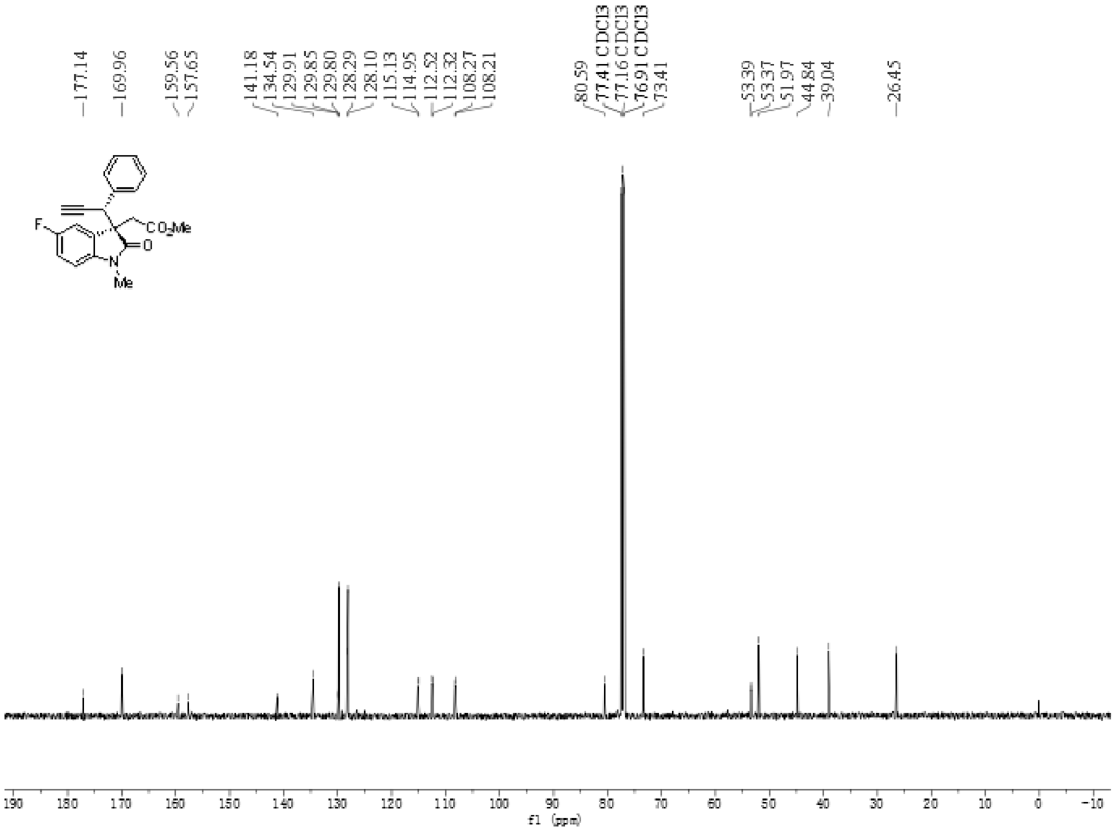
177.14
169.96
159.56 157.65
141.18 134.54 129.91 129.85 129.80 128.29 128.10 115.13 114.95 112.52 112.32 108.27 108.21
80.59 77.41 CDCl3 77.16 CDCl3 76.91 CDCl3 73.41
53.39 53.37 51.97 44.84 39.04
26.45
F
CO2Me
O
N
Me
190 180 170 160 150 140 130 120 110 100 90 80 70 60 50 40 30 20 10 0 -10
f1 (ppm)

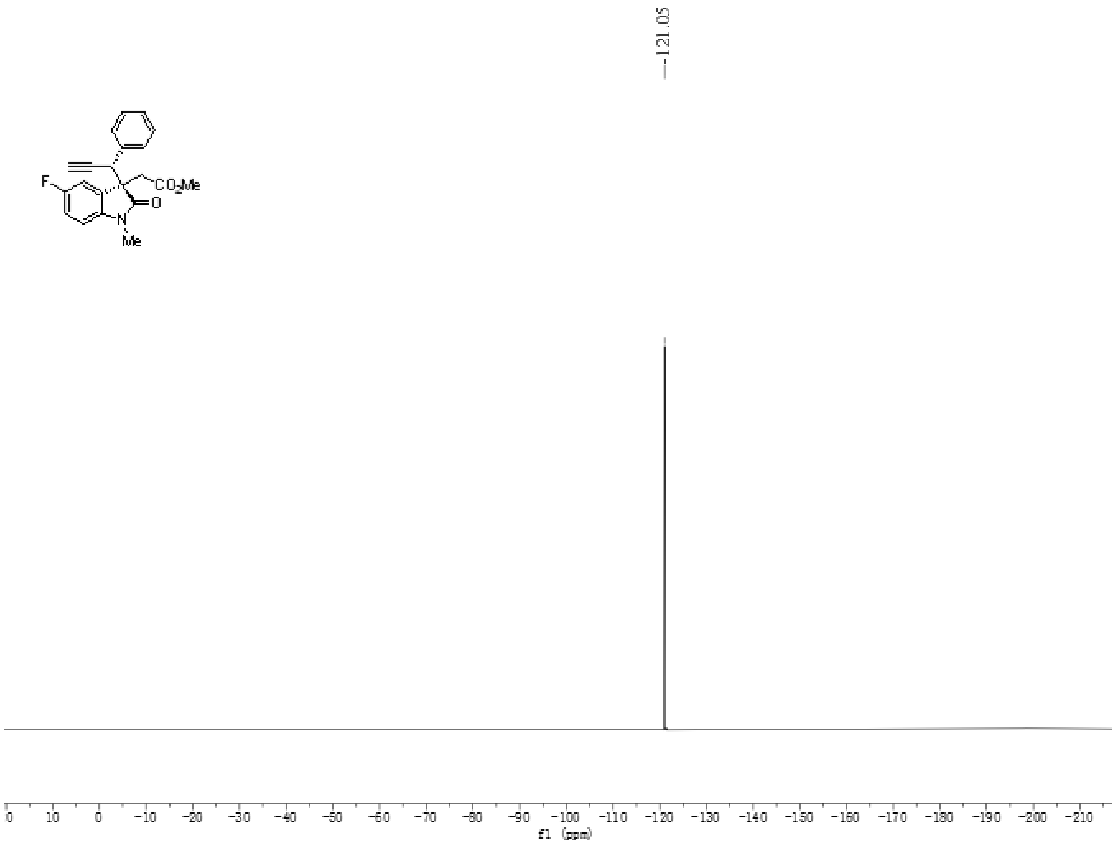

Supplementary Fig. 8. ^{1}H NMR ^{13}C NMR & ^{19}F NMR spectra of compound (*R*,*R*)-**3fa**

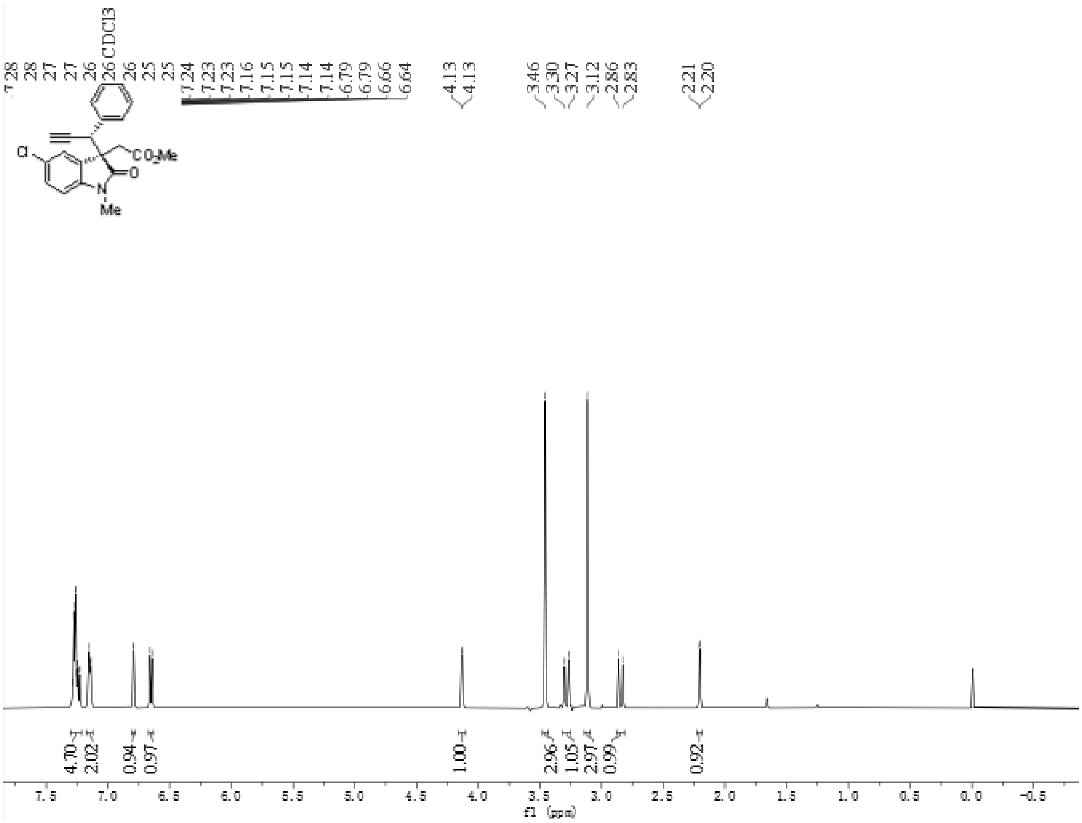

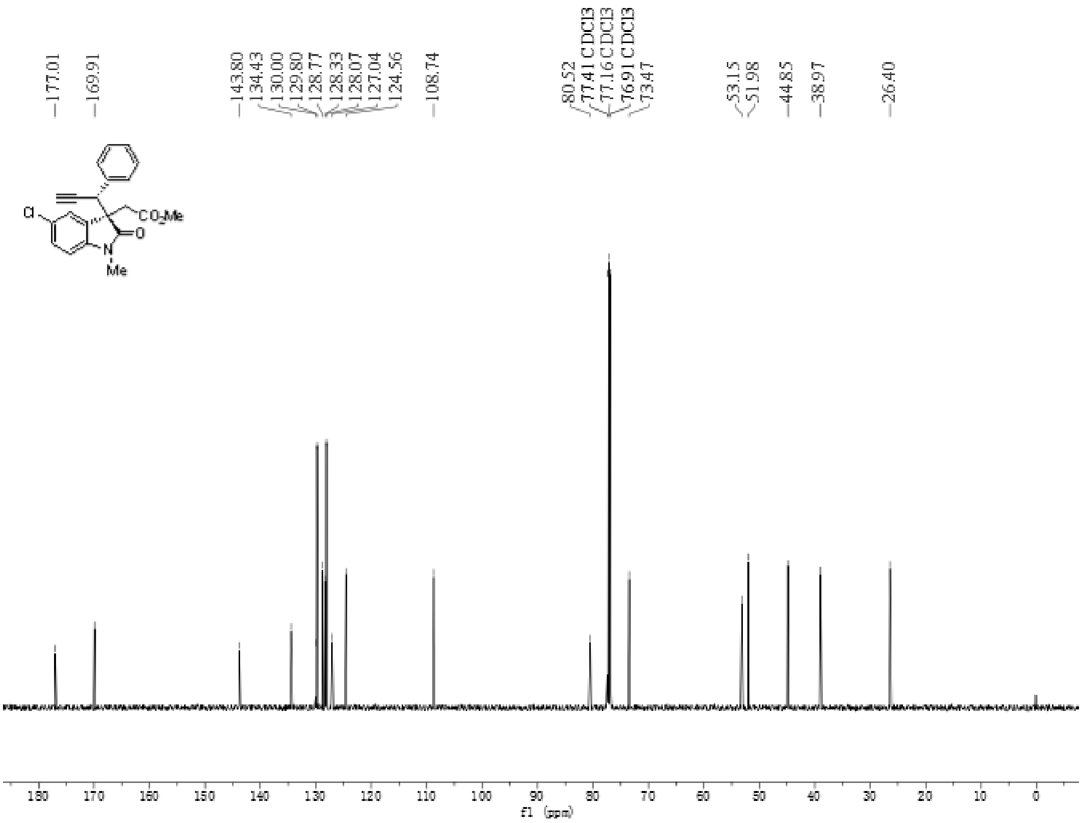

Supplementary Fig. 9. ^{1}H NMR & ^{13}C NMR spectra of compound (*R*,*R*)-**3ga**

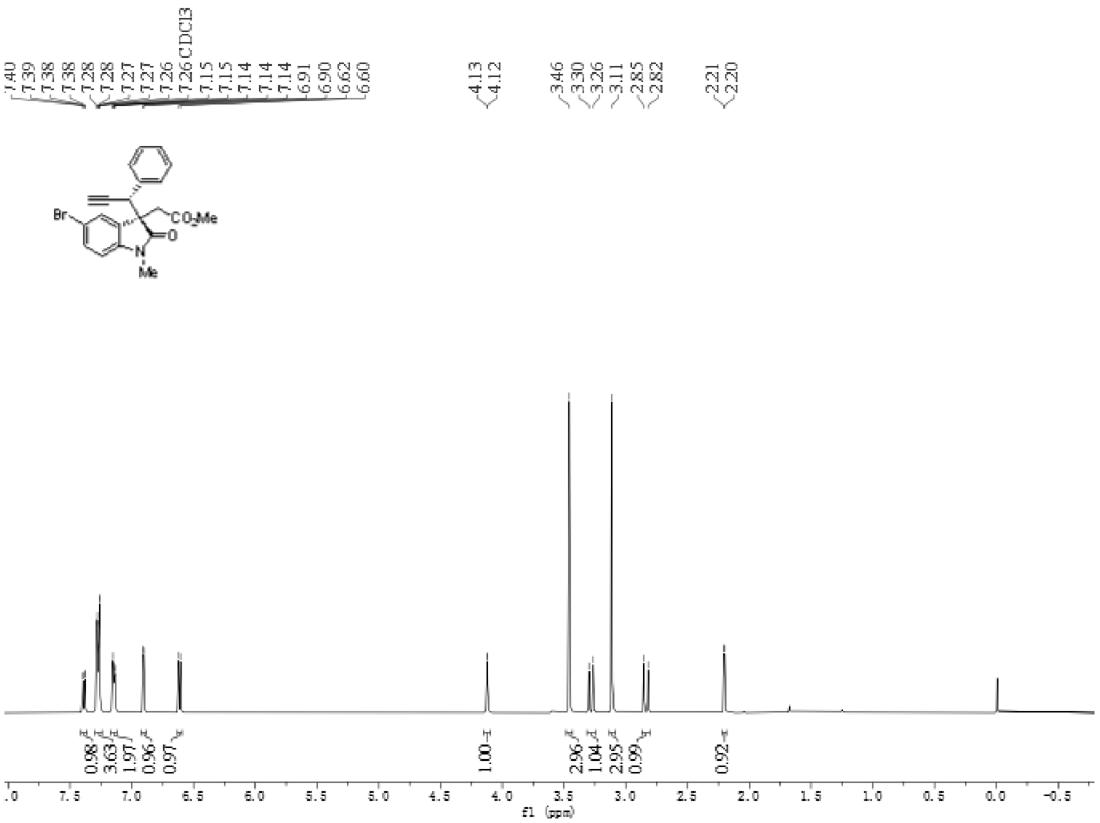

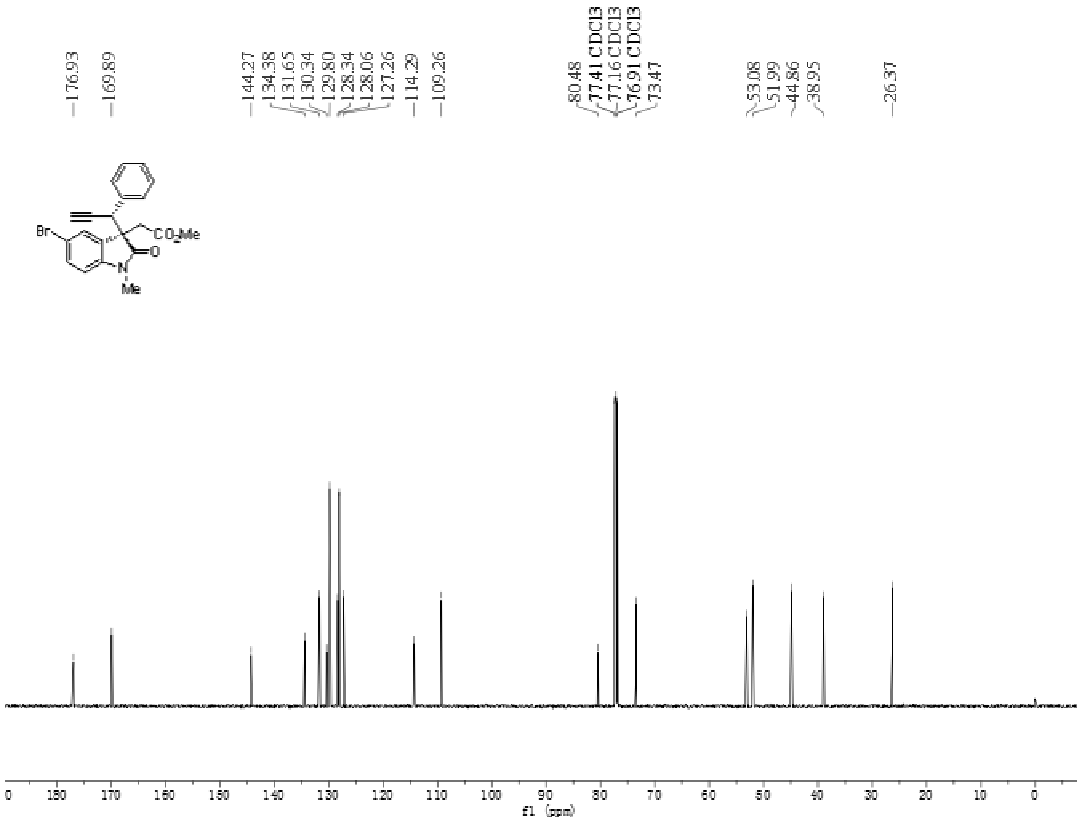

Supplementary Fig. 10. ^{1}H NMR & ^{13}C NMR spectra of compound (*R*,*R*)-**3ha**

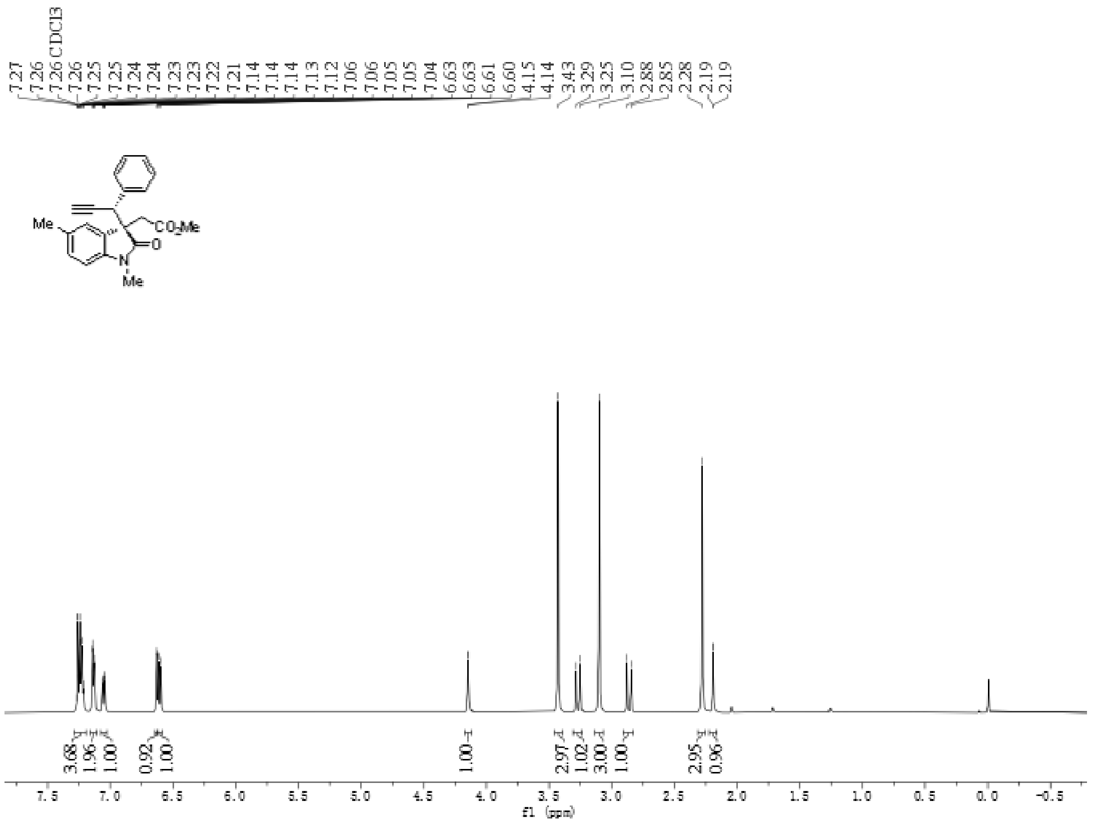

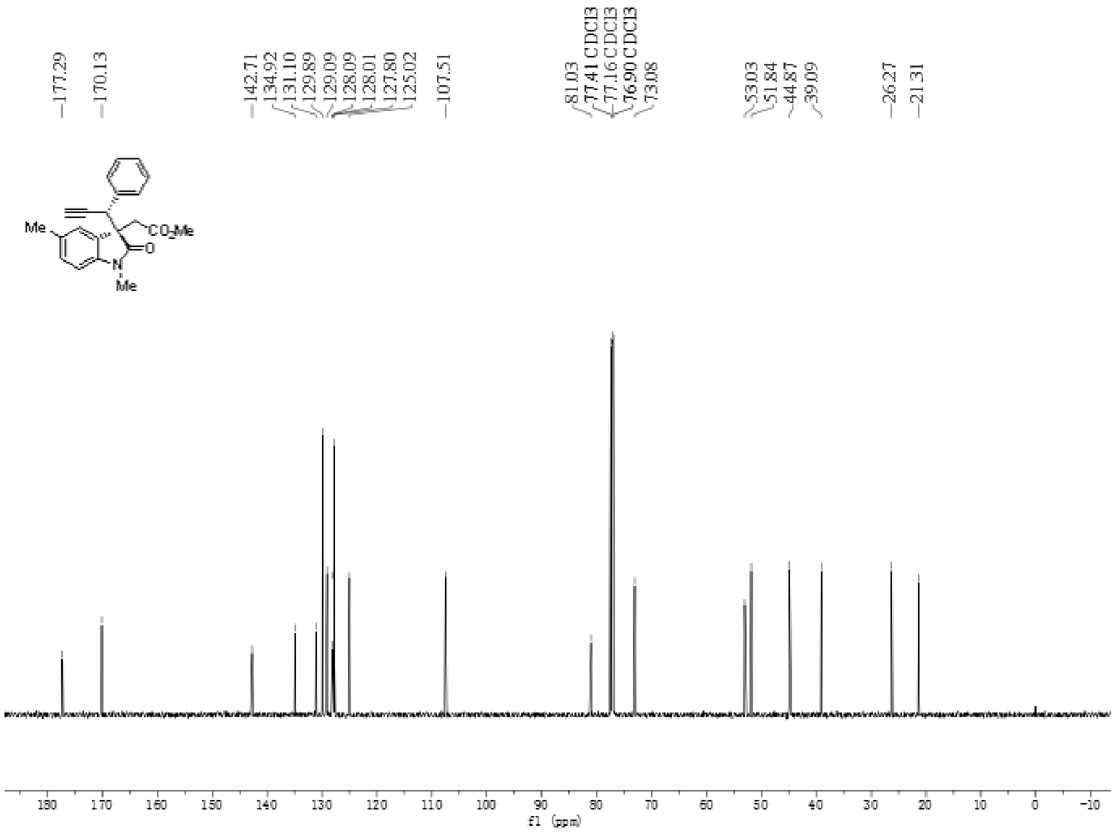

Supplementary Fig. 11. ^{1}H NMR & ^{13}C NMR spectra of compound (*R*,*R*)-**3ia**

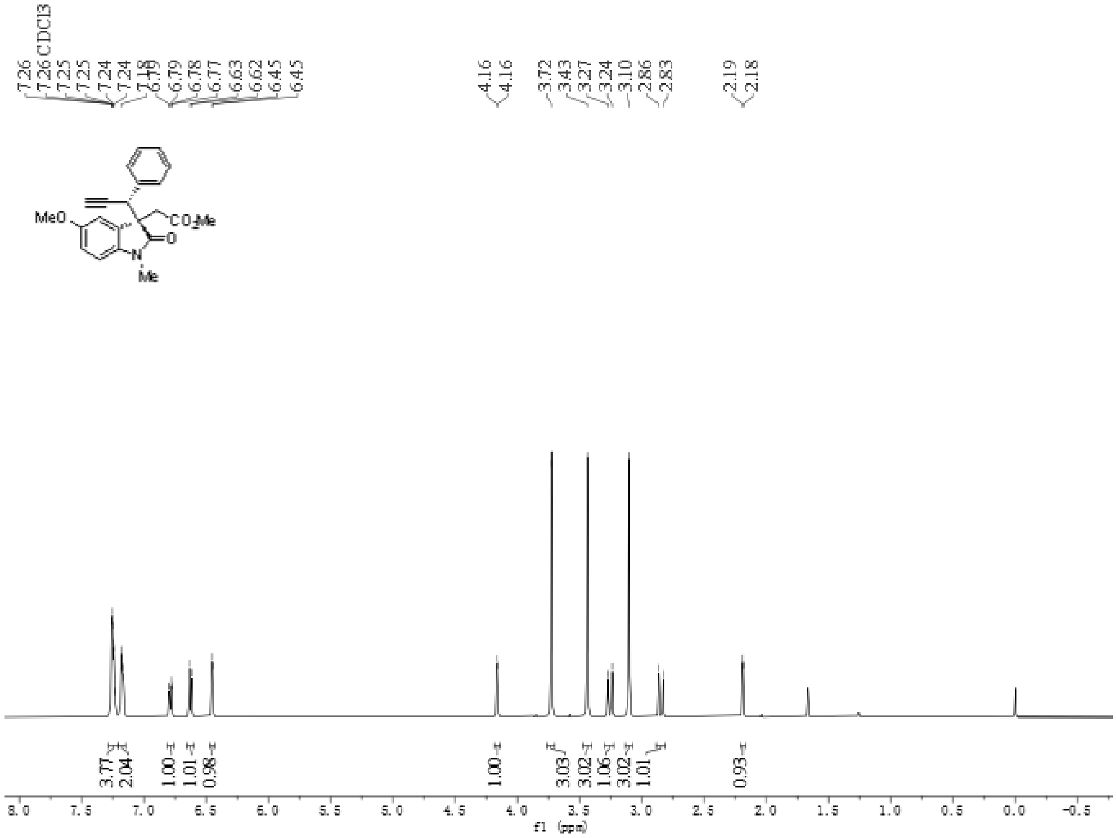

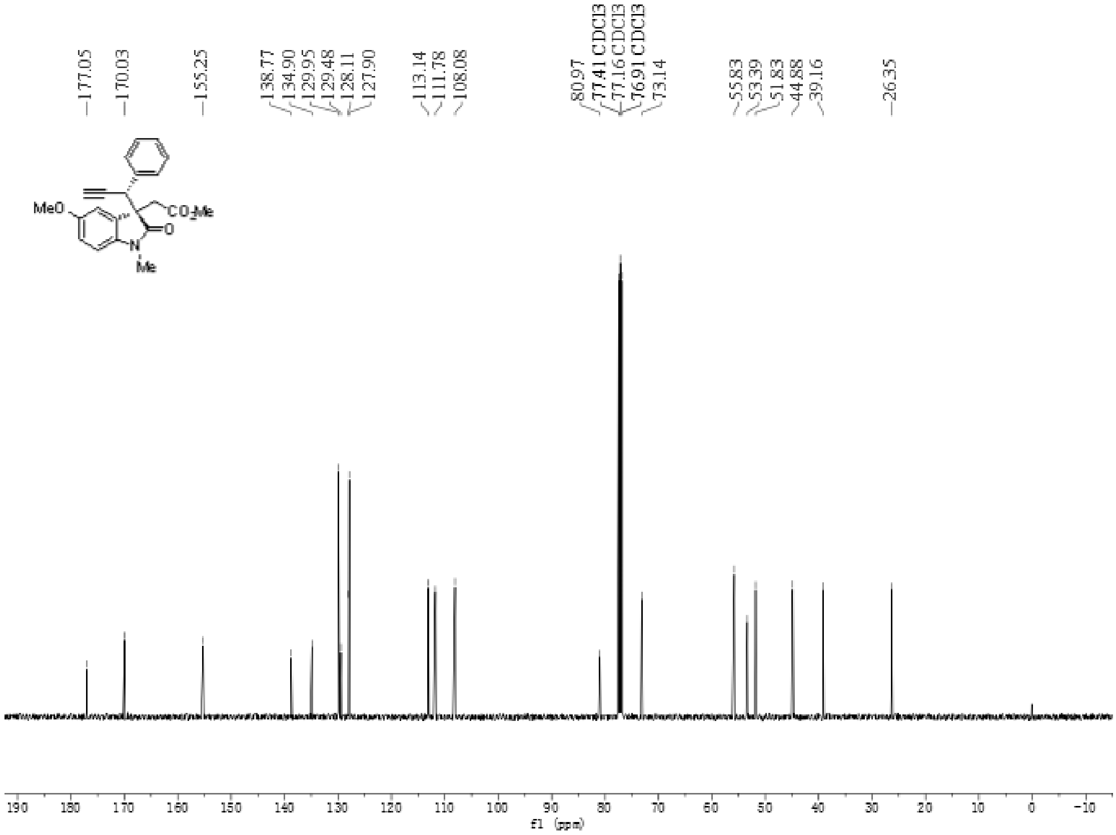

Supplementary Fig. 12. ^{1}H NMR & ^{13}C NMR spectra of compound (*R*,*R*)-**3ja**

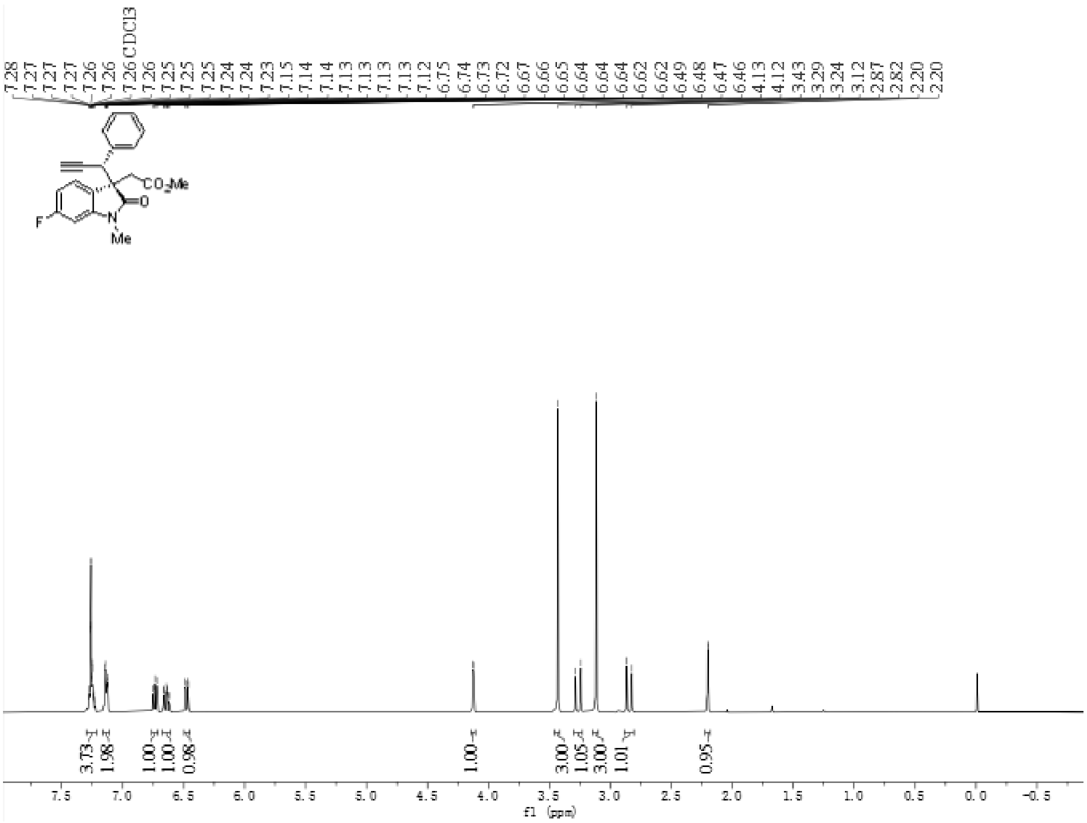

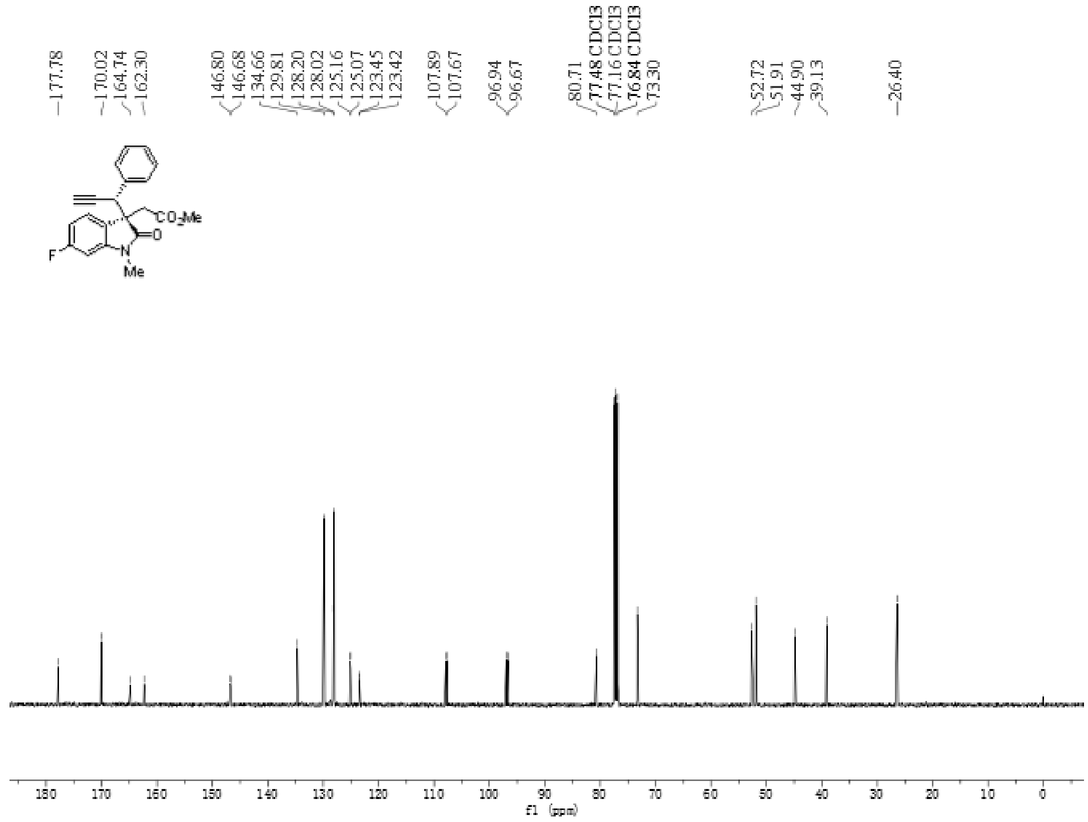

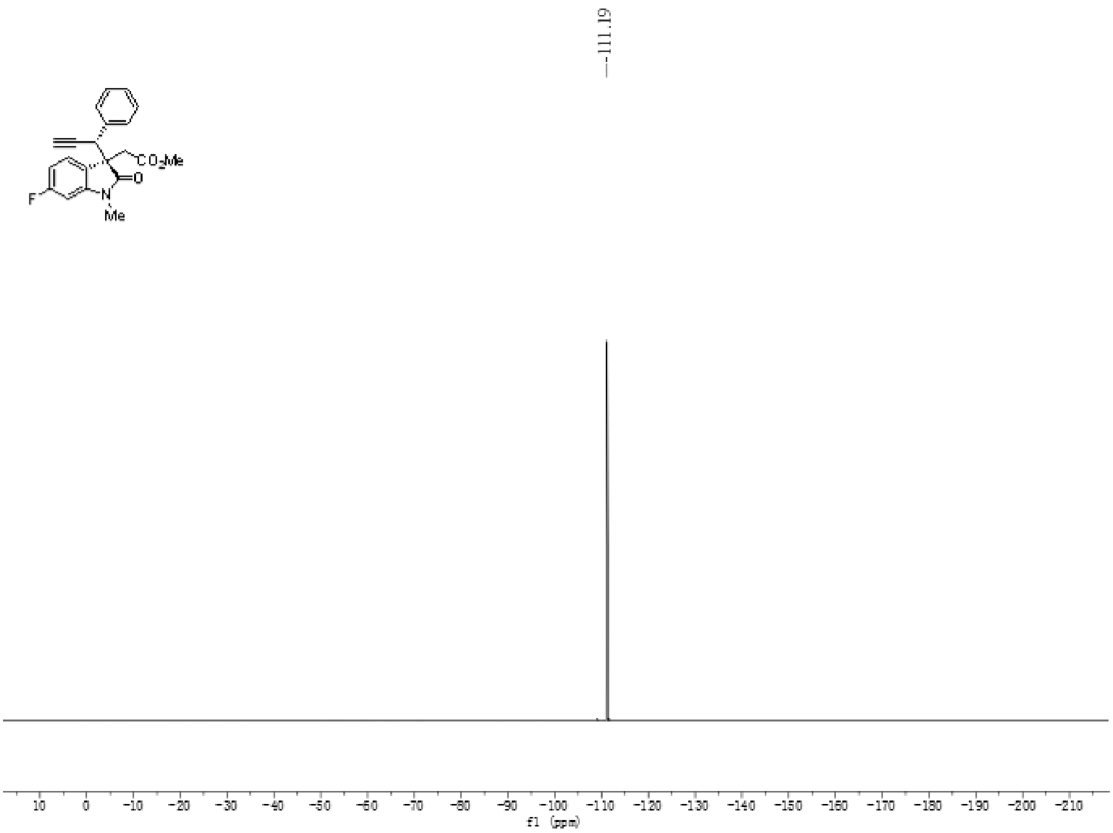

Supplementary Fig. 13. ^{1}H NMR ^{13}C NMR & ^{19}F NMR spectra of compound (*R*,*R*)-**3ka**

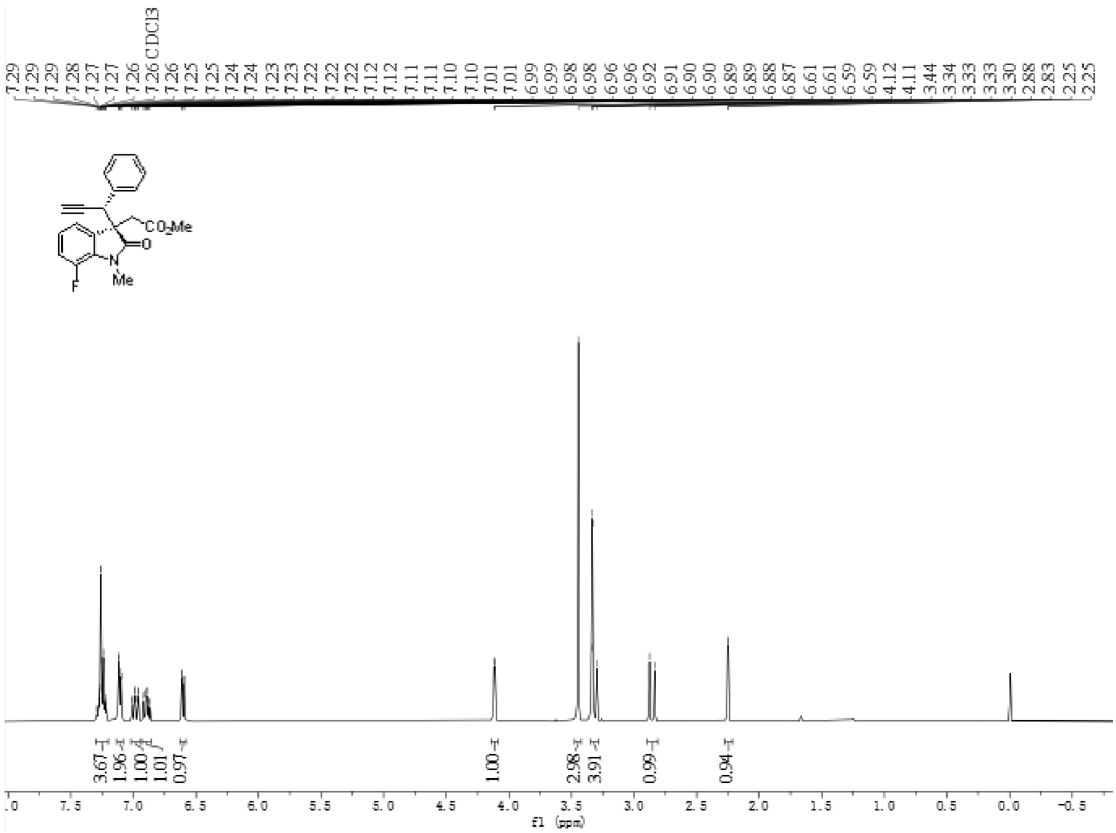

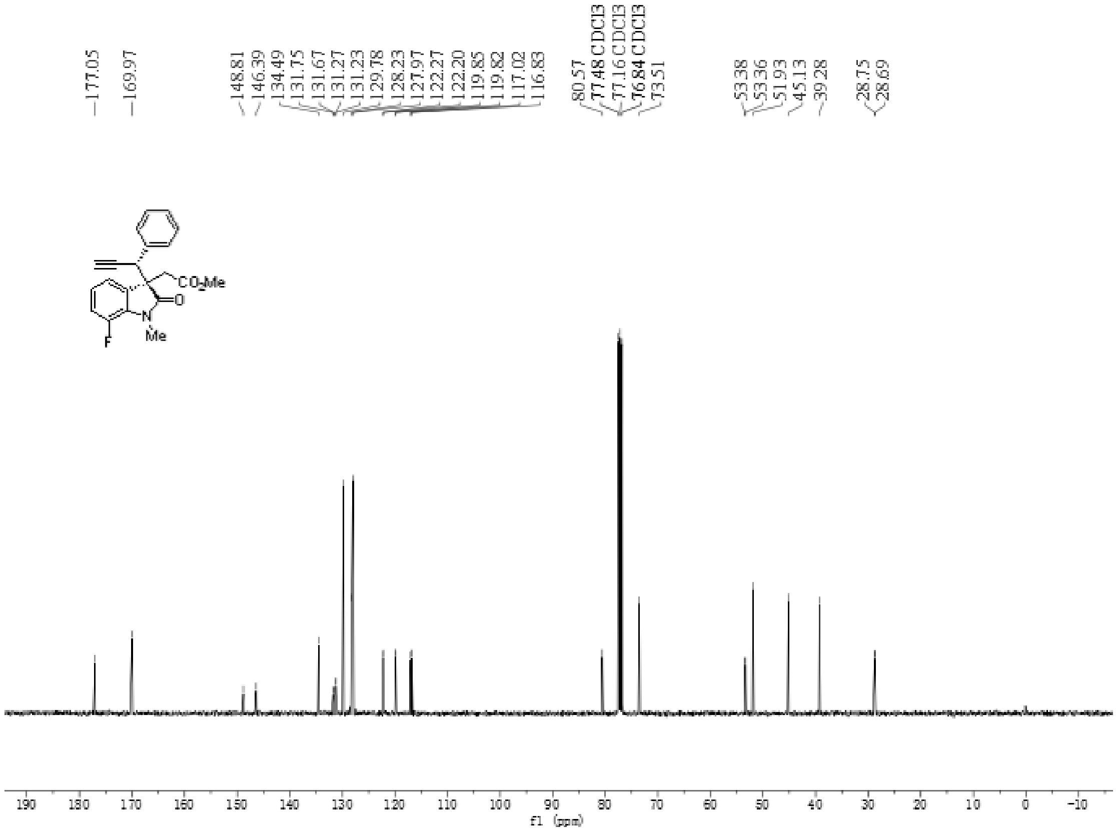

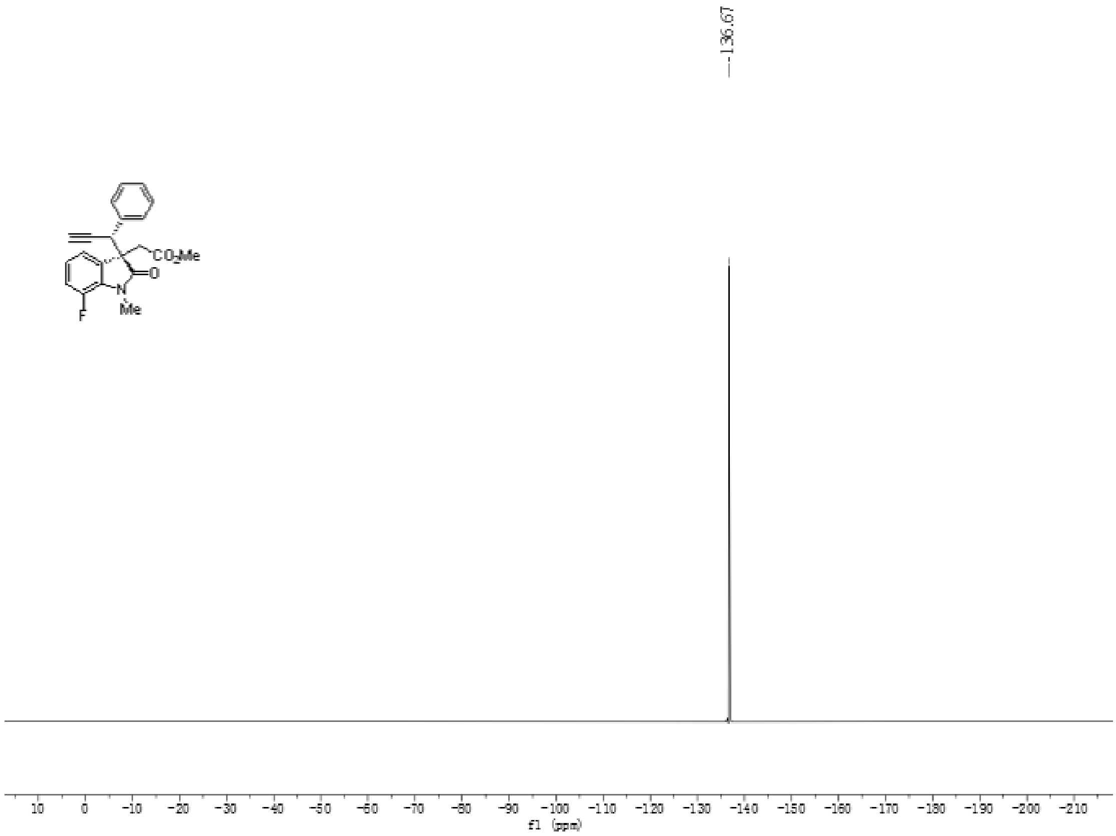

Supplementary Fig. 14. ^{1}H NMR ^{13}C NMR & ^{19}F NMR spectra of compound (*R*,*R*)-**3la**

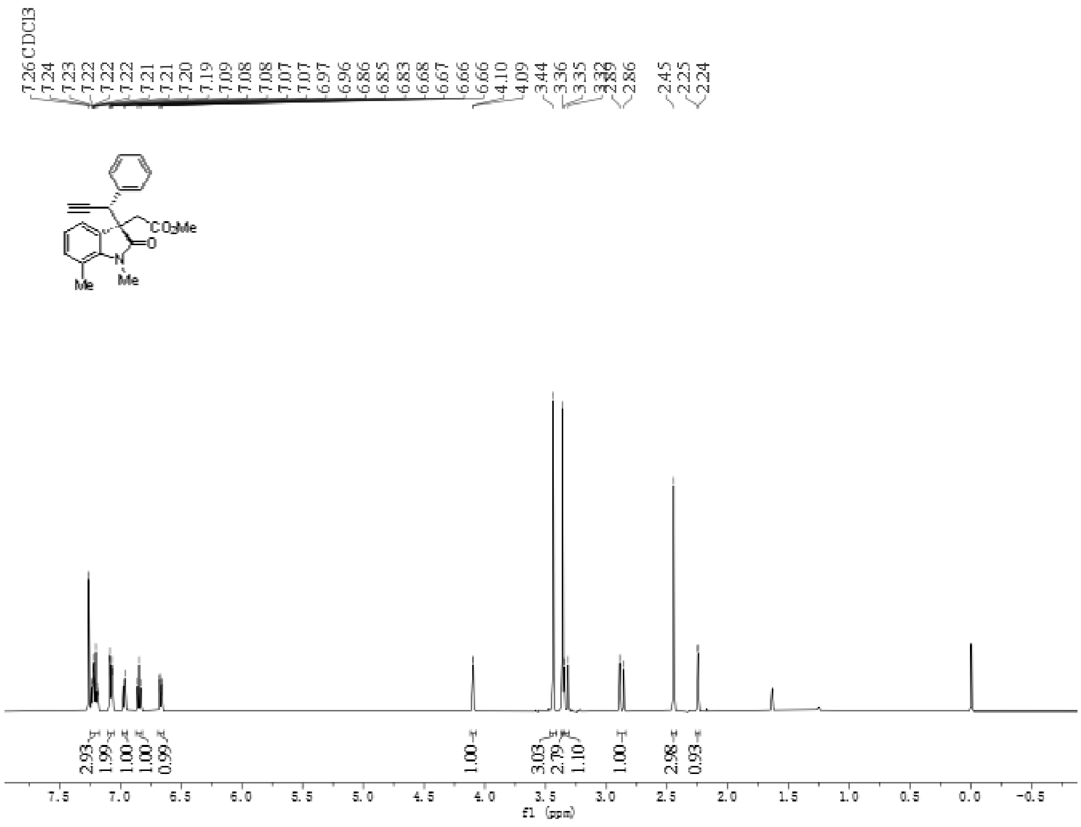

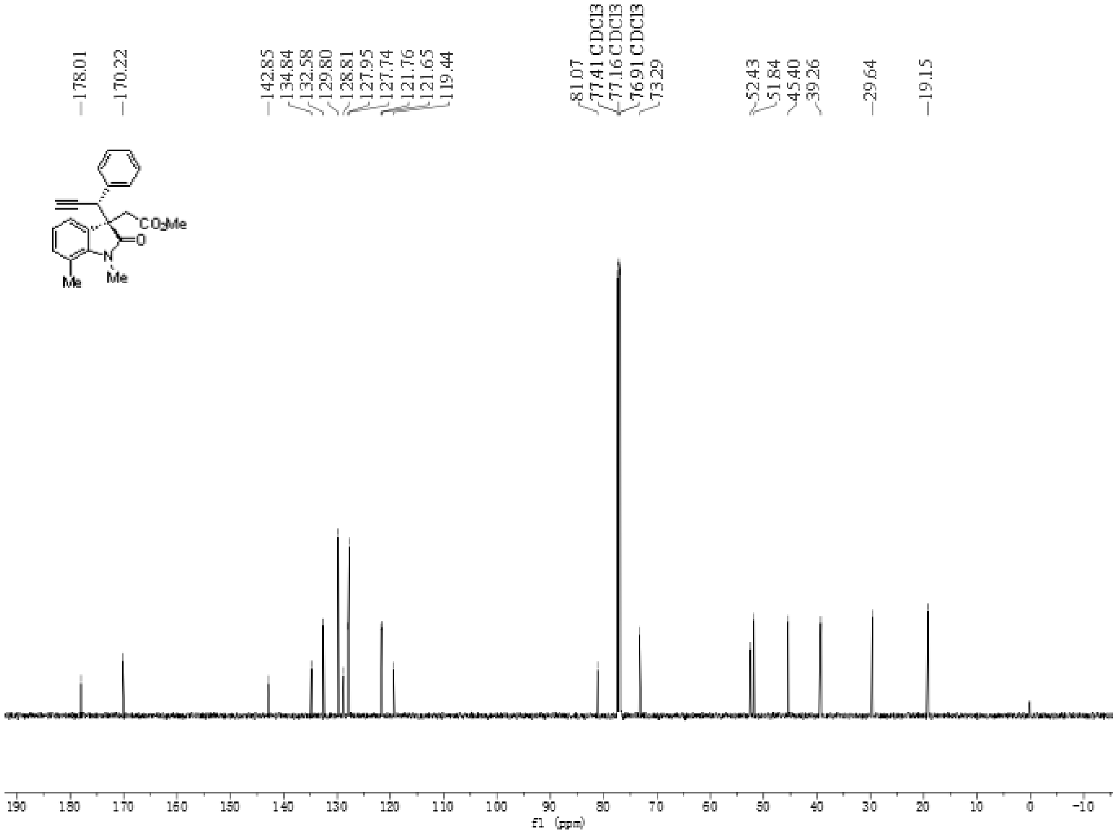

Supplementary Fig. 15. ^{1}H NMR & ^{13}C NMR spectra of compound (*R,R*)-**3ma**

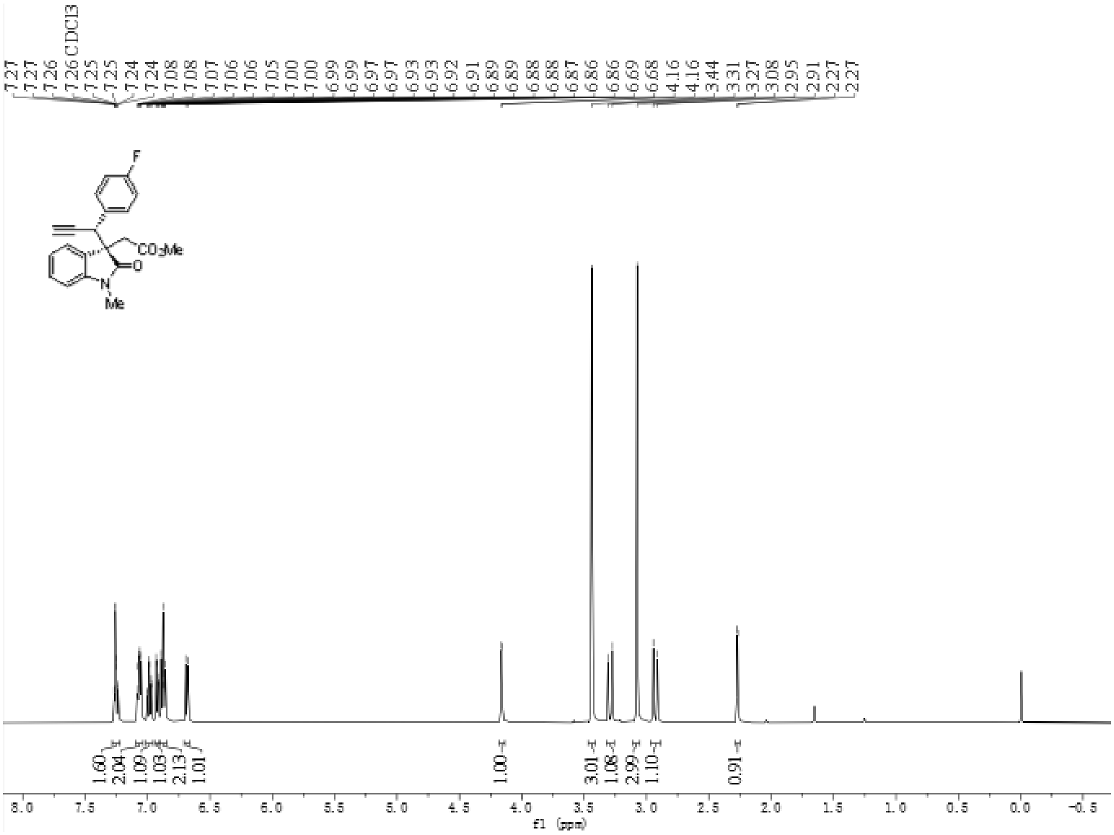

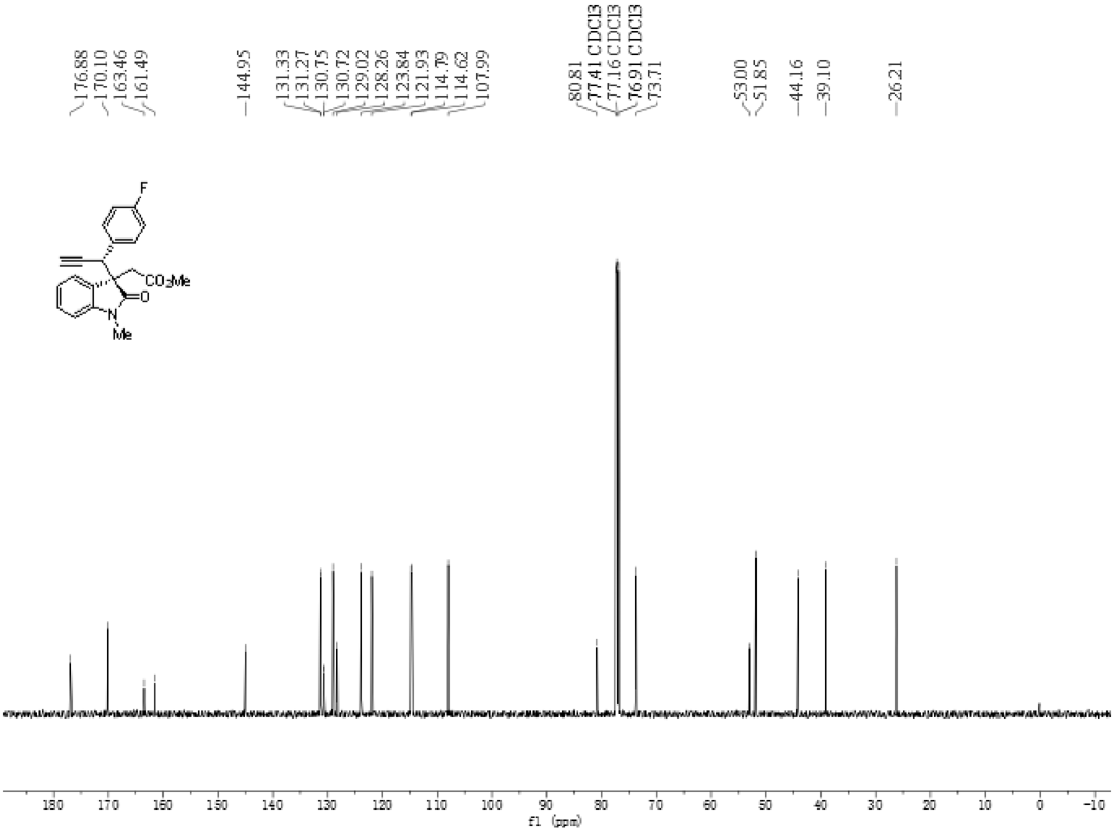

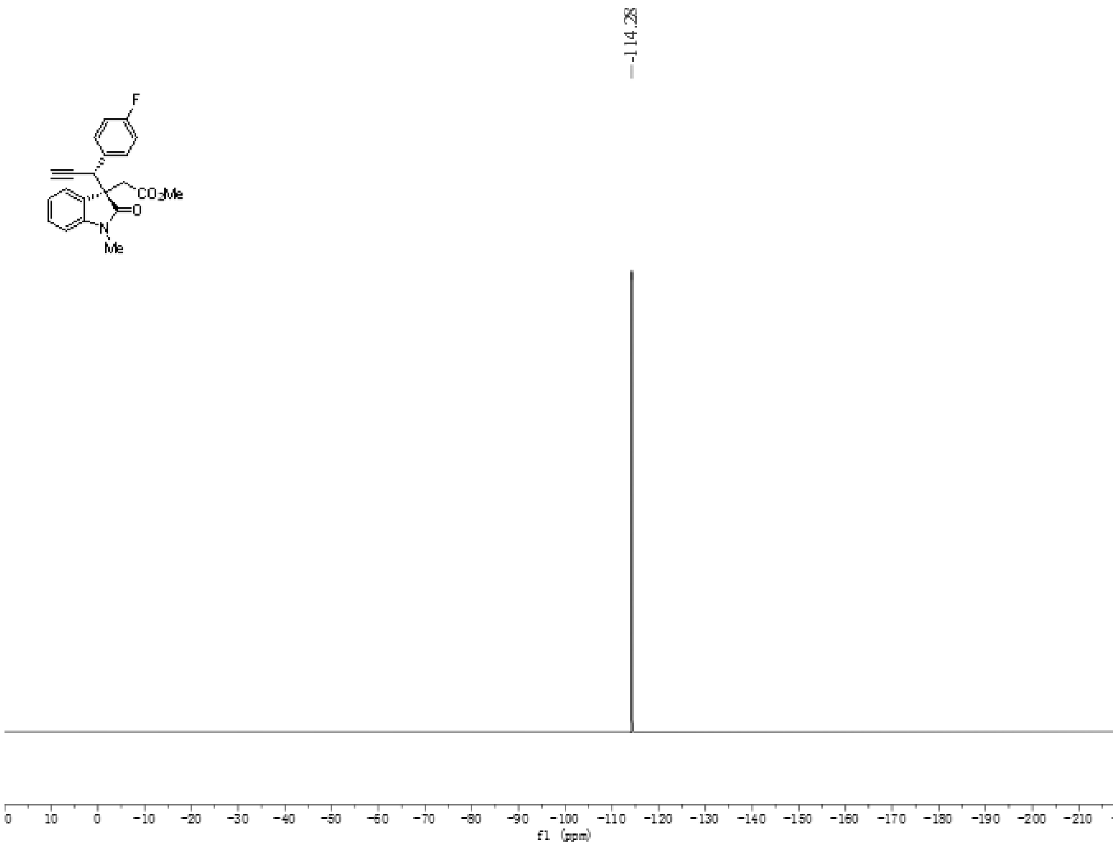

Supplementary Fig. 16. ^{1}H NMR ^{13}C NMR & ^{19}F NMR spectra of compound (*R*,*R*)-**3ab**

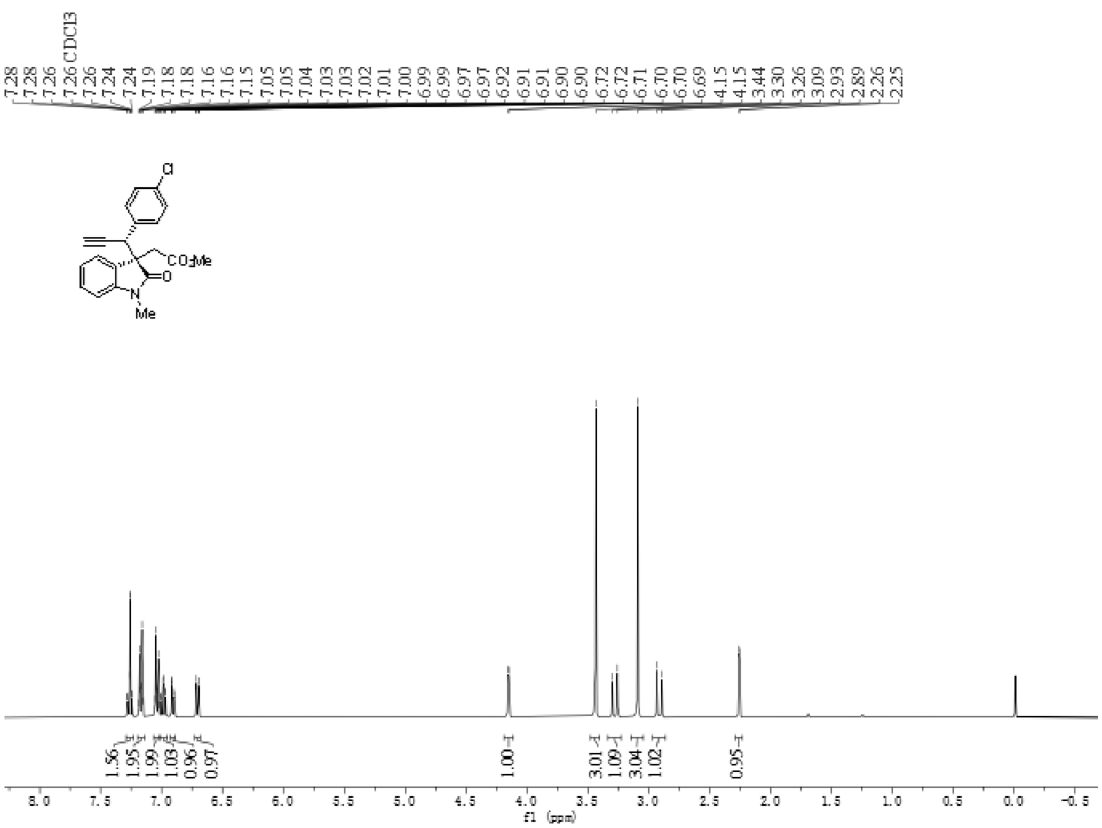

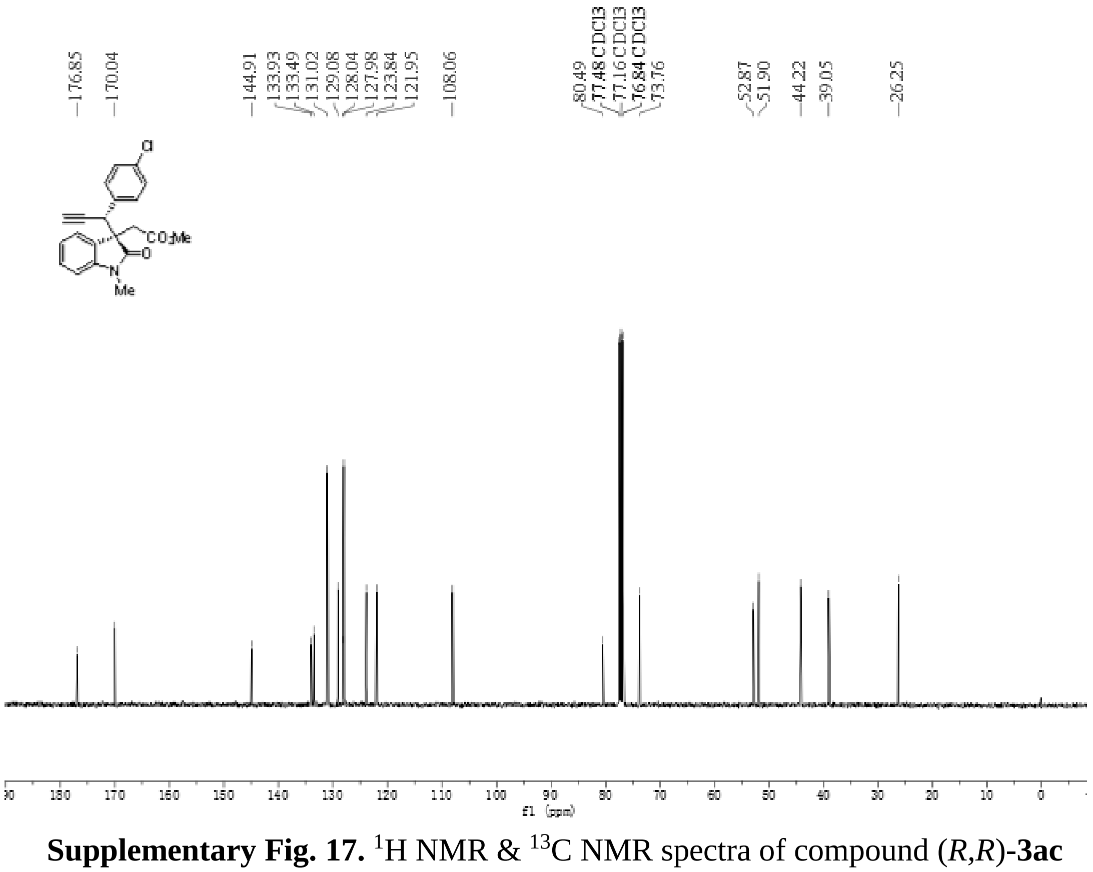

Supplementary Fig. 17. ^{1}H NMR & ^{13}C NMR spectra of compound (*R*,*R*)-**3ac**

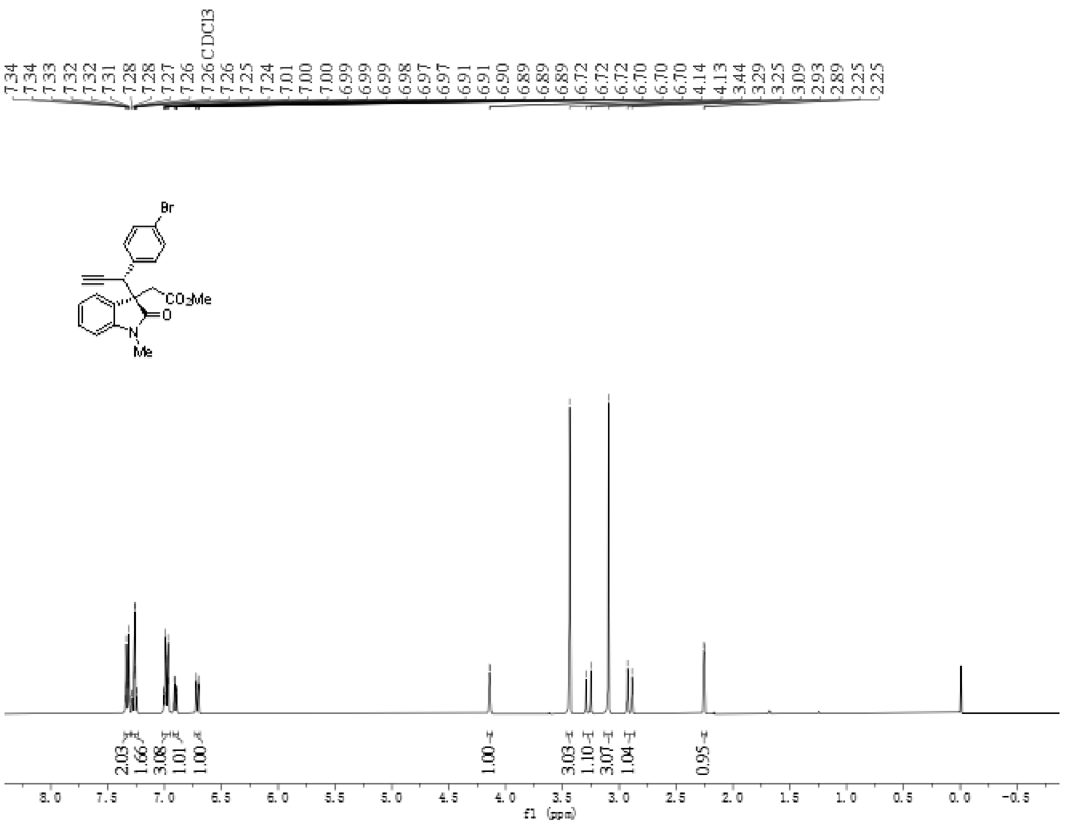

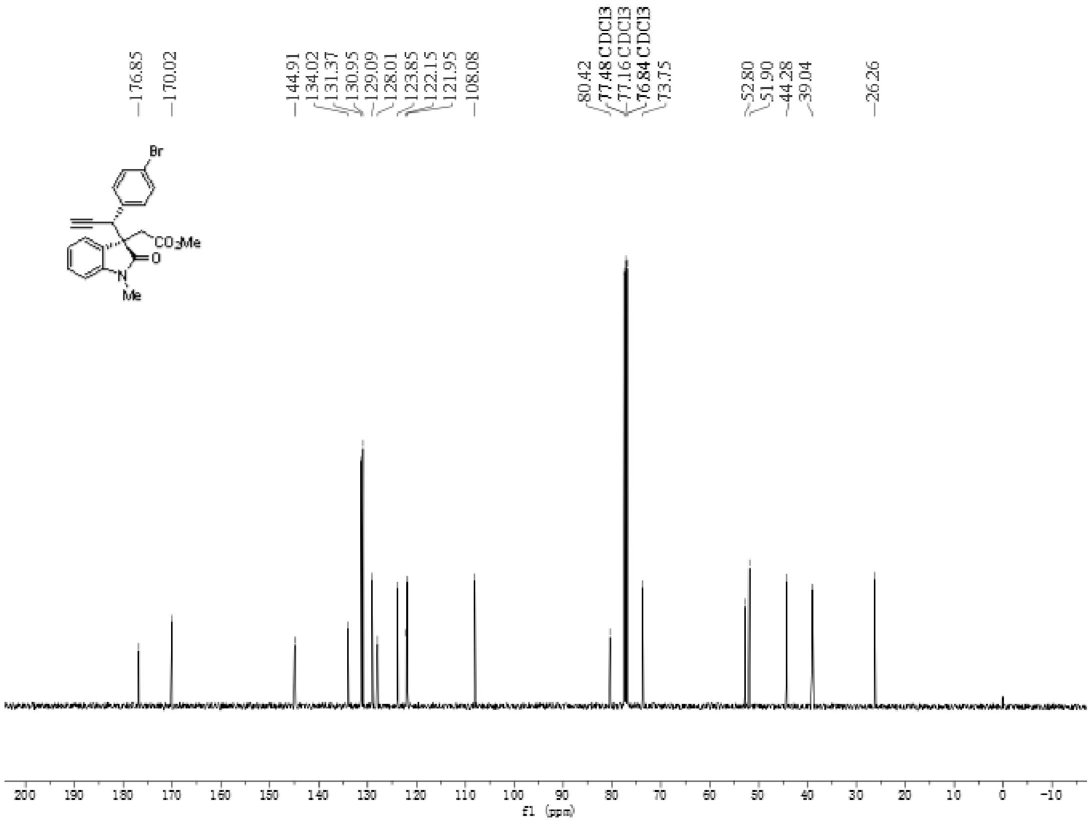

Supplementary Fig. 18. ^{1}H NMR & ^{13}C NMR spectra of compound (*R*,*R*)-**3ad**

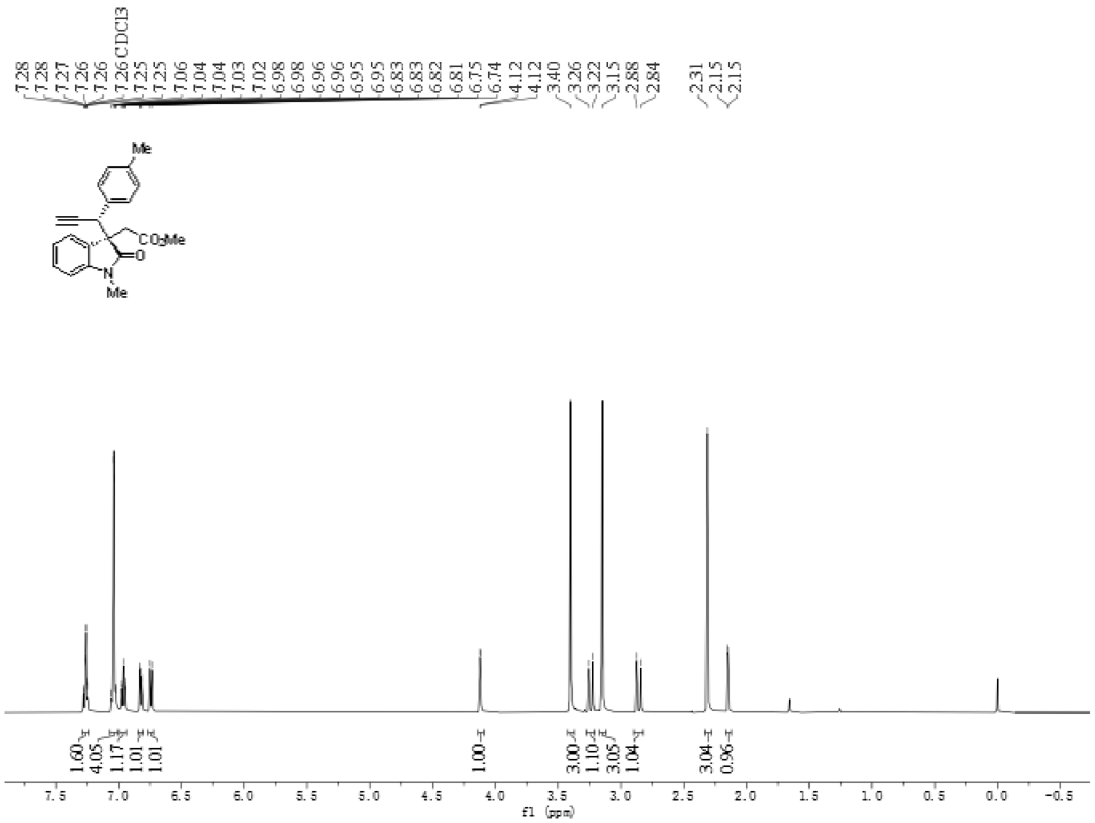

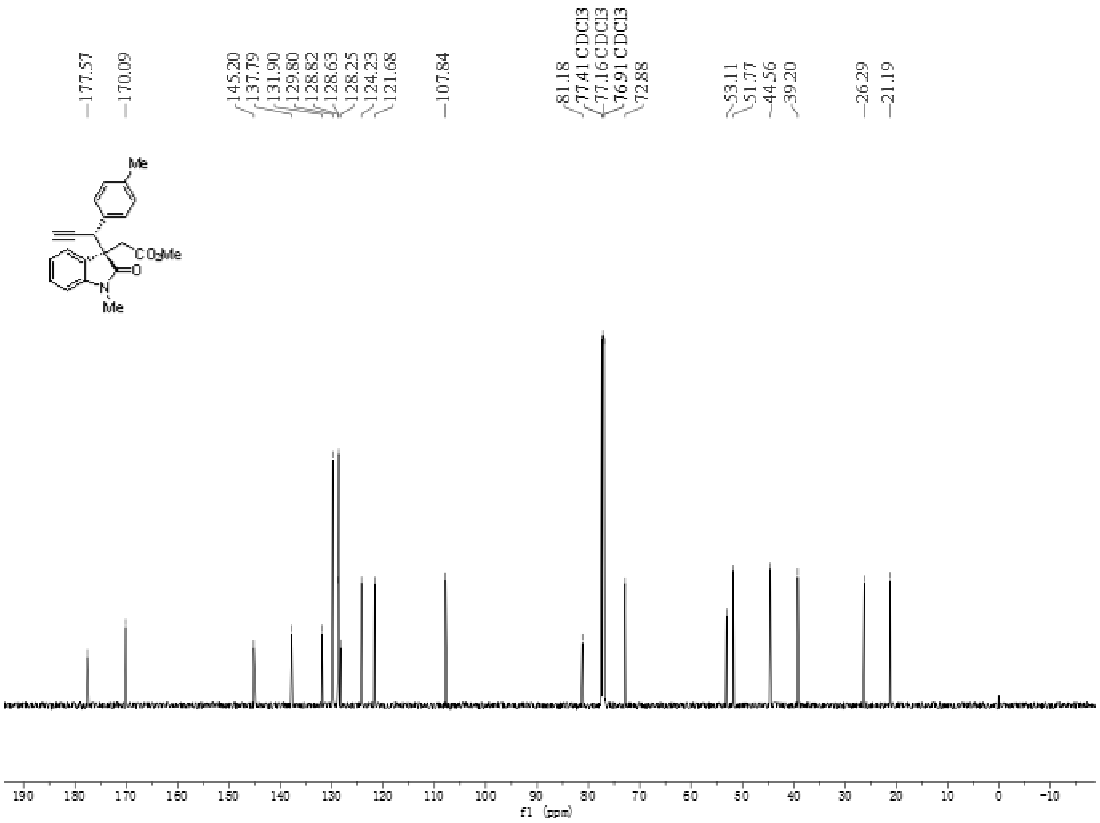

Supplementary Fig. 19. ^{1}H NMR & ^{13}C NMR spectra of compound (*R*,*R*)-**3ae**

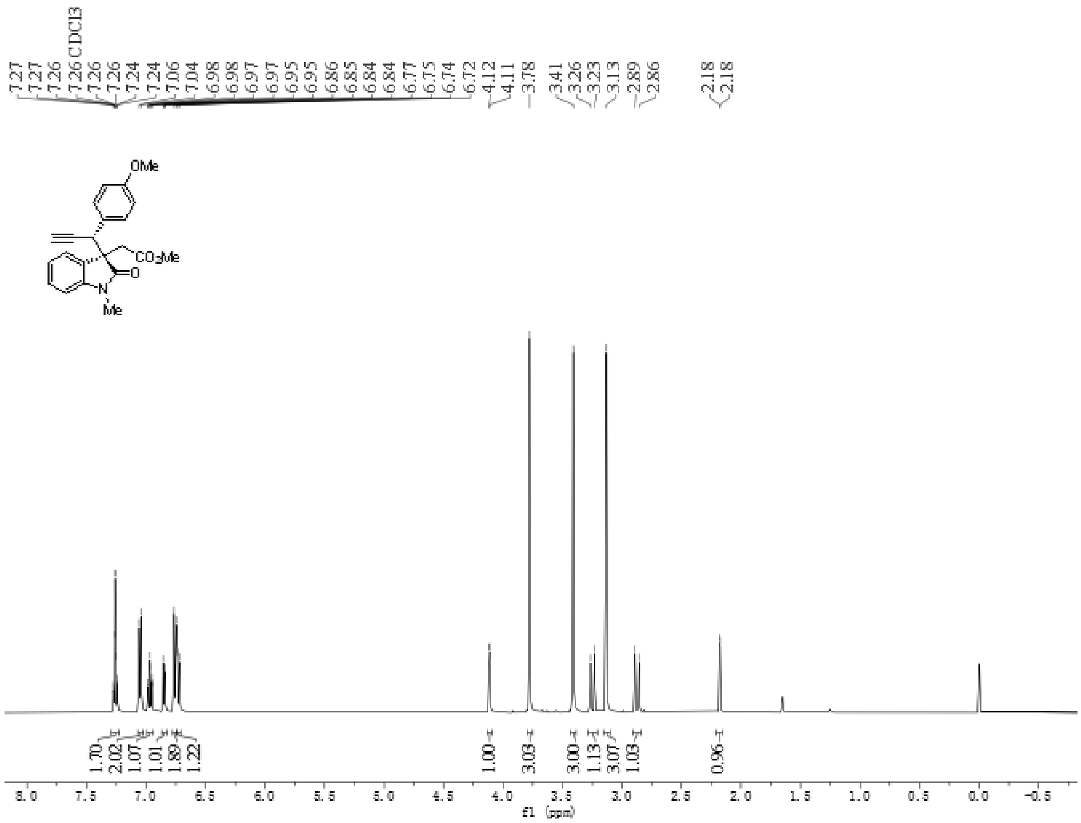

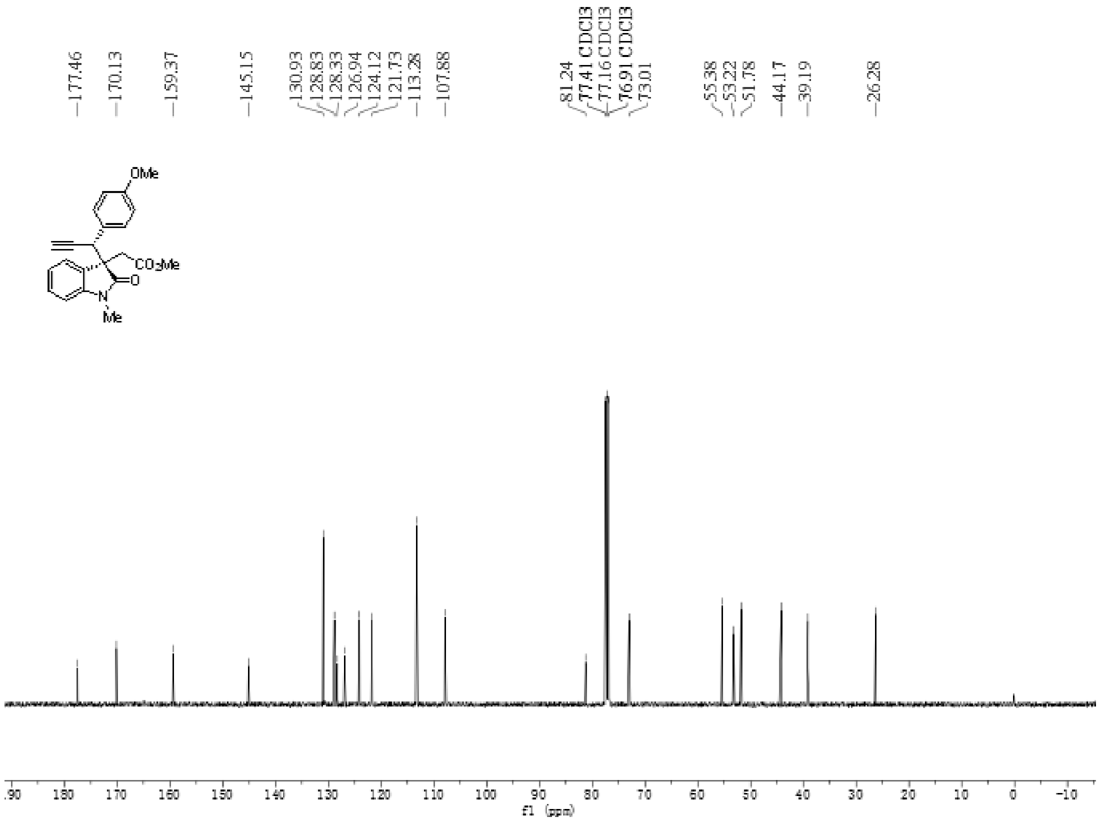

Supplementary Fig. 20. ^{1}H NMR & ^{13}C NMR spectra of compound (*R*,*R*)-**3af**

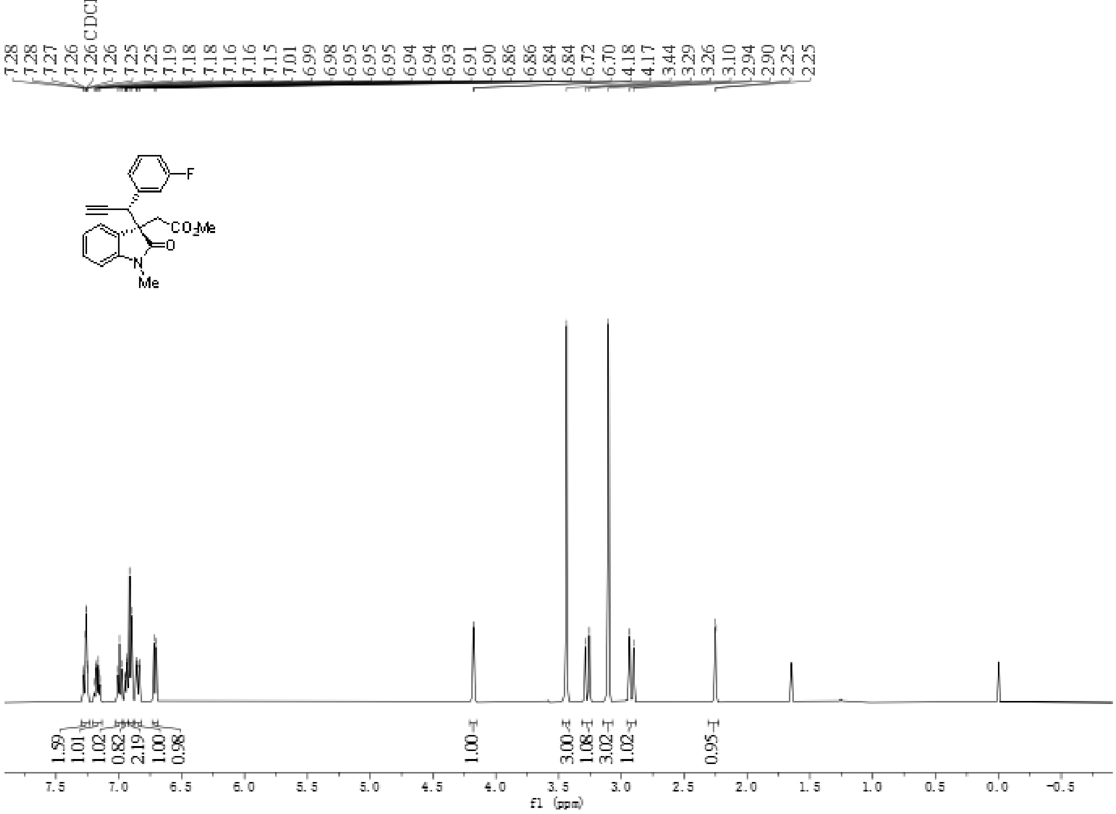
7.28 7.28 7.27 7.26 7.26 CDCl3 7.26 7.25 7.25 7.19 7.18 7.18 7.16 7.16 7.15 7.01 6.99 6.98 6.95 6.95 6.95 6.94 6.94 6.93 6.91 6.90 6.86 6.86 6.84 6.84 6.72 6.70 4.18 4.17 3.44 3.29 3.26 3.10 2.94 2.90 2.25 2.25
F
CO_2Me
O
N
Me
1.59 1.01 1.02 0.82 2.19 1.00 0.98 1.00 3.00 1.08 3.02 1.02 0.95
f1 (ppm)

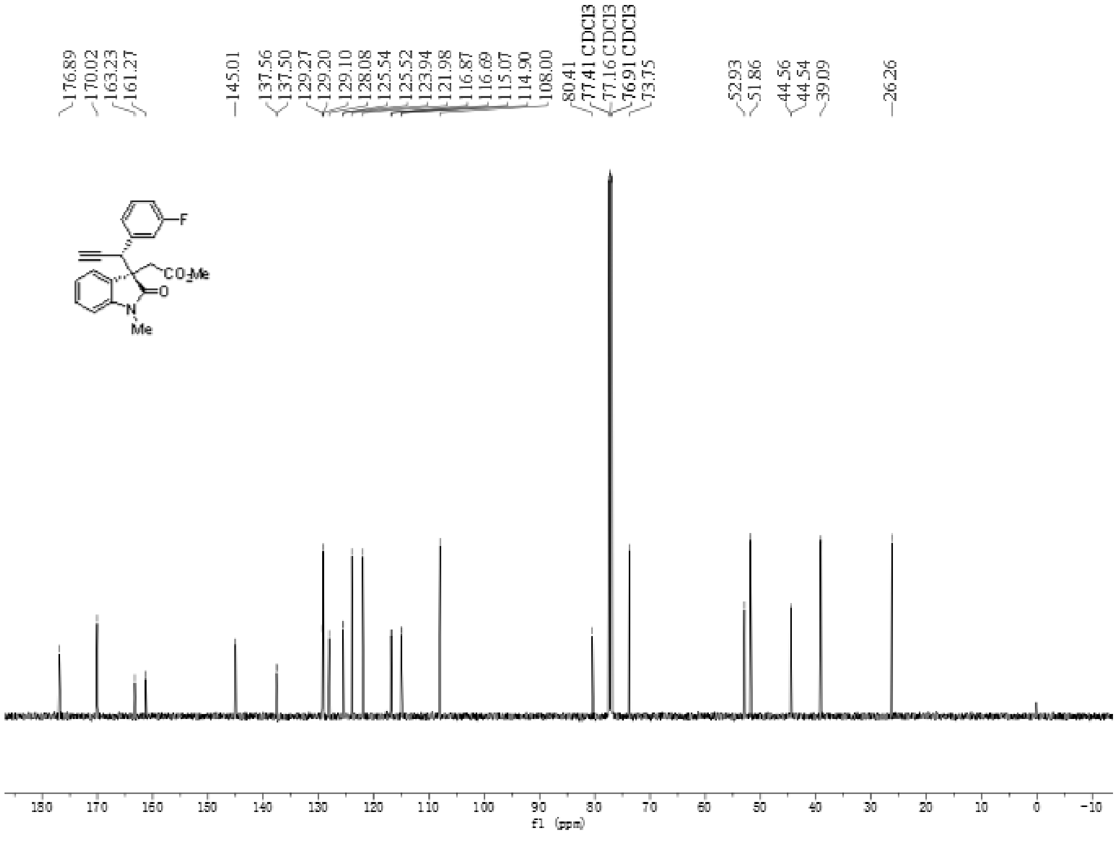
176.89 170.02 163.23 161.27 145.01 137.56 137.50 129.27 129.20 129.10 128.08 125.54 125.52 123.94 121.98 116.87 116.69 115.07 114.90 108.00 80.41 77.41 CDCl3 77.16 CDCl3 76.91 CDCl3 73.75 52.93 51.86 44.56 44.54 39.09 26.26
F
CO_2Me
O
N
Me
f1 (ppm)

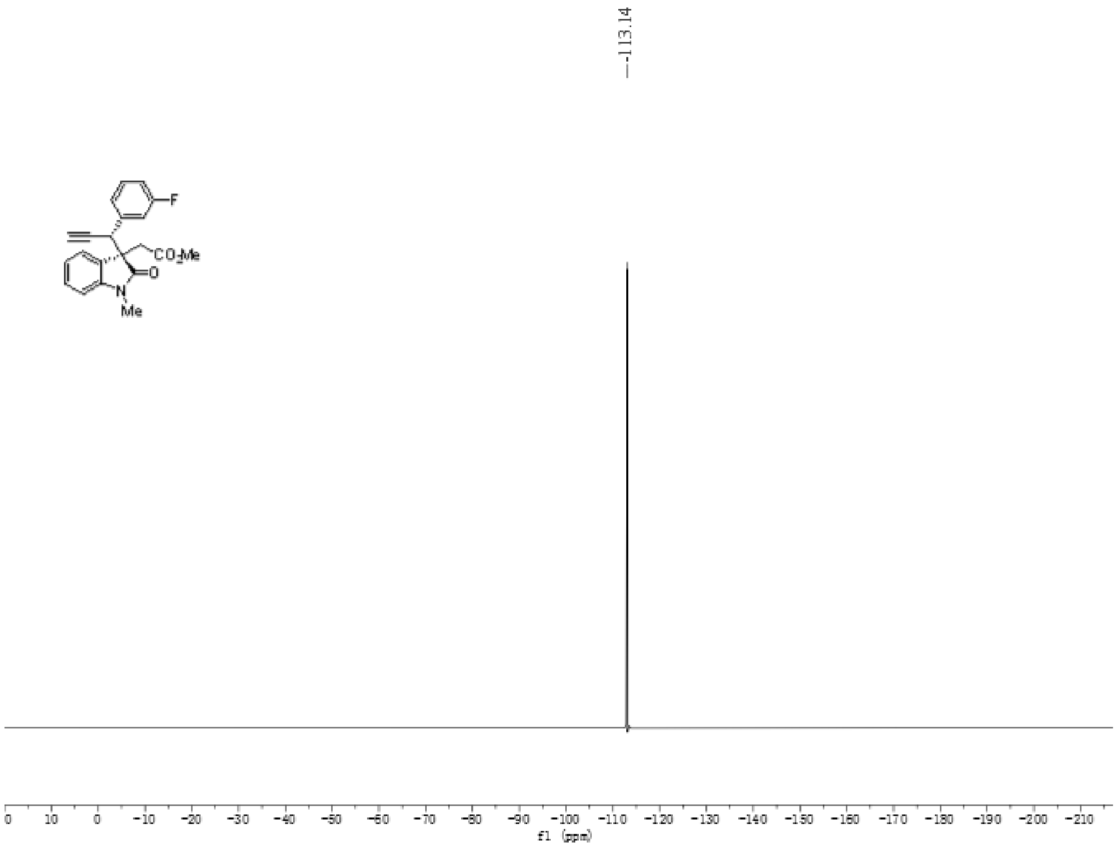

Supplementary Fig. 21. ^{1}H NMR ^{13}C NMR & ^{19}F NMR spectra of compound (*R*,*R*)-**3ag**

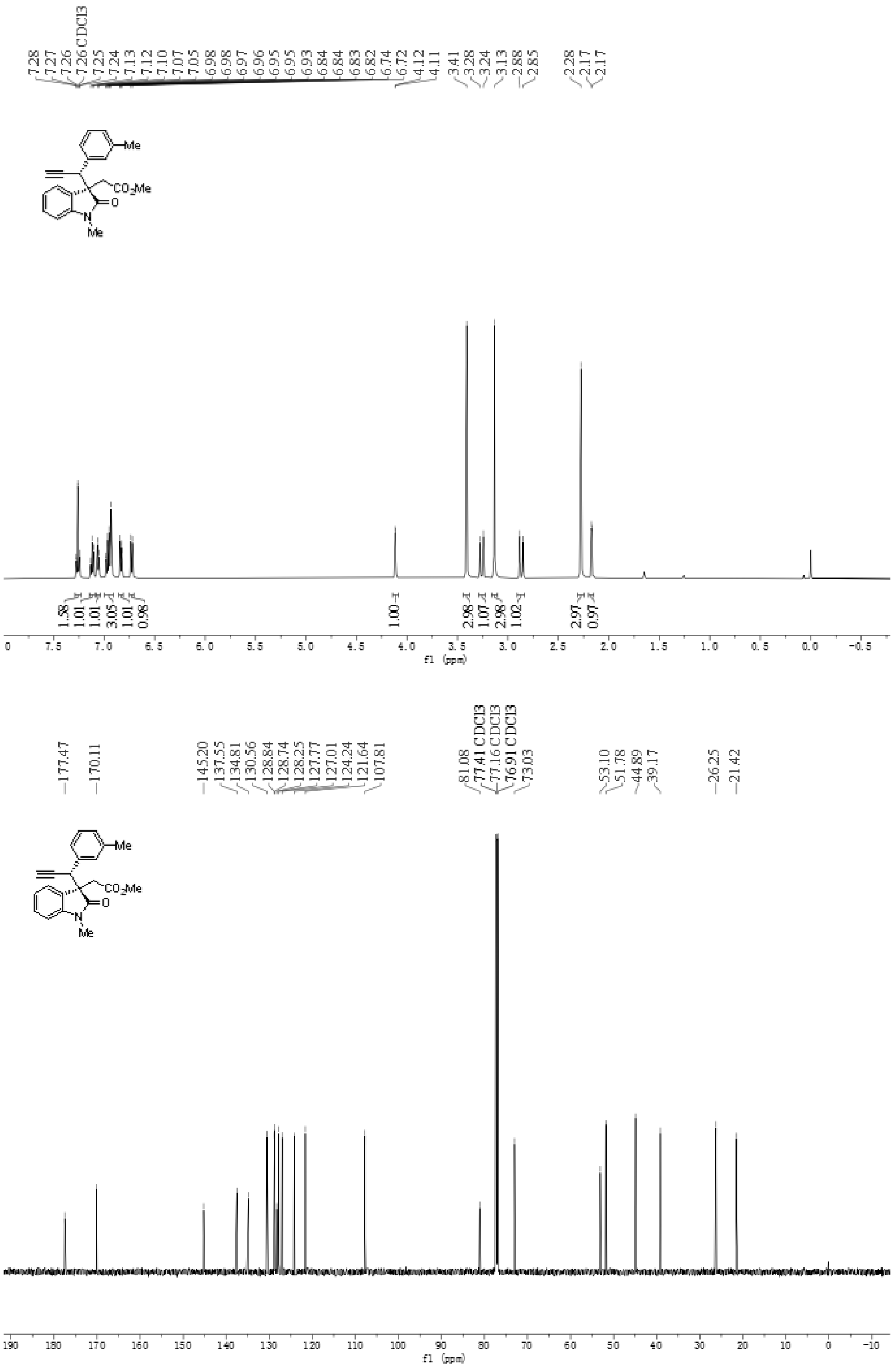

Supplementary Fig. 22. ^{1}H NMR & ^{13}C NMR spectra of compound (*R*,*R*)-**3ah**

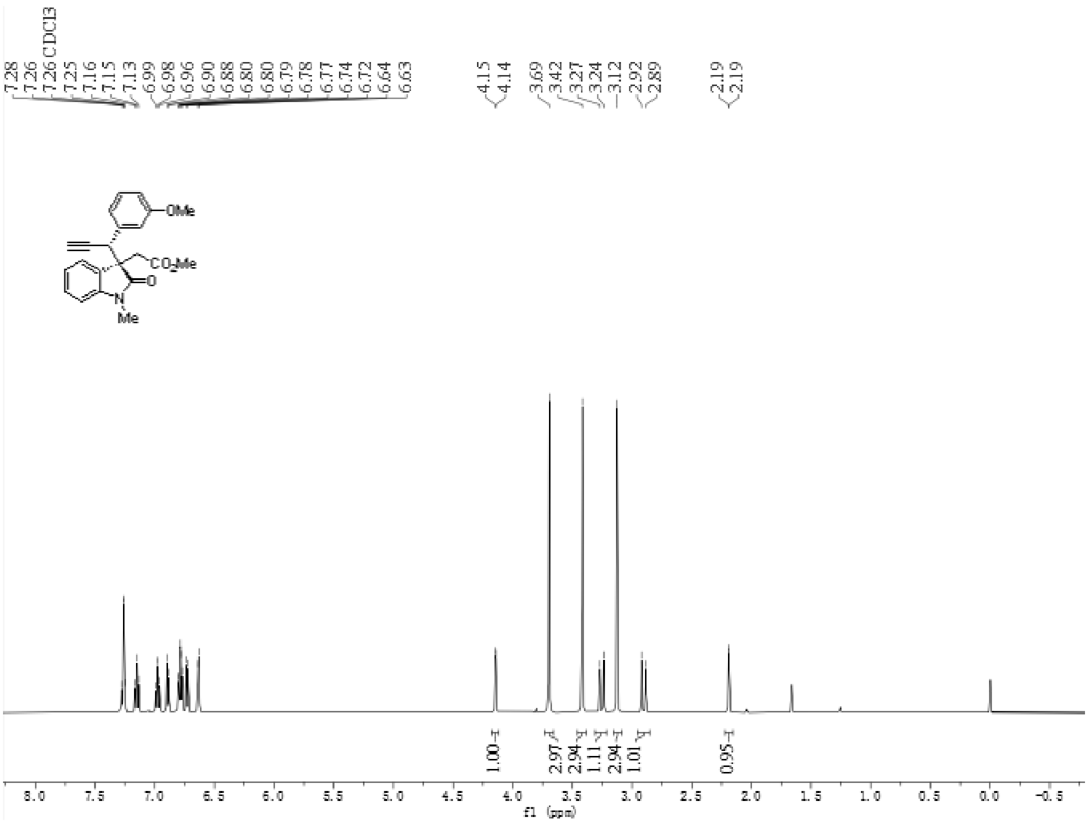

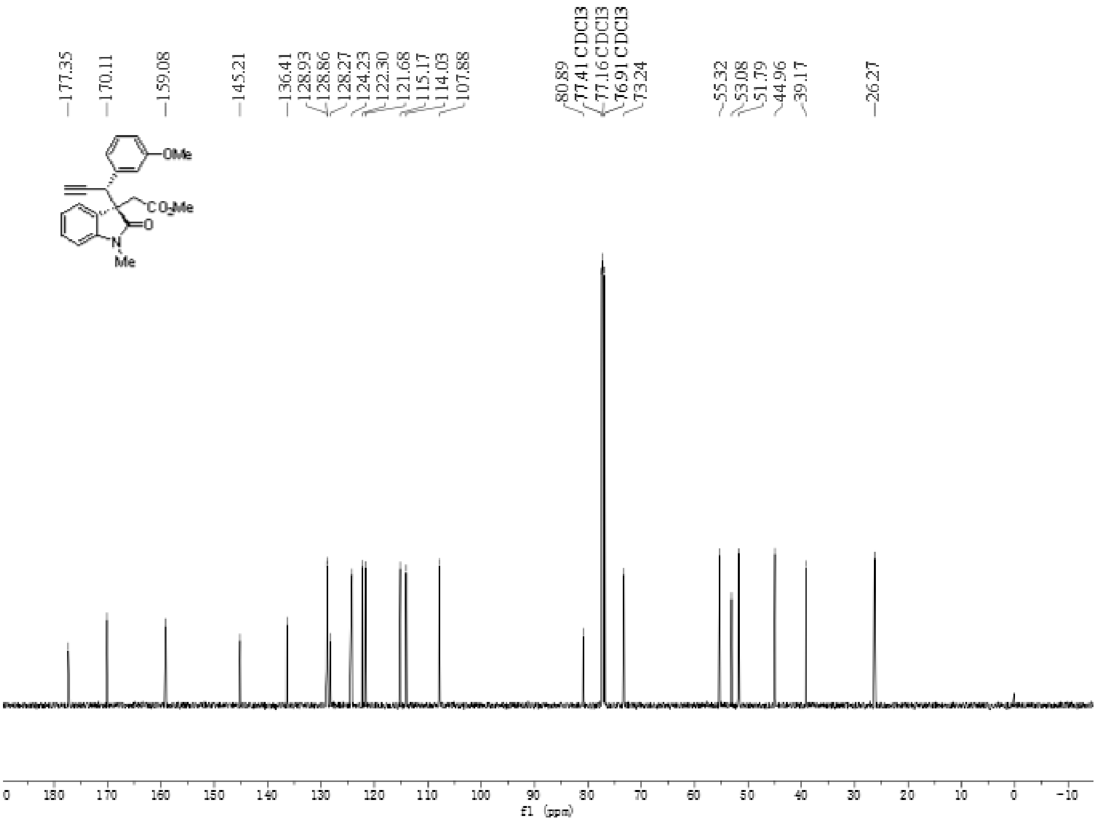

Supplementary Fig. 23. ^{1}H NMR & ^{13}C NMR spectra of compound (*R*,*R*)-**3ai**

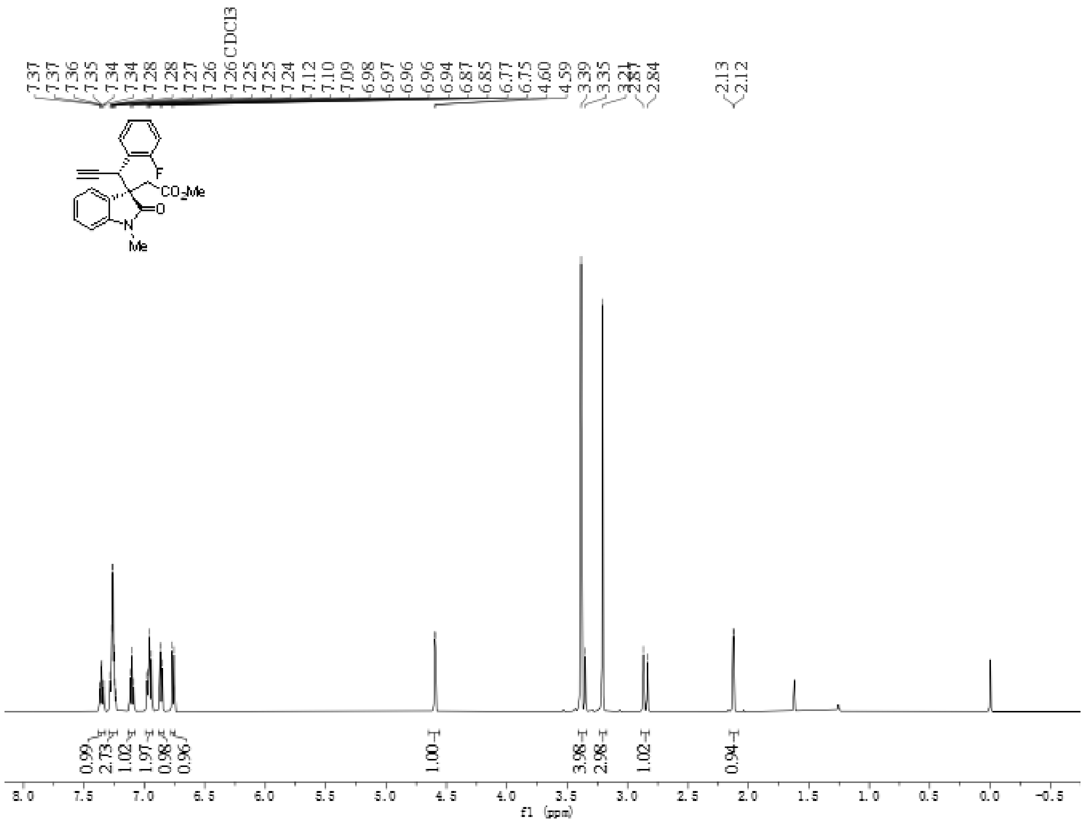

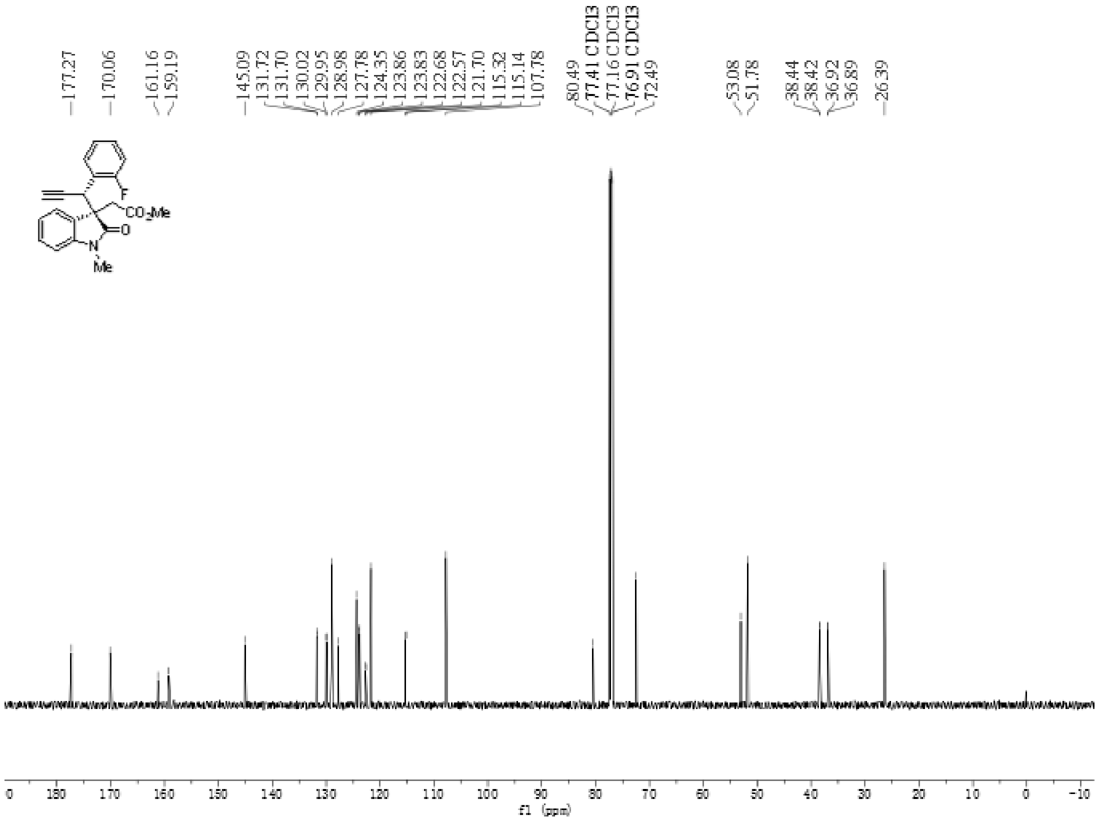

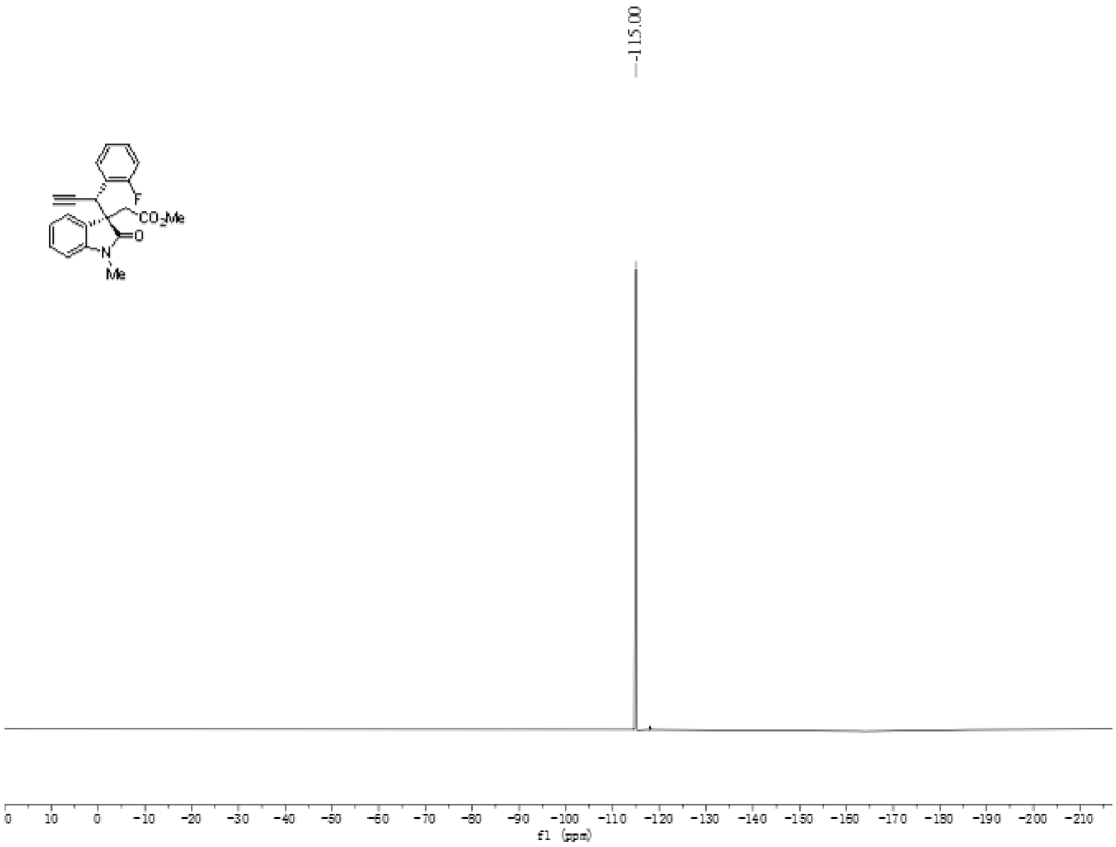

Supplementary Fig. 24. ^{1}H NMR ^{13}C NMR & ^{19}F NMR spectra of compound (*R*,*S*)-**3aj**

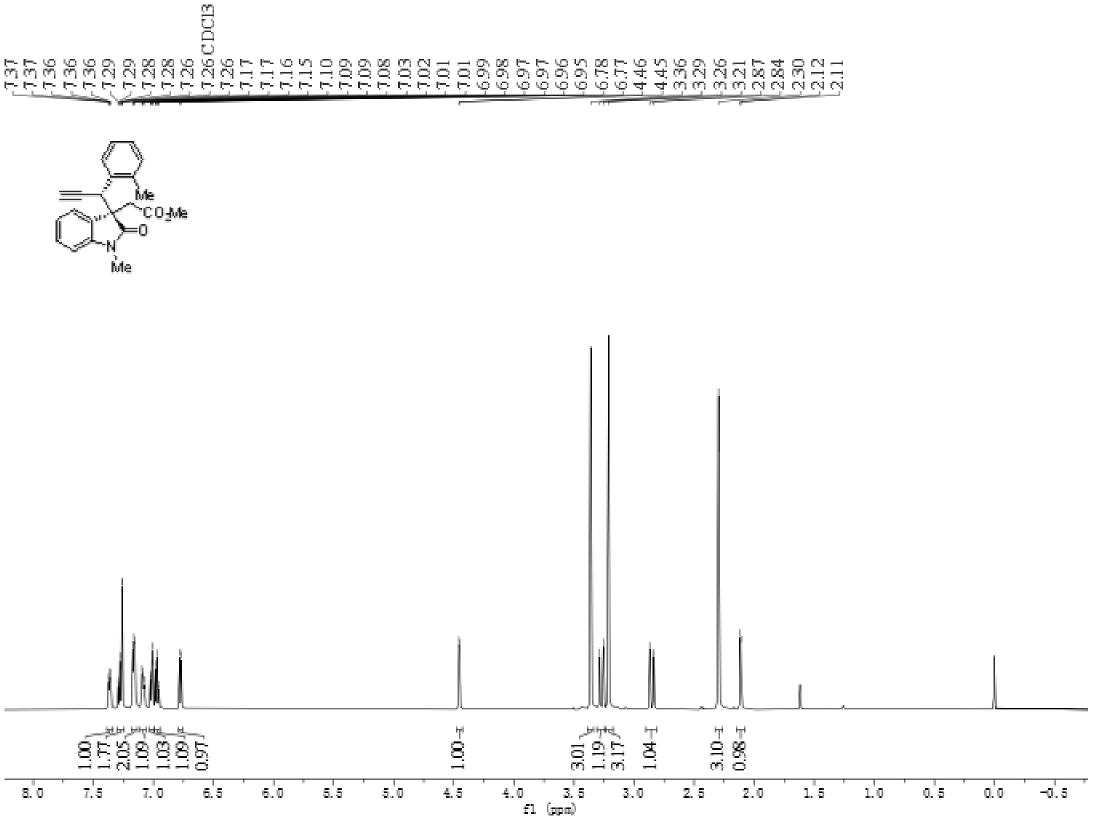

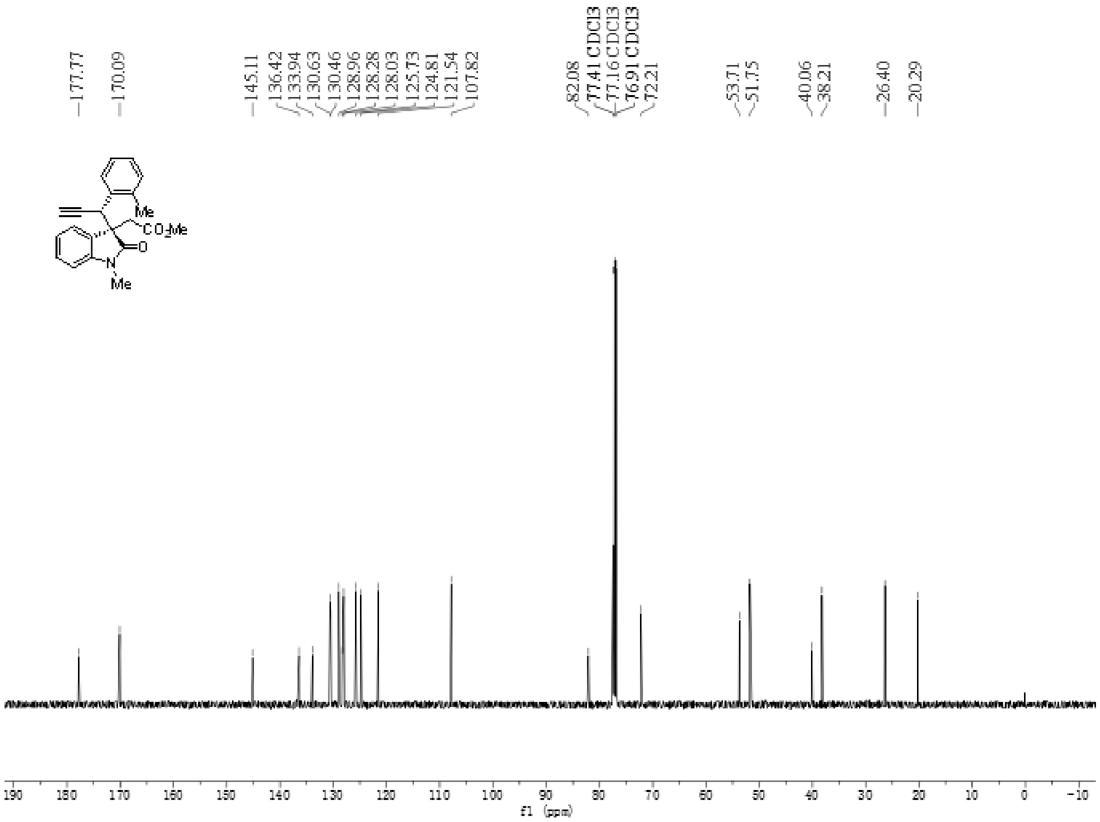

Supplementary Fig. 25. ^{1}H NMR & ^{13}C NMR spectra of compound (*R*,*R*)-**3ak**

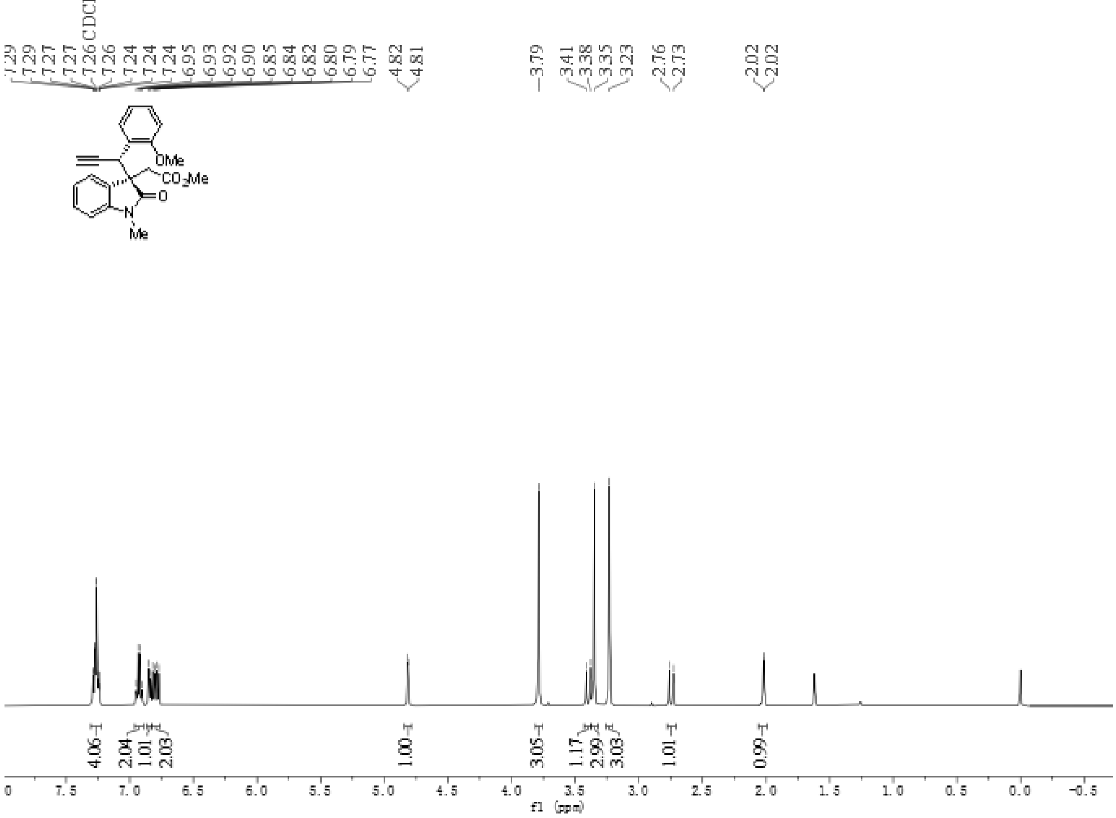

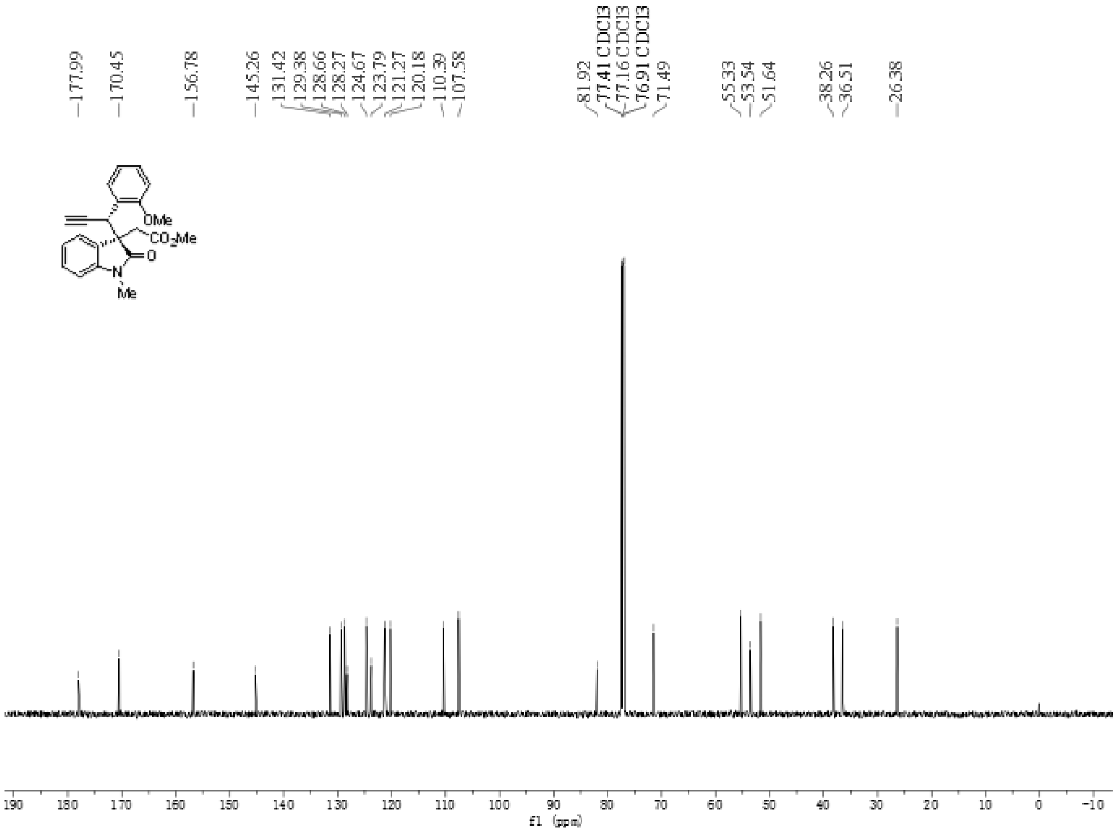

Supplementary Fig. 26. ^{1}H NMR & ^{13}C NMR spectra of compound (*R*,*R*)-**3al**

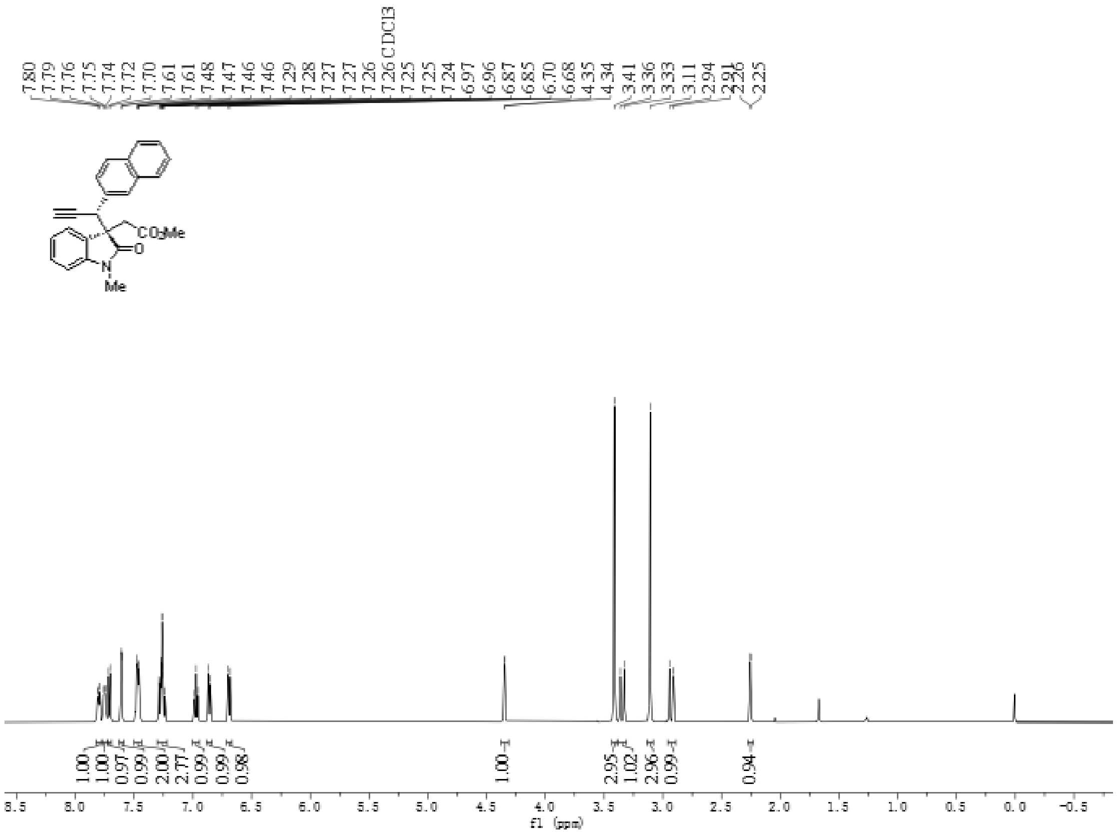

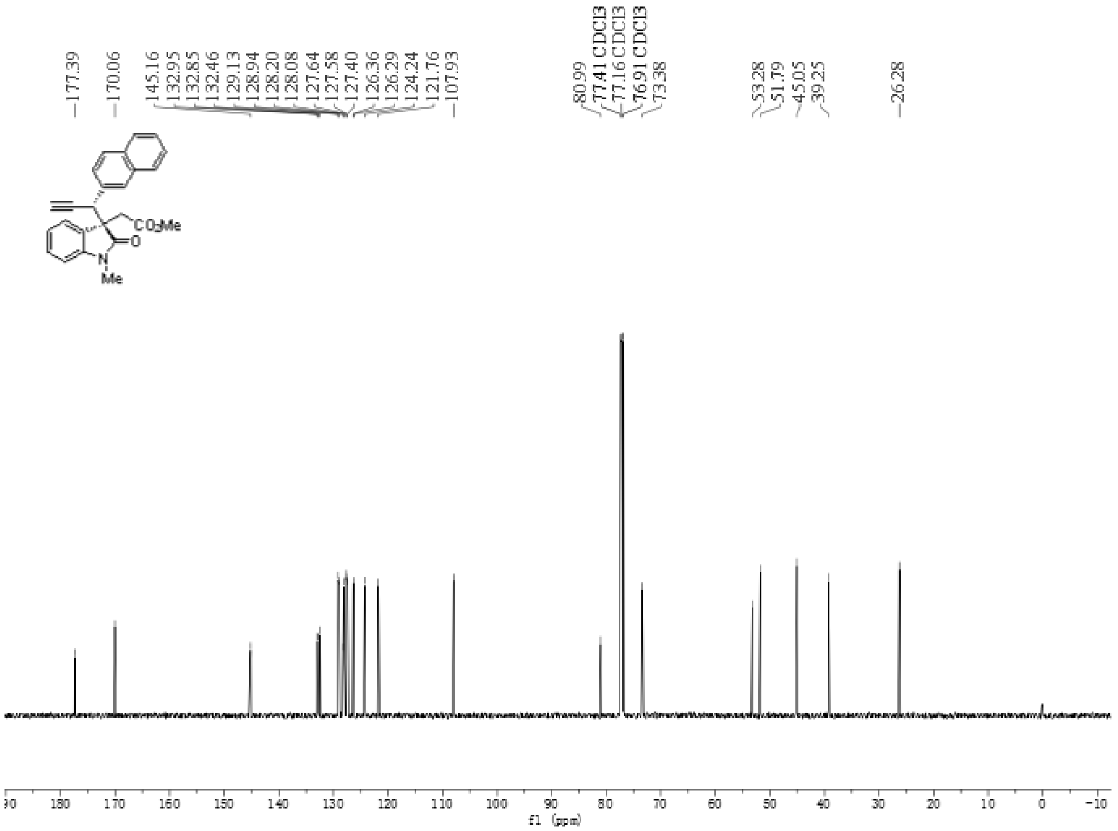

Supplementary Fig. 27. ^{1}H NMR & ^{13}C NMR spectra of compound (*R*,*R*)-**3am**

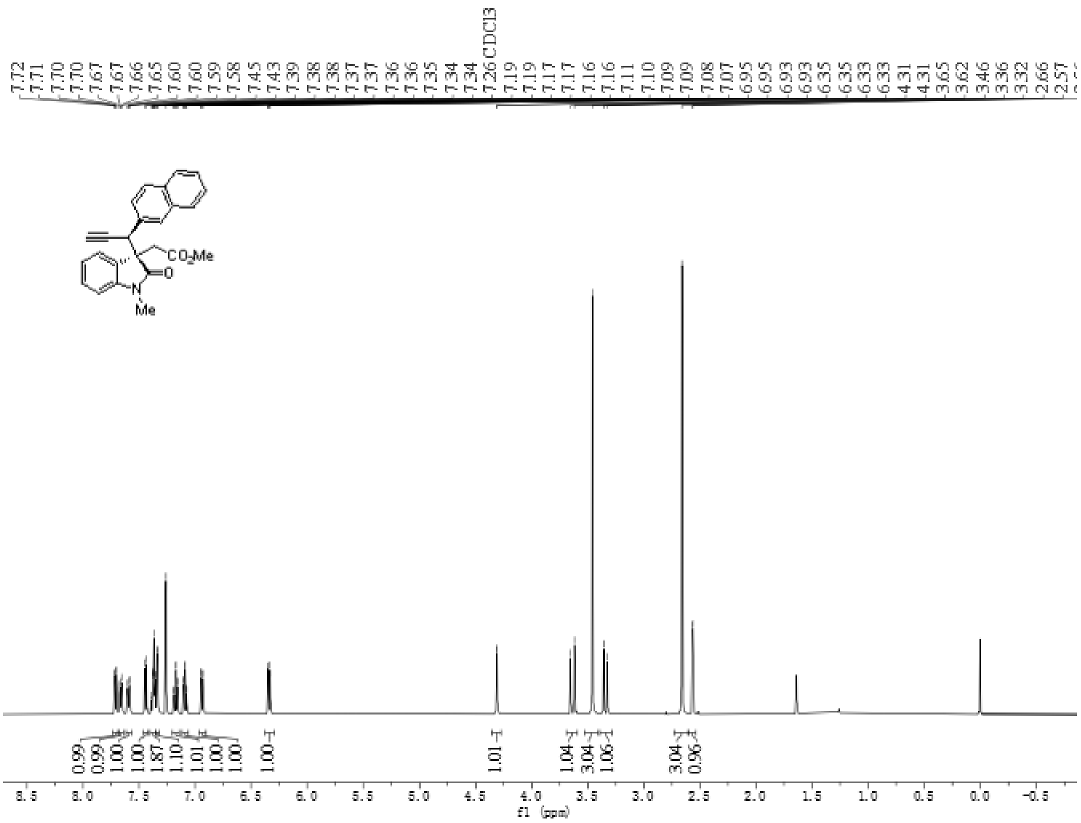

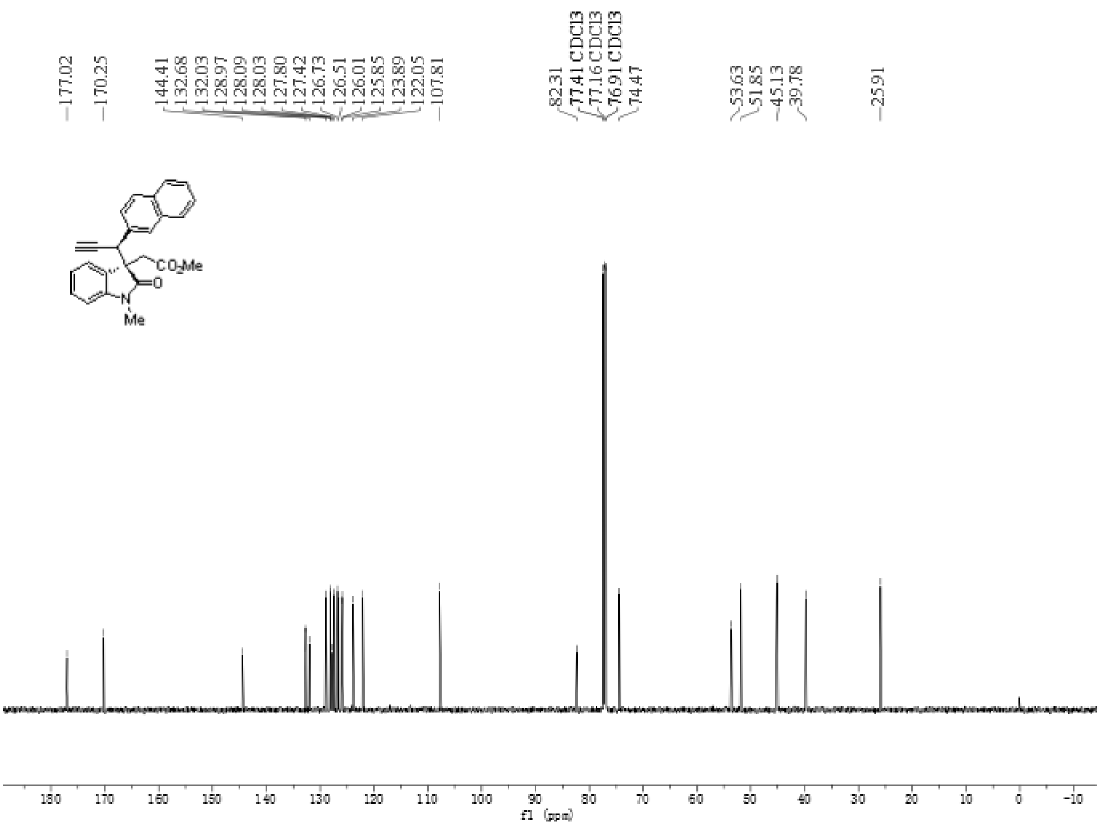

Supplementary Fig. 28. ^{1}H NMR & ^{13}C NMR spectra of compound (*R*,*S*)-**3am**

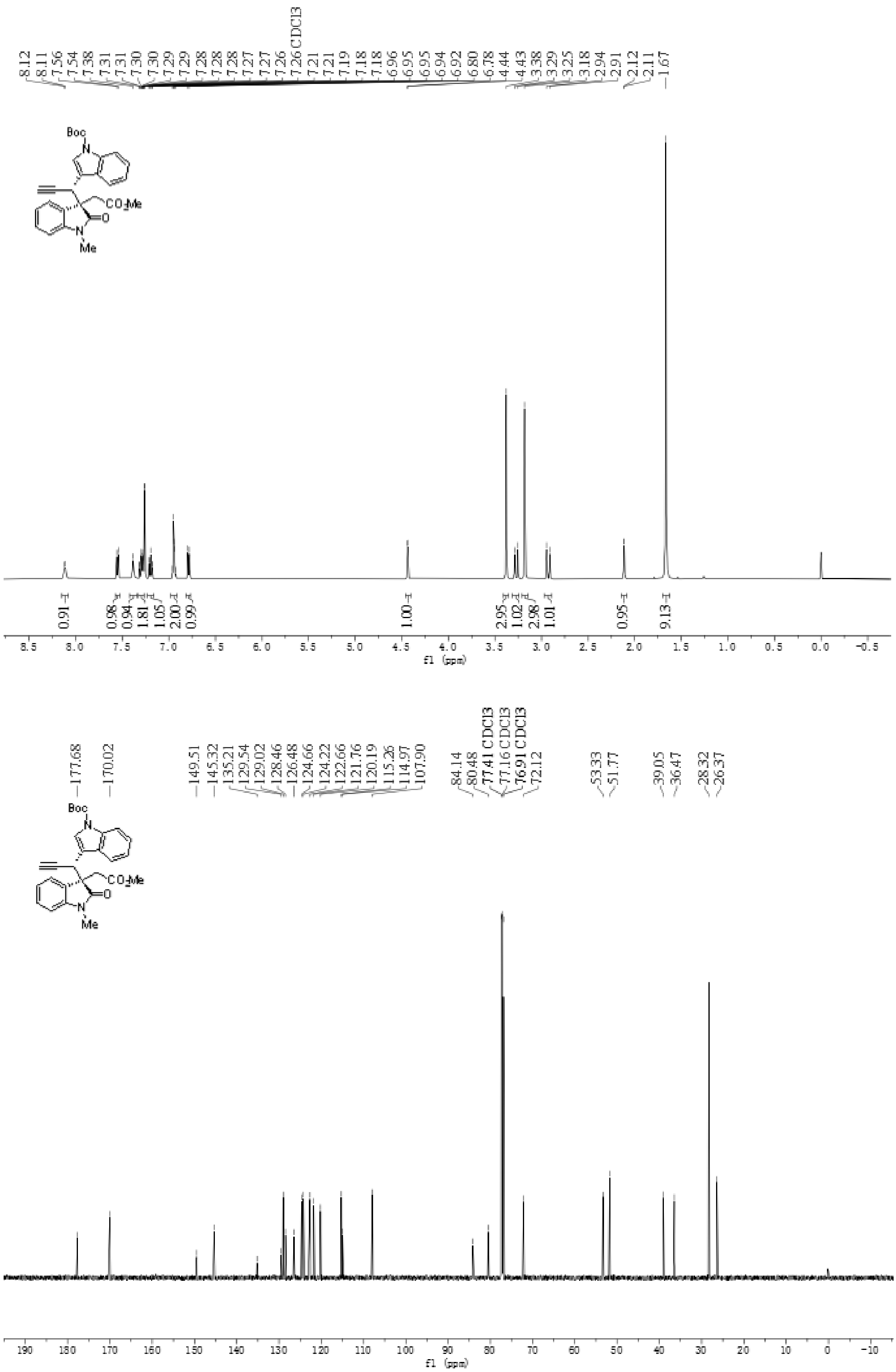

Supplementary Fig. 29. ^{1}H NMR & ^{13}C NMR spectra of compound (*R*,*R*)-**3an**

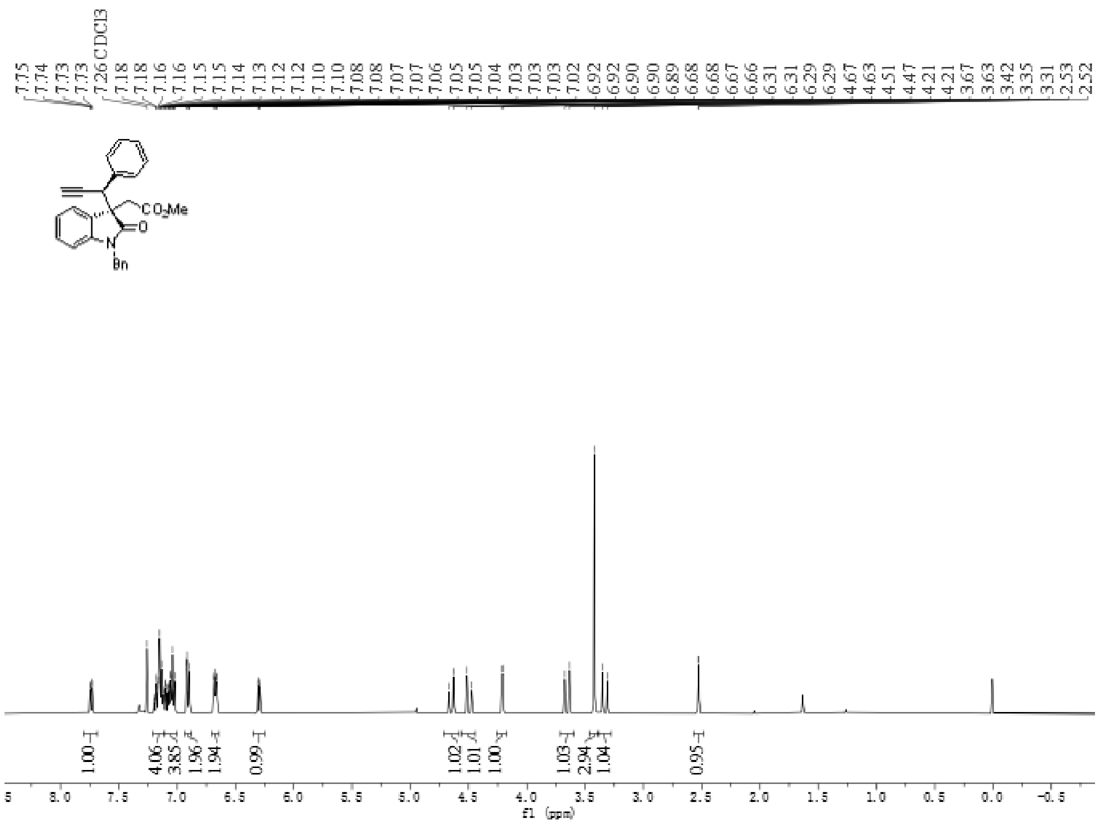

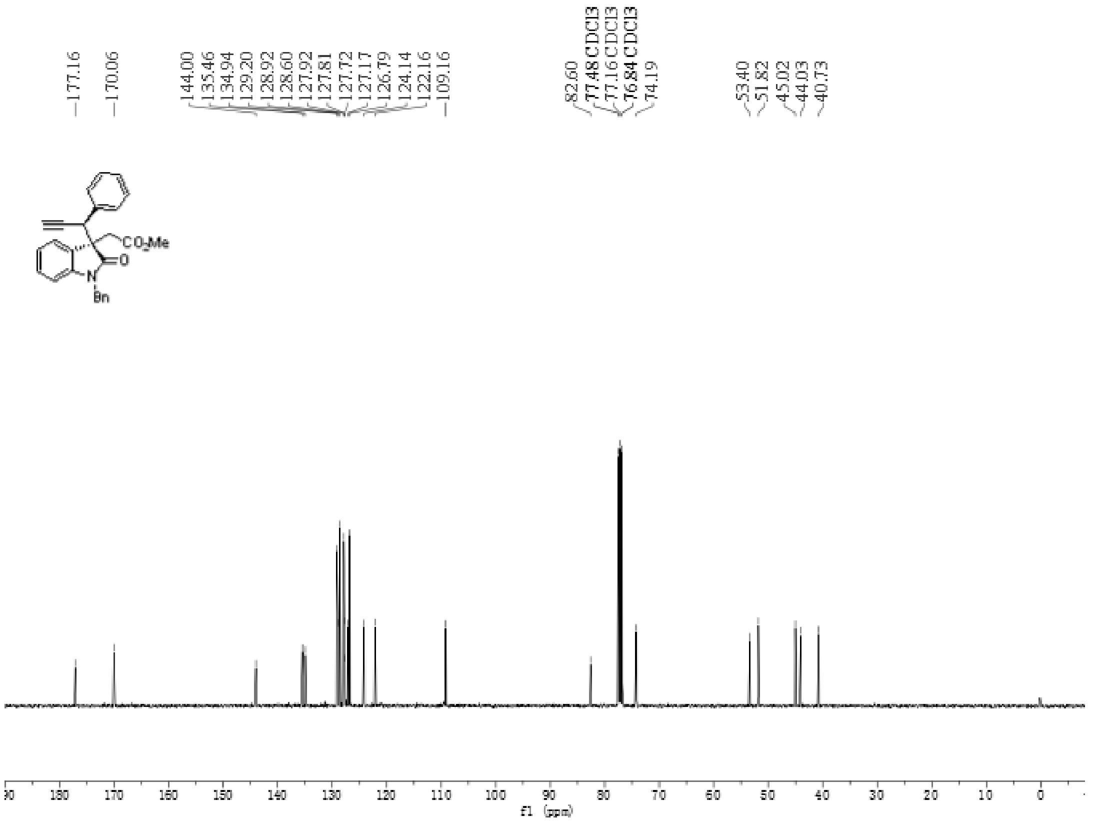

Supplementary Fig. 30. ^{1}H NMR & ^{13}C NMR spectra of compound (*R*,*S*)-**3ca**

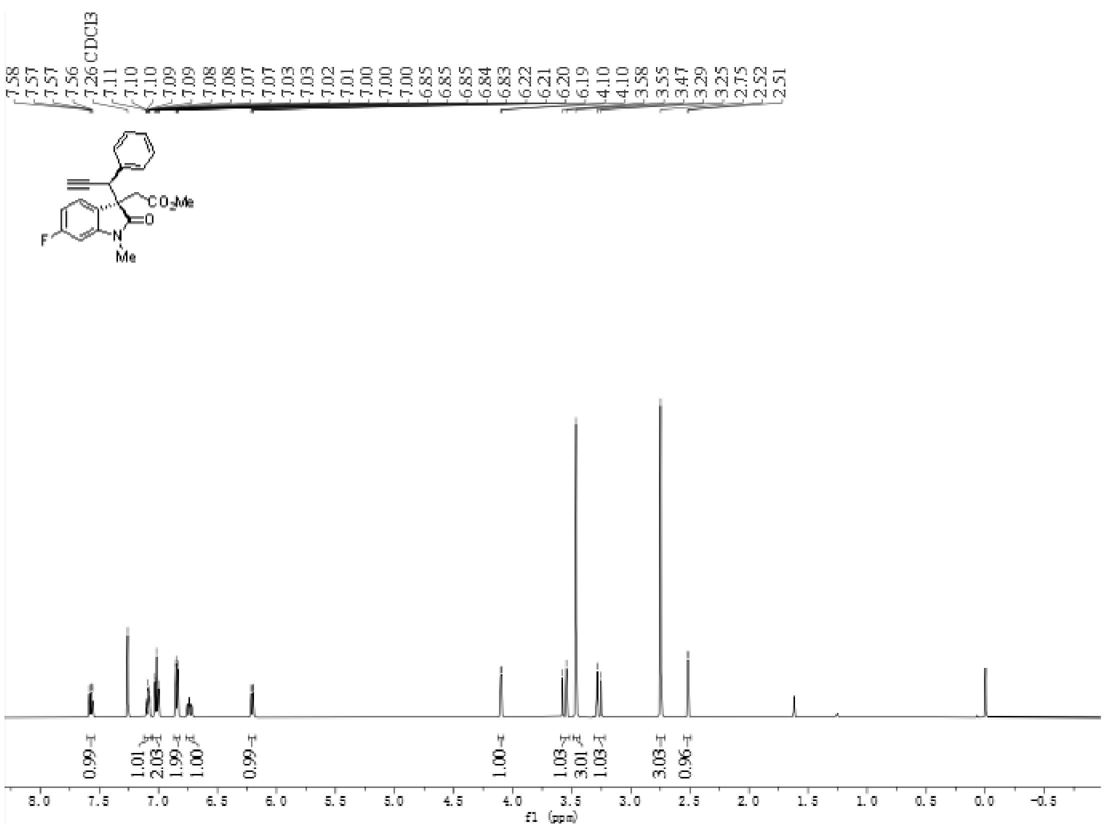
7.58
7.57
7.57
7.56
7.26 CDCl3
7.11
7.10
7.10
7.09
7.09
7.08
7.08
7.07
7.07
7.03
7.03
7.02
7.01
7.00
7.00
7.00
6.85
6.85
6.84
6.83
6.22
6.21
6.20
6.19
4.10
4.10
3.58
3.55
3.47
3.29
3.25
2.75
2.52
2.51
CO2Me
O
N
Me
F
0.99
1.01
2.03
1.99
1.00
0.99
1.00
1.03
3.01
1.03
3.03
0.96
8.0 7.5 7.0 6.5 6.0 5.5 5.0 4.5 4.0 3.5 3.0 2.5 2.0 1.5 1.0 0.5 0.0 -0.5
f1 (ppm)

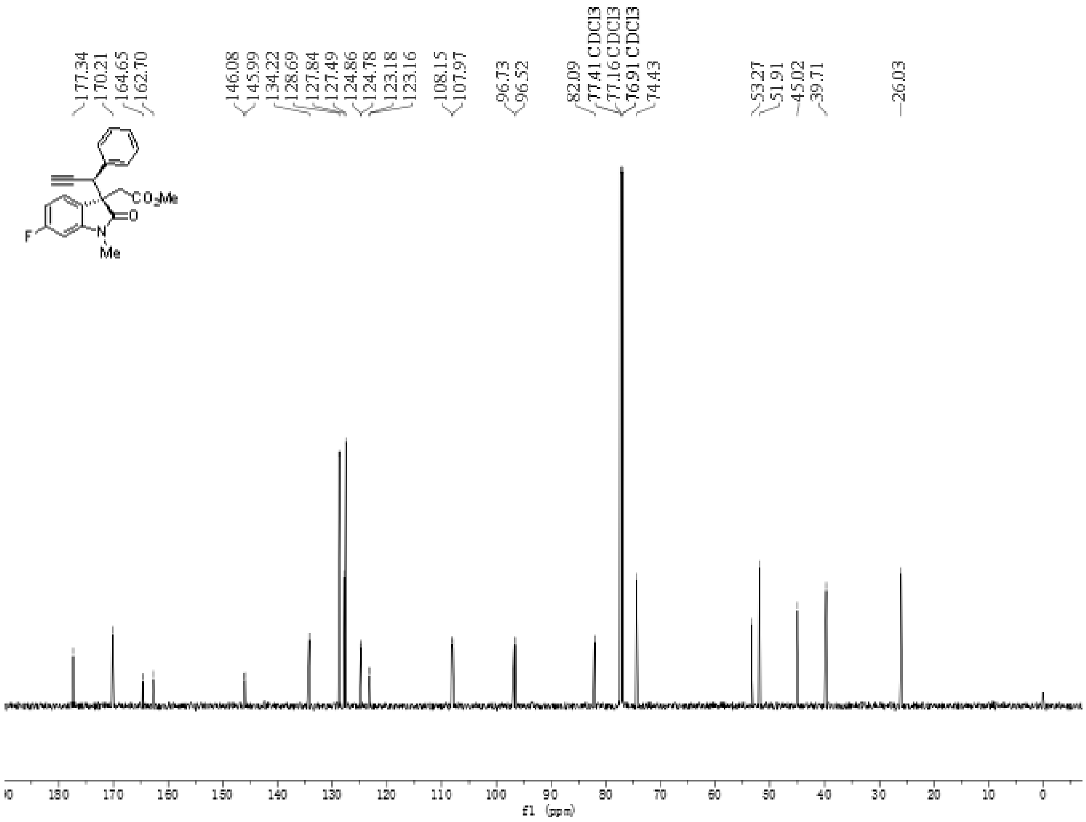
177.34
170.21
164.65
162.70
146.08
145.99
134.22
128.69
127.84
127.49
124.86
124.78
123.18
123.16
108.15
107.97
96.73
96.52
82.09
77.41 CDCl3
77.16 CDCl3
76.91 CDCl3
74.43
53.27
51.91
45.02
39.71
26.03
CO2Me
O
N
Me
F
180 170 160 150 140 130 120 110 100 90 80 70 60 50 40 30 20 10 0
f1 (ppm)

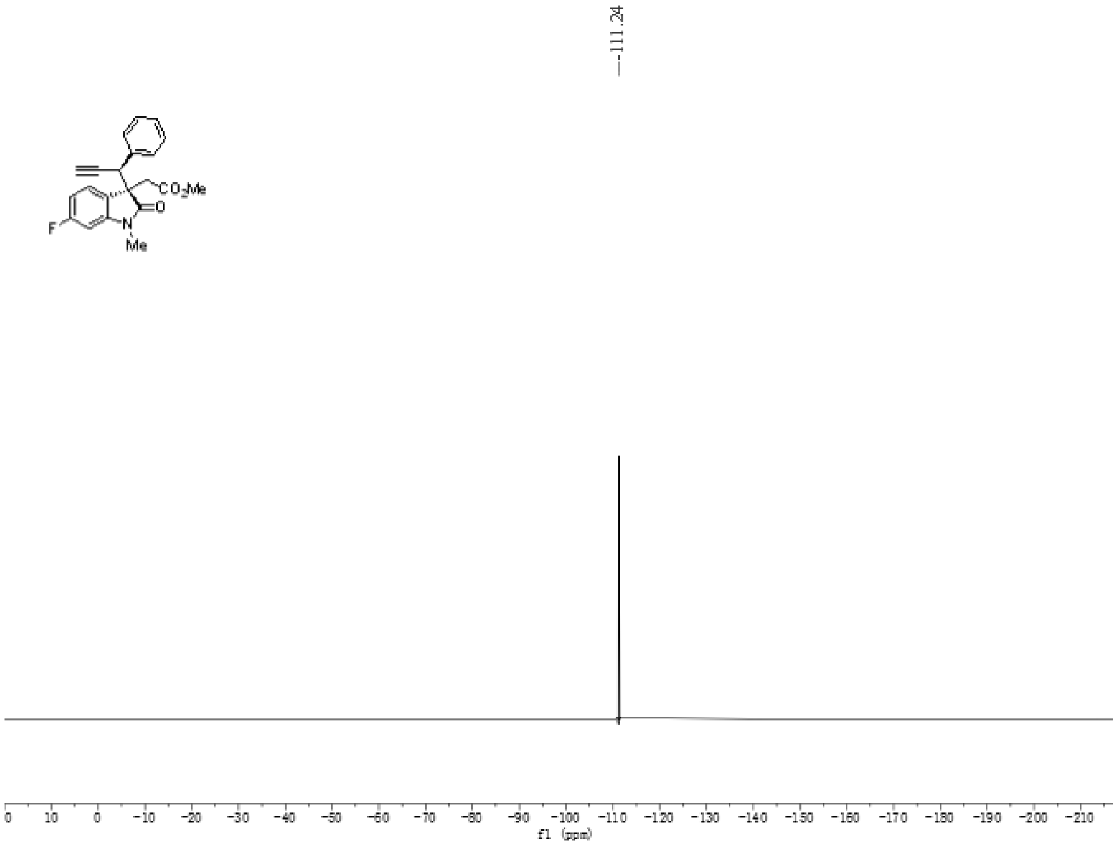

Supplementary Fig. 31. ^{1}H NMR ^{13}C NMR & ^{19}F NMR spectra of compound (*R*,*S*)-**3ka**

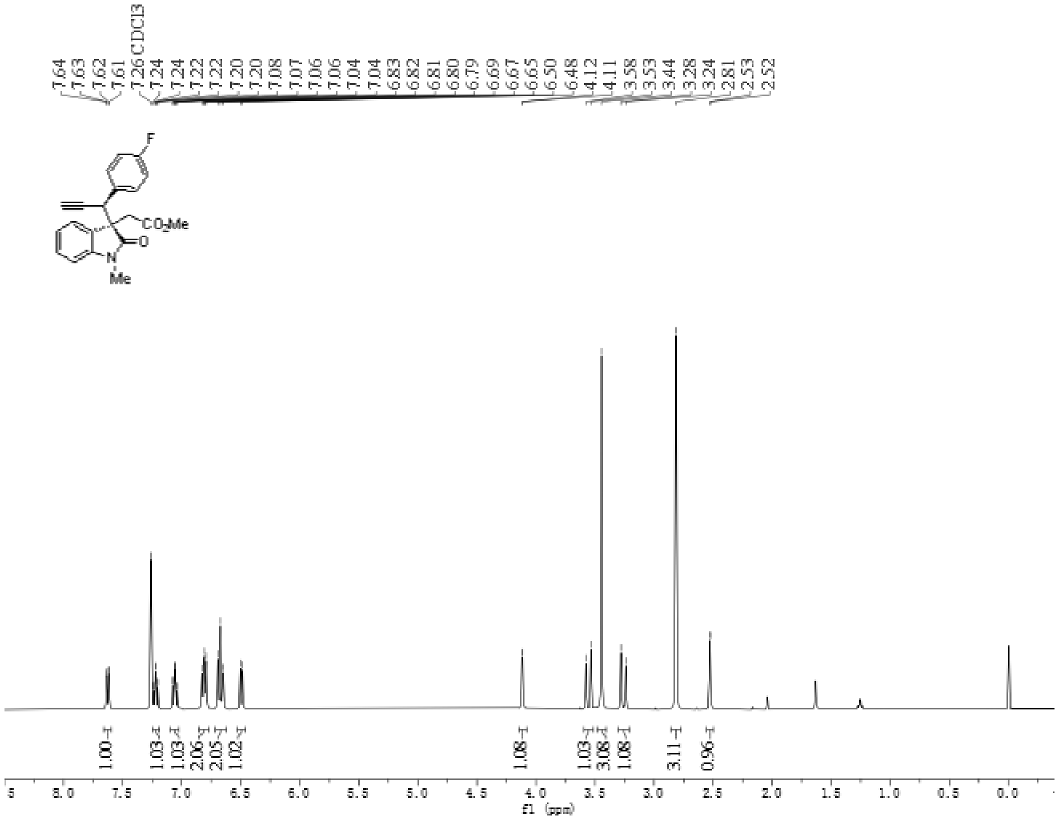

7.64
7.63
7.62
7.61
7.26 CDCl3
7.24
7.24
7.22
7.22
7.20
7.20
7.08
7.07
7.06
7.06
7.04
7.04
6.83
6.82
6.81
6.80
6.79
6.69
6.67
6.65
6.50
6.48
4.12
4.11
3.58
3.53
3.44
3.28
3.24
2.81
2.53
2.52
F
CO_2Me
O
N
Me
1.00
1.03
1.03
2.06
2.05
1.02
1.08
1.03
3.08
1.08
3.11
0.96
f1 (ppm)

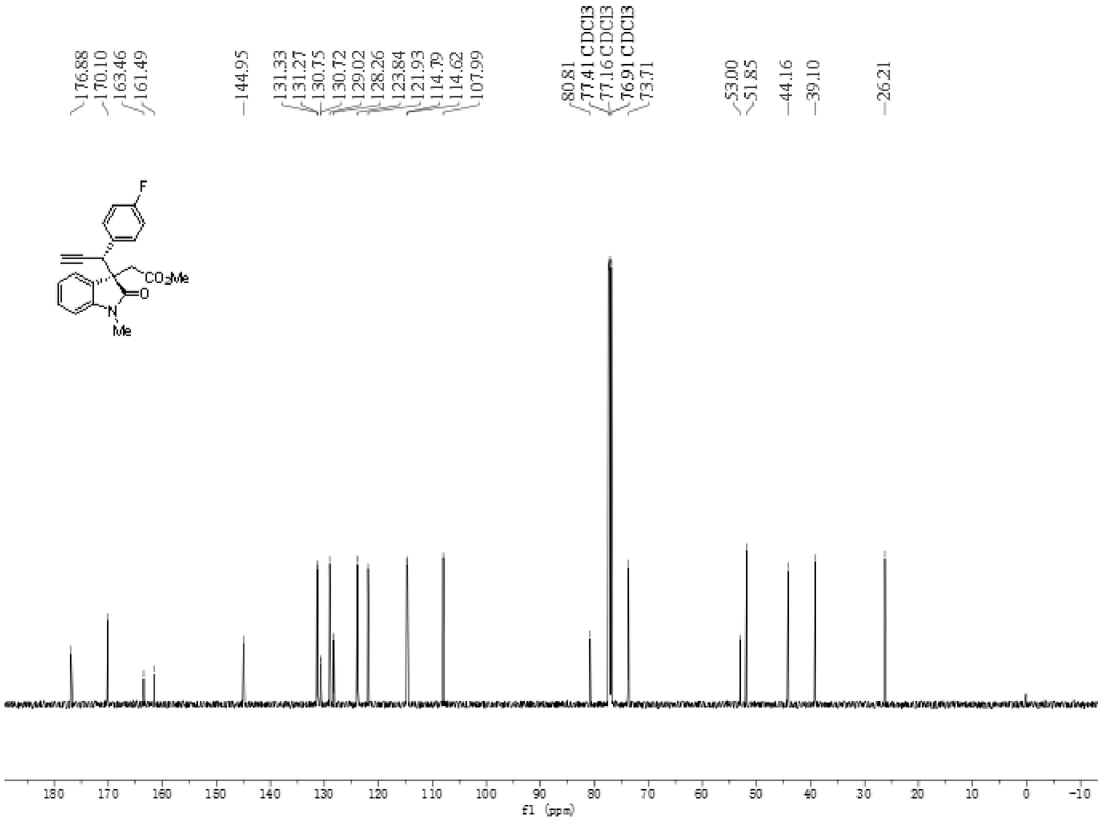

176.88
170.10
163.46
161.49
144.95
131.33
131.27
130.75
130.72
129.02
128.26
123.84
121.93
114.79
114.62
107.99
80.81
77.41 CDCl3
77.16 CDCl3
76.91 CDCl3
73.71
53.00
51.85
44.16
39.10
26.21
F
CO_2Me
O
N
Me
f1 (ppm)

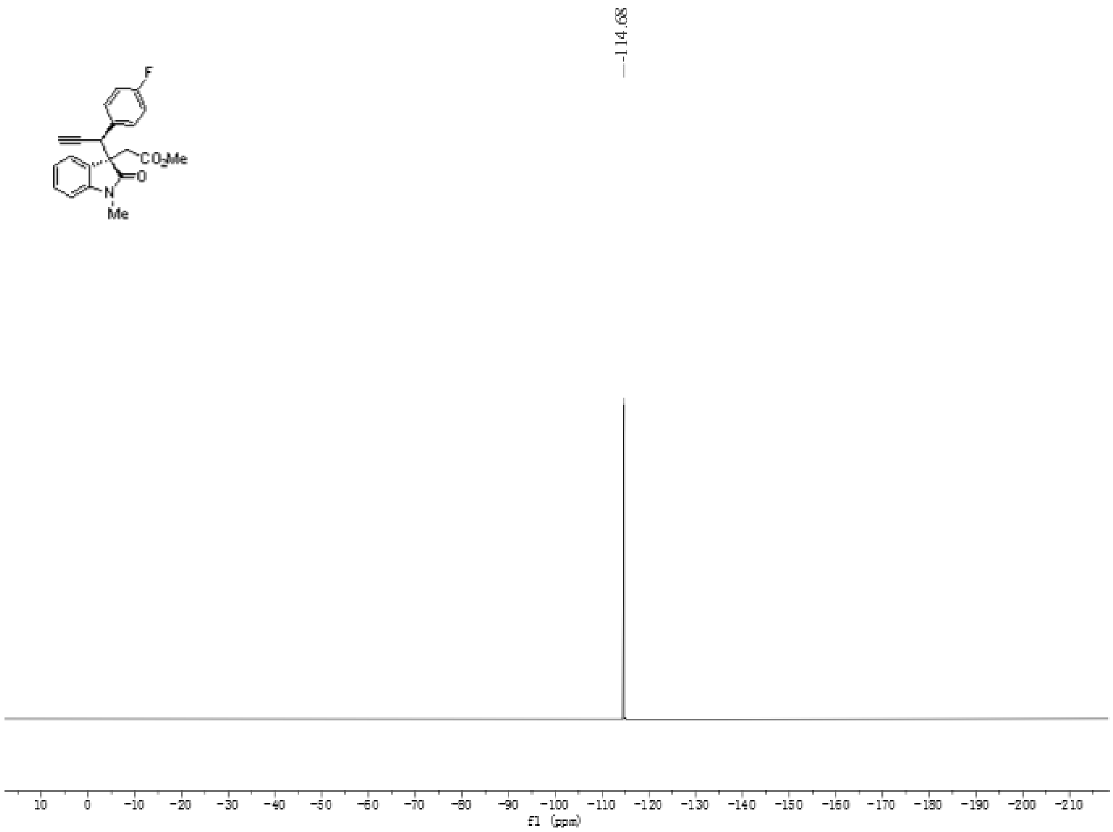

Supplementary Fig. 32. ^{1}H NMR ^{13}C NMR & ^{19}F NMR spectra of compound (*R*,*S*)-**3ab**

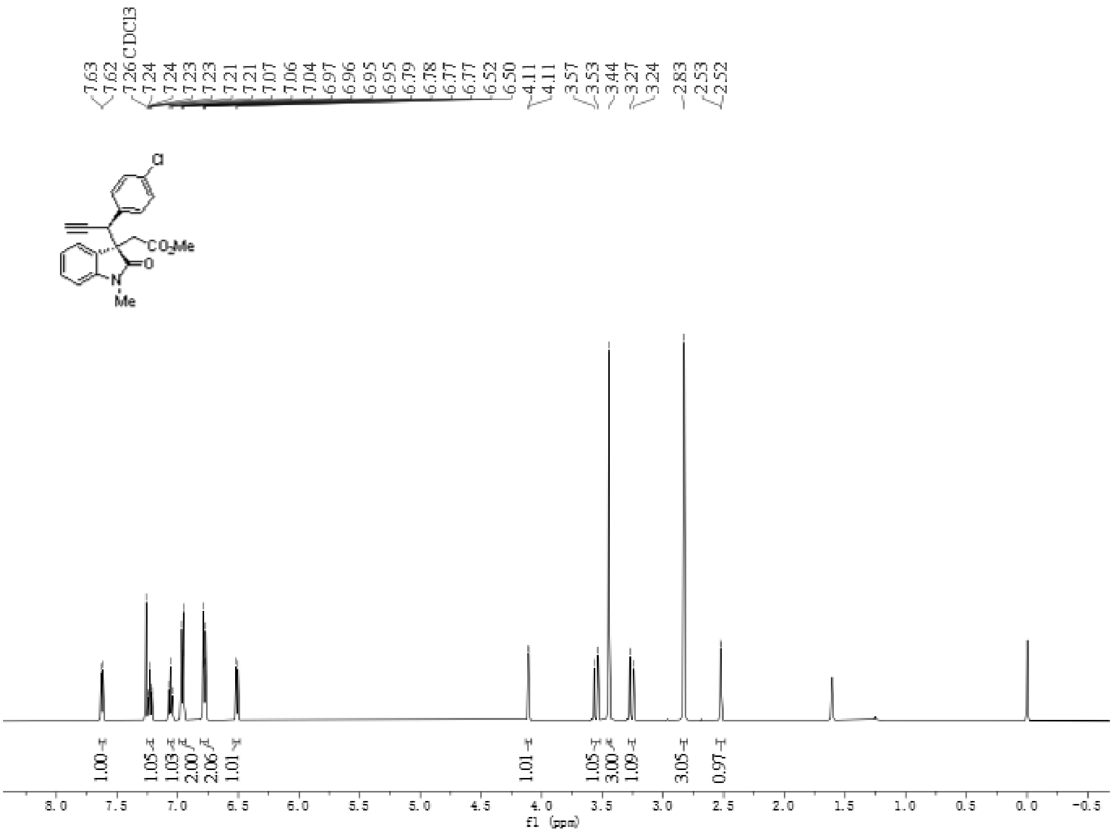

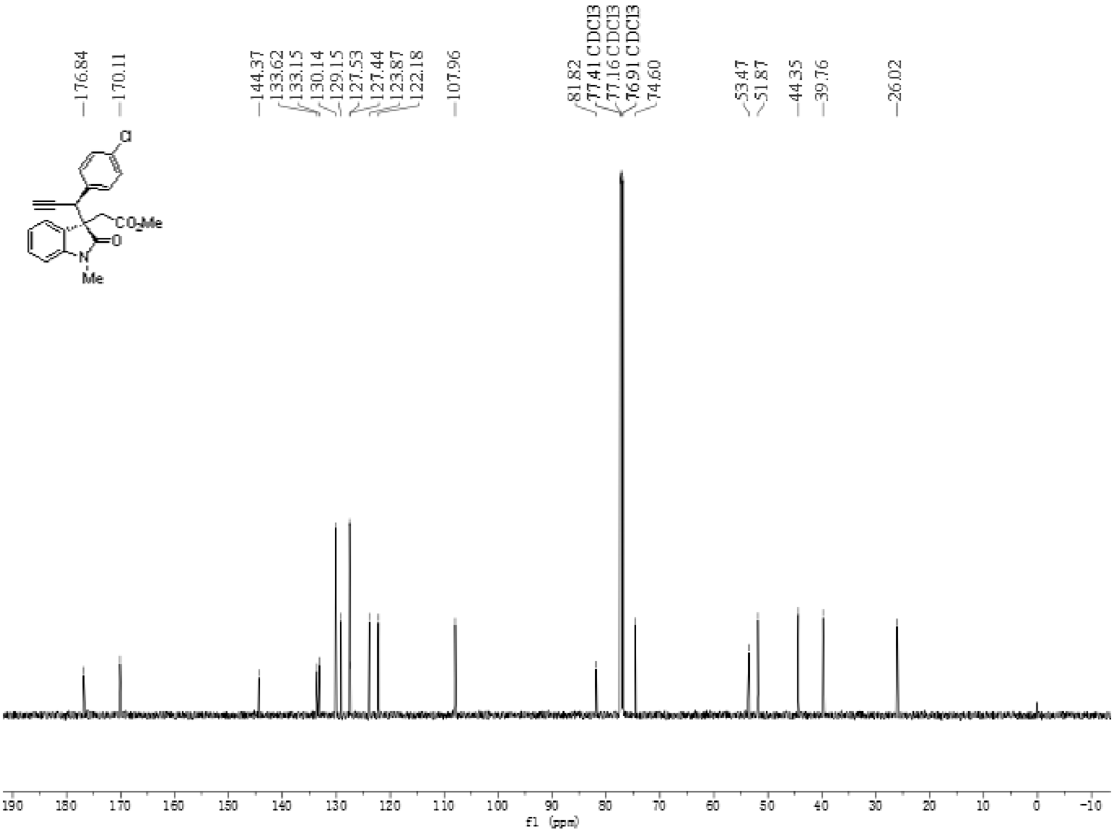

Supplementary Fig. 33. ^{1}H NMR & ^{13}C NMR spectra of compound (*R*,*S*)-**3ac**

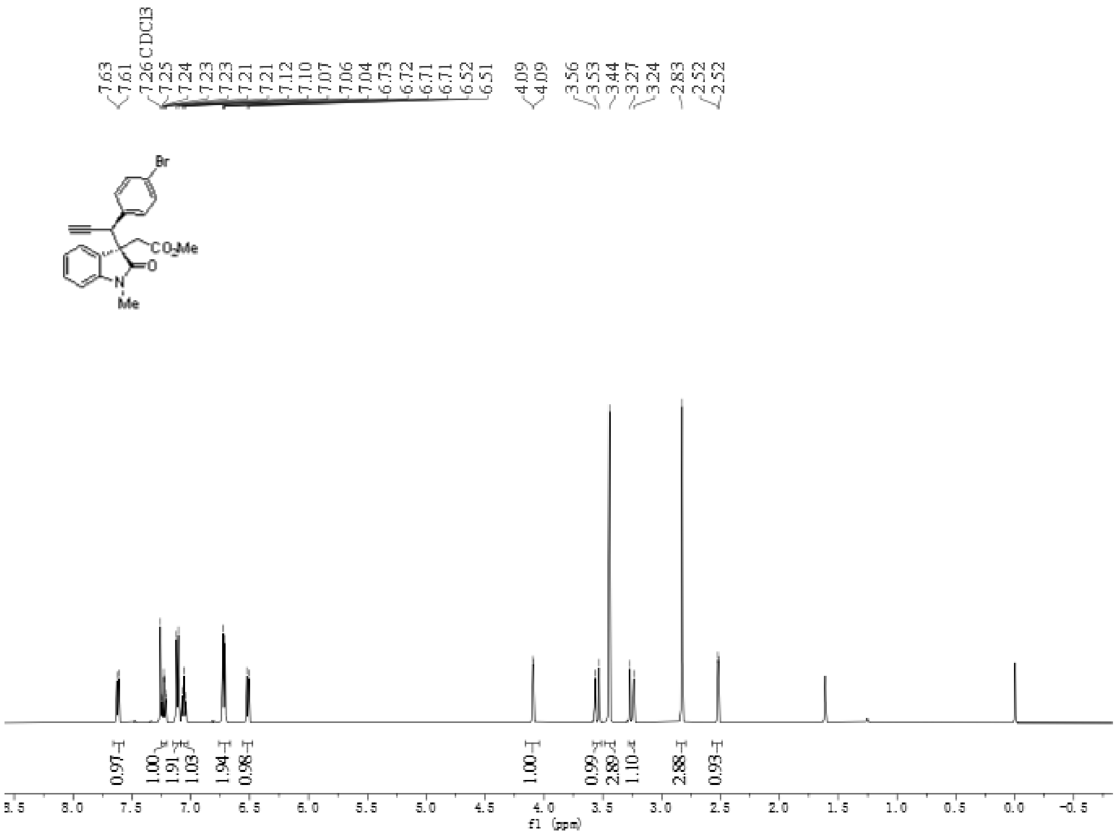

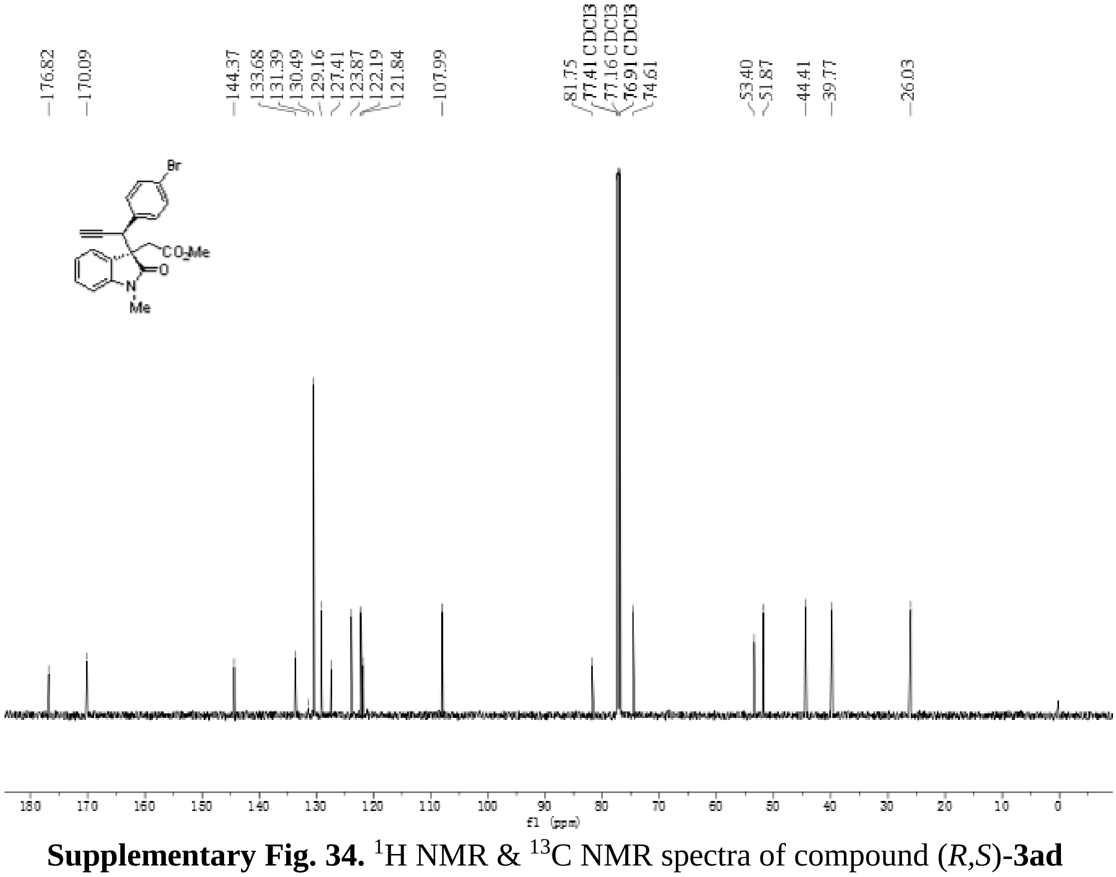

Supplementary Fig. 34. ^{1}H NMR & ^{13}C NMR spectra of compound (*R*,*S*)-**3ad**

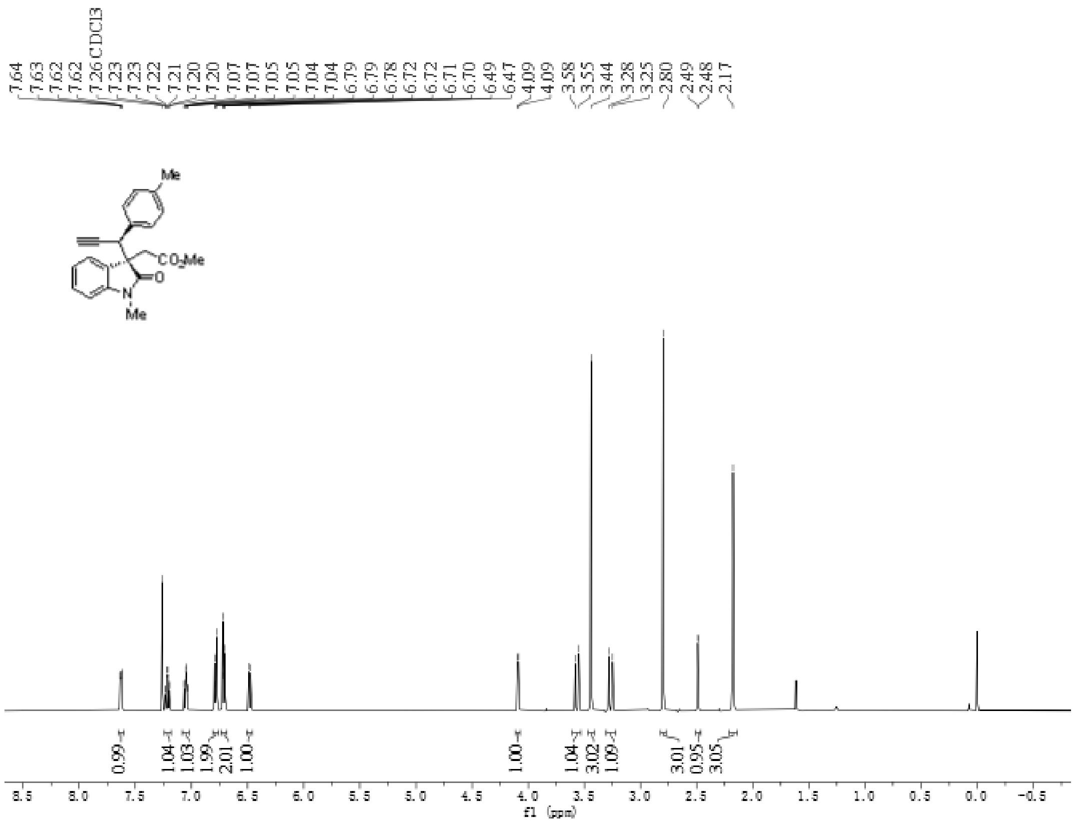

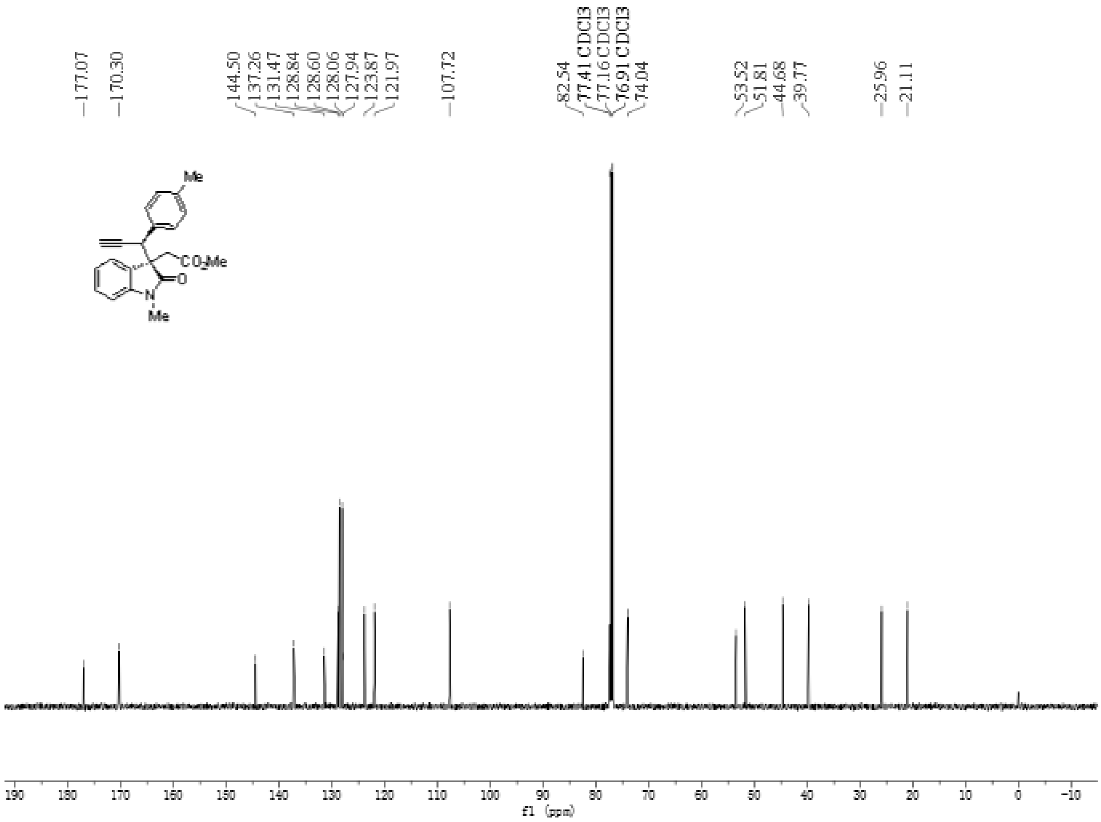

Supplementary Fig. 35. ^{1}H NMR & ^{13}C NMR spectra of compound (*R*,*S*)-**3ae**

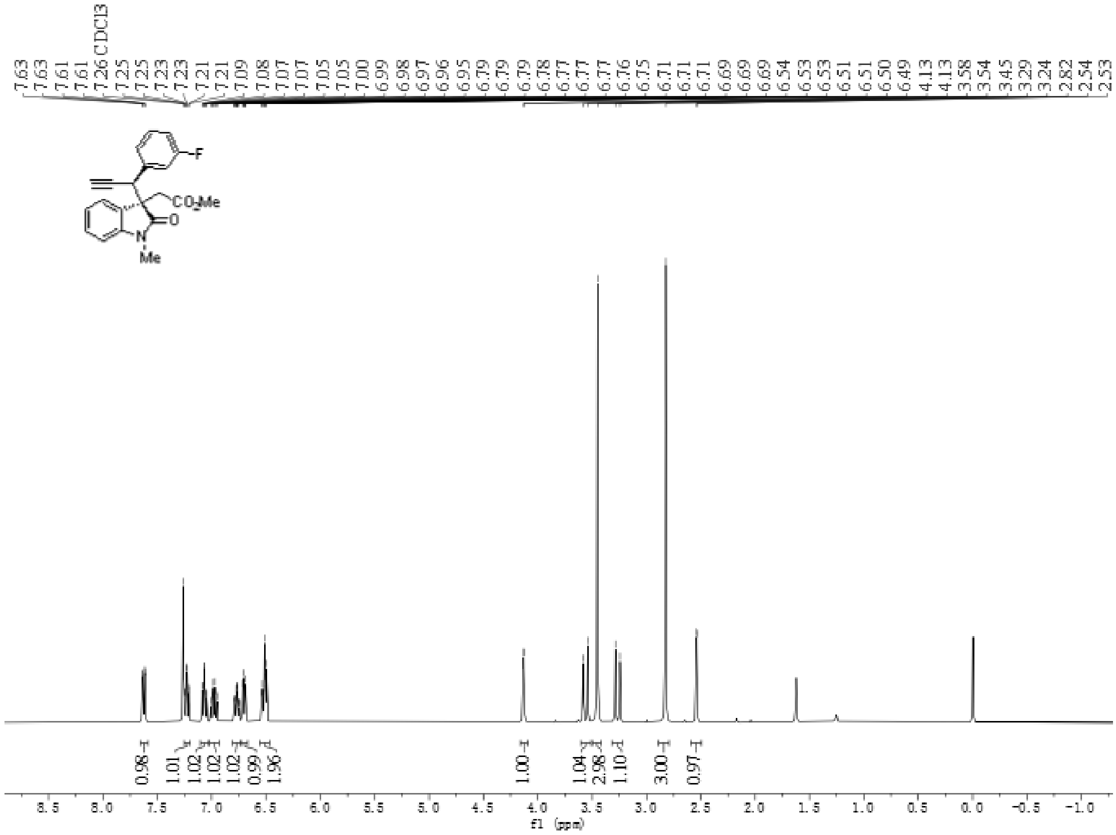

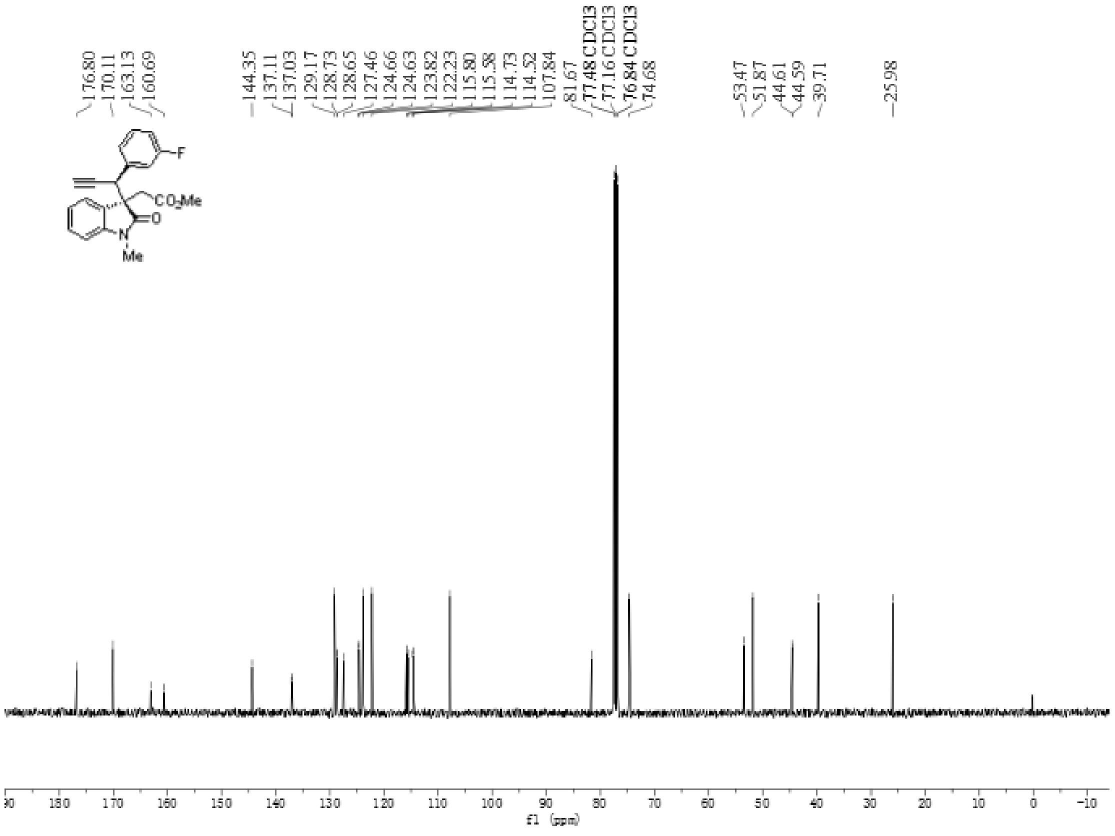

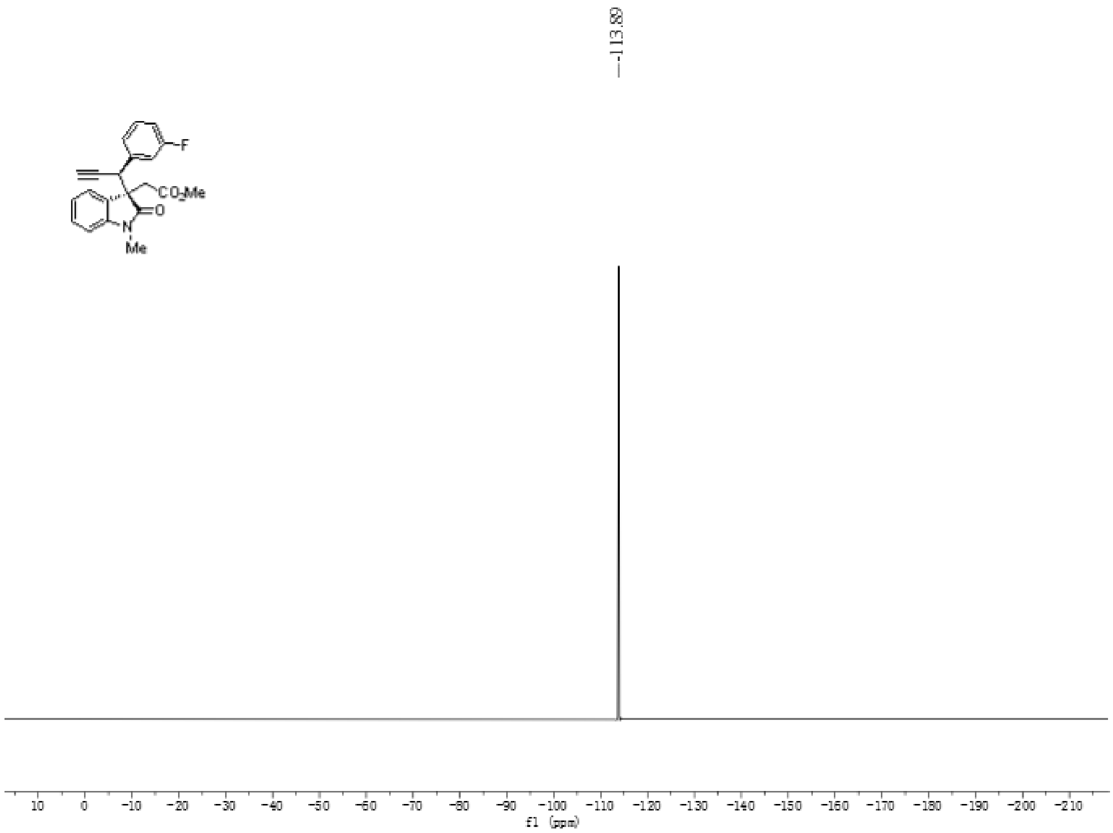

Supplementary Fig. 36. ^{1}H NMR ^{13}C NMR & ^{19}F NMR spectra of compound (*R*,*S*)-**3ag**

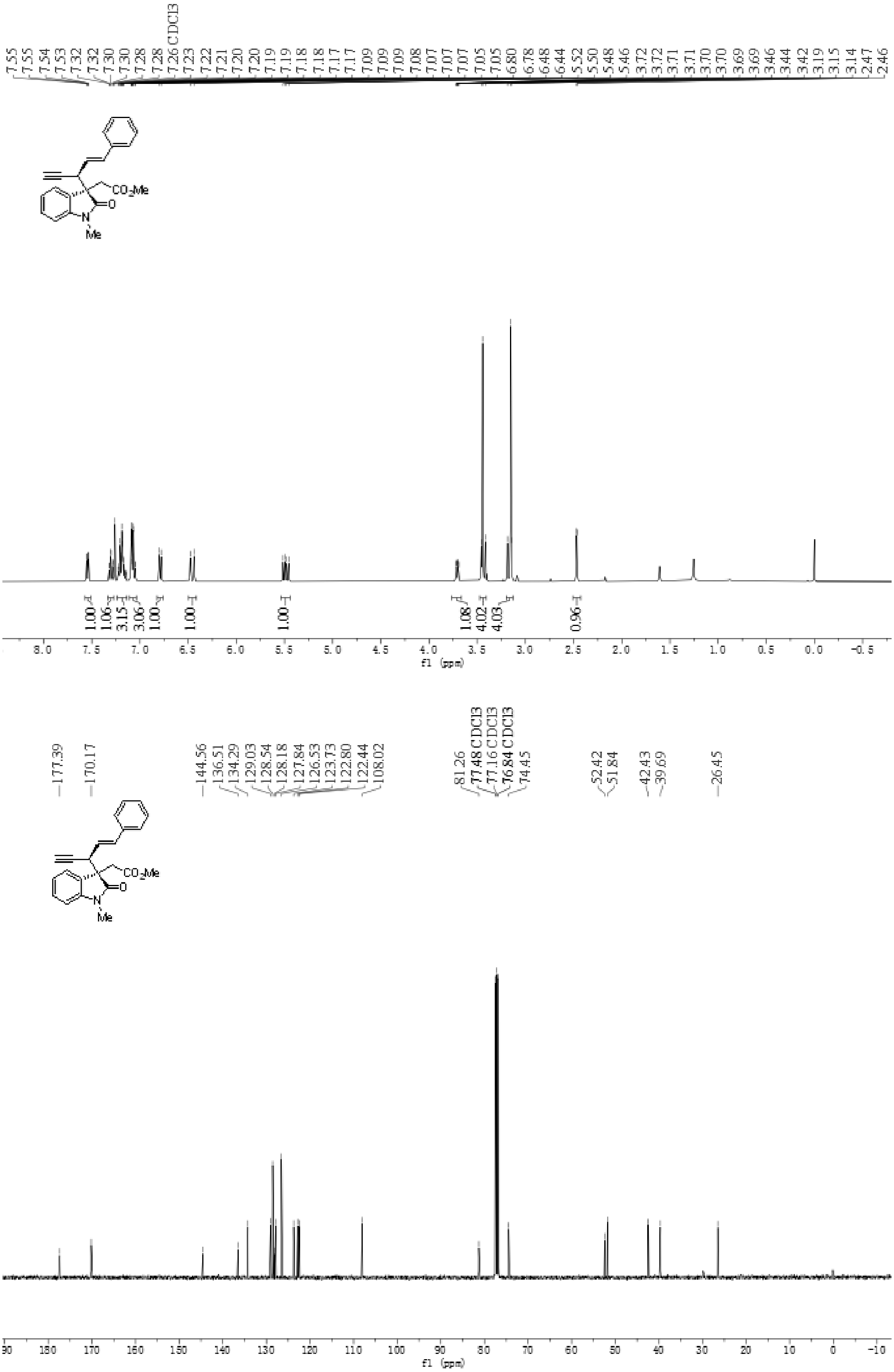

Supplementary Fig. 37. ^{1}H NMR & ^{13}C NMR spectra of compound (*R*,*R*)-**3ao**

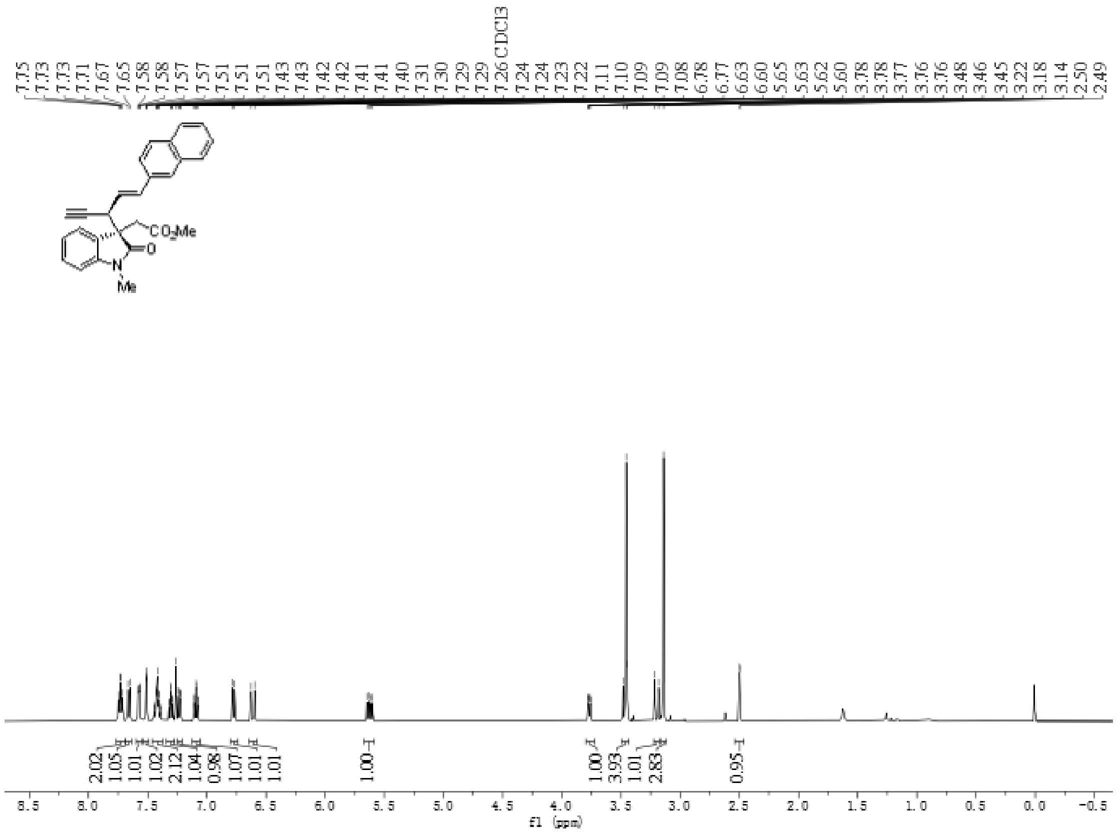

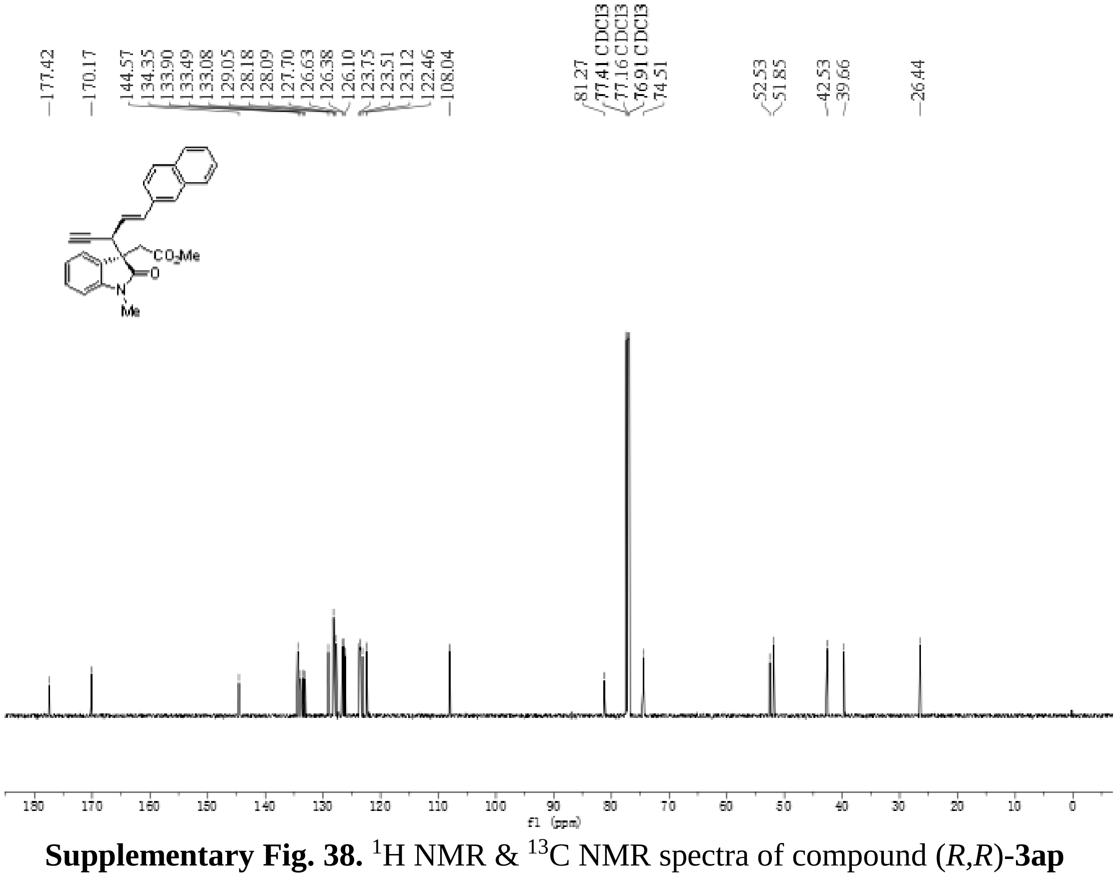

Supplementary Fig. 38. ^{1}H NMR & ^{13}C NMR spectra of compound (*R*,*R*)-**3ap**

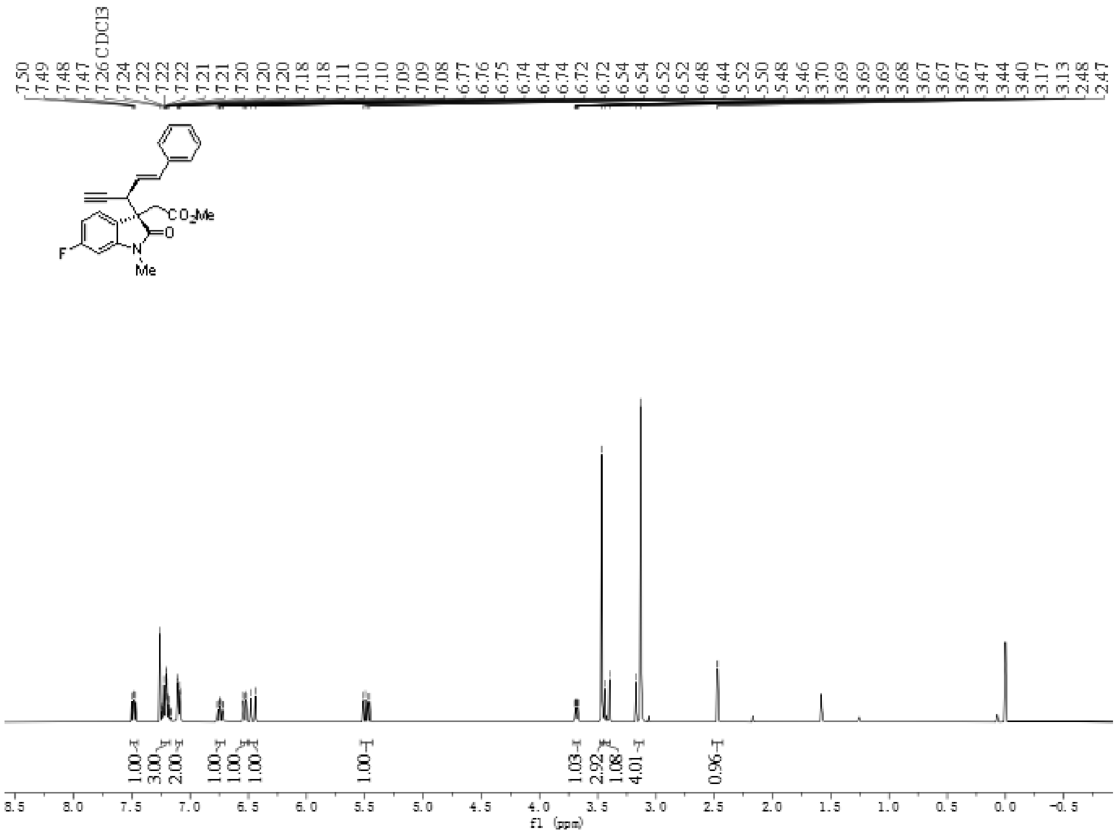
7.50
7.49
7.48
7.47
7.26 CDCl3
7.24
7.22
7.22
7.22
7.21
7.21
7.20
7.20
7.20
7.18
7.18
7.11
7.10
7.10
7.09
7.09
7.08
6.77
6.76
6.75
6.74
6.74
6.74
6.72
6.72
6.54
6.54
6.52
6.52
6.48
6.44
5.52
5.50
5.48
5.46
3.70
3.69
3.69
3.69
3.68
3.67
3.67
3.67
3.47
3.44
3.40
3.17
3.13
2.48
2.47
CO2Me
O
F
N
Me
1.00
3.00
2.00
1.00
1.00
1.00
1.00
1.03
2.92
1.08
4.01
0.96
f1 (ppm)

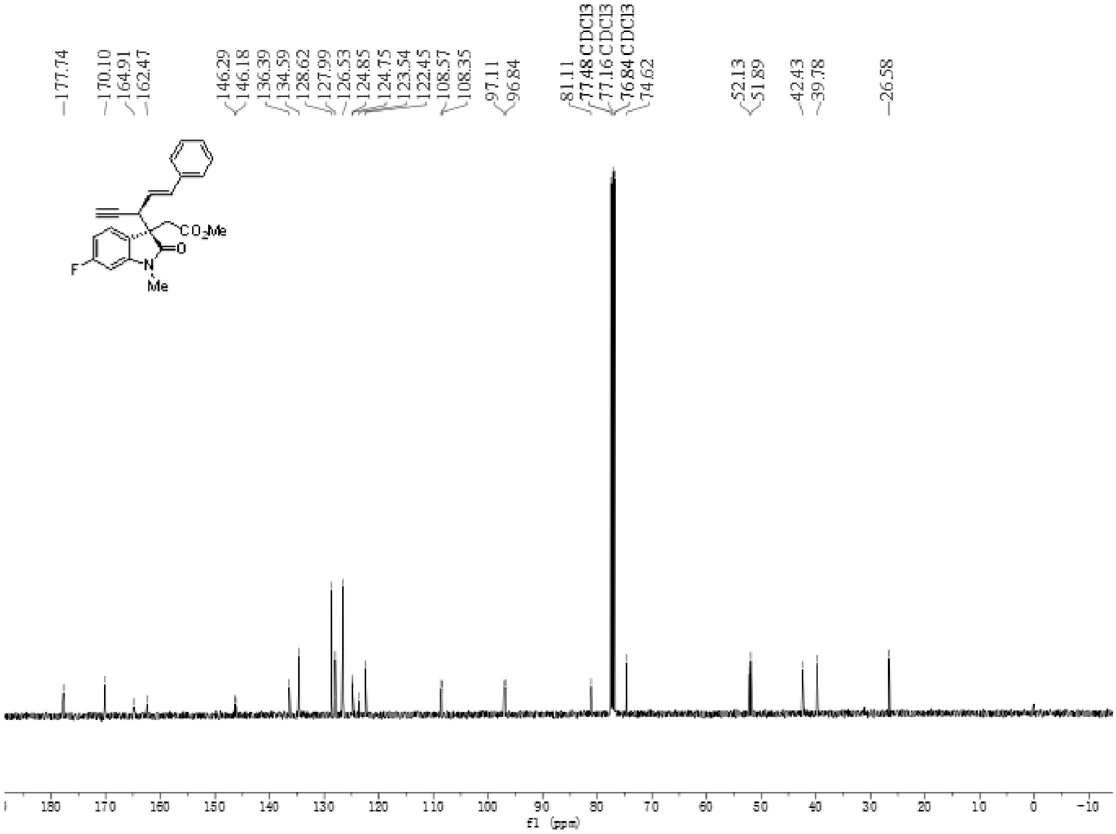
177.74
170.10
164.91
162.47
146.29
146.18
136.39
134.59
128.62
127.99
126.53
124.85
124.75
123.54
122.45
108.57
108.35
97.11
96.84
81.11
77.48 CDCl3
77.16 CDCl3
76.84 CDCl3
74.62
52.13
51.89
42.43
39.78
26.58
CO2Me
O
F
N
Me
f1 (ppm)

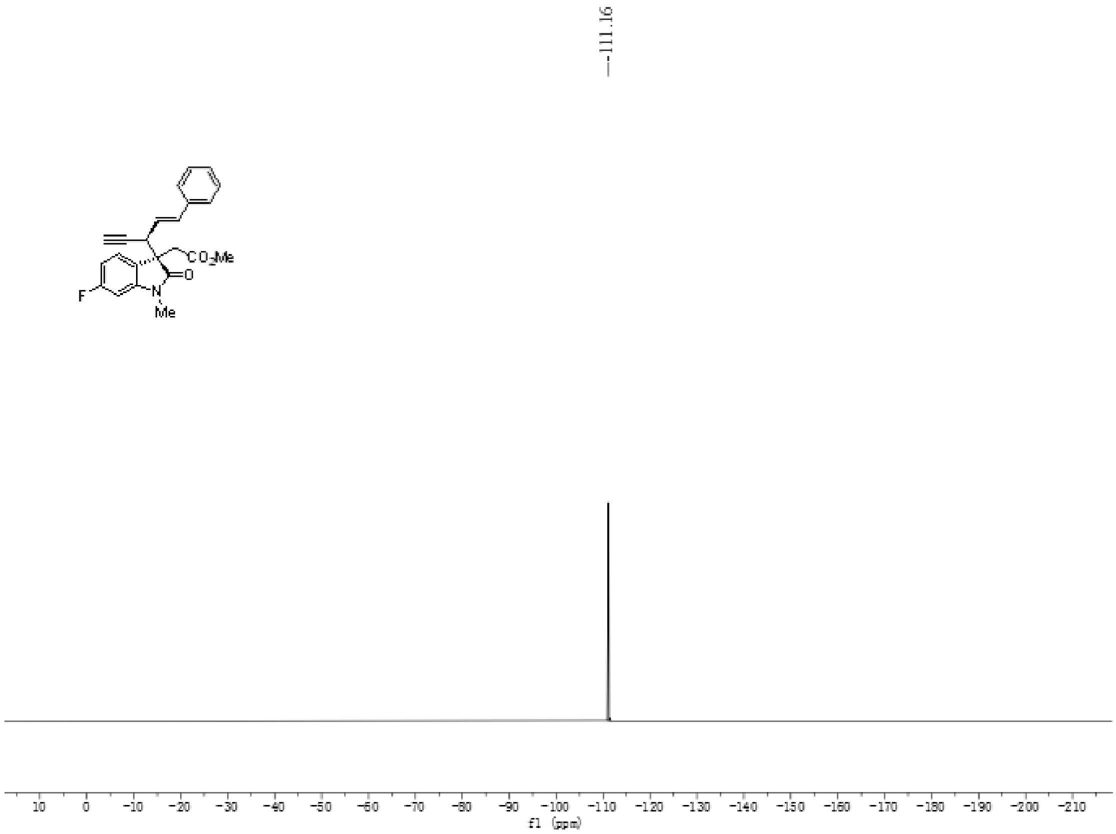

Supplementary Fig. 39. ^{1}H NMR ^{13}C NMR & ^{19}F NMR spectra of compound (*R*,*R*)-**3ko**

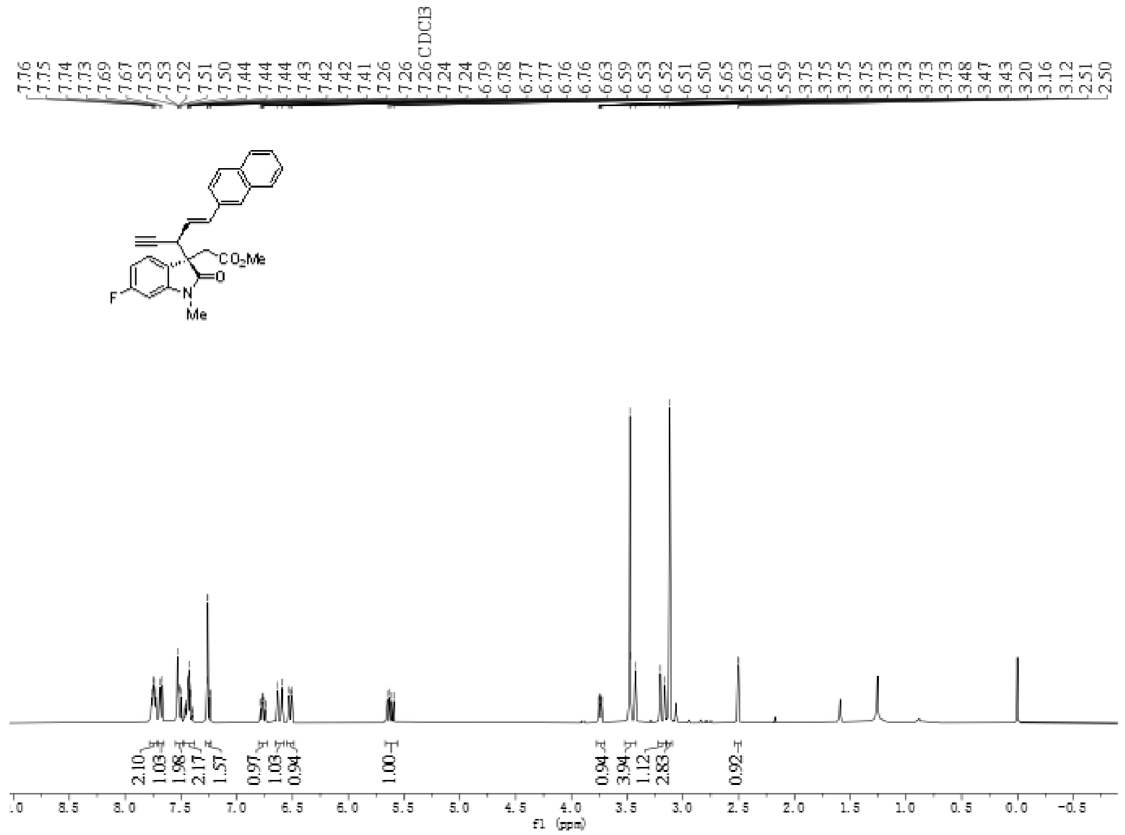
7.76 7.75 7.74 7.73 7.69 7.67 7.53 7.53 7.52 7.51 7.50 7.44 7.44 7.44 7.43 7.42 7.42 7.41 7.26 7.26 7.26 CDCl3 7.24 7.24 6.79 6.78 6.77 6.77 6.76 6.76 6.63 6.59 6.53 6.52 6.51 6.50 5.65 5.63 5.61 5.59 3.75 3.75 3.75 3.75 3.73 3.73 3.73 3.73 3.48 3.47 3.43 3.20 3.16 3.12 2.51 2.50
CO2Me
O
N
Me
F
2.10 1.03 1.98 2.17 1.57 0.97 1.03 0.94 1.00 0.94 3.94 1.12 2.83 0.92
f1 (ppm)

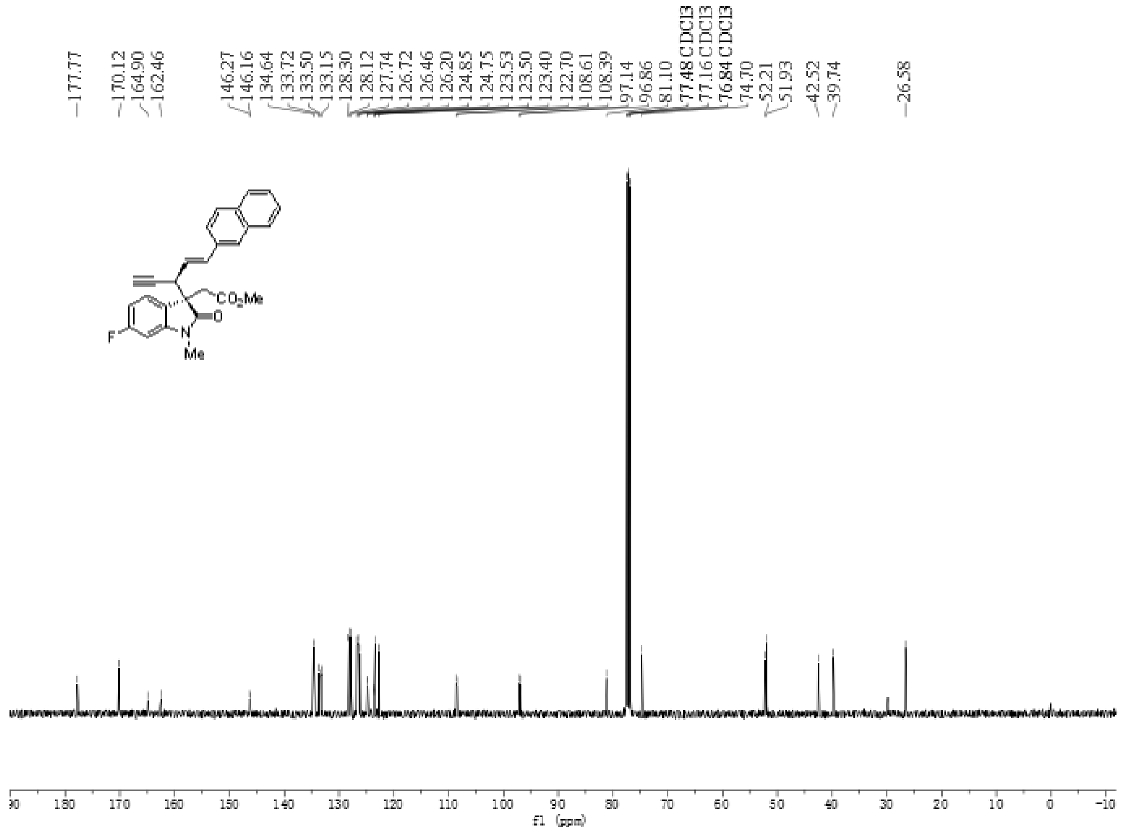
177.77 170.12 164.90 162.46 146.27 146.16 134.64 133.72 133.50 133.15 128.30 128.12 127.74 126.72 126.46 126.20 124.85 124.75 123.53 123.50 123.40 122.70 108.61 108.39 97.14 96.86 81.10 77.48 CDCl3 77.16 CDCl3 76.84 CDCl3 74.70 52.21 51.93 42.52 39.74 26.58
CO2Me
O
N
Me
F
f1 (ppm)

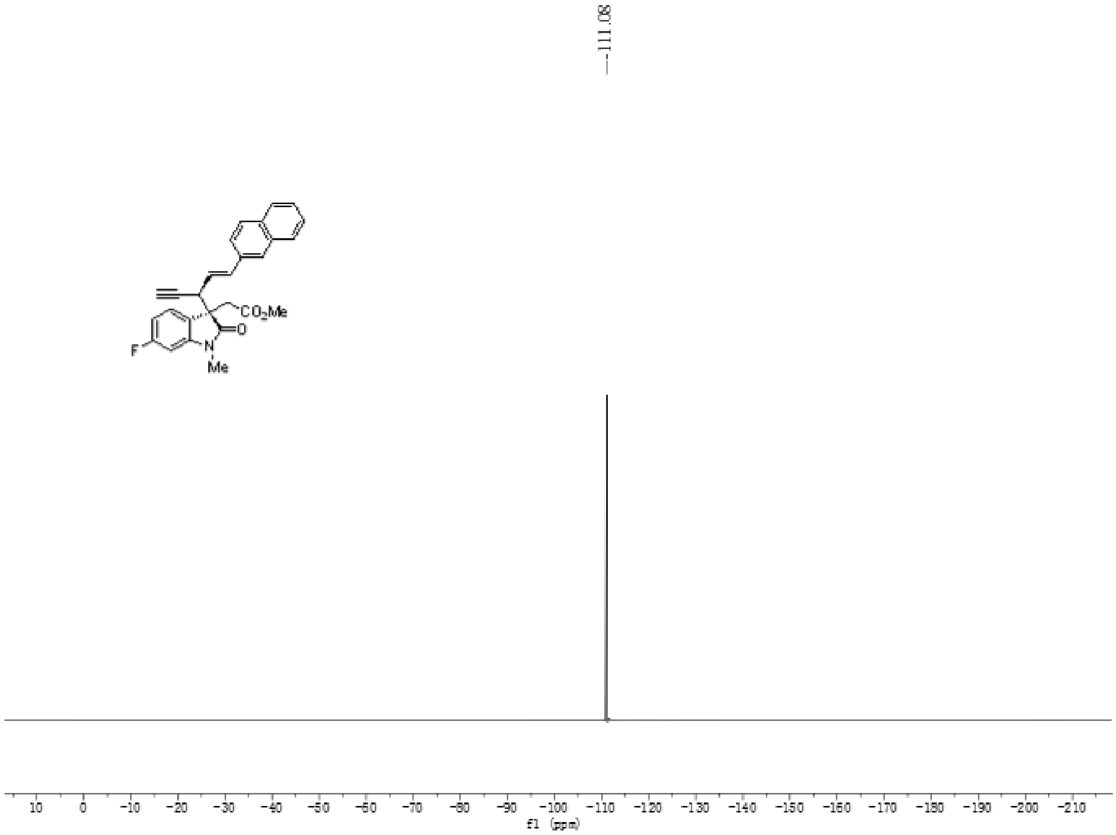

Supplementary Fig. 40. ^{1}H NMR ^{13}C NMR & ^{19}F NMR spectra of compound (*R*,*R*)-**3kp**

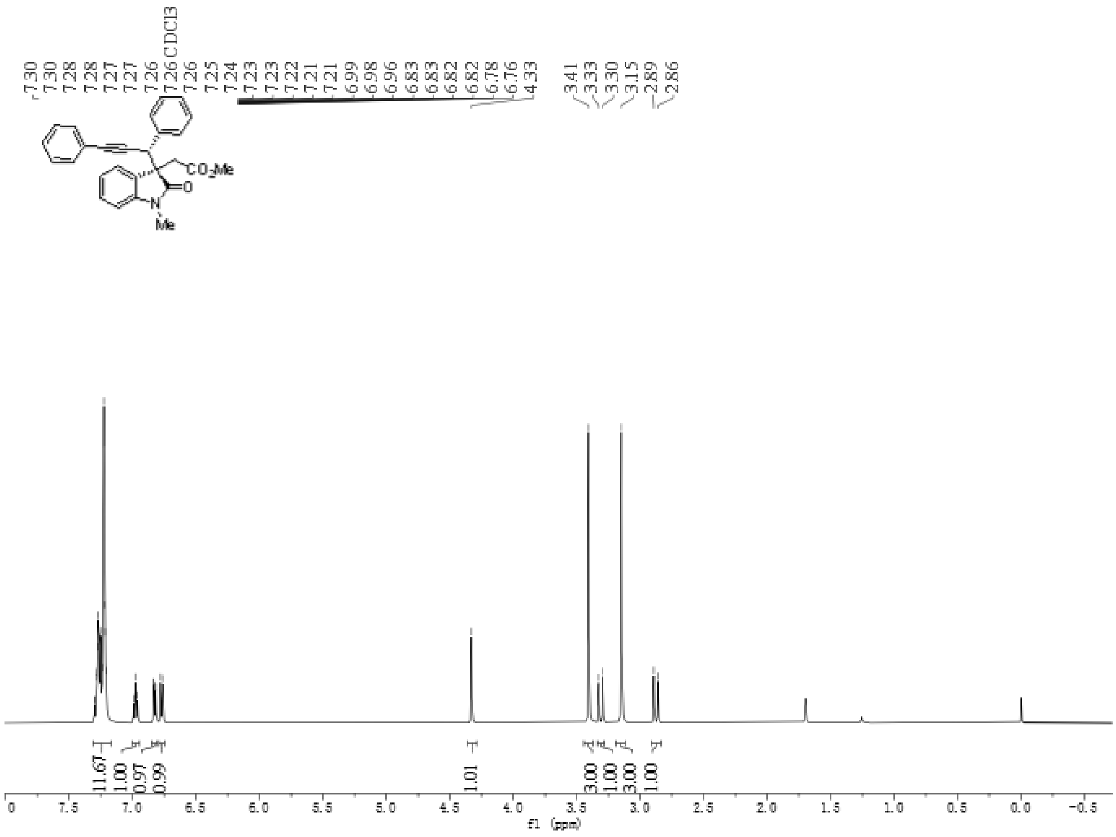

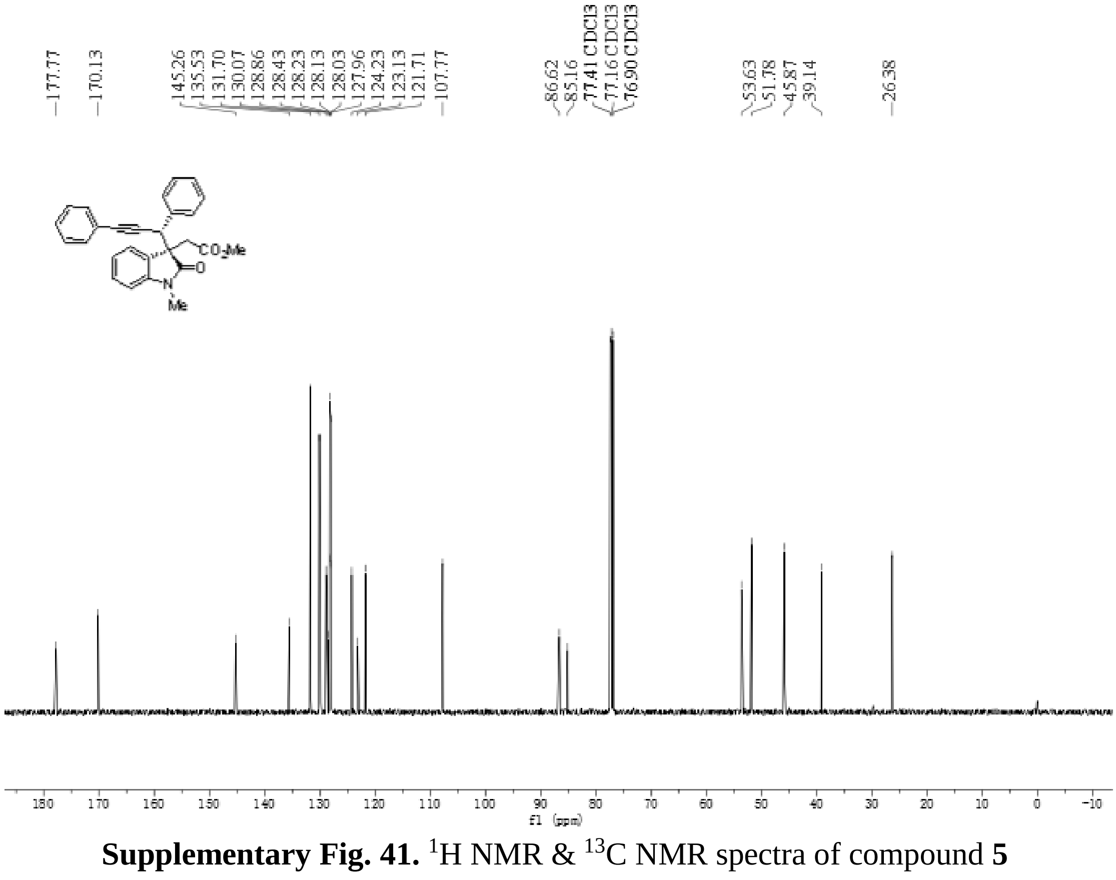

Supplementary Fig. 41. ^{1}H NMR & ^{13}C NMR spectra of compound **5**

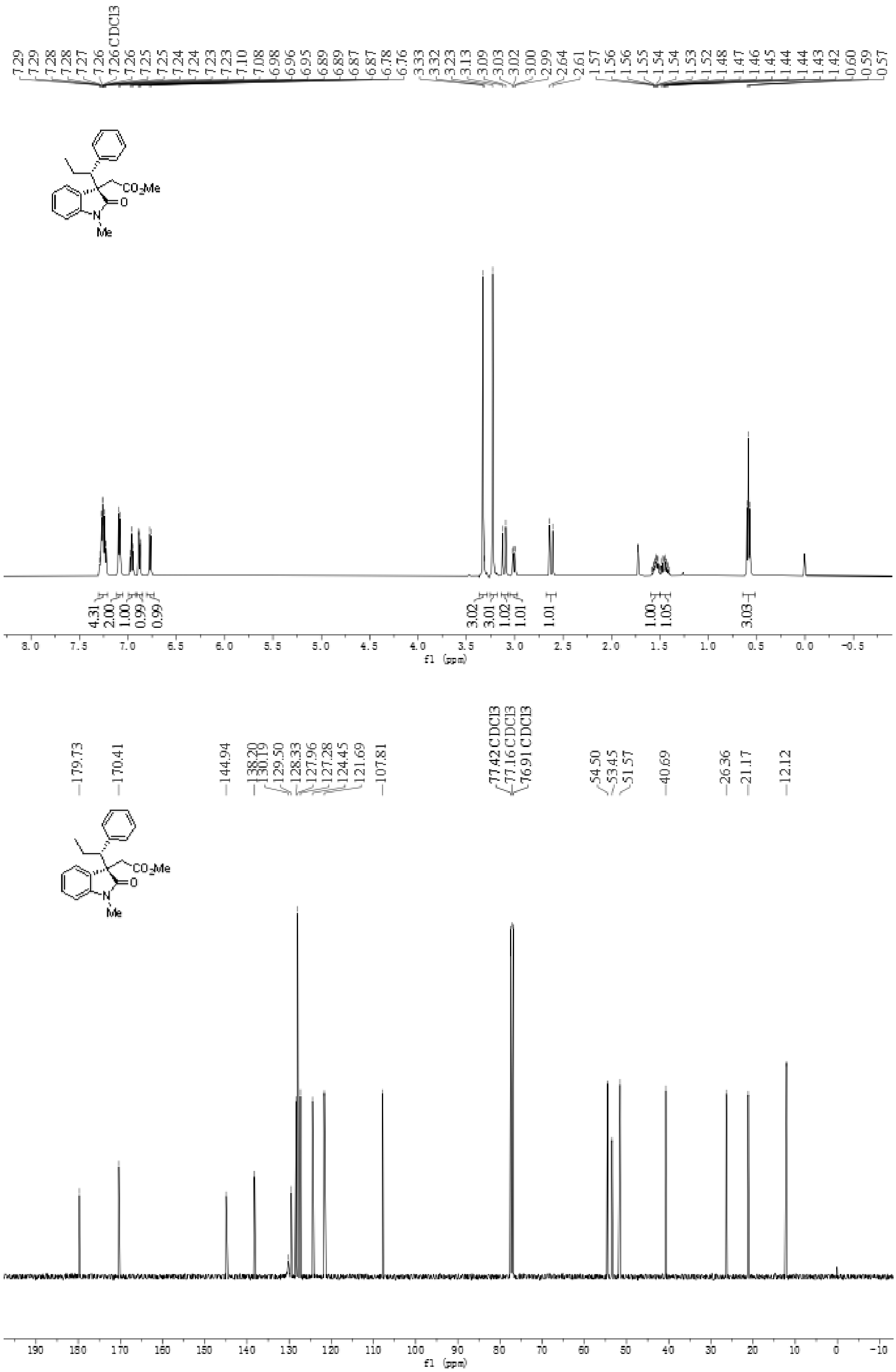

Supplementary Fig. 42. ^{1}H NMR & ^{13}C NMR spectra of compound **6**

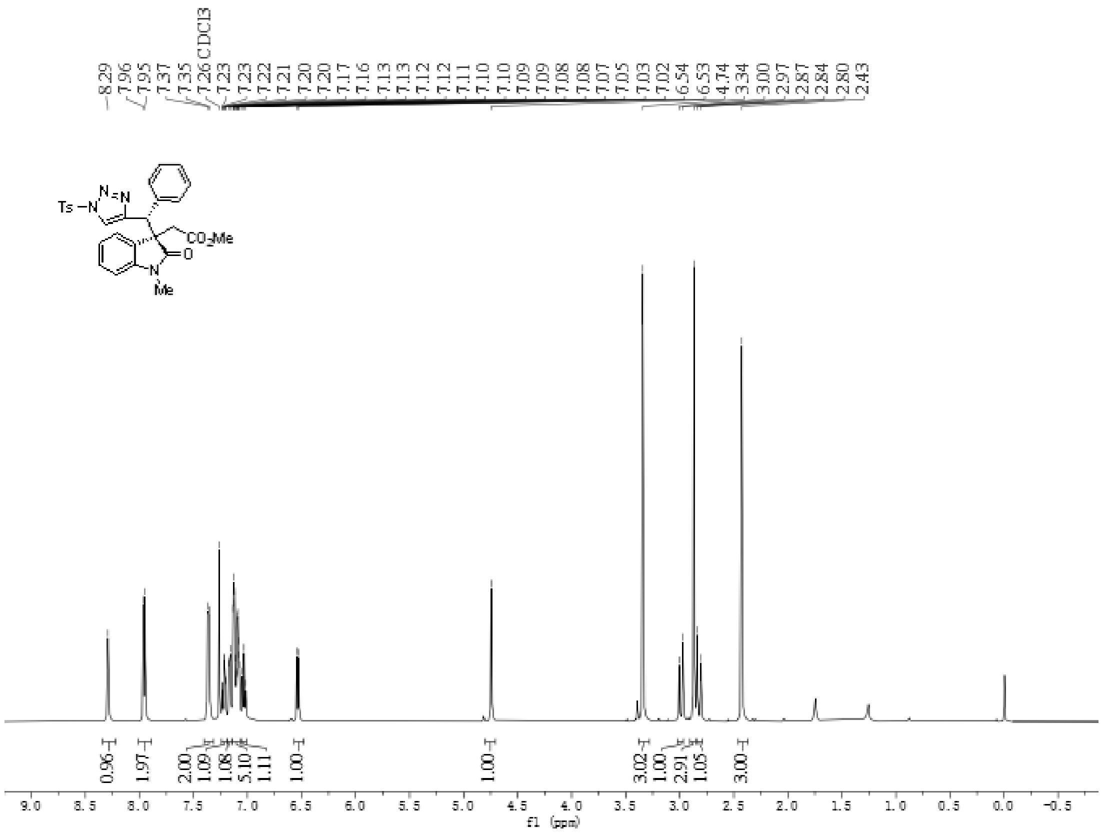

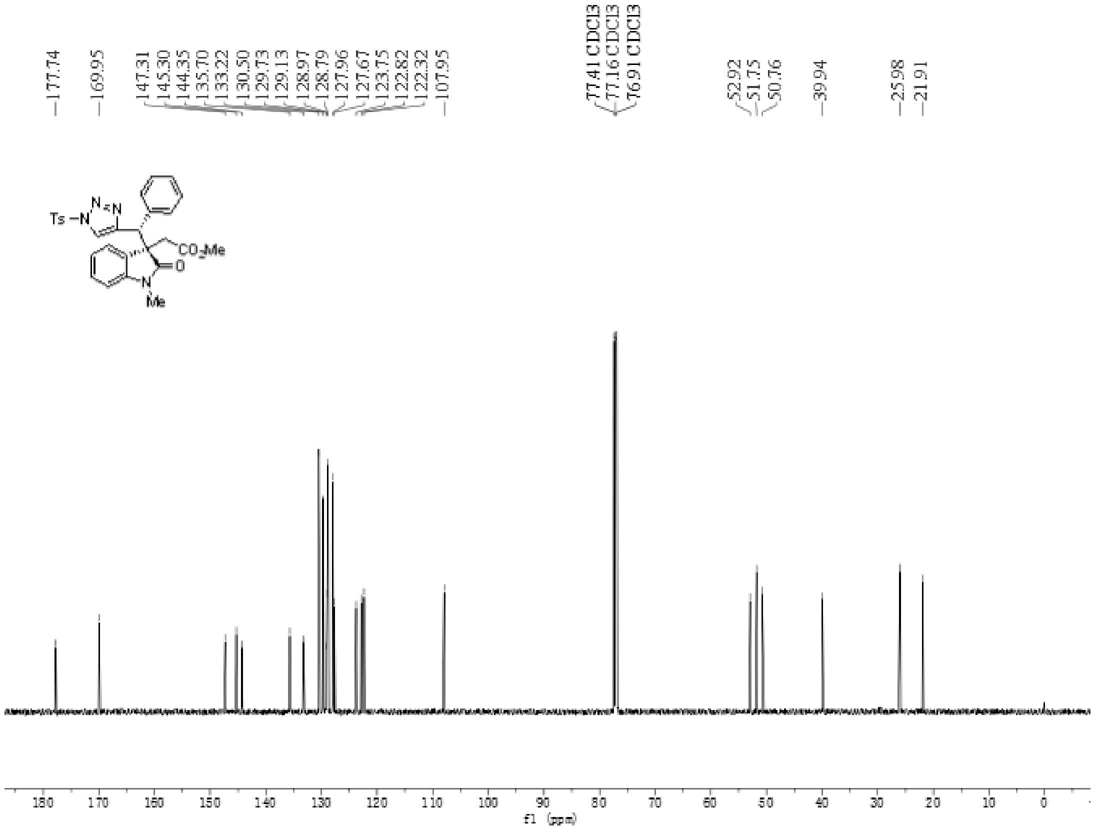

Supplementary Fig. 43. ^{1}H NMR & ^{13}C NMR spectra of compound **7**

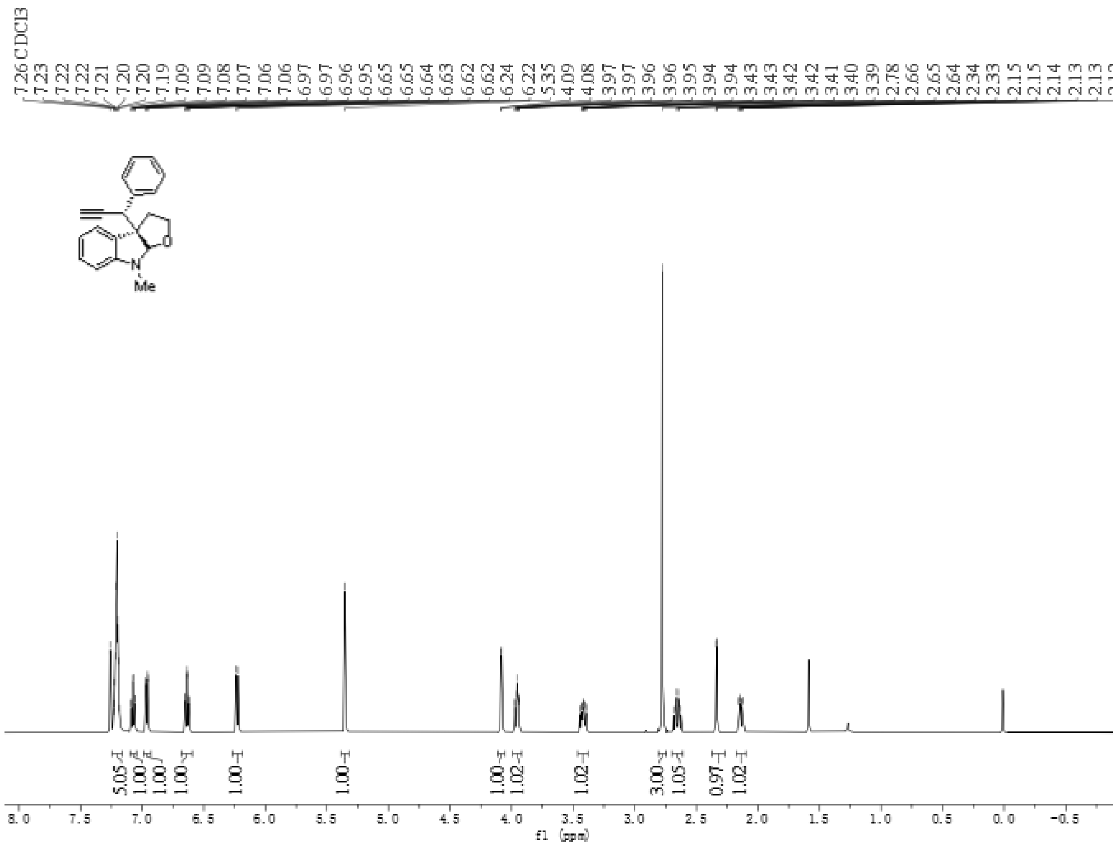

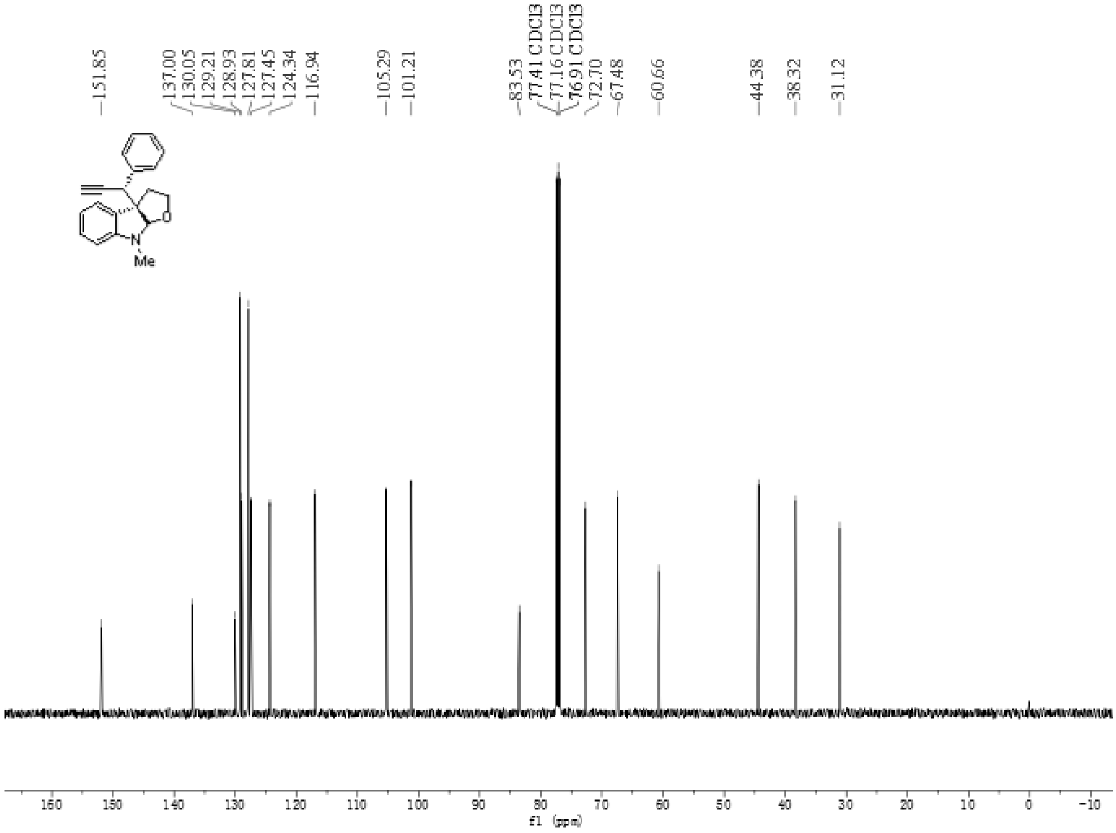

Supplementary Fig. 44. ^{1}H NMR & ^{13}C NMR spectra of compound **8**

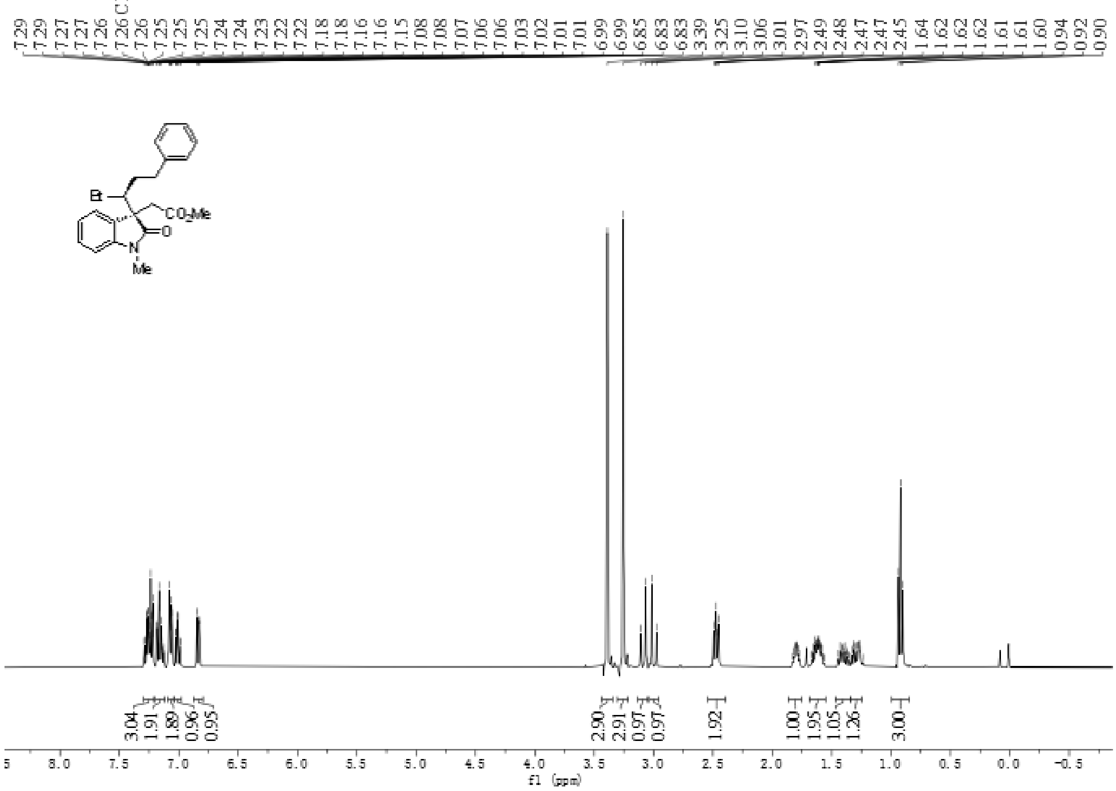

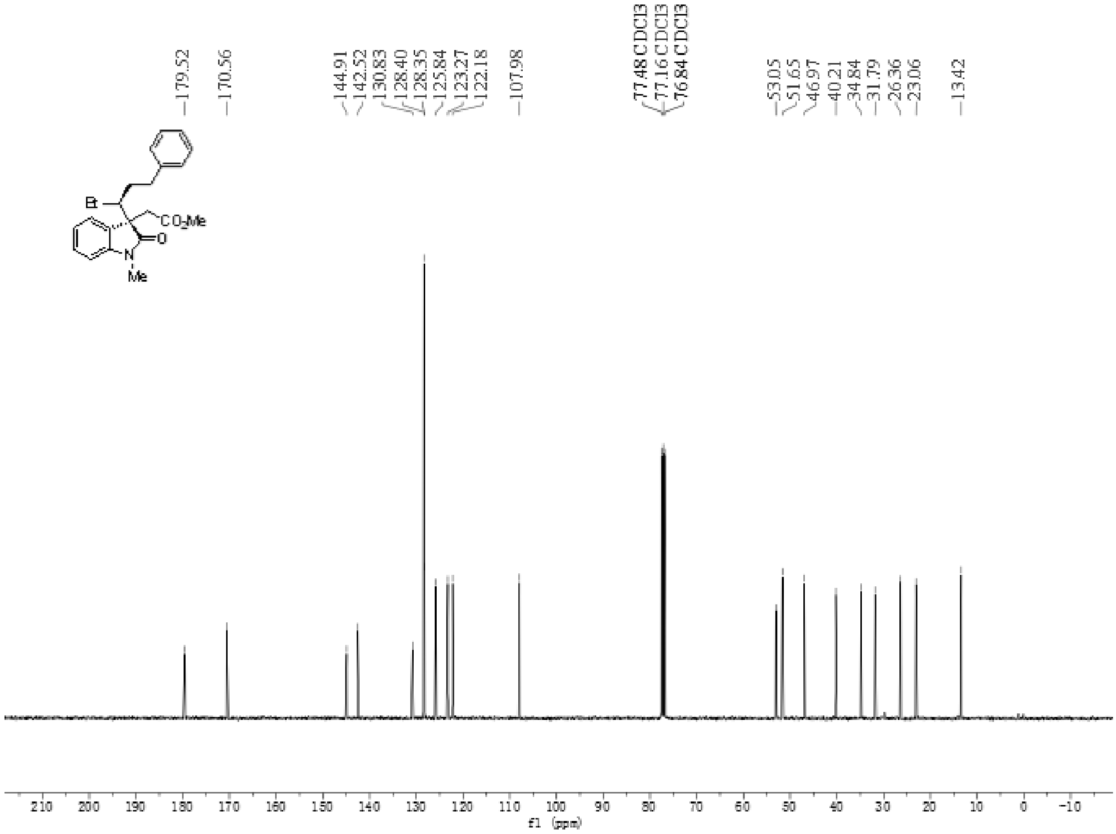

Supplementary Fig. 45. ^{1}H NMR & ^{13}C NMR spectra of compound **9**

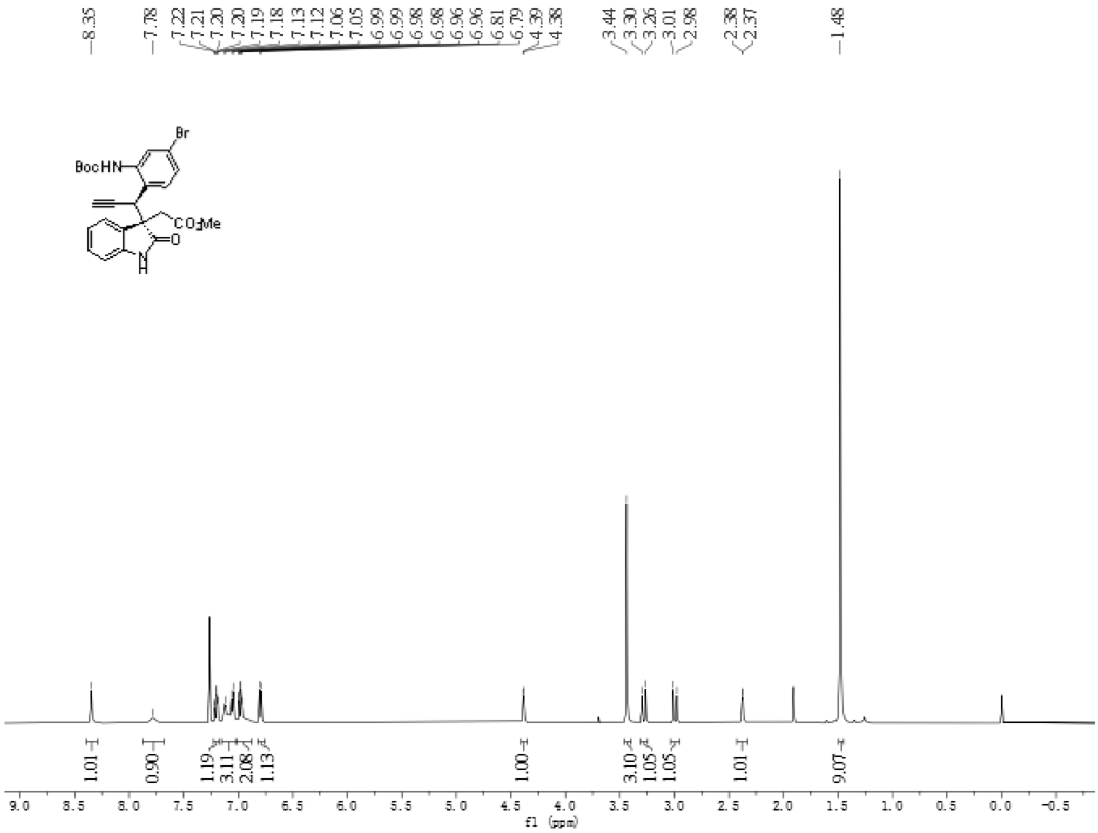

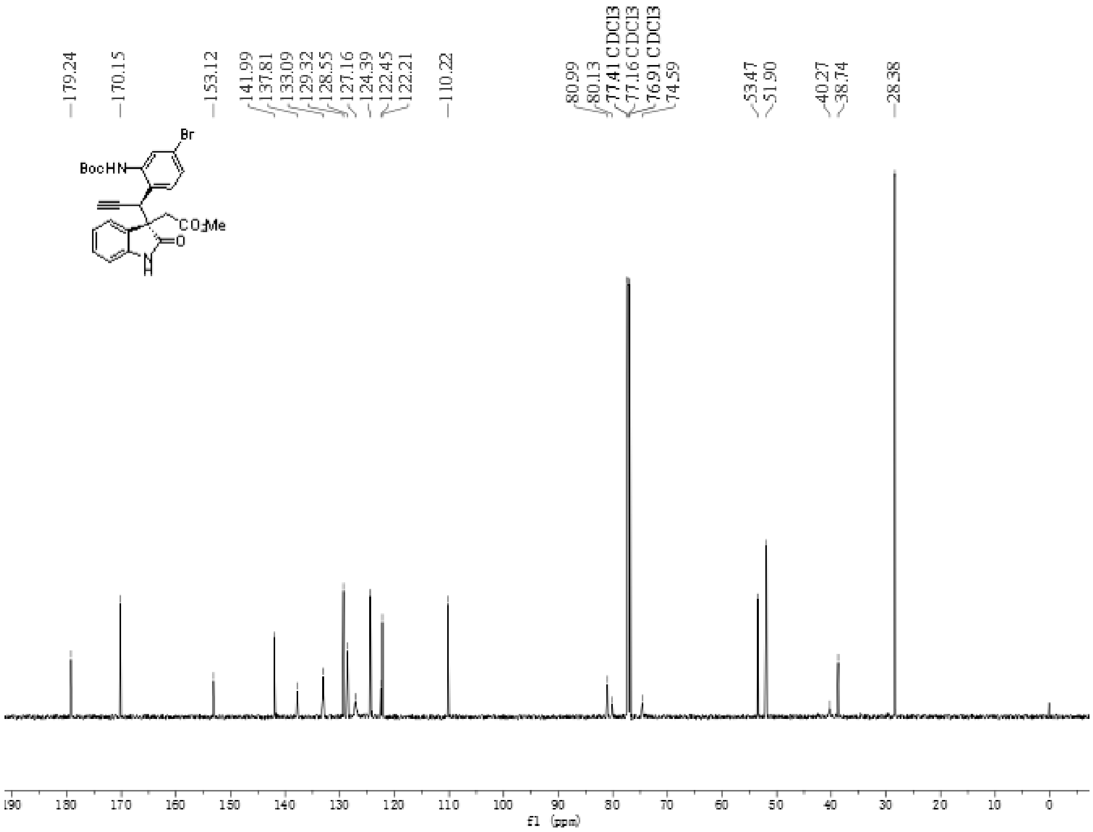

Supplementary Fig. 46. ^{1}H NMR & ^{13}C NMR spectra of compound **3nq**

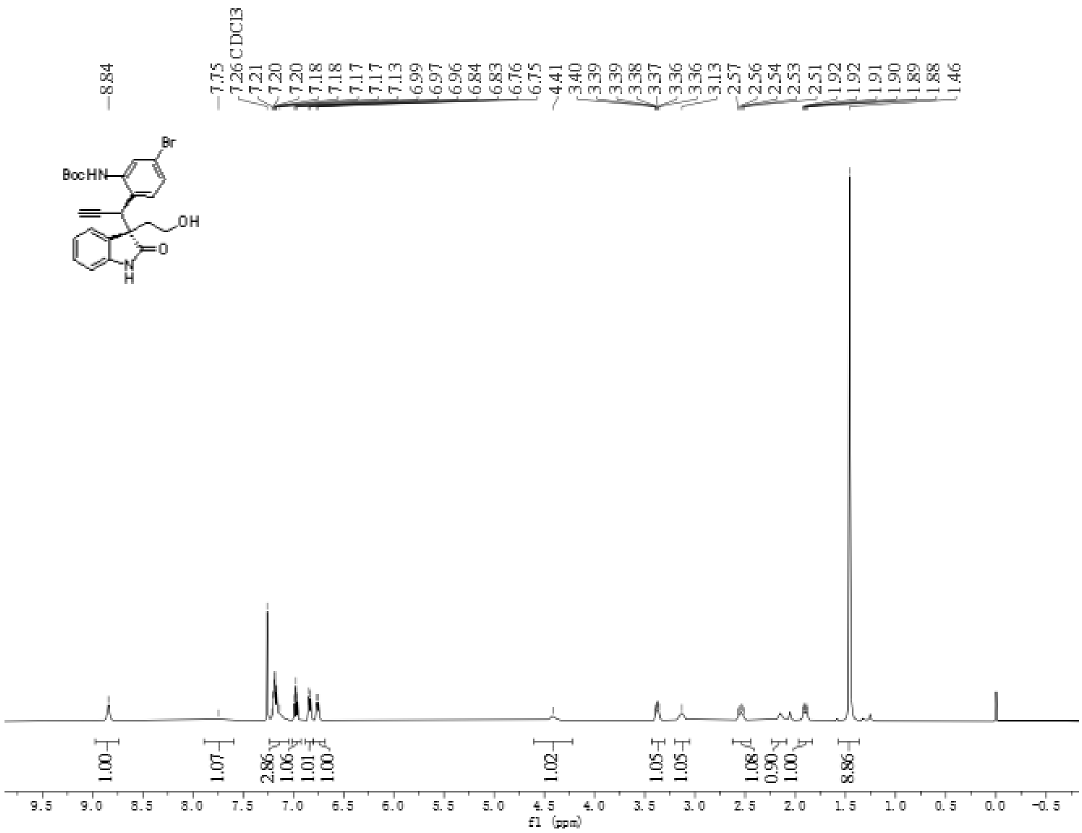

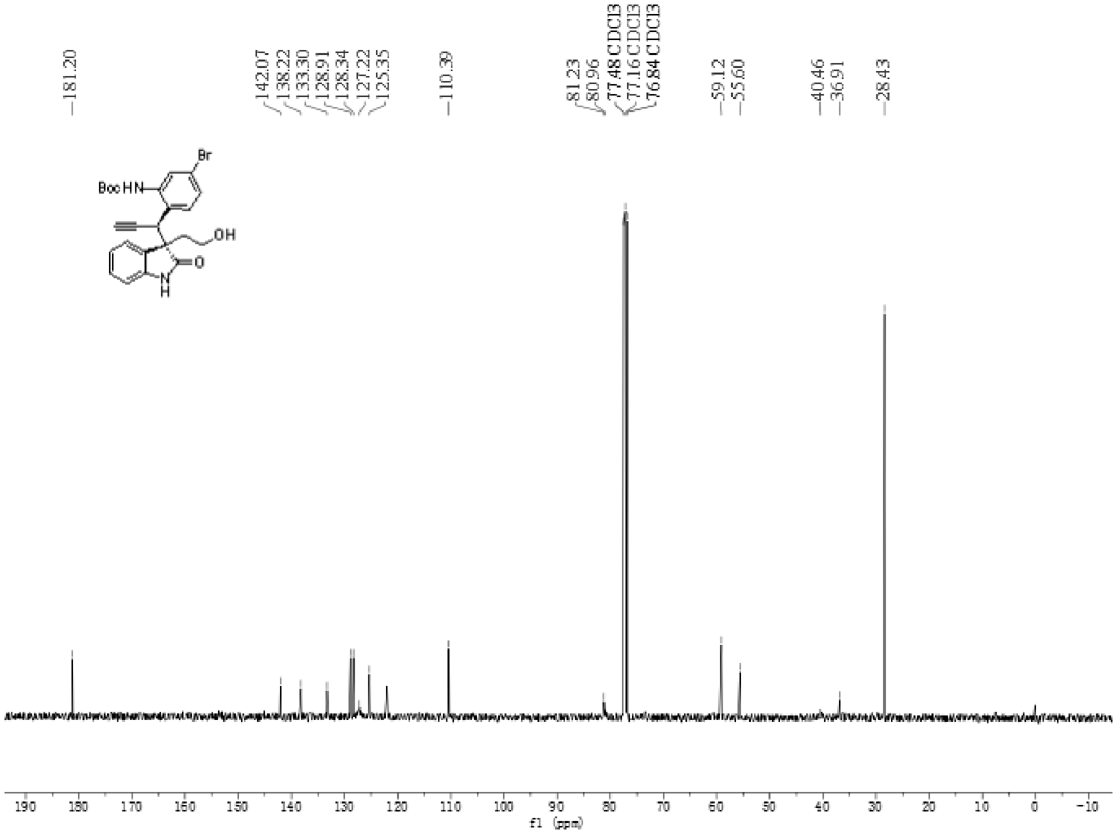

Supplementary Fig. 47. ^{1}H NMR & ^{13}C NMR spectra of compound **10**

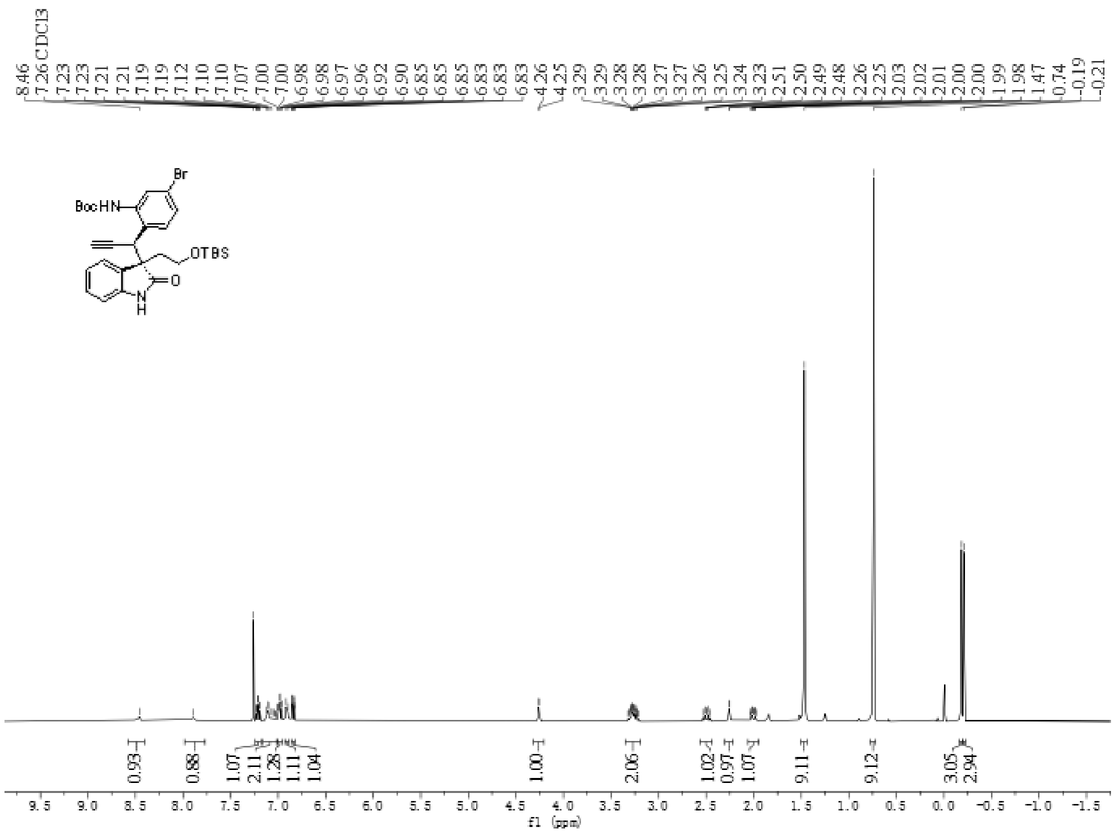

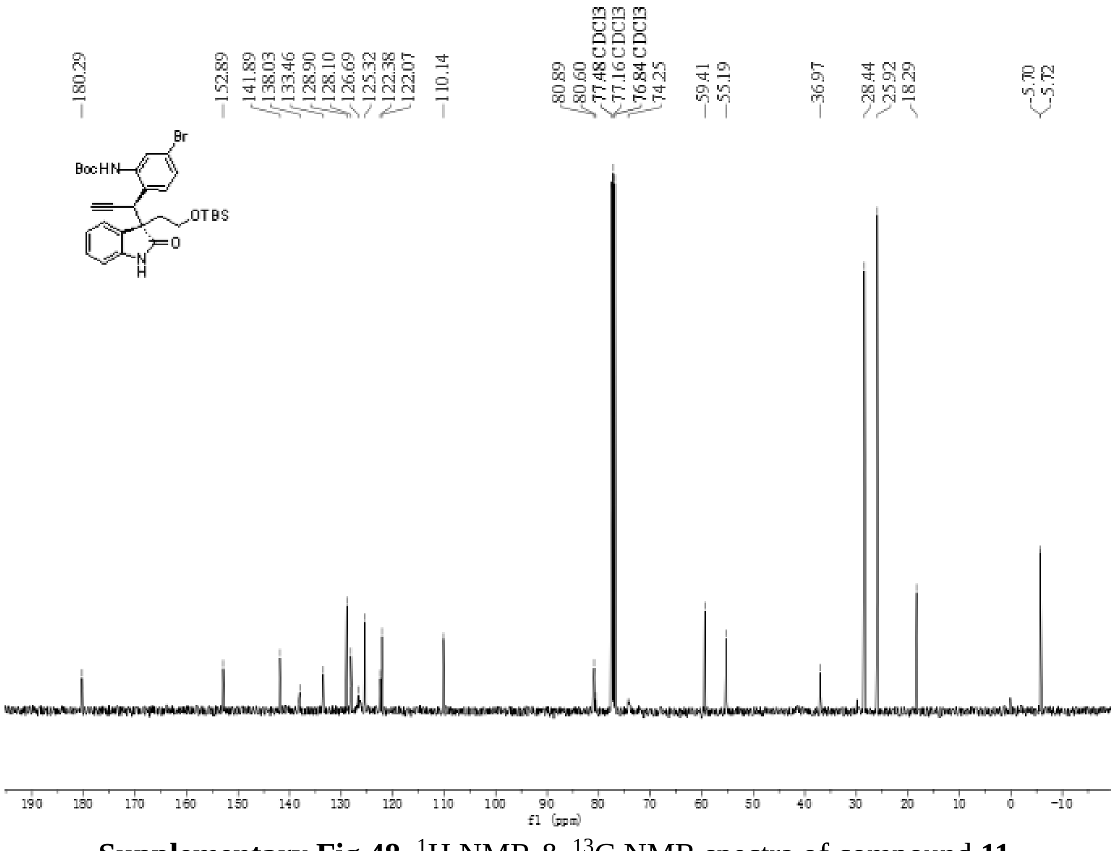

Supplementary Fig.48. ^{1}H NMR & ^{13}C NMR spectra of compound **11**

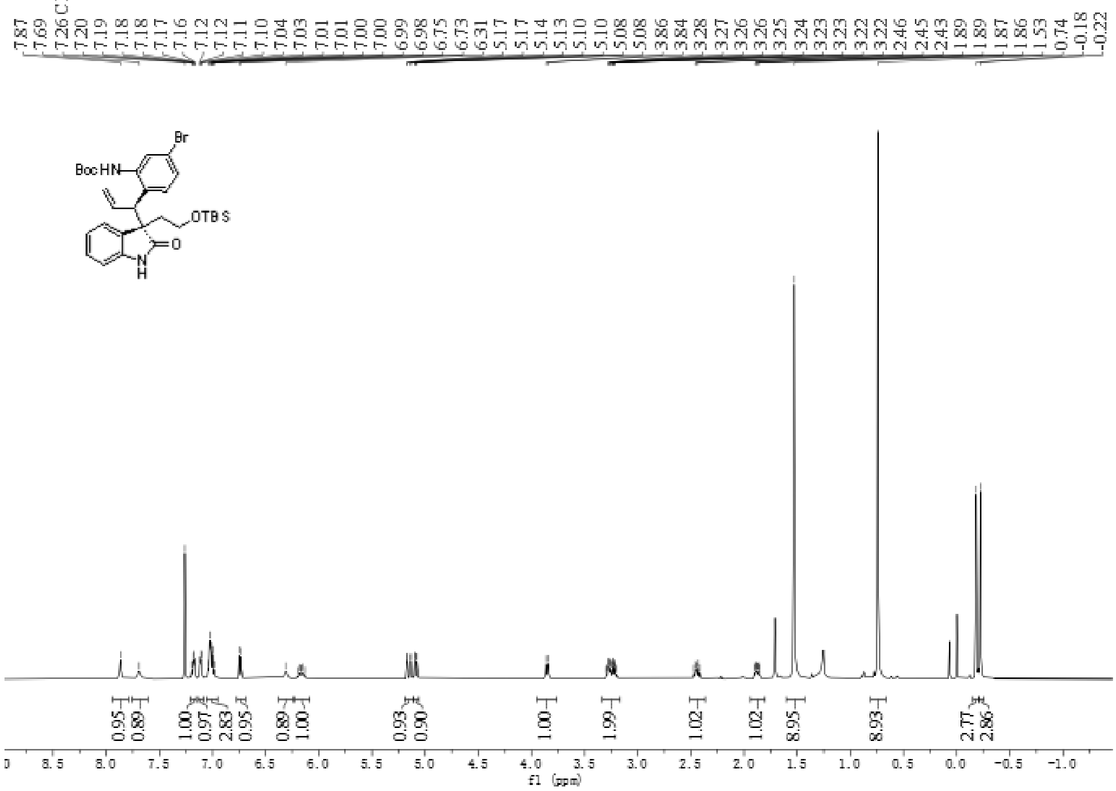

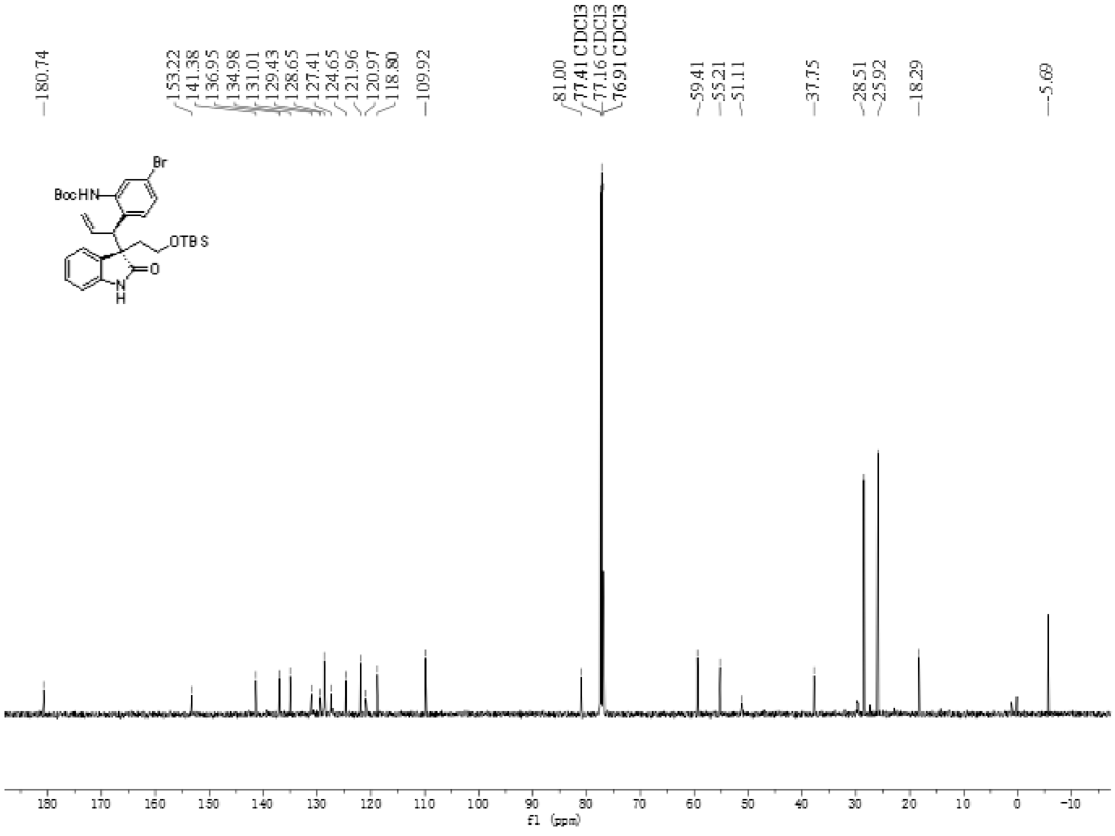

Supplementary Fig. 49. ^{1}H NMR & ^{13}C NMR spectra of compound **12**

13. HPLC of Products

SAMPLE INFORMATION

Sample Name:	wyh-1-167-1-rac-IG-30%-1ml/min	Acquired By:	System
Sample Type:	Unknown	Sample Set Name	wyh16713
Vial:	51	Acq. Method Set:	30% 1ml
Injection #:	1	Processing Method	1
Injection Volume:	10.00 ul	Channel Name:	230.0nm
Run Time:	24.0 Minutes	Proc. Chnl. Descr.:	2998 PDA 230.0 nm (2998
Date Acquired:	7/13/2021 5:55:33 PM CST		
Date Processed:	7/13/2021 7:36:08 PM CST		

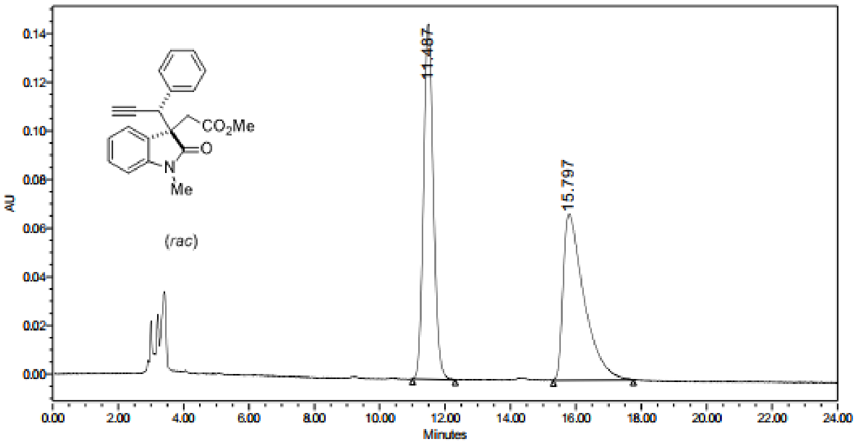

	RT	Area	% Area	Height
1	11.487	3089227	50.37	145990
2	15.797	3043361	49.63	68143

SAMPLE INFORMATION

Sample Name:	wyh-1-75-1-asy-IG-30%-1ml/min	Acquired By:	System
Sample Type:	Unknown	Sample Set Name:	wyh1751
Vial:	32	Acq. Method Set:	30% 1ml
Injection #:	1	Processing Method:	1
Injection Volume:	10.00 ul	Channel Name:	230.0nm
Run Time:	20.0 Minutes	Proc. Chnl. Descr.:	2998 PDA 230.0 nm (2998
Date Acquired:	1/5/2021 4:02:00 PM CST		
Date Processed:	7/14/2021 8:11:35 PM CST		

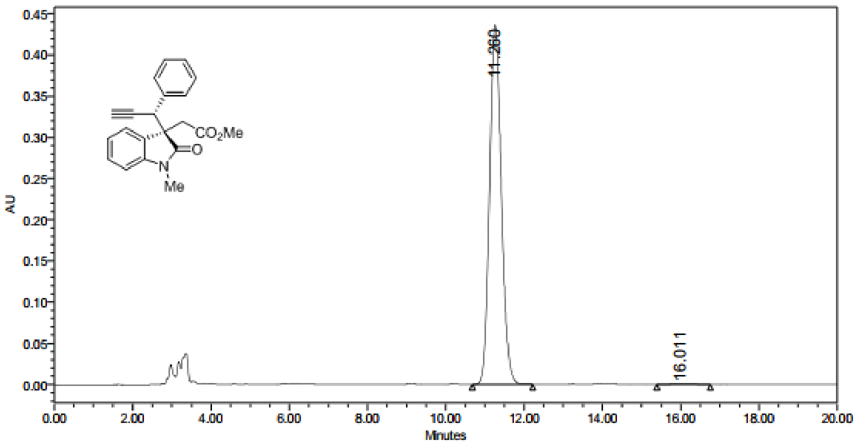

	RT	Area	% Area	Height
1	11.260	9107741	99.75	436059
2	16.011	22534	0.25	553

Supplementary Fig. 50. HPLC spectra of compound **(*R*,*R*)-3aa**

SAMPLE INFORMATION

Sample Name:	wyh-1-167-1-rac-IG-30%-1ml/min	Acquired By:	System
Sample Type:	Unknown	Sample Set Name	wyh16713
Vial:	51	Acq. Method Set:	30% 1ml
Injection #:	1	Processing Method	1
Injection Volume:	10.00 ul	Channel Name:	230.0nm
Run Time:	24.0 Minutes	Proc. Chnl. Descr.:	2998 PDA 230.0 nm (2998
Date Acquired:	7/13/2021 5:55:33 PM CST		
Date Processed:	7/13/2021 7:36:08 PM CST		

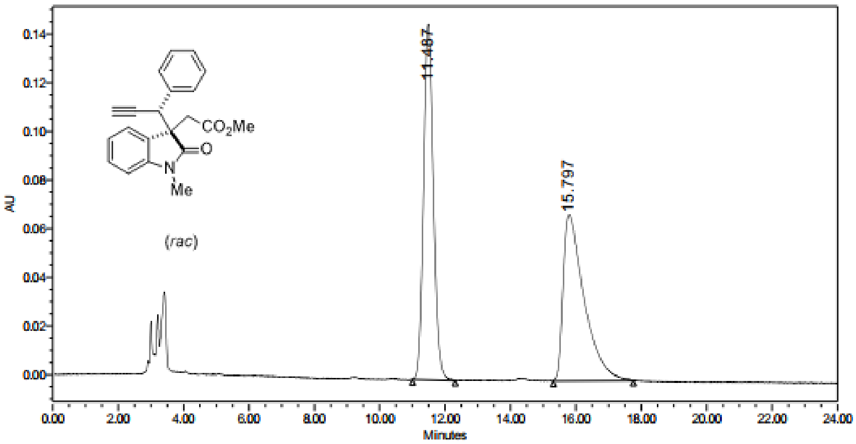

	RT	Area	% Area	Height
1	11.487	3089227	50.37	145990
2	15.797	3043361	49.63	68143

SAMPLE INFORMATION

Sample Name:		Acquired By:	System
Sample Type:	Unknown	Sample Set Name	wyh16713
Vial:	53	Acq. Method Set:	30% 1ml
Injection #:	1	Processing Method	1
Injection Volume:	10.00 ul	Channel Name:	230.0nm
Run Time:	24.0 Minutes	Proc. Chnl. Descr.:	2998 PDA 230.0 nm (2998
Date Acquired:	7/13/2021 6:44:55 PM CST		
Date Processed:	7/13/2021 7:37:15 PM CST		

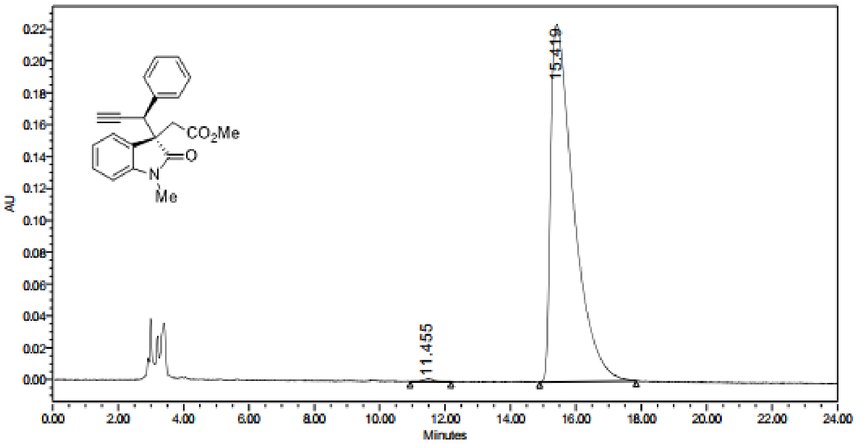

	RT	Area	% Area	Height
1	11.455	40971	0.37	1824
2	15.419	11001117	99.63	224374

Supplementary Fig. 51. HPLC spectra of compound **(*S*,*S*)-3aa**

SAMPLE INFORMATION

Sample Name:		Acquired By:	System
Sample Type:	Unknown	Sample Set Name	wyh182up
Vial:	12	Acq. Method Set:	30% 1ml
Injection #:	1	Processing Method	1
Injection Volume:	10.00 ul	Channel Name:	270.0nm
Run Time:	13.0 Minutes	Proc. Chnl. Descr.:	2998 PDA 270.0 nm (2998
Date Acquired:	3/6/2021 1:01:37 PM CST		
Date Processed:	3/6/2021 2:41:06 PM CST		

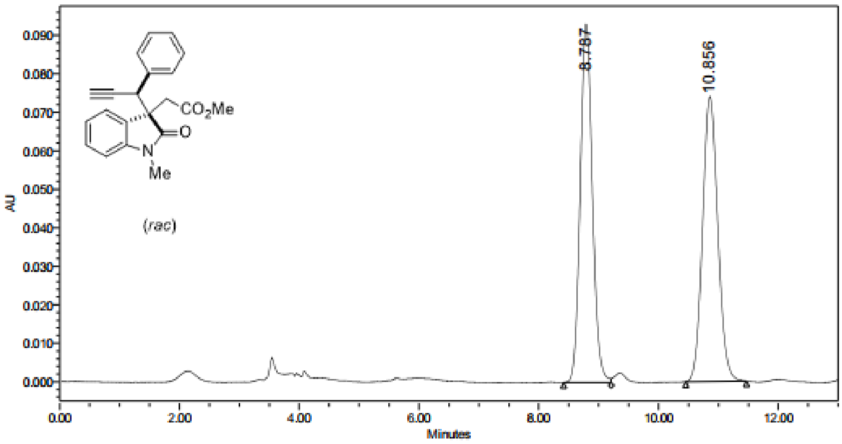

	RT	Area	% Area	Height
1	8.787	1318498	50.20	93123
2	10.856	1307970	49.80	74057

SAMPLE INFORMATION

Sample Name:	wyh-1-82-1-asy-IG-30%-1ml/min	Acquired By:	System
Sample Type:	Unknown	Sample Set Name	wyh182up
Vial:	13	Acq. Method Set:	30% 1ml
Injection #:	1	Processing Method	1
Injection Volume:	10.00 ul	Channel Name:	270.0nm
Run Time:	13.0 Minutes	Proc. Chnl. Descr.:	2998 PDA 270.0 nm (2998
Date Acquired:	3/6/2021 1:20:26 PM CST		
Date Processed:	3/6/2021 2:43:32 PM CST		

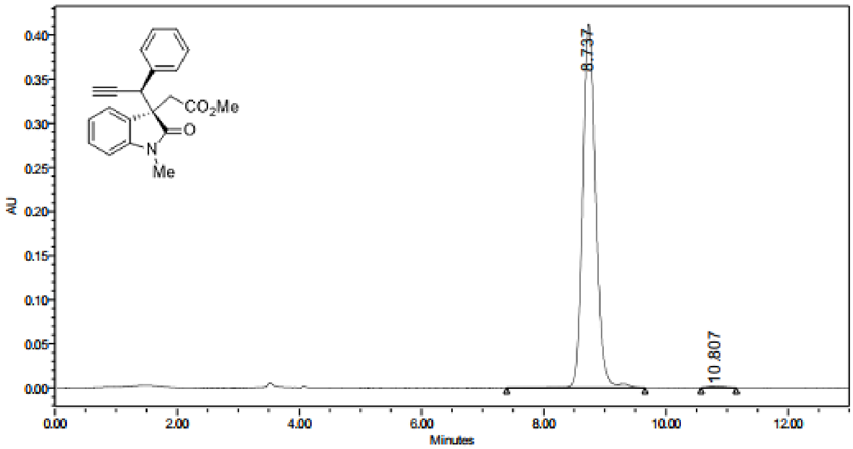

	RT	Area	% Area	Height
1	8.737	6079519	99.57	411878
2	10.807	26183	0.43	1625

Supplementary Fig. 52. HPLC spectra of compound **(*R*,*S*)-3aa**

SAMPLE INFORMATION

Sample Name:		Acquired By:	System
Sample Type:	Unknown	Sample Set Name	wyh182up
Vial:	12	Acq. Method Set:	30% 1ml
Injection #:	1	Processing Method	1
Injection Volume:	10.00 ul	Channel Name:	270.0nm
Run Time:	13.0 Minutes	Proc. Chnl. Descr.:	2998 PDA 270.0 nm (2998
Date Acquired:	3/6/2021 1:01:37 PM CST		
Date Processed:	3/6/2021 2:41:06 PM CST		

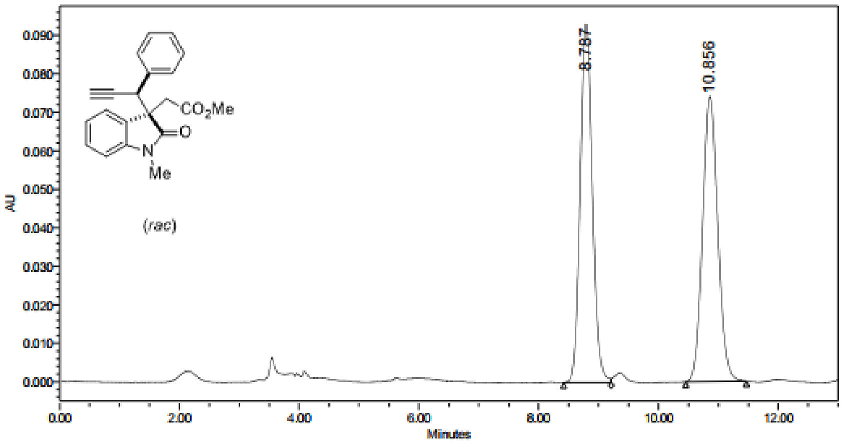

	RT	Area	% Area	Height
1	8.787	1318498	50.20	93123
2	10.856	1307970	49.80	74057

SAMPLE INFORMATION

Sample Name:	wyh-1-82-2-asy-IG-30%-1ml/min	Acquired By:	System
Sample Type:	Unknown	Sample Set Name	wyh182up
Vial:	14	Acq. Method Set:	30% 1ml
Injection #:	1	Processing Method	1
Injection Volume:	10.00 ul	Channel Name:	270.0nm
Run Time:	13.0 Minutes	Proc. Chnl. Descr.:	2998 PDA 270.0 nm (2998
Date Acquired:	3/6/2021 1:39:19 PM CST		
Date Processed:	3/6/2021 2:43:00 PM CST		

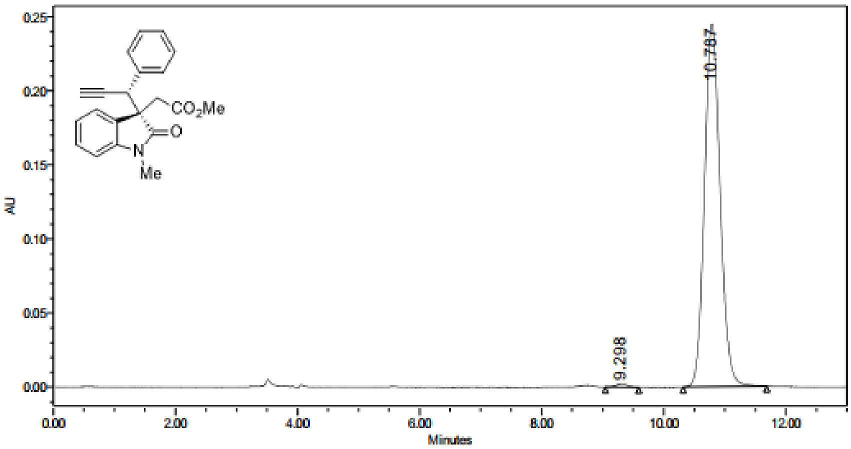

	RT	Area	% Area	Height
1	9.298	27777	0.63	2335
2	10.787	4386613	99.37	244641

Supplementary Fig. 53. HPLC spectra of compound **(*S*,*R*)-3aa**

SAMPLE INFORMATION

Sample Name:	wyh-1-74-1-d-IG-30%-1ml/min	Acquired By:	System
Sample Type:	Unknown	Sample Set Name:	wyhh174
Vial:	32	Acq. Method Set:	30% 1ml
Injection #:	1	Processing Method:	1
Injection Volume:	10.00 ul	Channel Name:	254.0nm
Run Time:	20.0 Minutes	Proc. Chnl. Descr.:	2998 PDA 254.0 nm (2998
Date Acquired:	12/25/2020 1:26:58 PM CST		
Date Processed:	12/30/2020 3:37:34 PM CST		

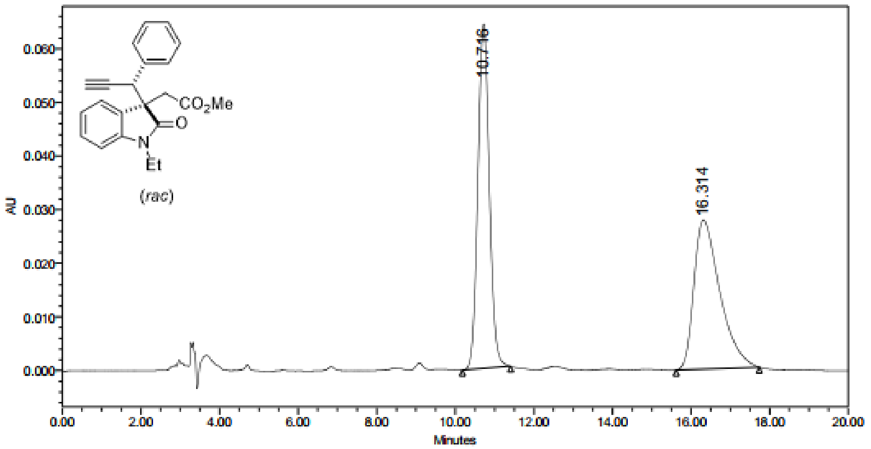

	RT	Area	% Area	Height
1	10.716	1294232	50.37	64133
2	16.314	1275325	49.63	27833

SAMPLE INFORMATION

Sample Name:	wyh-1-74-1-asy-IG-30%-1ml/min	Acquired By:	System
Sample Type:	Unknown	Sample Set Name:	wyh174
Vial:	41	Acq. Method Set:	30% 1ml
Injection #:	1	Processing Method:	1
Injection Volume:	10.00 ul	Channel Name:	254.0nm
Run Time:	20.0 Minutes	Proc. Chnl. Descr.:	2998 PDA 254.0 nm (2998
Date Acquired:	12/24/2020 11:13:25 AM CST		
Date Processed:	12/30/2020 3:38:05 PM CST		

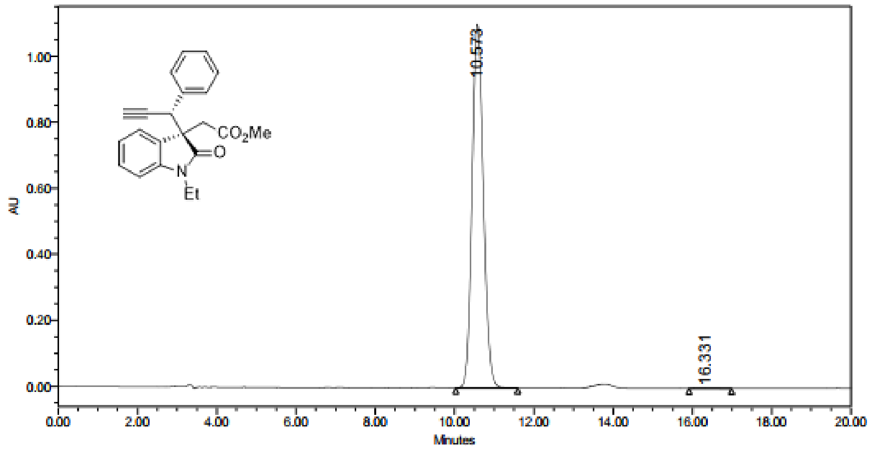

	RT	Area	% Area	Height
1	10.573	21835641	99.91	1100912
2	16.331	20046	0.09	533

Supplementary Fig. 54. HPLC spectra of compound **(*R*,*R*)-3ba**

SAMPLE INFORMATION			
Sample Name:	wyh-1-74-3-rac-d-IG-30%-1ml/min	Acquired By:	System
Sample Type:	Unknown	Sample Set Name:	wyh1743
Vial:	8	Acq. Method Set:	30% 1ml
Injection #:	1	Processing Method:	1
Injection Volume:	10.00 ul	Channel Name:	254.0nm
Run Time:	32.0 Minutes	Proc. Chnl. Descr.:	2998 PDA 254.0 nm (2998
Date Acquired:	12/31/2020 4:12:33 PM CST		
Date Processed:	12/31/2020 5:11:54 PM CST		

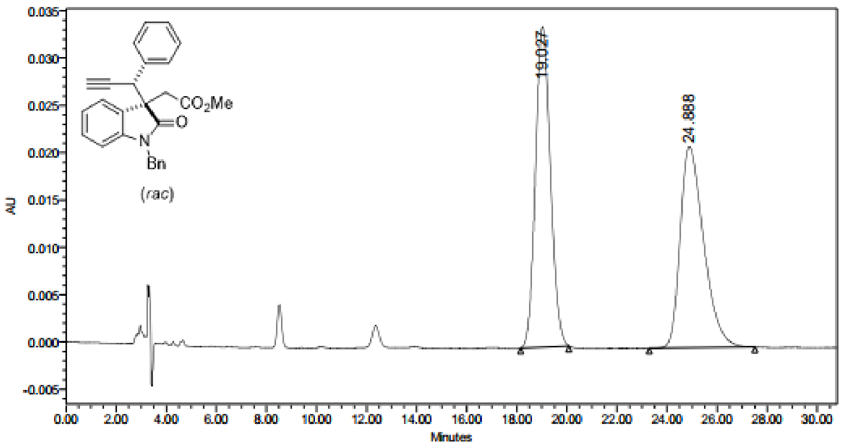

	RT	Area	% Area	Height
1	19.027	1398690	50.38	33886
2	24.888	1377494	49.62	21262

SAMPLE INFORMATION			
Sample Name:	wyh-1-74-3-asy-IG-30%-1ml/min	Acquired By:	System
Sample Type:	Unknown	Sample Set Name:	wyh1743
Vial:	9	Acq. Method Set:	30% 1ml
Injection #:	1	Processing Method:	1
Injection Volume:	10.00 ul	Channel Name:	254.0nm
Run Time:	32.0 Minutes	Proc. Chnl. Descr.:	2998 PDA 254.0 nm (2998
Date Acquired:	12/31/2020 4:44:51 PM CST		
Date Processed:	1/1/2021 5:07:10 PM CST		

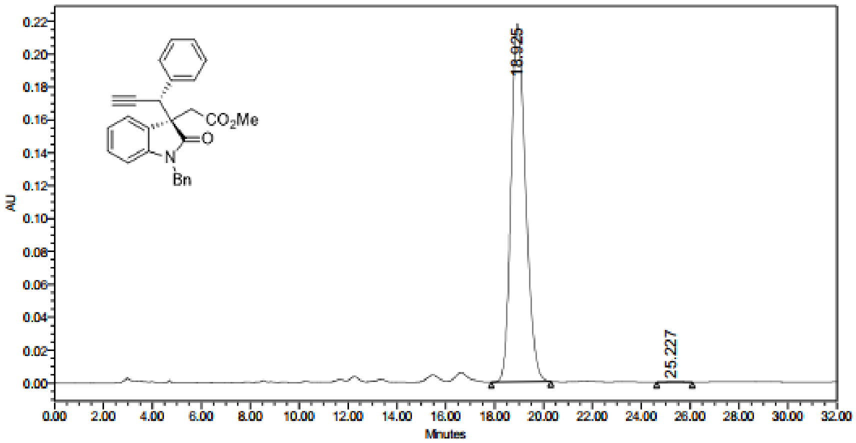

	RT	Area	% Area	Height
1	18.925	8999172	99.94	217624
2	25.227	4996	0.06	109

Supplementary Fig. 55. HPLC spectra of compound **(*R*,*R*)-3ca**

SAMPLE INFORMATION

Sample Name:	wyh-1-74-2-rac-d-IE-30%-1ml/min	Acquired By:	System
Sample Type:	Unknown	Sample Set Name:	wyh1742
Vial:	43	Acq. Method Set:	30% 1ml
Injection #:	1	Processing Method:	1
Injection Volume:	10.00 ul	Channel Name:	254.0nm
Run Time:	37.0 Minutes	Proc. Chnl. Descr.:	2998 PDA 254.0 nm (2998
Date Acquired:	12/30/2020 8:43:58 PM CST		
Date Processed:	12/30/2020 10:07:48 PM CST		

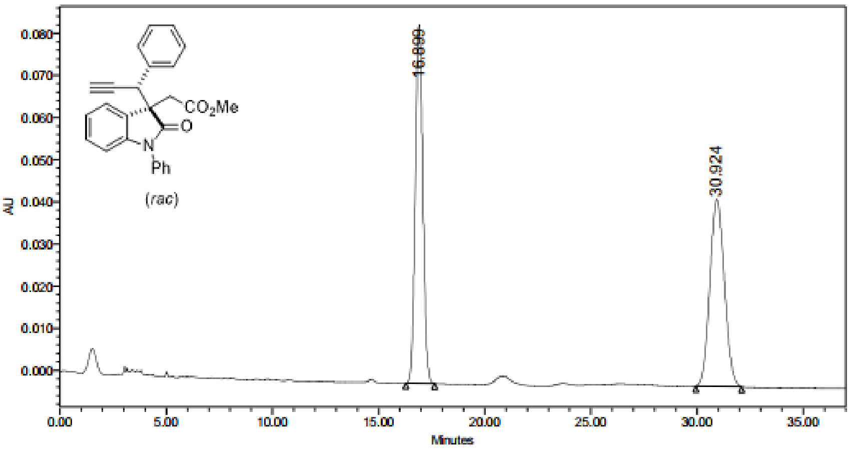

	RT	Area	% Area	Height
1	16.899	2110483	49.99	85122
2	30.924	2110915	50.01	44390

SAMPLE INFORMATION

Sample Name:	wyh-1-74-2-asy-IE-30%-1ml/min	Acquired By:	System
Sample Type:	Unknown	Sample Set Name:	wyh1742
Vial:	44	Acq. Method Set:	30% 1ml
Injection #:	1	Processing Method:	1
Injection Volume:	10.00 ul	Channel Name:	254.0nm
Run Time:	37.0 Minutes	Proc. Chnl. Descr.:	2998 PDA 254.0 nm (2998
Date Acquired:	12/30/2020 9:21:42 PM CST		
Date Processed:	12/30/2020 10:08:48 PM CST		

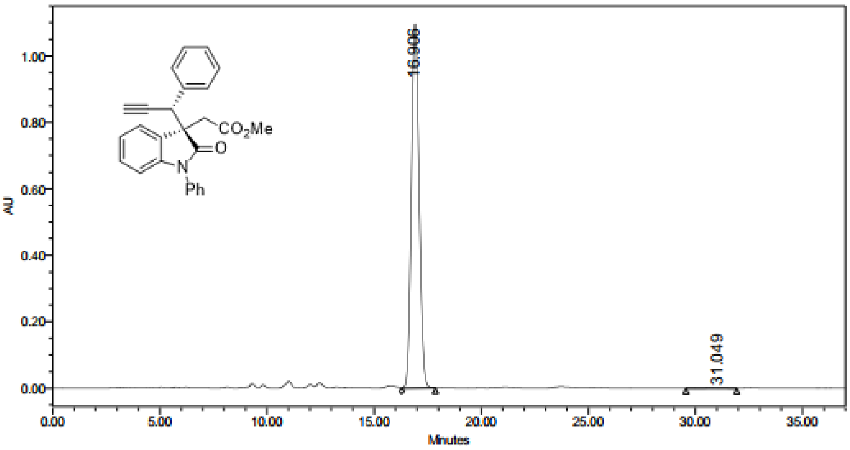

	RT	Area	% Area	Height
1	16.906	27421176	99.75	1095000
2	31.049	68443	0.25	1349

Supplementary Fig. 56. HPLC spectra of compound **(*R*,*R*)-3da**

SAMPLE INFORMATION

Sample Name:	wyh-1-76-1-rac-d-IG-30%-1ml/min	Acquired By:	System
Sample Type:	Unknown	Sample Set Name:	wyh_1_76_123
Vial:	91	Acq. Method Set:	30% 1ml
Injection #:	1	Processing Method:	1
Injection Volume:	10.00 ul	Channel Name:	254.0nm
Run Time:	11.0 Minutes	Proc. Chnl. Descr.:	2998 PDA 254.0 nm (2998
Date Acquired:	1/1/2021 4:33:24 PM CST		
Date Processed:	1/1/2021 5:10:03 PM CST		

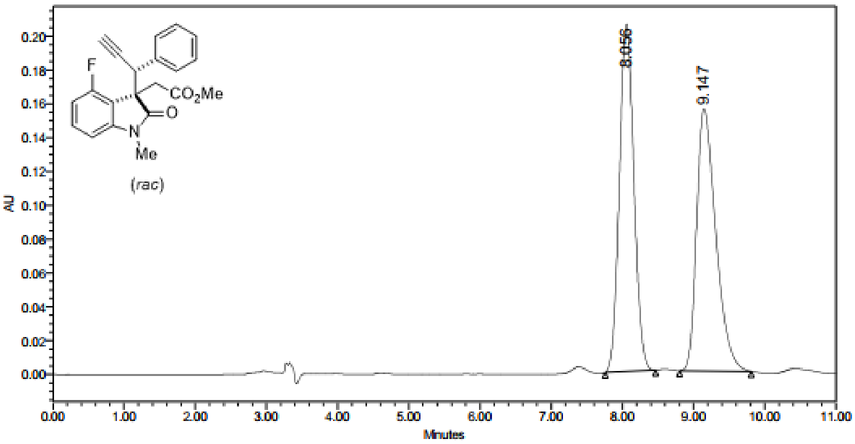

	RT	Area	% Area	Height
1	8.056	2896300	49.99	205215
2	9.147	2896903	50.01	154854

SAMPLE INFORMATION

Sample Name:	wyh-1-76-1-asy-IG-30%-1ml/min	Acquired By:	System
Sample Type:	Unknown	Sample Set Name:	
Vial:	92	Acq. Method Set:	30% 1ml
Injection #:	1	Processing Method:	1
Injection Volume:	10.00 ul	Channel Name:	254.0nm
Run Time:	10.0 Minutes	Proc. Chnl. Descr.:	2998 PDA 254.0 nm (2998
Date Acquired:	1/1/2021 9:23:37 PM CST		
Date Processed:	1/1/2021 9:34:28 PM CST		

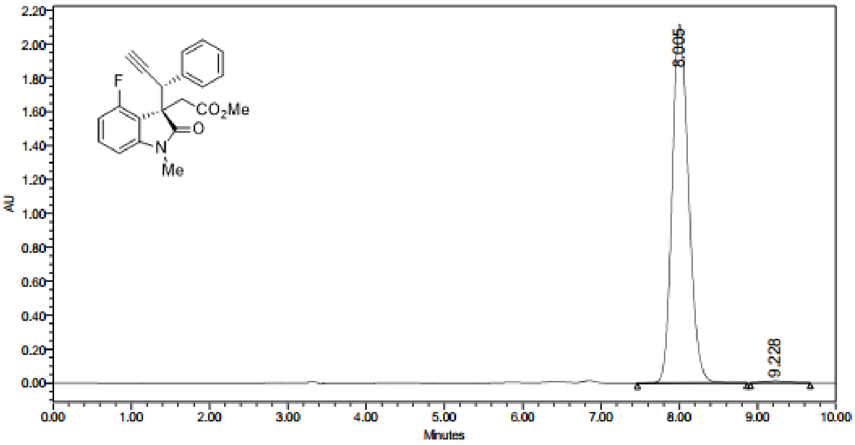

	RT	Area	% Area	Height
1	8.005	30960952	99.30	2117349
2	9.228	217384	0.70	11289

Supplementary Fig. 57. HPLC spectra of compound **(*R*,*R*)-3ea**

SAMPLE INFORMATION			
Sample Name:	wyh-1-76-6-rac-d-IG-30%-1ml/min	Acquired By:	System
Sample Type:	Unknown	Sample Set Name:	
Vial:	10	Acq. Method Set:	30% 1ml
Injection #:	1	Processing Method:	1
Injection Volume:	10.00 ul	Channel Name:	254.0nm
Run Time:	40.0 Minutes	Proc. Chnl. Descr.:	2998 PDA 254.0 nm (2998
Date Acquired:	1/6/2021 2:48:50 PM CST		
Date Processed:	1/6/2021 8:00:35 PM CST		

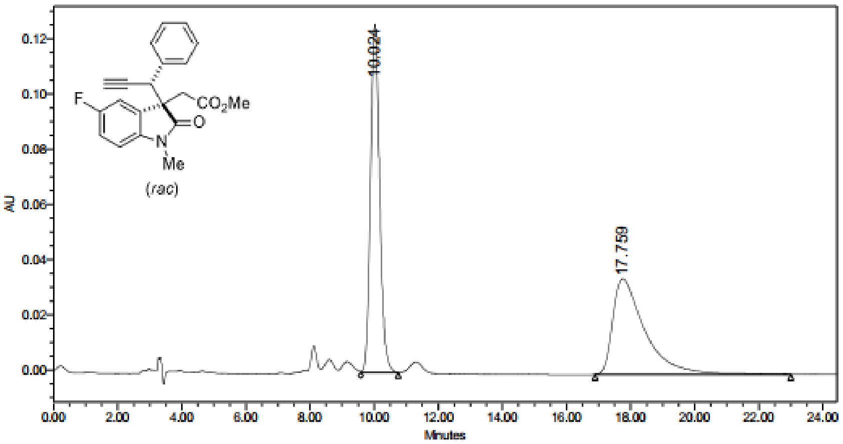

	RT	Area	% Area	Height
1	10.024	2472165	50.77	126221
2	17.759	2396769	49.23	34582

SAMPLE INFORMATION			
Sample Name:	wyh-1-76-6-asy-IG-30%-1ml/min	Acquired By:	System
Sample Type:	Unknown	Sample Set Name:	wyh17667
Vial:	28	Acq. Method Set:	30% 1ml
Injection #:	1	Processing Method:	1
Injection Volume:	10.00 ul	Channel Name:	254.0nm
Run Time:	24.0 Minutes	Proc. Chnl. Descr.:	2998 PDA 254.0 nm (2998
Date Acquired:	1/6/2021 7:07:41 PM CST		
Date Processed:	1/6/2021 7:59:54 PM CST		

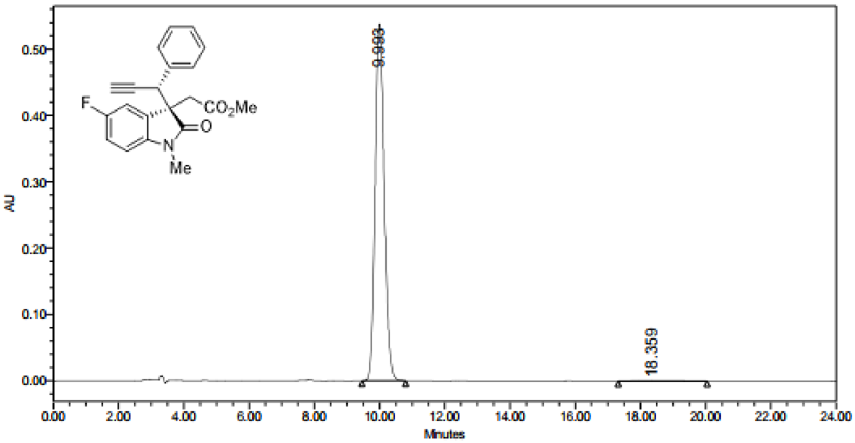

	RT	Area	% Area	Height
1	9.993	10311953	99.65	537791
2	18.359	36149	0.35	490

Supplementary Fig. 58. HPLC spectra of compound **(*R*,*R*)-3fa**

SAMPLE INFORMATION

Sample Name:	wyh-1-76-7-rac-d-IG-30%-1ml/min	Acquired By:	System
Sample Type:	Unknown	Sample Set Name:	
Vial:	11	Acq. Method Set:	30% 1ml
Injection #:	1	Processing Method:	1
Injection Volume:	10.00 ul	Channel Name:	254.0nm@1
Run Time:	40.0 Minutes	Proc. Chnl. Descr.:	2998 PDA 254.0 nm (2998
Date Acquired:	1/6/2021 3:14:08 PM CST		
Date Processed:	1/6/2021 3:40:22 PM CST		

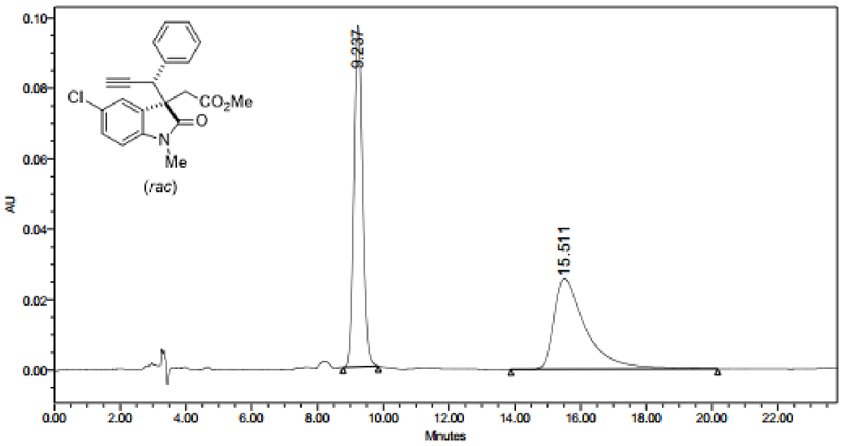

	RT	Area	% Area	Height
1	9.237	1759930	50.68	97015
2	15.511	1713005	49.32	25701

SAMPLE INFORMATION

Sample Name:	wyh-1-76-7-asy-IG-30%-1ml/min	Acquired By:	System
Sample Type:	Unknown	Sample Set Name:	wyh17667
Vial:	29	Acq. Method Set:	30% 1ml
Injection #:	1	Processing Method:	1
Injection Volume:	10.00 ul	Channel Name:	254.0nm
Run Time:	20.0 Minutes	Proc. Chnl. Descr.:	2998 PDA 254.0 nm (2998
Date Acquired:	1/6/2021 7:32:23 PM CST		
Date Processed:	1/6/2021 7:57:01 PM CST		

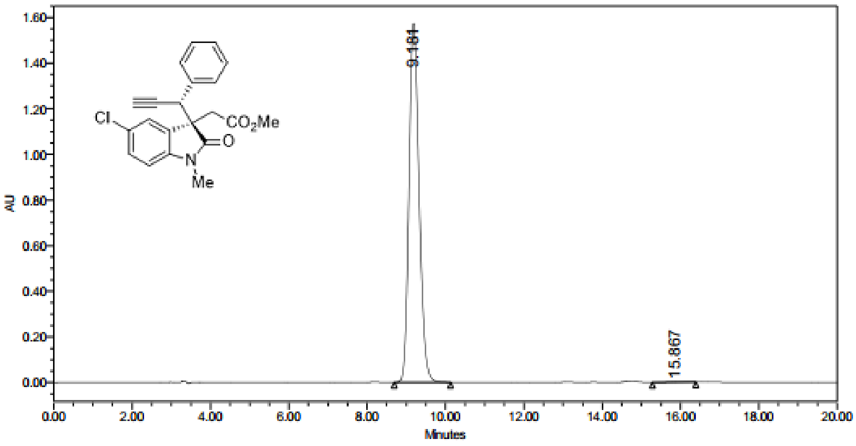

	RT	Area	% Area	Height
1	9.181	28351847	99.88	1570425
2	15.867	33145	0.12	914

Supplementary Fig. 59. HPLC spectra of compound **(*R*,*R*)-3ga**

SAMPLE INFORMATION

Sample Name:	wyh-1-76-2-rac-d-IG-30%-1ml/min	Acquired By:	System
Sample Type:	Unknown	Sample Set Name:	wyh_1_76_123
Vial:	93	Acq. Method Set:	30% 1ml
Injection #:	1	Processing Method:	1
Injection Volume:	10.00 ul	Channel Name:	254.0nm@1
Run Time:	18.0 Minutes	Proc. Chnl. Descr.:	2998 PDA 254.0 nm (2998
Date Acquired:	1/1/2021 5:02:01 PM CST		
Date Processed:	1/1/2021 9:27:07 PM CST		

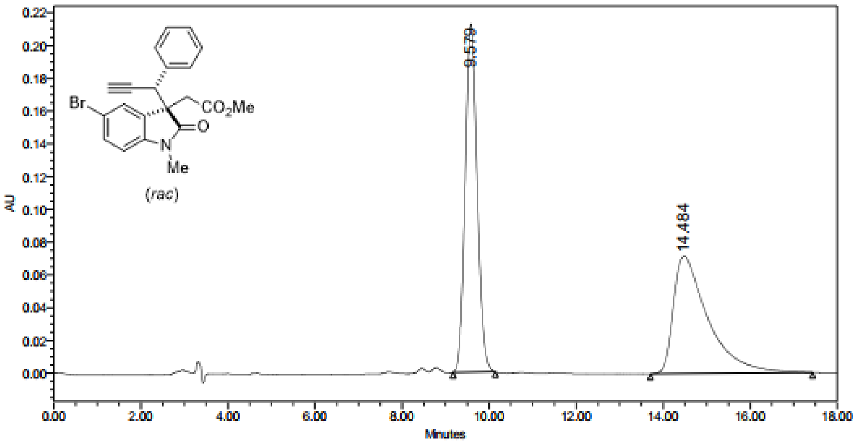

	RT	Area	% Area	Height
1	9.579	4062612	50.72	212415
2	14.484	3946988	49.28	71766

SAMPLE INFORMATION

Sample Name:	wyh-1-76-2-asy-IG-30%-1ml/min	Acquired By:	System
Sample Type:	Unknown	Sample Set Name:	wyh_1_76_123
Vial:	94	Acq. Method Set:	30% 1ml
Injection #:	1	Processing Method:	1
Injection Volume:	10.00 ul	Channel Name:	254.0nm@1
Run Time:	18.0 Minutes	Proc. Chnl. Descr.:	2998 PDA 254.0 nm (2998
Date Acquired:	1/1/2021 5:20:46 PM CST		
Date Processed:	1/1/2021 9:28:50 PM CST		

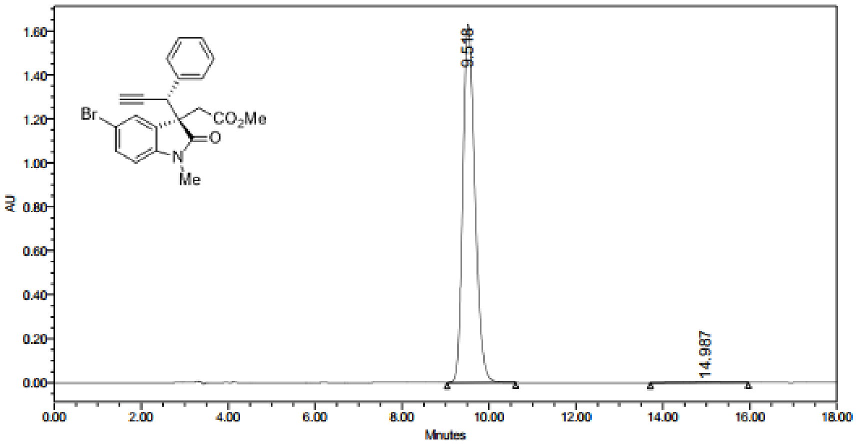

	RT	Area	% Area	Height
1	9.518	31432586	99.62	1629903
2	14.987	119882	0.38	2038

Supplementary Fig. 60. HPLC spectra of compound **(*R*,*R*)-3ha**

SAMPLE INFORMATION

Sample Name:	wyh-1-76-3-rac-d-IG-30%-1ml/min	Acquired By:	System
Sample Type:	Unknown	Sample Set Name:	itmustbefinal
Vial:	40	Acq. Method Set:	30% 1ml
Injection #:	1	Processing Method:	1
Injection Volume:	10.00 ul	Channel Name:	254.0nm
Run Time:	19.0 Minutes	Proc. Chnl. Descr.:	2998 PDA 254.0 nm (2998
Date Acquired:	1/4/2021 5:50:14 PM CST		
Date Processed:	1/4/2021 6:50:30 PM CST		

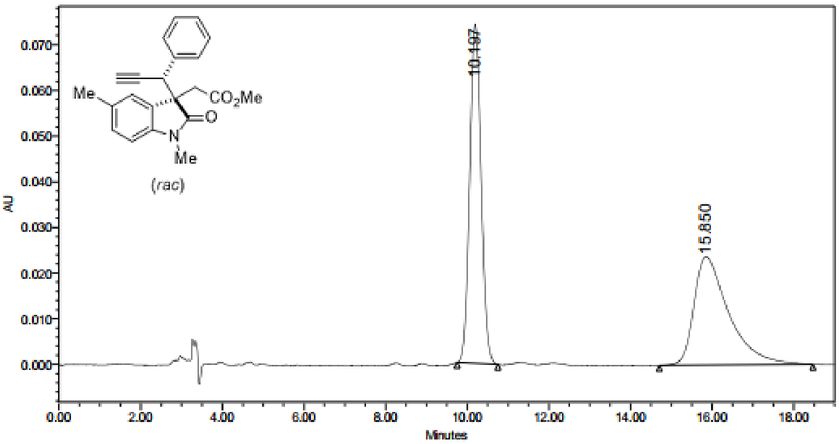

	RT	Area	% Area	Height
1	10.197	1432801	50.45	74221
2	15.850	1407517	49.55	23716

SAMPLE INFORMATION

Sample Name:	wyh-1-76-3-asy-IG-30%-1ml/min	Acquired By:	System
Sample Type:	Unknown	Sample Set Name:	itmustbefinal
Vial:	41	Acq. Method Set:	30% 1ml
Injection #:	1	Processing Method:	1
Injection Volume:	10.00 ul	Channel Name:	254.0nm
Run Time:	19.0 Minutes	Proc. Chnl. Descr.:	2998 PDA 254.0 nm (2998
Date Acquired:	1/4/2021 6:09:57 PM CST		
Date Processed:	7/12/2021 4:45:43 PM CST		

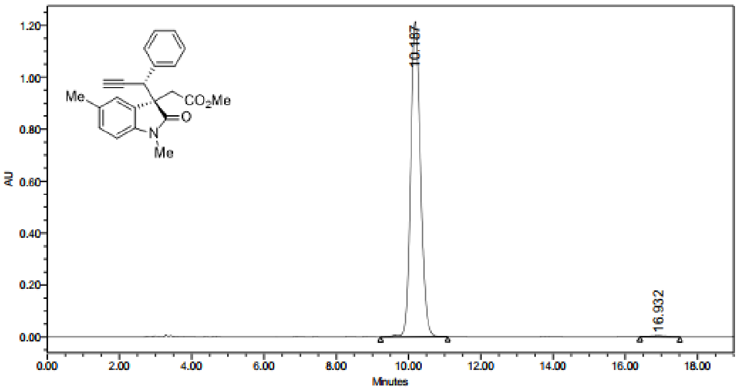

	RT	Area	% Area	Height
1	10.187	23141433	99.48	1213865
2	16.932	119855	0.52	4052

Supplementary Fig. 61. HPLC spectra of compound **(*R*,*R*)-3ia**

SAMPLE INFORMATION

Sample Name:	wyh-1-76-9-rac-d-IA-15%-1ml/min	Acquired By:	System
Sample Type:	Unknown	Sample Set Name:	wyh1769
Vial:	92	Acq. Method Set:	15% 1ml
Injection #:	1	Processing Method:	1
Injection Volume:	10.00 ul	Channel Name:	254.0nm
Run Time:	18.0 Minutes	Proc. Chnl. Descr.:	2998 PDA 254.0 nm (2998
Date Acquired:	1/29/2021 2:49:53 PM CST		
Date Processed:	1/29/2021 3:30:06 PM CST		

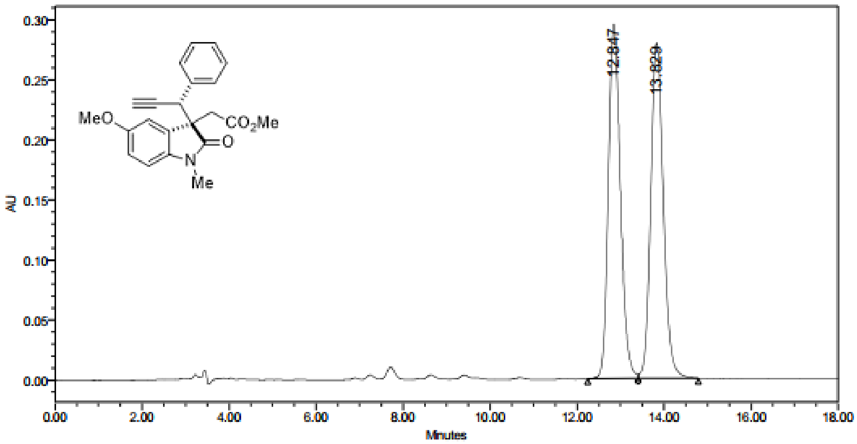

	RT	Area	% Area	Height
1	12.847	5674250	49.93	295158
2	13.829	5689212	50.07	279436

SAMPLE INFORMATION

Sample Name:	wyh-1-76-9-asy-IA-15%-1ml/min	Acquired By:	System
Sample Type:	Unknown	Sample Set Name:	wyh1769
Vial:	93	Acq. Method Set:	15% 1ml
Injection #:	1	Processing Method:	1
Injection Volume:	10.00 ul	Channel Name:	254.0nm
Run Time:	18.0 Minutes	Proc. Chnl. Descr.:	2998 PDA 254.0 nm (2998
Date Acquired:	1/29/2021 3:08:35 PM CST		
Date Processed:	1/29/2021 3:32:12 PM CST		

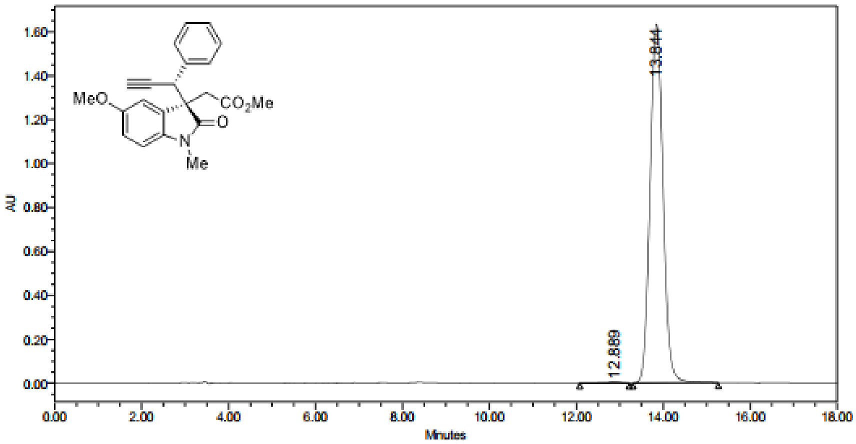

	RT	Area	% Area	Height
1	12.889	108058	0.32	5399
2	13.844	34016725	99.68	1633558

Supplementary Fig. 62. HPLC spectra of compound **(*R*,*R*)-3ja**

SAMPLE INFORMATION

Sample Name:	wyh-1-167-3-rac-IG-30%-1ml/min	Acquired By:	System
Sample Type:	Unknown	Sample Set Name	wyh16713
Vial:	54	Acq. Method Set:	30% 1ml
Injection #:	1	Processing Method	1
Injection Volume:	10.00 ul	Channel Name:	254.0nm@1
Run Time:	17.0 Minutes	Proc. Chnl. Descr.:	2998 PDA 254.0 nm (2998
Date Acquired:	7/13/2021 7:09:37 PM CST		
Date Processed:	7/13/2021 7:46:30 PM CST		

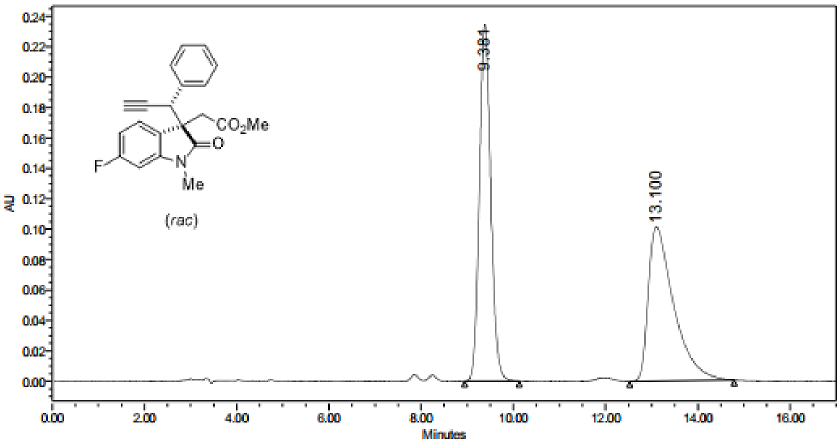

	RT	Area	% Area	Height
1	9.381	4025654	50.72	234855
2	13.100	3911431	49.28	101478

SAMPLE INFORMATION

Sample Name:	wyh-1-167-3-asy-IG-30%-1ml/min	Acquired By:	System
Sample Type:	Unknown	Sample Set Name	wyh16713
Vial:	55	Acq. Method Set:	30% 1ml
Injection #:	1	Processing Method	1
Injection Volume:	10.00 ul	Channel Name:	254.0nm@1
Run Time:	17.0 Minutes	Proc. Chnl. Descr.:	2998 PDA 254.0 nm (2998
Date Acquired:	7/13/2021 7:27:19 PM CST		
Date Processed:	7/13/2021 7:47:11 PM CST		

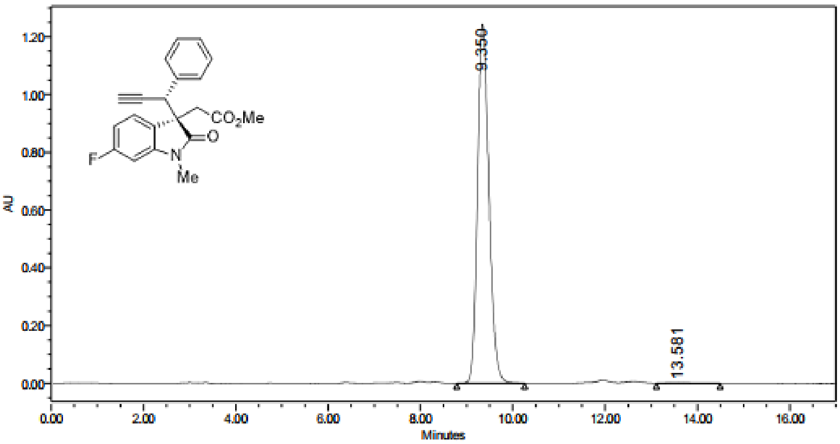

	RT	Area	% Area	Height
1	9.350	21321429	99.81	1242968
2	13.581	41080	0.19	1206

Supplementary Fig. 63. HPLC spectra of compound ***(R,R)*-3ka**

SAMPLE INFORMATION

Sample Name:	wyh-1-76-5-rac-d-IG-30%-1ml/min	Acquired By:	System
Sample Type:	Unknown	Sample Set Name:	
Vial:	94	Acq. Method Set:	30% 1ml
Injection #:	1	Processing Method:	1
Injection Volume:	10.00 ul	Channel Name:	254.0nm
Run Time:	40.0 Minutes	Proc. Chnl. Descr.:	2998 PDA 254.0 nm (2998
Date Acquired:	1/3/2021 4:00:18 PM CST		
Date Processed:	1/3/2021 4:15:48 PM CST		

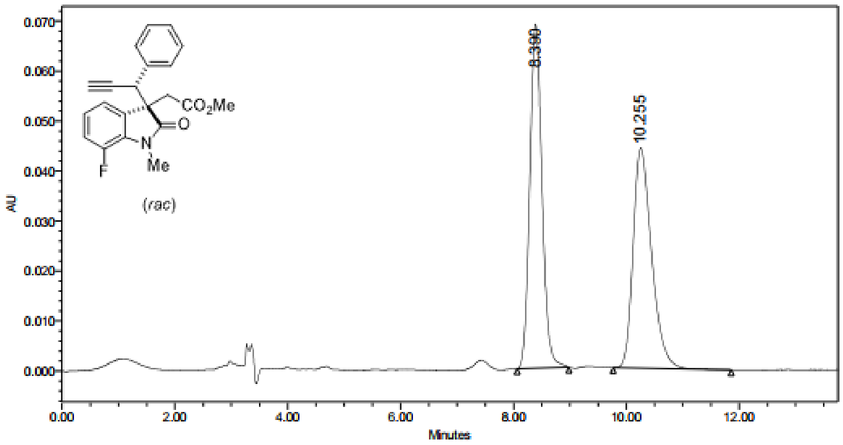

	RT	Area	% Area	Height
1	8.390	1040207	50.86	68815
2	10.255	1005115	49.14	44068

SAMPLE INFORMATION

Sample Name:	wyh-1-76-5-asy-IG-30%-1ml/min	Acquired By:	System
Sample Type:	Unknown	Sample Set Name:	
Vial:	95	Acq. Method Set:	30% 1ml
Injection #:	1	Processing Method:	1
Injection Volume:	10.00 ul	Channel Name:	254.0nm
Run Time:	40.0 Minutes	Proc. Chnl. Descr.:	2998 PDA 254.0 nm (2998
Date Acquired:	1/3/2021 4:15:06 PM CST		
Date Processed:	1/3/2021 4:31:28 PM CST		

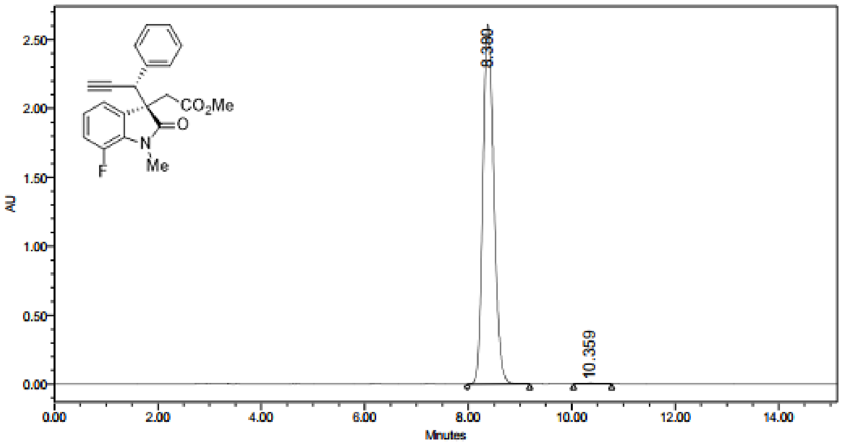

	RT	Area	% Area	Height
1	8.380	38698850	99.76	2610008
2	10.359	91211	0.24	4878

Supplementary Fig. 64. HPLC spectra of compound **(*R*,*R*)-3la**

SAMPLE INFORMATION

Sample Name:	wyh-1-76-8-rac-d-IG-30%-1ml/min	Acquired By:	System
Sample Type:	Unknown	Sample Set Name:	
Vial:	12	Acq. Method Set:	30% 1ml
Injection #:	1	Processing Method:	1
Injection Volume:	10.00 ul	Channel Name:	230.0nm
Run Time:	40.0 Minutes	Proc. Chnl. Descr.:	2998 PDA 230.0 nm (2998
Date Acquired:	1/6/2021 3:38:51 PM CST		
Date Processed:	1/6/2021 8:17:31 PM CST		

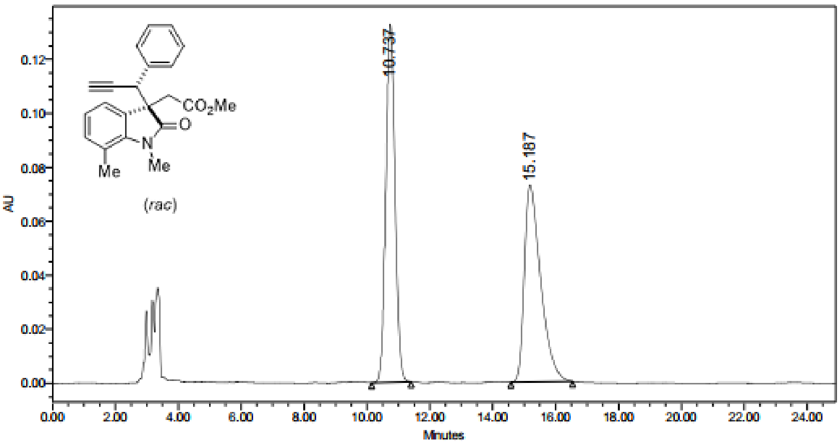

	RT	Area	% Area	Height
1	10.737	2664485	50.05	132562
2	15.187	2659201	49.95	72991

SAMPLE INFORMATION

Sample Name:	wyh-1-76-8-asy-IG-30%-1ml/min	Acquired By:	System
Sample Type:	Unknown	Sample Set Name:	
Vial:	59	Acq. Method Set:	30% 1ml
Injection #:	1	Processing Method:	1
Injection Volume:	10.00 ul	Channel Name:	230.0nm
Run Time:	40.0 Minutes	Proc. Chnl. Descr.:	2998 PDA 230.0 nm (2998
Date Acquired:	1/6/2021 7:55:34 PM CST		
Date Processed:	1/6/2021 8:18:38 PM CST		

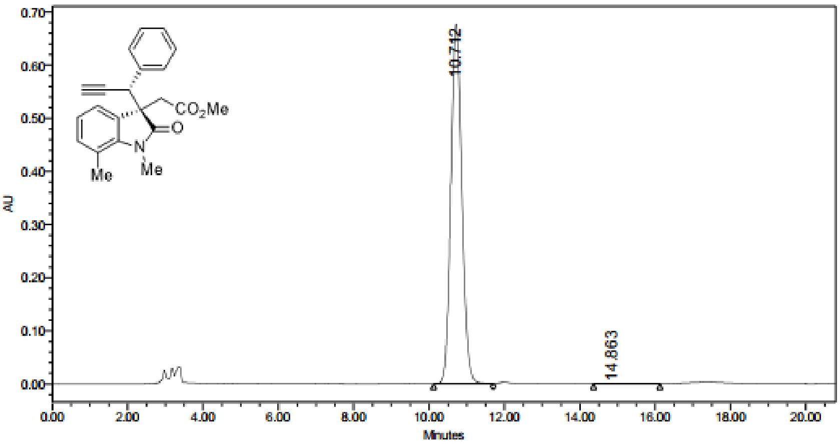

	RT	Area	% Area	Height
1	10.712	13756777	99.77	676506
2	14.863	31147	0.23	807

Supplementary Fig. 65. HPLC spectra of compound **(*R*,*R*)-3ma**

SAMPLE INFORMATION

Sample Name:	wyh-1-78-3-rac-d-IG-30%-1ml/min	Acquired By:	System
Sample Type:	Unknown	Sample Set Name:	wyh1783
Vial:	57	Acq. Method Set:	30% 1ml
Injection #:	1	Processing Method:	1
Injection Volume:	10.00 ul	Channel Name:	254.0nm
Run Time:	15.0 Minutes	Proc. Chnl. Descr.:	2998 PDA 254.0 nm (2998
Date Acquired:	1/16/2021 7:33:56 PM CST		
Date Processed:	1/16/2021 8:07:41 PM CST		

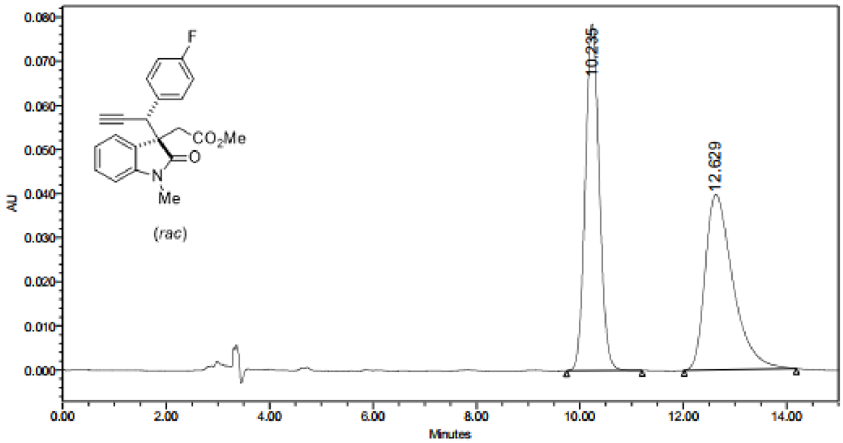

	RT	Area	% Area	Height
1	10.235	1488308	49.96	78611
2	12.629	1490860	50.04	39788

SAMPLE INFORMATION

Sample Name:	wyh-1-78-3-asy-IG-30%-1ml/min	Acquired By:	System
Sample Type:	Unknown	Sample Set Name:	wyh1783
Vial:	58	Acq. Method Set:	30% 1ml
Injection #:	1	Processing Method:	1
Injection Volume:	10.00 ul	Channel Name:	254.0nm
Run Time:	15.0 Minutes	Proc. Chnl. Descr.:	2998 PDA 254.0 nm (2998
Date Acquired:	1/16/2021 7:49:39 PM CST		
Date Processed:	1/16/2021 8:08:22 PM CST		

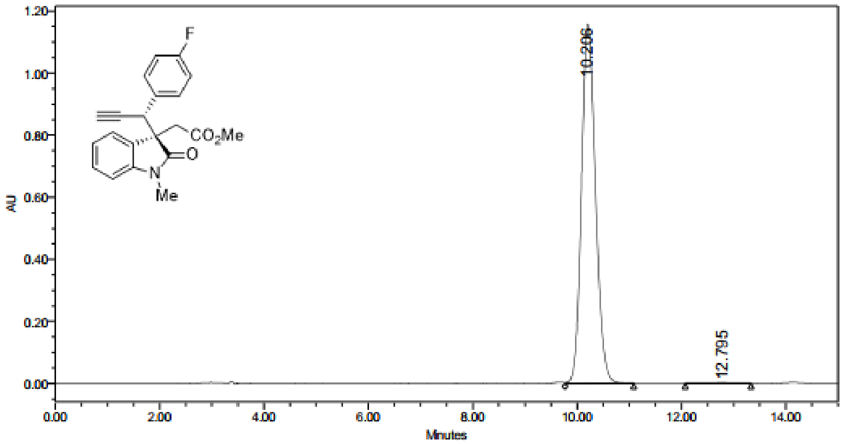

	RT	Area	% Area	Height
1	10.206	21680665	99.84	1157434
2	12.795	34892	0.16	1011

Supplementary Fig. 66. HPLC spectra of compound **(*R*,*R*)-3ab**

SAMPLE INFORMATION

Sample Name:	wyh-1-78-1-rac-d-IG-30%-1ml/min	Acquired By:	System
Sample Type:	Unknown	Sample Set Name:	wyh1781
Vial:	77	Acq. Method Set:	30% 1ml
Injection #:	1	Processing Method:	1
Injection Volume:	10.00 ul	Channel Name:	230.0nm
Run Time:	40.0 Minutes	Proc. Chnl. Descr.:	2998 PDA 230.0 nm (2998
Date Acquired:	1/8/2021 9:41:54 AM CST		
Date Processed:	1/8/2021 11:07:37 AM CST		

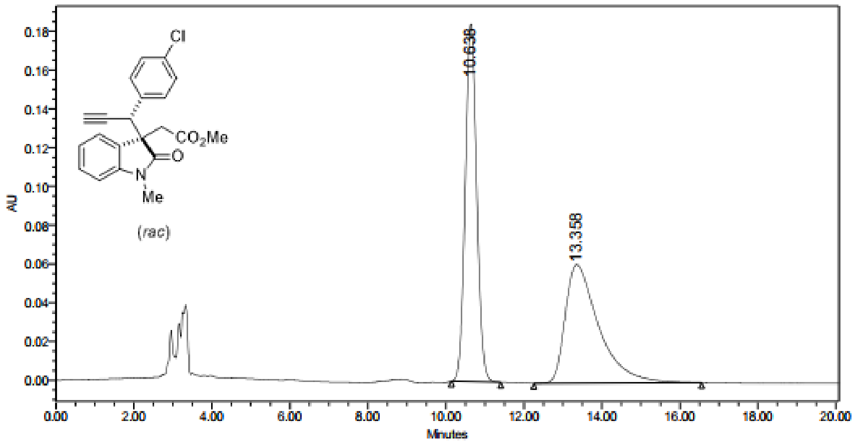

	RT	Area	% Area	Height
1	10.638	3755736	50.78	184290
2	13.358	3639639	49.22	61008

SAMPLE INFORMATION

Sample Name:	wyh-1-78-1-asy-IG-30%-1ml/min	Acquired By:	System
Sample Type:	Unknown	Sample Set Name:	wyh17812
Vial:	78	Acq. Method Set:	30% 1ml
Injection #:	1	Processing Method:	1
Injection Volume:	10.00 ul	Channel Name:	230.0nm
Run Time:	20.0 Minutes	Proc. Chnl. Descr.:	2998 PDA 230.0 nm (2998
Date Acquired:	1/8/2021 10:28:22 AM CST		
Date Processed:	1/8/2021 11:06:44 AM CST		

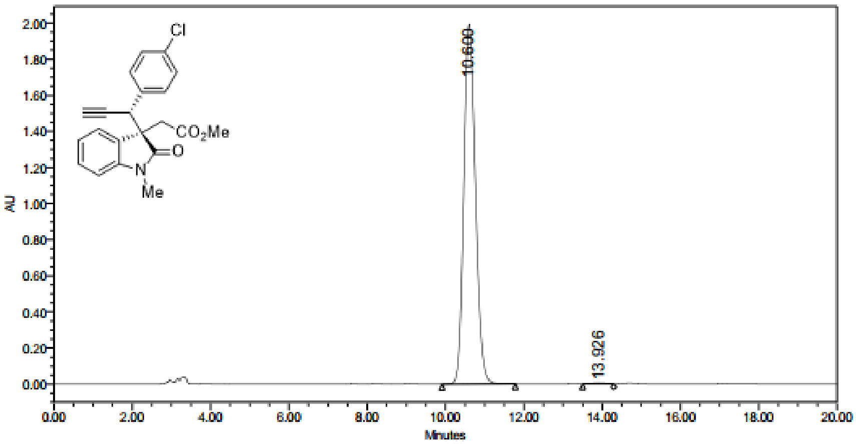

	RT	Area	% Area	Height
1	10.600	41249361	99.70	1992212
2	13.926	122091	0.30	5613

Supplementary Fig. 67. HPLC spectra of compound **(*R*,*R*)-3ac**

SAMPLE INFORMATION			
Sample Name:	wyh-1-78-2-rac-d-IG-30%-1ml/min	Acquired By:	System
Sample Type:	Unknown	Sample Set Name:	
Vial:	79	Acq. Method Set:	30% 1ml
Injection #:	1	Processing Method:	1
Injection Volume:	10.00 ul	Channel Name:	230.0nm
Run Time:	40.0 Minutes	Proc. Chnl. Descr.:	2998 PDA 230.0 nm (2998
Date Acquired:	1/8/2021 10:03:30 AM CST		
Date Processed:	1/8/2021 11:10:59 AM CST		

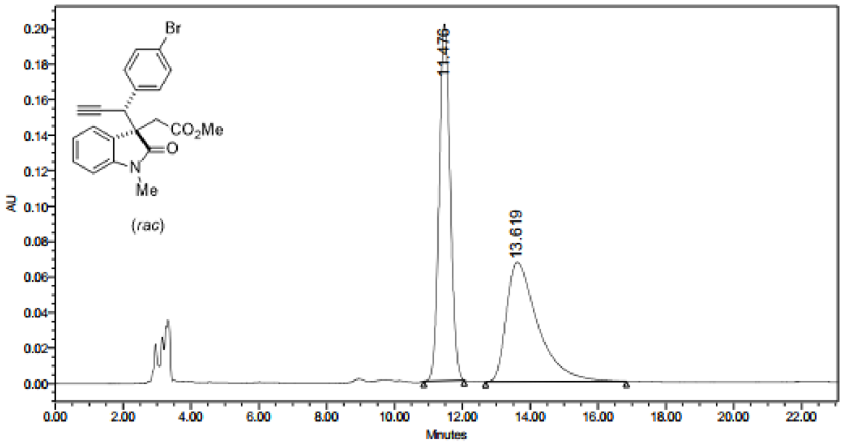

	RT	Area	% Area	Height
1	11.476	4476860	50.75	200959
2	13.619	4344056	49.25	67375

SAMPLE INFORMATION			
Sample Name:	wyh-1-78-2-asy-IG-30%-1ml/min	Acquired By:	System
Sample Type:	Unknown	Sample Set Name:	wyh17812
Vial:	80	Acq. Method Set:	30% 1ml
Injection #:	1	Processing Method:	1
Injection Volume:	10.00 ul	Channel Name:	230.0nm
Run Time:	20.0 Minutes	Proc. Chnl. Descr.:	2998 PDA 230.0 nm (2998
Date Acquired:	1/8/2021 10:49:04 AM CST		
Date Processed:	1/8/2021 11:11:15 AM CST		

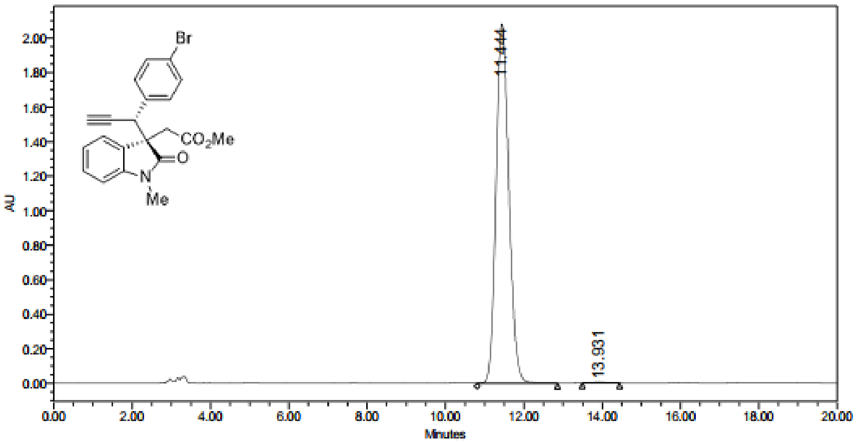

	RT	Area	% Area	Height
1	11.444	47026853	99.74	2080336
2	13.931	123600	0.26	5846

Supplementary Fig. 68. HPLC spectra of compound **(*R*,*R*)-3ad**

SAMPLE INFORMATION

Sample Name:		Acquired By:	System
Sample Type:	Unknown	Sample Set Name	wyh1784racdown
Vial:	49	Acq. Method Set:	30% 1ml
Injection #:	1	Processing Method	1
Injection Volume:	10.00 ul	Channel Name:	254.0nm
Run Time:	60.0 Minutes	Proc. Chnl. Descr.:	2998 PDA 254.0 nm (2998
Date Acquired:	1/17/2021 5:20:35 PM CST		
Date Processed:	1/17/2021 6:03:35 PM CST		

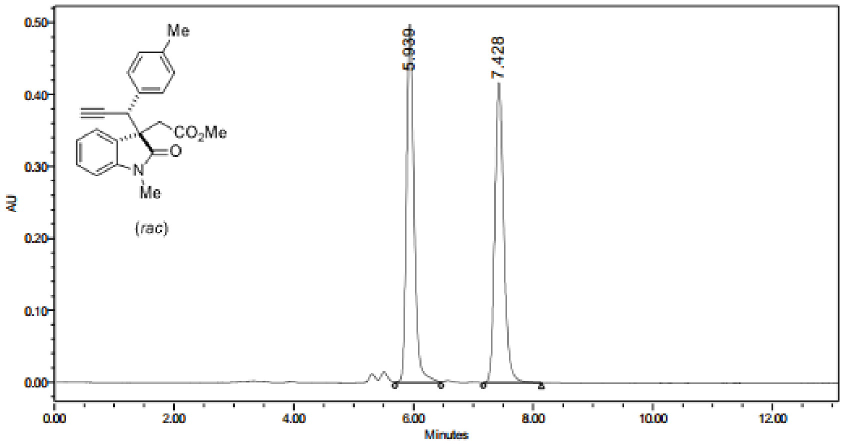

	RT	Area	% Area	Height
1	5.939	4396267	50.48	497966
2	7.428	4313244	49.52	417090

SAMPLE INFORMATION

Sample Name:	wyh-1-78-4-asy-IA-30%-1ml/min	Acquired By:	System
Sample Type:	Unknown	Sample Set Name	
Vial:	50	Acq. Method Set:	30% 1ml
Injection #:	1	Processing Method	1
Injection Volume:	10.00 ul	Channel Name:	254.0nm
Run Time:	11.0 Minutes	Proc. Chnl. Descr.:	2998 PDA 254.0 nm (2998
Date Acquired:	1/17/2021 5:51:33 PM CST		
Date Processed:	1/17/2021 6:04:02 PM CST		

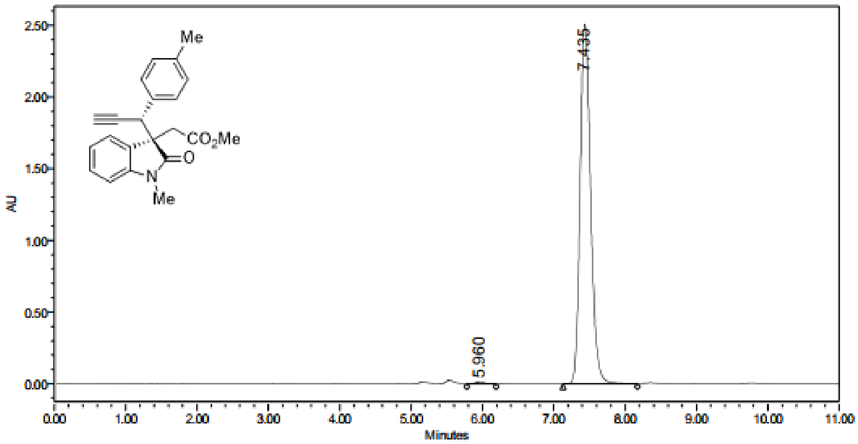

	RT	Area	% Area	Height
1	5.960	111198	0.43	13336
2	7.435	25470899	99.57	2507009

Supplementary Fig. 69. HPLC spectra of compound **(*R*,*R*)-3ae**

SAMPLE INFORMATION			
Sample Name:	wyh-1-78-5-rac-d-IG-30%-1ml/min	Acquired By:	System
Sample Type:	Unknown	Sample Set Name:	wyh_1_78_5
Vial:	19	Acq. Method Set:	30% 1ml
Injection #:	1	Processing Method:	1
Injection Volume:	10.00 ul	Channel Name:	254.0nm
Run Time:	24.0 Minutes	Proc. Chnl. Descr.:	2998 PDA 254.0 nm (2998
Date Acquired:	1/15/2021 1:03:31 PM CST		
Date Processed:	1/15/2021 2:17:55 PM CST		

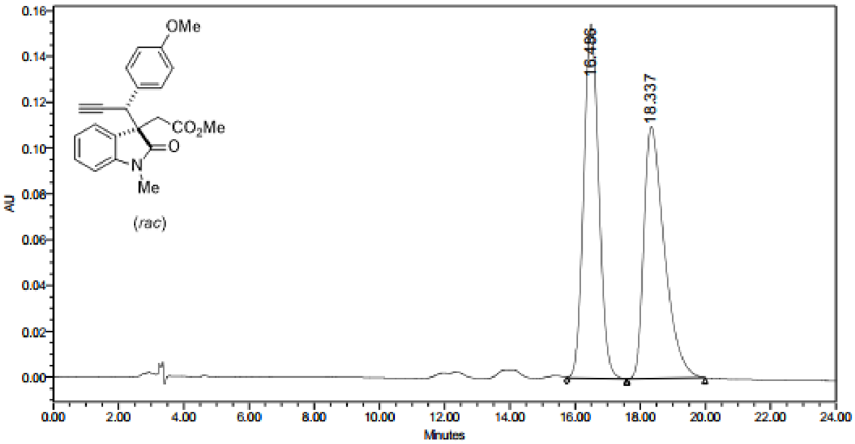

	RT	Area	% Area	Height
1	16.486	4992718	50.65	154628
2	18.337	4864342	49.35	109942

SAMPLE INFORMATION			
Sample Name:	wyh-1-78-5-asy-IG-30%-1ml/min	Acquired By:	System
Sample Type:	Unknown	Sample Set Name:	wyh_1_78_5
Vial:	20	Acq. Method Set:	30% 1ml
Injection #:	1	Processing Method:	1
Injection Volume:	10.00 ul	Channel Name:	254.0nm
Run Time:	24.0 Minutes	Proc. Chnl. Descr.:	2998 PDA 254.0 nm (2998
Date Acquired:	1/15/2021 1:28:15 PM CST		
Date Processed:	1/15/2021 2:18:12 PM CST		

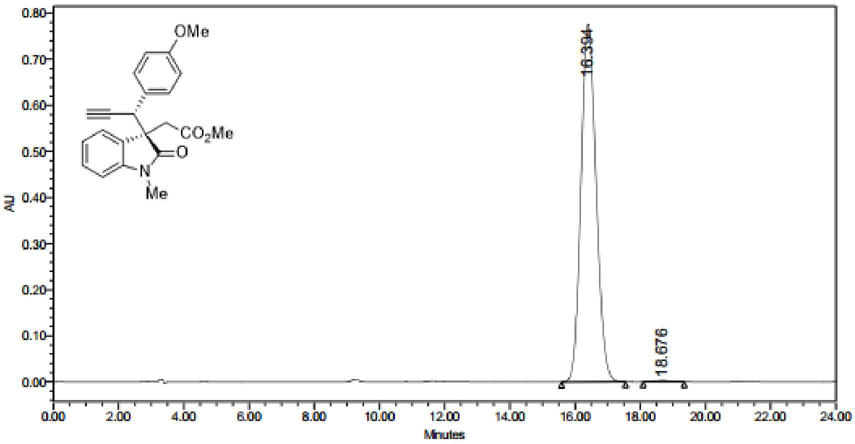

	RT	Area	% Area	Height
1	16.394	25084232	99.61	776259
2	18.676	99206	0.39	2505

Supplementary Fig. 70. HPLC spectra of compound **(*R*,*R*)-3af**

SAMPLE INFORMATION

Sample Name:	wyh-1-78-9-rac-d-IG-30%-1ml/min	Acquired By:	System
Sample Type:	Unknown	Sample Set Name:	wyh2021123
Vial:	8	Acq. Method Set:	30% 1ml
Injection #:	1	Processing Method:	1
Injection Volume:	10.00 ul	Channel Name:	254.0nm
Run Time:	20.0 Minutes	Proc. Chnl. Descr.:	2998 PDA 254.0 nm (2998
Date Acquired:	1/23/2021 10:33:21 AM CST		
Date Processed:	1/24/2021 9:45:25 PM CST		

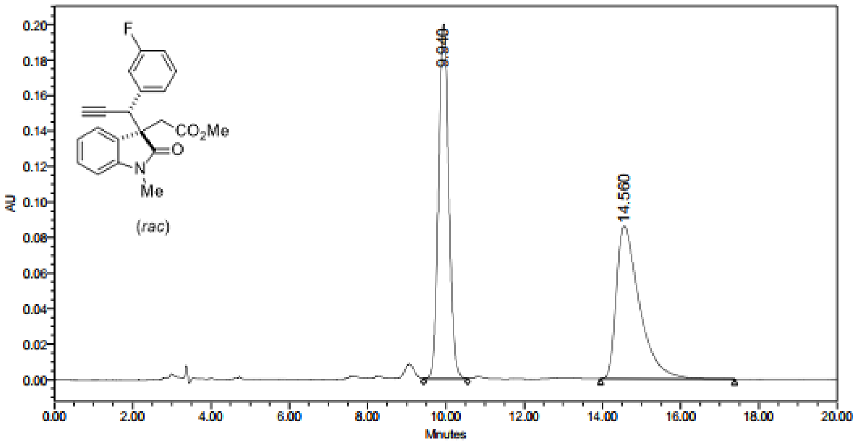

	RT	Area	% Area	Height
1	9.940	3578794	50.46	199591
2	14.560	3513676	49.54	86076

SAMPLE INFORMATION

Sample Name:	wyh-1-78-9-asy-IG-30%-1ml/min	Acquired By:	System
Sample Type:	Unknown	Sample Set Name:	wyh1789
Vial:	5	Acq. Method Set:	30% 1ml
Injection #:	1	Processing Method:	1
Injection Volume:	10.00 ul	Channel Name:	254.0nm
Run Time:	20.0 Minutes	Proc. Chnl. Descr.:	2998 PDA 254.0 nm (2998
Date Acquired:	1/24/2021 9:27:23 PM CST		
Date Processed:	1/24/2021 9:48:10 PM CST		

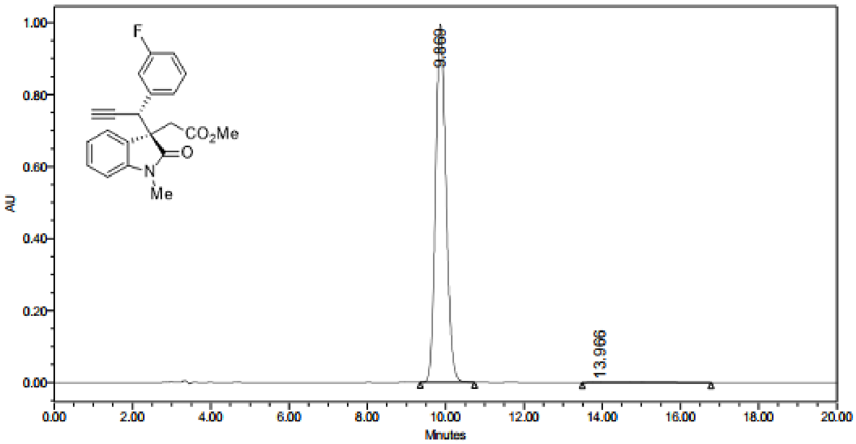

	RT	Area	% Area	Height
1	9.869	18185456	99.65	994770
2	13.966	62965	0.35	725

Supplementary Fig. 71. HPLC spectra of compound **(*R*,*R*)-3ag**

SAMPLE INFORMATION

Sample Name:	wyh-1-78-6-rac-d-IG-15%-1ml/min	Acquired By:	System
Sample Type:	Unknown	Sample Set Name:	
Vial:	88	Acq. Method Set:	15% 1ml
Injection #:	1	Processing Method:	1
Injection Volume:	10.00 ul	Channel Name:	254.0nm
Run Time:	100.0 Minutes	Proc. Chnl. Descr.:	2998 PDA 254.0 nm (2998
Date Acquired:	1/16/2021 8:14:32 PM CST		
Date Processed:	1/16/2021 9:24:36 PM CST		

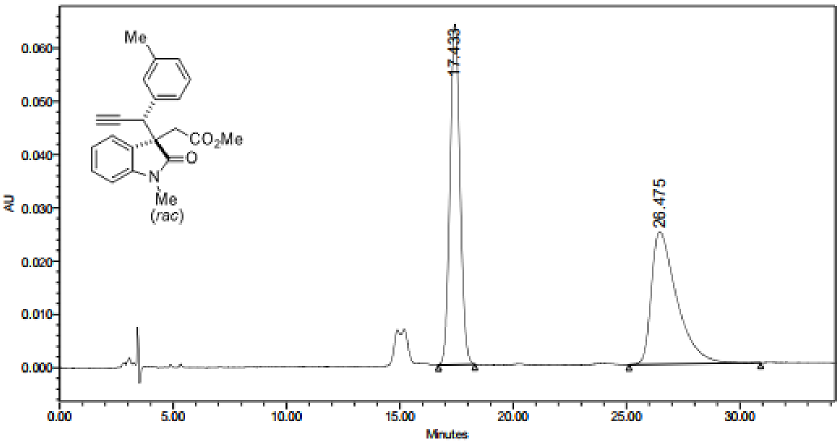

	RT	Area	% Area	Height
1	17.433	1962375	50.28	63918
2	26.475	1940503	49.72	24919

SAMPLE INFORMATION

Sample Name:	wyh-1-78-6-asy-IG-15%-1ml/min	Acquired By:	System
Sample Type:	Unknown	Sample Set Name:	
Vial:	89	Acq. Method Set:	15% 1ml
Injection #:	1	Processing Method:	1
Injection Volume:	10.00 ul	Channel Name:	254.0nm
Run Time:	34.0 Minutes	Proc. Chnl. Descr.:	2998 PDA 254.0 nm (2998
Date Acquired:	1/16/2021 8:49:48 PM CST		
Date Processed:	1/16/2021 9:25:43 PM CST		

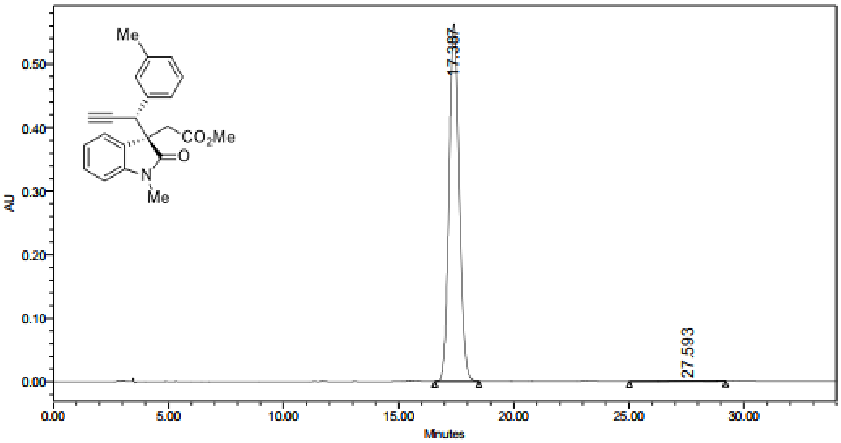

	RT	Area	% Area	Height
1	17.387	17235743	99.76	562416
2	27.593	41009	0.24	673

Supplementary Fig. 72. HPLC spectra of compound **(*R*,*R*)-3ah**

SAMPLE INFORMATION

Sample Name:	wyh-1-78-10-rac-d-IG-30%-1ml/min	Acquired By:	System
Sample Type:	Unknown	Sample Set Name:	wyh17810racdown
Vial:	3	Acq. Method Set:	30% 1ml
Injection #:	1	Processing Method:	1
Injection Volume:	10.00 ul	Channel Name:	254.0nm
Run Time:	60.0 Minutes	Proc. Chnl. Descr.:	2998 PDA 254.0 nm (2998
Date Acquired:	1/21/2021 9:58:55 AM CST		
Date Processed:	1/21/2021 11:00:38 AM CST		

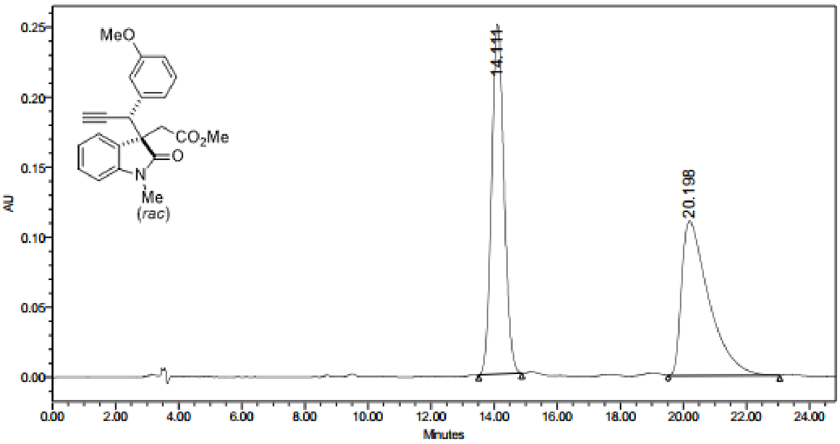

	RT	Area	% Area	Height
1	14.111	6763591	50.70	249968
2	20.198	6576263	49.30	110259

SAMPLE INFORMATION

Sample Name:	wyh-1-78-10-asy-IG-30%-1ml/min	Acquired By:	System
Sample Type:	Unknown	Sample Set Name:	
Vial:	5	Acq. Method Set:	30% 1ml
Injection #:	1	Processing Method:	1
Injection Volume:	10.00 ul	Channel Name:	254.0nm@1
Run Time:	25.0 Minutes	Proc. Chnl. Descr.:	2998 PDA 254.0 nm (2998
Date Acquired:	1/21/2021 10:29:34 AM CST		
Date Processed:	1/21/2021 11:01:07 AM CST		

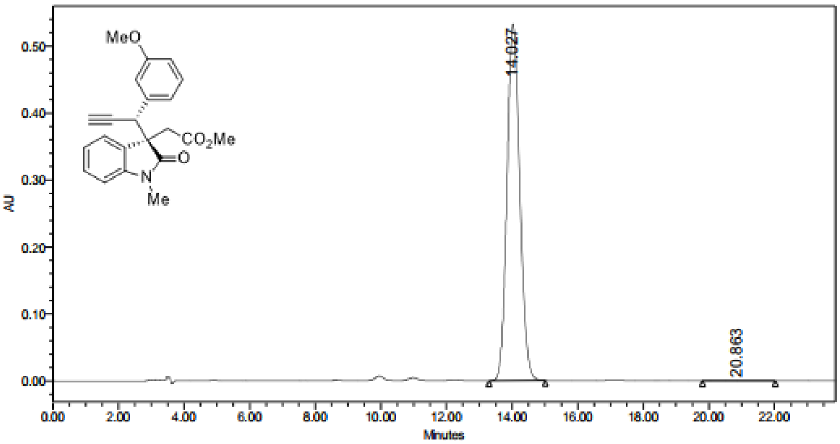

	RT	Area	% Area	Height
1	14.027	14326966	99.86	532379
2	20.863	19388	0.14	321

Supplementary Fig. 73. HPLC spectra of compound **(*R*,*R*)-3ai**

SAMPLE INFORMATION

Sample Name:	wyh-1-78-11-rac-d-IC-30%-1ml/min	Acquired By:	System
Sample Type:	Unknown	Sample Set Name:	
Vial:	24	Acq. Method Set:	30% 1ml
Injection #:	1	Processing Method:	1
Injection Volume:	10.00 ul	Channel Name:	254.0nm
Run Time:	60.0 Minutes	Proc. Chnl. Descr.:	2998 PDA 254.0 nm (2998
Date Acquired:	1/22/2021 6:54:16 PM CST		
Date Processed:	1/22/2021 7:15:27 PM CST		

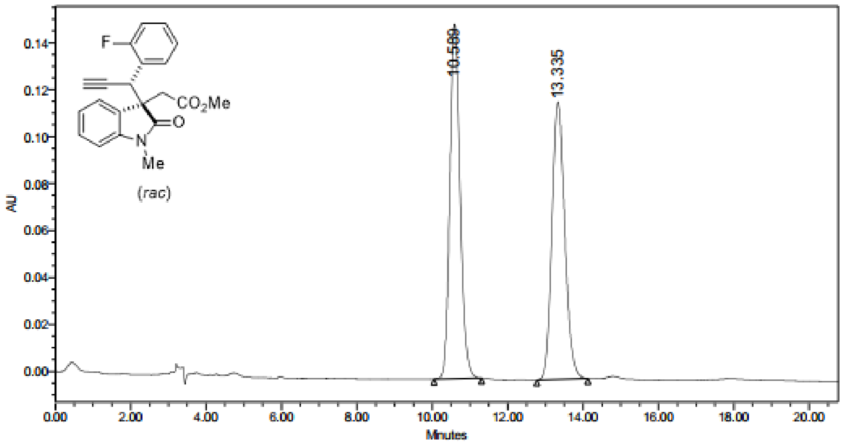

	RT	Area	% Area	Height
1	10.589	2829050	50.36	150997
2	13.335	2788973	49.64	118080

SAMPLE INFORMATION

Sample Name:	wyh-1-78-11-asy-IC-30%-1ml/min	Acquired By:	System
Sample Type:	Unknown	Sample Set Name:	wyh17811asy
Vial:	4	Acq. Method Set:	30% 1ml
Injection #:	1	Processing Method:	1
Injection Volume:	10.00 ul	Channel Name:	254.0nm
Run Time:	28.0 Minutes	Proc. Chnl. Descr.:	2998 PDA 254.0 nm (2998
Date Acquired:	1/22/2021 11:51:48 AM CST		
Date Processed:	1/22/2021 7:15:48 PM CST		

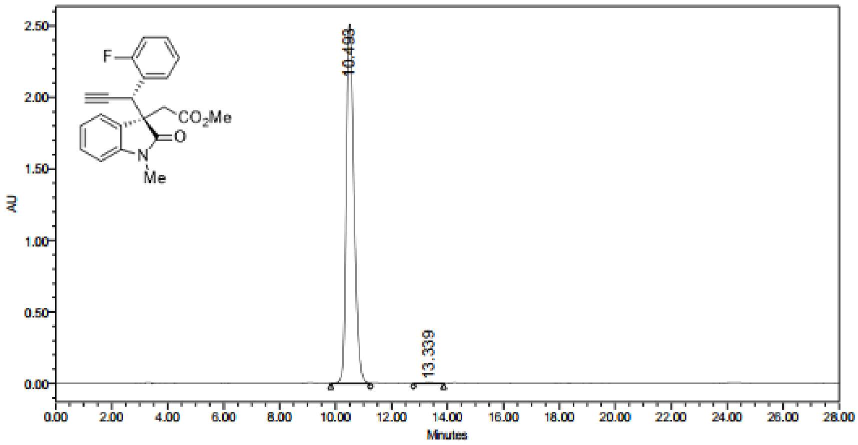

	RT	Area	% Area	Height
1	10.493	48496476	99.76	2511254
2	13.339	117048	0.24	4801

Supplementary Fig. 74. HPLC spectra of compound **(*R*,*S*)-3aj**

SAMPLE INFORMATION

Sample Name:		Acquired By:	System
Sample Type:	Unknown	Sample Set Name	wyh1787racdown
Vial:	51	Acq. Method Set:	10% 1ml
Injection #:	1	Processing Method	1
Injection Volume:	10.00 ul	Channel Name:	254.0nm
Run Time:	60.0 Minutes	Proc. Chnl. Descr.:	2998 PDA 254.0 nm (2998
Date Acquired:	1/17/2021 7:01:54 PM CST		
Date Processed:	1/17/2021 7:39:47 PM CST		

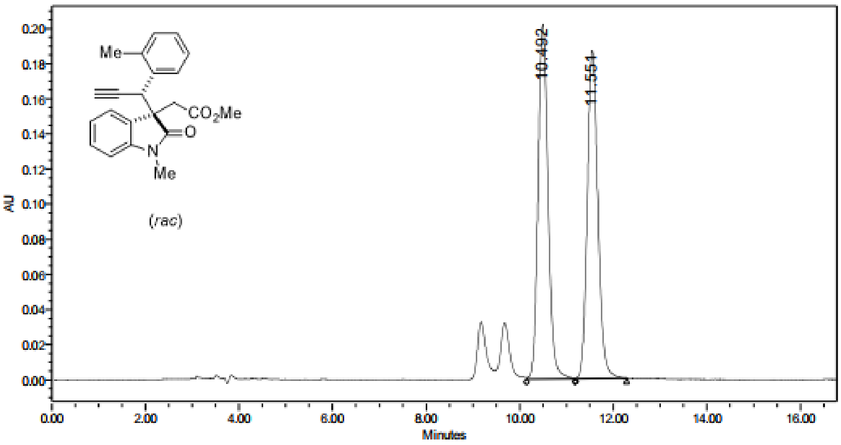

	RT	Area	% Area	Height
1	10.492	2942377	50.14	201948
2	11.551	2926496	49.86	186821

SAMPLE INFORMATION

Sample Name:	wyh-1-78-7-asy-IA-10%-1ml/min	Acquired By:	System
Sample Type:	Unknown	Sample Set Name	wyh1787
Vial:	52	Acq. Method Set:	10% 1ml
Injection #:	1	Processing Method	1
Injection Volume:	10.00 ul	Channel Name:	254.0nm
Run Time:	15.0 Minutes	Proc. Chnl. Descr.:	2998 PDA 254.0 nm (2998
Date Acquired:	1/17/2021 7:20:58 PM CST		
Date Processed:	1/17/2021 7:39:57 PM CST		

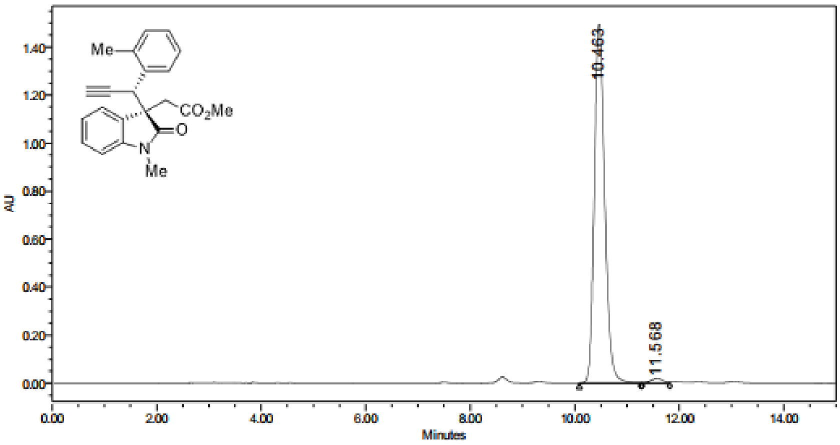

	RT	Area	% Area	Height
1	10.463	20680670	98.49	1494485
2	11.568	316564	1.51	19925

Supplementary Fig.75. HPLC spectra of compound **(*R*,*R*)-3ak**

SAMPLE INFORMATION

Sample Name:	wyh-1-78-12-rac-d-IA-30%-1ml/min	Acquired By:	System
Sample Type:	Unknown	Sample Set Name:	
Vial:	68	Acq. Method Set:	30% 1ml
Injection #:	1	Processing Method:	1
Injection Volume:	10.00 ul	Channel Name:	230.0nm
Run Time:	30.0 Minutes	Proc. Chnl. Descr.:	2998 PDA 230.0 nm (2998
Date Acquired:	1/24/2021 8:44:31 PM CST		
Date Processed:	1/24/2021 8:55:51 PM CST		

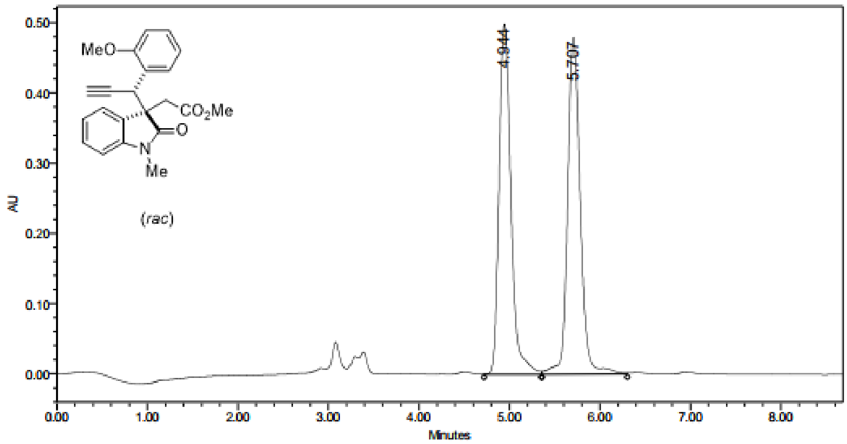

	RT	Area	% Area	Height
1	4.944	4541874	49.33	498260
2	5.707	4665923	50.67	479562

SAMPLE INFORMATION

Sample Name:	wyh-1-78-12-asy-IA-30%-1ml/min	Acquired By:	System
Sample Type:	Unknown	Sample Set Name:	wyh17812
Vial:	60	Acq. Method Set:	30% 1ml
Injection #:	1	Processing Method:	1
Injection Volume:	10.00 ul	Channel Name:	254.0nm
Run Time:	8.0 Minutes	Proc. Chnl. Descr.:	2998 PDA 254.0 nm (2998
Date Acquired:	1/25/2021 4:31:58 PM CST		
Date Processed:	1/25/2021 4:43:43 PM CST		

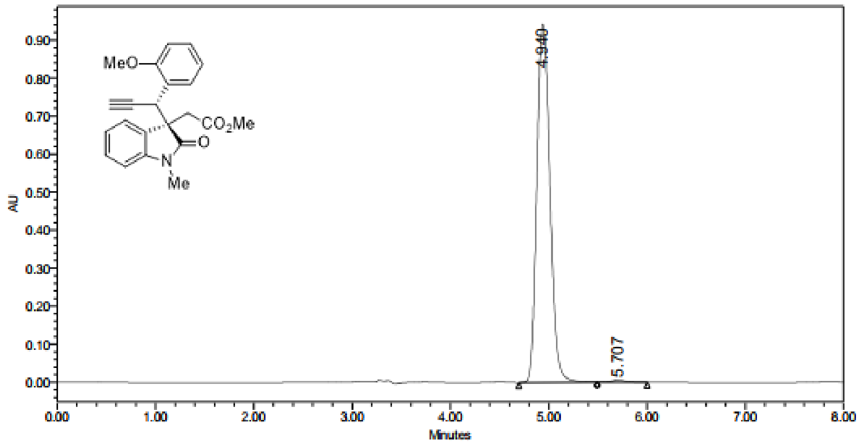

	RT	Area	% Area	Height
1	4.940	8726516	99.29	942020
2	5.707	62633	0.71	5387

Supplementary Fig.76. HPLC spectra of compound **(*R*,*R*)-3al**

SAMPLE INFORMATION

Sample Name:		Acquired By:	System
Sample Type:	Unknown	Sample Set Name	wyhsgygt
Vial:	9	Acq. Method Set:	30% 1ml
Injection #:	1	Processing Method	1
Injection Volume:	10.00 ul	Channel Name:	254.0nm
Run Time:	27.0 Minutes	Proc. Chnl. Descr.:	2998 PDA 254.0 nm (2998
Date Acquired:	3/6/2021 12:59:18 AM CST		
Date Processed:	3/6/2021 8:54:21 AM CST		

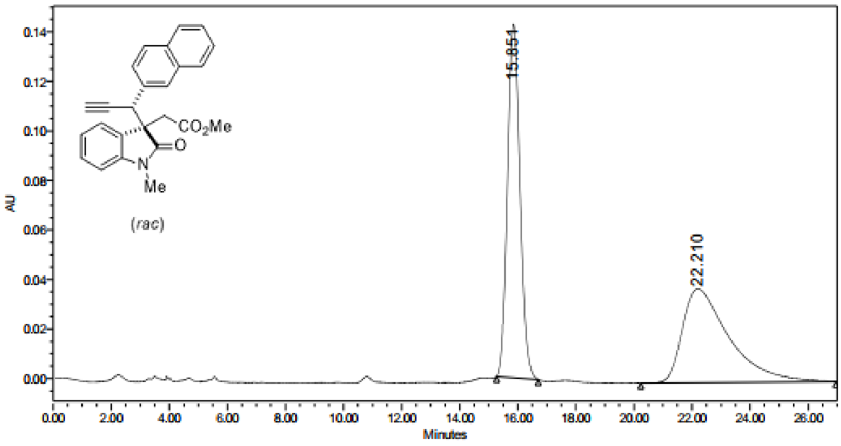

	RT	Area	% Area	Height
1	15.851	4293469	50.51	142803
2	22.210	4206939	49.49	37815

SAMPLE INFORMATION

Sample Name:	wyh-1-78-13-asy-IG-30%-1ml/min	Acquired By:	System
Sample Type:	Unknown	Sample Set Name	wyhsgygt
Vial:	10	Acq. Method Set:	30% 1ml
Injection #:	1	Processing Method	1
Injection Volume:	10.00 ul	Channel Name:	254.0nm
Run Time:	27.0 Minutes	Proc. Chnl. Descr.:	2998 PDA 254.0 nm (2998
Date Acquired:	3/6/2021 1:26:58 AM CST		
Date Processed:	3/6/2021 9:00:32 AM CST		

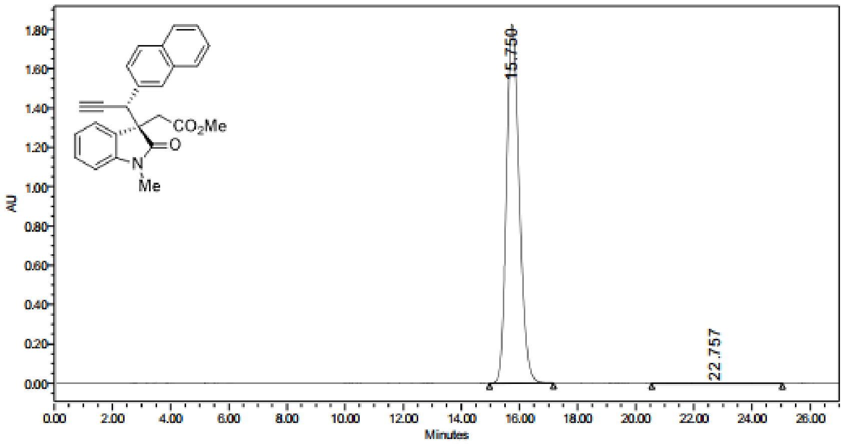

	RT	Area	% Area	Height
1	15.750	55916784	99.73	1825723
2	22.757	153174	0.27	1338

Supplementary Fig.77. HPLC spectra of compound **(*R*,*R*)-3am**

SAMPLE INFORMATION			
Sample Name:		Acquired By:	System
Sample Type:	Unknown	Sample Set Name	wyhsgygt
Vial:	9	Acq. Method Set:	30% 1ml
Injection #:	1	Processing Method	1
Injection Volume:	10.00 ul	Channel Name:	254.0nm
Run Time:	27.0 Minutes	Proc. Chnl. Descr.:	2998 PDA 254.0 nm (2998
Date Acquired:	3/6/2021 12:59:18 AM CST		
Date Processed:	3/6/2021 8:54:21 AM CST		

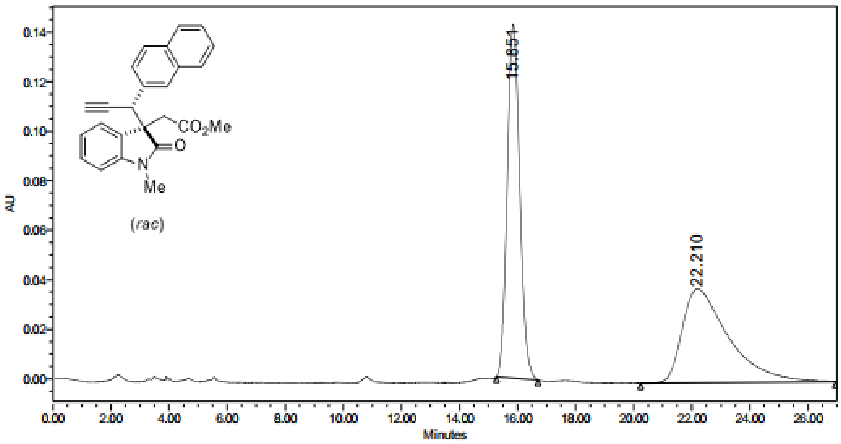

	RT	Area	% Area	Height
1	15.851	4293469	50.51	142803
2	22.210	4206939	49.49	37815

SAMPLE INFORMATION			
Sample Name:	wyh-1-81-2-asy-IG-30%-1ml/min	Acquired By:	System
Sample Type:	Unknown	Sample Set Name	wyhsgygt
Vial:	11	Acq. Method Set:	30% 1ml
Injection #:	1	Processing Method	1
Injection Volume:	10.00 ul	Channel Name:	254.0nm
Run Time:	27.0 Minutes	Proc. Chnl. Descr.:	2998 PDA 254.0 nm (2998
Date Acquired:	3/6/2021 1:54:38 AM CST		
Date Processed:	3/6/2021 9:00:16 AM CST		

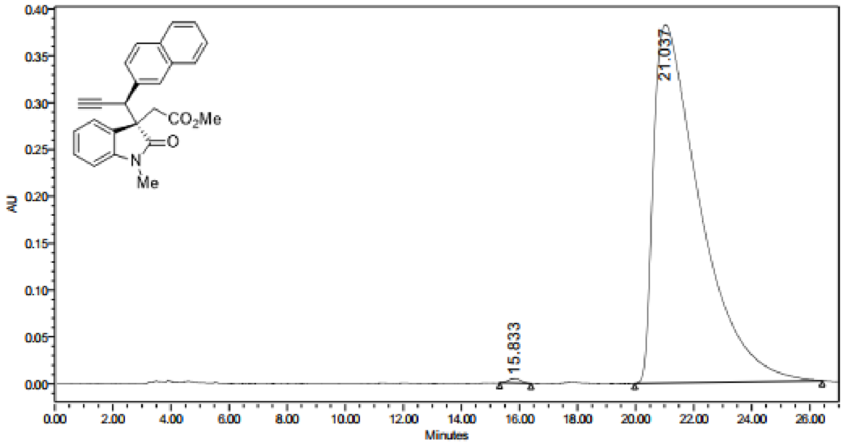

	RT	Area	% Area	Height
1	15.833	149575	0.35	5216
2	21.037	42630567	99.65	382487

Supplementary Fig. 78. HPLC spectra of compound **(*S*,*S*)-3am**

SAMPLE INFORMATION

Sample Name:		Acquired By:	System
Sample Type:	Unknown	Sample Set Name	wyh1797
Vial:	6	Acq. Method Set:	30% 1ml
Injection #:	1	Processing Method	1
Injection Volume:	10.00 ul	Channel Name:	254.0nm
Run Time:	16.0 Minutes	Proc. Chnl. Descr.:	2998 PDA 254.0 nm (2998
Date Acquired:	3/6/2021 10:18:15 AM CST		
Date Processed:	3/6/2021 11:10:53 AM CST		

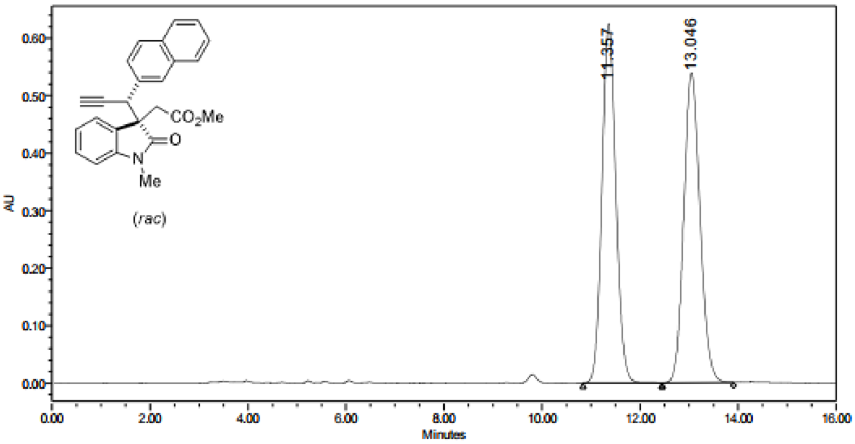

	RT	Area	% Area	Height
1	11.357	12358270	49.81	624155
2	13.046	12453815	50.19	538474

SAMPLE INFORMATION

Sample Name:	wyh-1-79-7-asy-IG-30%-1ml/min	Acquired By:	System
Sample Type:	Unknown	Sample Set Name	wyh1797asy
Vial:	7	Acq. Method Set:	30% 1ml
Injection #:	1	Processing Method	1
Injection Volume:	10.00 ul	Channel Name:	254.0nm
Run Time:	16.0 Minutes	Proc. Chnl. Descr.:	2998 PDA 254.0 nm (2998
Date Acquired:	3/6/2021 11:20:24 AM CST		
Date Processed:	3/6/2021 11:37:21 AM CST		

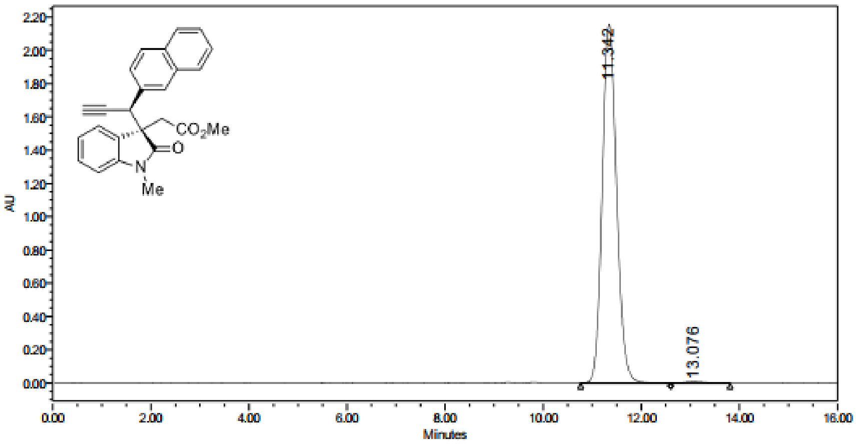

	RT	Area	% Area	Height
1	11.342	44389796	99.54	2156068
2	13.076	204593	0.46	7815

Supplementary Fig. 79. HPLC spectra of compound **(*R*,*S*)-3am**

SAMPLE INFORMATION

Sample Name:		Acquired By:	System
Sample Type:	Unknown	Sample Set Name	wyh1797
Vial:	6	Acq. Method Set:	30% 1ml
Injection #:	1	Processing Method	1
Injection Volume:	10.00 ul	Channel Name:	254.0nm
Run Time:	16.0 Minutes	Proc. Chnl. Descr.:	2998 PDA 254.0 nm (2998
Date Acquired:	3/6/2021 10:18:15 AM CST		
Date Processed:	3/6/2021 11:10:53 AM CST		

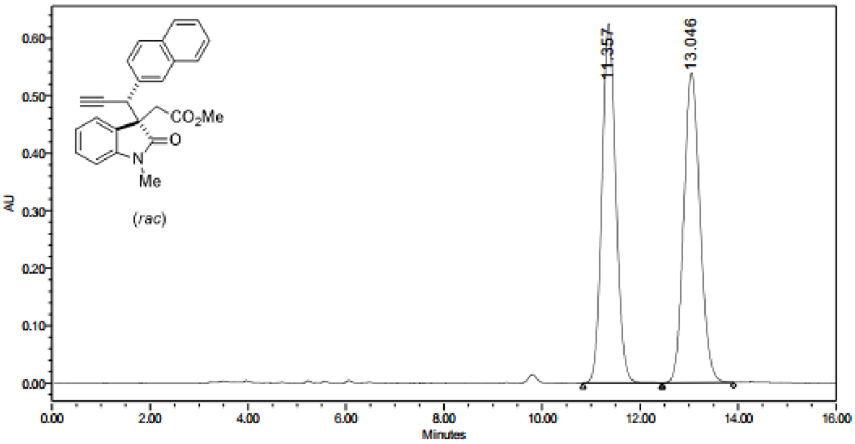

	RT	Area	% Area	Height
1	11.357	12358270	49.81	624155
2	13.046	12453815	50.19	538474

SAMPLE INFORMATION

Sample Name:	wyh-1-81-1-asy-IG-30%-1ml/min	Acquired By:	System
Sample Type:	Unknown	Sample Set Name	wyh1797
Vial:	8	Acq. Method Set:	30% 1ml
Injection #:	1	Processing Method	1
Injection Volume:	10.00 ul	Channel Name:	254.0nm@1
Run Time:	16.0 Minutes	Proc. Chnl. Descr.:	2998 PDA 254.0 nm (2998
Date Acquired:	3/6/2021 10:51:34 AM CST		
Date Processed:	3/6/2021 11:10:41 AM CST		

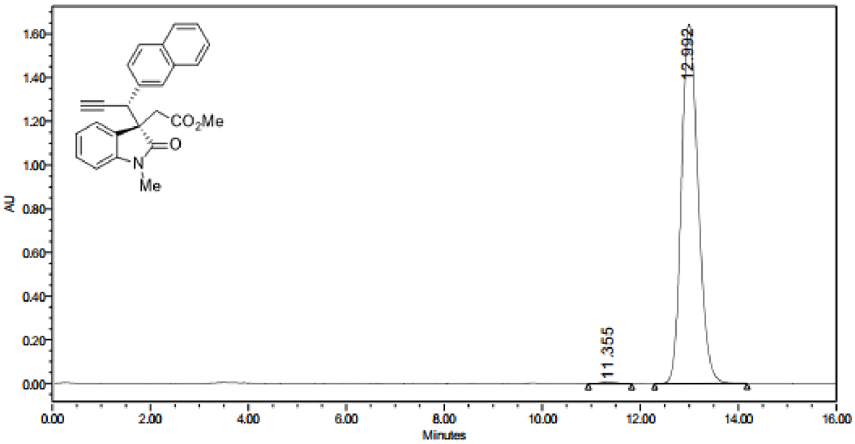

	RT	Area	% Area	Height
1	11.355	167580	0.43	8336
2	12.992	38868789	99.57	1644916

Supplementary Fig. 80. HPLC spectra of compound **(*S*,*R*)-3am**

SAMPLE INFORMATION

Sample Name:	wyh-1-78-8-rac-d-IG-30%-1ml/min	Acquired By:	System
Sample Type:	Unknown	Sample Set Name:	wyh1788racdown
Vial:	112	Acq. Method Set:	30% 1ml
Injection #:	1	Processing Method:	1
Injection Volume:	10.00 ul	Channel Name:	254.0nm
Run Time:	60.0 Minutes	Proc. Chnl. Descr.:	2998 PDA 254.0 nm (2998
Date Acquired:	1/16/2021 9:35:08 PM CST		
Date Processed:	1/16/2021 10:07:39 PM CST		

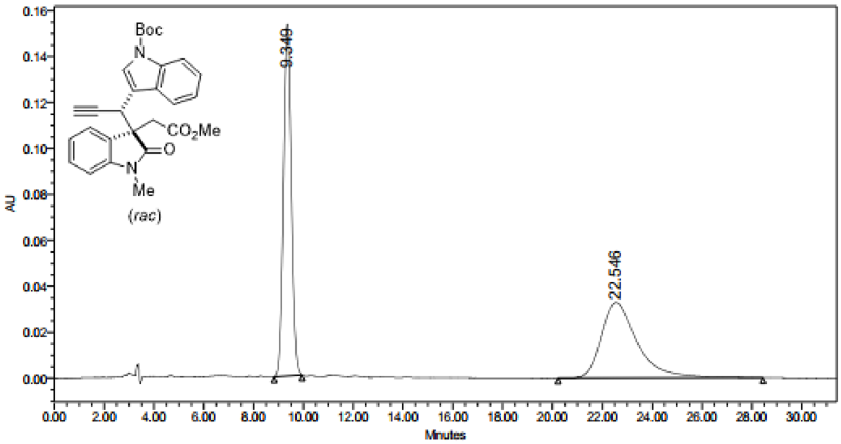

	RT	Area	% Area	Height
1	9.349	3357364	50.65	152974
2	22.546	3271077	49.35	32767

SAMPLE INFORMATION

Sample Name:	wyh-1-78-8-asy-IG-30%-1ml/min	Acquired By:	System
Sample Type:	Unknown	Sample Set Name:	
Vial:	113	Acq. Method Set:	30% 1ml
Injection #:	1	Processing Method:	1
Injection Volume:	10.00 ul	Channel Name:	254.0nm
Run Time:	31.0 Minutes	Proc. Chnl. Descr.:	2998 PDA 254.0 nm (2998
Date Acquired:	1/16/2021 10:07:49 PM CST		
Date Processed:	1/16/2021 10:42:50 PM CST		

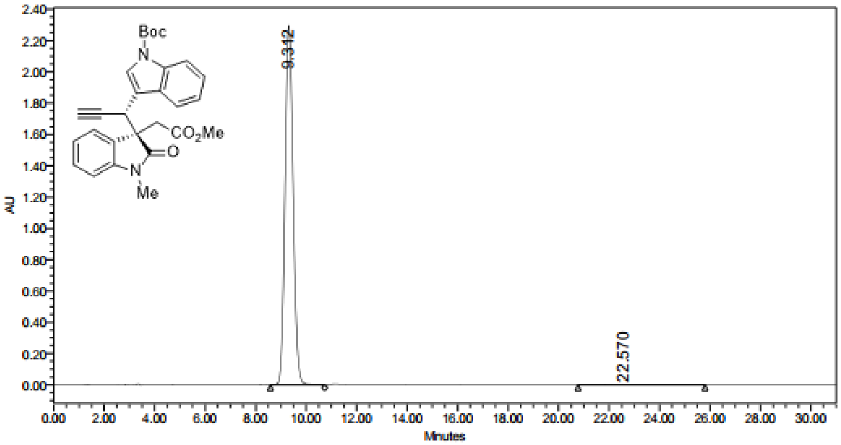

	RT	Area	% Area	Height
1	9.312	51242671	99.54	2295437
2	22.570	237176	0.46	2396

Supplementary Fig. 81. HPLC spectra of compound **(*R*,*R*)-3an**

SAMPLE INFORMATION

Sample Name:	wyh-1-167-2-rac-IE-30%-1ml/min	Acquired By:	System
Sample Type:	Unknown	Sample Set Name	wyh11672
Vial:	61	Acq. Method Set:	30% 1ml
Injection #:	1	Processing Method	1
Injection Volume:	10.00 ul	Channel Name:	254.0nm
Run Time:	14.0 Minutes	Proc. Chnl. Descr.:	2998 PDA 254.0 nm (2998
Date Acquired:	7/13/2021 5:11:20 PM CST		
Date Processed:	7/13/2021 7:38:39 PM CST		

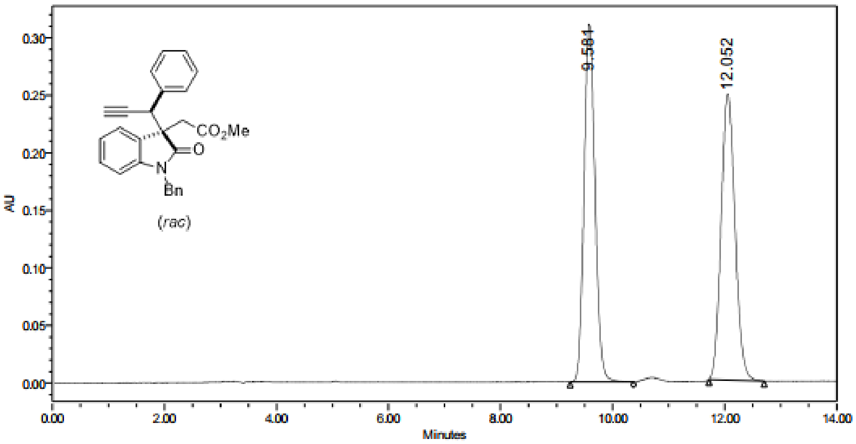

	RT	Area	% Area	Height
1	9.581	4188053	49.66	310632
2	12.052	4245610	50.34	248490

SAMPLE INFORMATION

Sample Name:	wyh-1-167-2-asy-IE-30%-1ml/min	Acquired By:	System
Sample Type:	Unknown	Sample Set Name	wyh11672
Vial:	62	Acq. Method Set:	30% 1ml
Injection #:	1	Processing Method	1
Injection Volume:	10.00 ul	Channel Name:	254.0nm
Run Time:	14.0 Minutes	Proc. Chnl. Descr.:	2998 PDA 254.0 nm (2998
Date Acquired:	7/13/2021 5:26:15 PM CST		
Date Processed:	7/13/2021 7:39:57 PM CST		

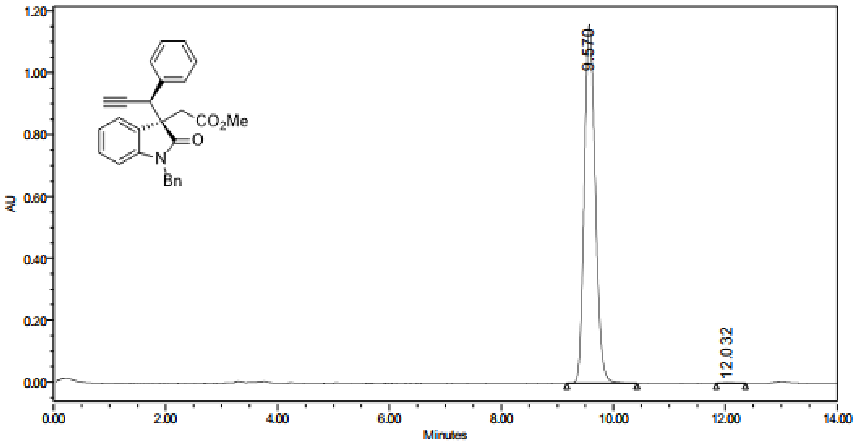

	RT	Area	% Area	Height
1	9.570	15667461	99.91	1159870
2	12.032	14575	0.09	942

Supplementary Fig. 82. HPLC spectra of compound **(*R*,*S*)-3ca**

SAMPLE INFORMATION

Sample Name:	wyh-1-79-11-rac-u-IG-10%-1ml/min	Acquired By:	System
Sample Type:	Unknown	Sample Set Name:	wyh17911
Vial:	1	Acq. Method Set:	10% 1ml
Injection #:	1	Processing Method:	1
Injection Volume:	10.00 ul	Channel Name:	254.0nm
Run Time:	30.0 Minutes	Proc. Chnl. Descr.:	2998 PDA 254.0 nm (2998
Date Acquired:	3/4/2021 5:45:05 PM CST		
Date Processed:	3/4/2021 7:19:15 PM CST		

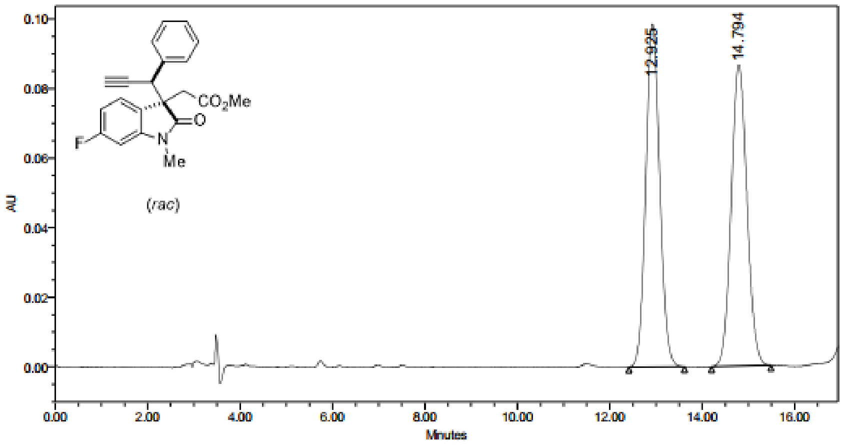

	RT	Area	% Area	Height
1	12.925	2070808	50.19	98570
2	14.794	2054934	49.81	86600

SAMPLE INFORMATION

Sample Name:	wyh-1-79-11-asy-IG-10%-1ml/min	Acquired By:	System
Sample Type:	Unknown	Sample Set Name:	wyh17911asy
Vial:	2	Acq. Method Set:	10% 1ml
Injection #:	1	Processing Method:	1
Injection Volume:	10.00 ul	Channel Name:	254.0nm
Run Time:	18.0 Minutes	Proc. Chnl. Descr.:	2998 PDA 254.0 nm (2998
Date Acquired:	3/4/2021 6:14:47 PM CST		
Date Processed:	3/4/2021 7:19:00 PM CST		

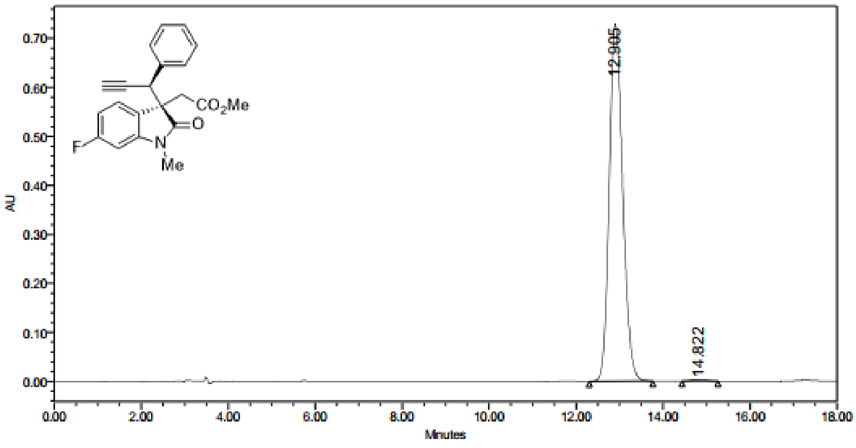

	RT	Area	% Area	Height
1	12.905	15538888	99.57	729031
2	14.822	67286	0.43	3007

Supplementary Fig. 83. HPLC spectra of compound **(*R*,*S*)-3ka**

SAMPLE INFORMATION

Sample Name:	wyh-1-79-5-rac-u-IG-30%-1ml/min	Acquired By:	System
Sample Type:	Unknown	Sample Set Name:	
Vial:	49	Acq. Method Set:	30% 1ml
Injection #:	1	Processing Method:	1
Injection Volume:	10.00 ul	Channel Name:	254.0nm
Run Time:	30.0 Minutes	Proc. Chnl. Descr.:	2998 PDA 254.0 nm (2998
Date Acquired:	2/4/2021 12:54:31 PM CST		
Date Processed:	2/4/2021 1:05:26 PM CST		

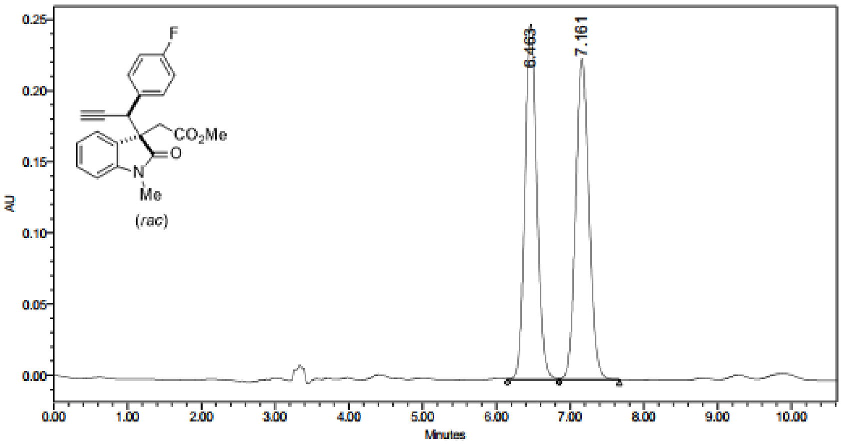

	RT	Area	% Area	Height
1	6.463	2844874	50.07	249568
2	7.161	2836586	49.93	225363

SAMPLE INFORMATION

Sample Name:	wyh-1-79-5-asy-IG-30%-1ml/min	Acquired By:	System
Sample Type:	Unknown	Sample Set Name:	
Vial:	50	Acq. Method Set:	30% 1ml
Injection #:	1	Processing Method:	1
Injection Volume:	10.00 ul	Channel Name:	254.0nm
Run Time:	30.0 Minutes	Proc. Chnl. Descr.:	2998 PDA 254.0 nm (2998
Date Acquired:	2/4/2021 11:40:57 AM CST		
Date Processed:	2/4/2021 1:01:56 PM CST		

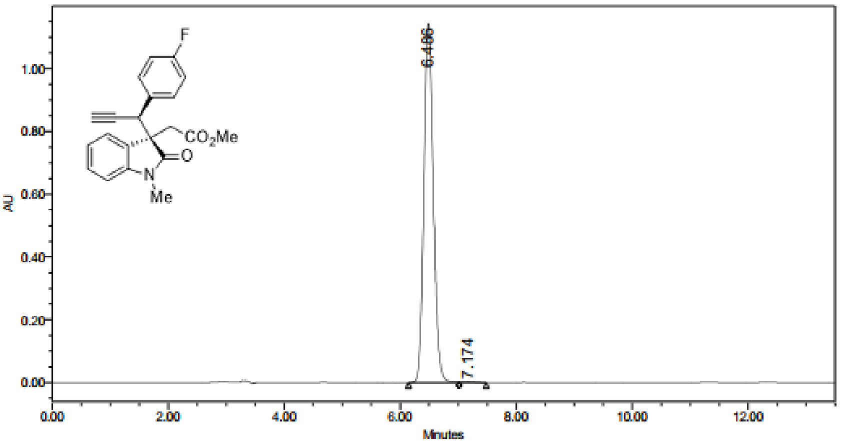

	RT	Area	% Area	Height
1	6.486	12936766	99.71	1141676
2	7.174	37169	0.29	2530

Supplementary Fig. 84. HPLC spectra of compound **(*R*,*S*)-3ab**

SAMPLE INFORMATION

Sample Name:	LS-2-4-1-rac-up-IG-30%-1ml/min	Acquired By:	System
Sample Type:	Unknown	Sample Set Name:	LS_2_4
Vial:	7	Acq. Method Set:	30% 1ml
Injection #:	1	Processing Method:	1
Injection Volume:	10.00 ul	Channel Name:	230.0nm
Run Time:	20.0 Minutes	Proc. Chnl. Descr.:	2998 PDA 230.0 nm (2998
Date Acquired:	3/14/2021 9:17:59 PM CST		
Date Processed:	5/7/2021 5:52:53 PM CST		

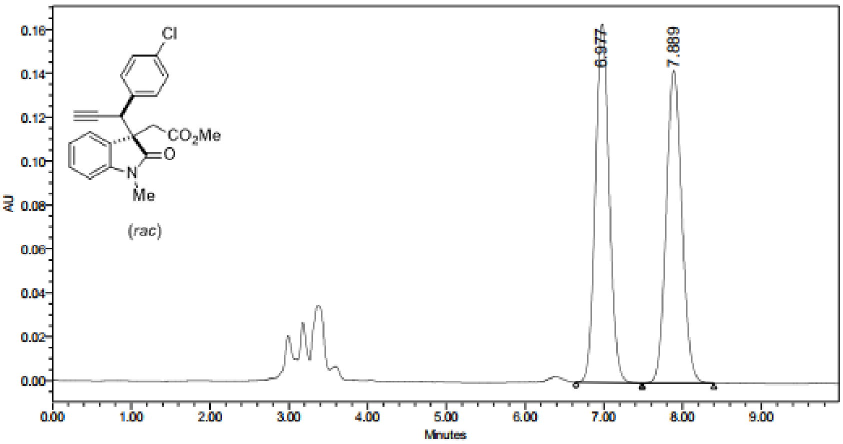

	RT	Area	% Area	Height
1	6.977	1997021	50.28	163296
2	7.889	1974968	49.72	142436

SAMPLE INFORMATION

Sample Name:	LS-2-4-1-asy-IG-30%-1ml/min	Acquired By:	System
Sample Type:	Unknown	Sample Set Name:	LS_2_4
Vial:	8	Acq. Method Set:	30% 1ml
Injection #:	1	Processing Method:	1
Injection Volume:	10.00 ul	Channel Name:	230.0nm
Run Time:	10.0 Minutes	Proc. Chnl. Descr.:	2998 PDA 230.0 nm (2998
Date Acquired:	3/14/2021 9:29:07 PM CST		
Date Processed:	5/7/2021 5:52:42 PM CST		

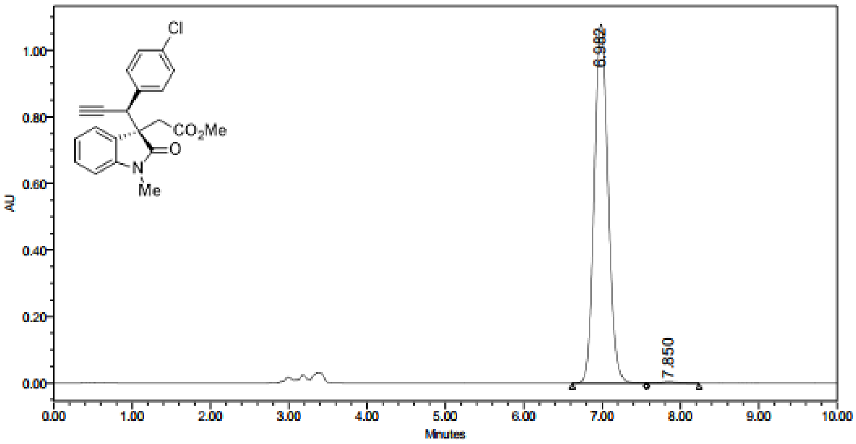

	RT	Area	% Area	Height
1	6.982	13264165	99.41	1079165
2	7.850	78258	0.59	4800

Supplementary Fig. 85. HPLC spectra of compound **(*R*,*S*)-3ac**

SAMPLE INFORMATION

Sample Name:	LS-2-4-2-rac-up-IG-30%-1ml/min	Acquired By:	System
Sample Type:	Unknown	Sample Set Name:	LS24
Vial:	9	Acq. Method Set:	30% 1ml
Injection #:	1	Processing Method:	1
Injection Volume:	10.00 ul	Channel Name:	230.0nm
Run Time:	10.0 Minutes	Proc. Chnl. Descr.:	2998 PDA 230.0 nm (2998
Date Acquired:	3/14/2021 10:15:08 PM CST		
Date Processed:	5/7/2021 5:52:08 PM CST		

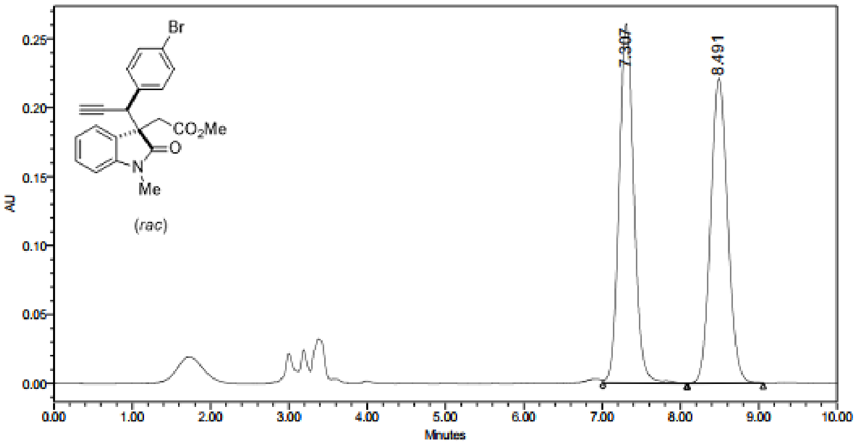

	RT	Area	% Area	Height
1	7.307	3410831	50.49	260433
2	8.491	3344450	49.51	220935

SAMPLE INFORMATION

Sample Name:	LS-2-4-2-asy-IG-30%-1ml/min	Acquired By:	System
Sample Type:	Unknown	Sample Set Name:	LS_2_4
Vial:	10	Acq. Method Set:	30% 1ml
Injection #:	1	Processing Method:	1
Injection Volume:	10.00 ul	Channel Name:	230.0nm
Run Time:	20.0 Minutes	Proc. Chnl. Descr.:	2998 PDA 230.0 nm (2998
Date Acquired:	3/14/2021 10:00:32 PM CST		
Date Processed:	5/7/2021 5:51:54 PM CST		

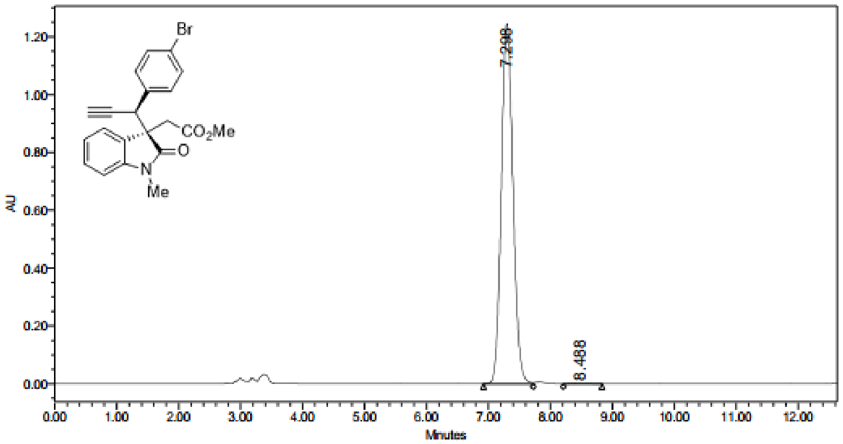

	RT	Area	% Area	Height
1	7.298	16148747	99.76	1243849
2	8.488	38956	0.24	2485

Supplementary Fig. 86. HPLC spectra of compound **(*R*,*S*)-3ad**

SAMPLE INFORMATION			
Sample Name:	wyh-1-79-12-rac-up	Acquired By:	System
Sample Type:	Unknown	Sample Set Name:	wyh17912racup
Vial:	1	Acq. Method Set:	30% 1ml
Injection #:	1	Processing Method:	1
Injection Volume:	10.00 ul	Channel Name:	254.0nm
Run Time:	20.0 Minutes	Proc. Chnl. Descr.:	2998 PDA 254.0 nm (2998
Date Acquired:	3/4/2021 9:56:50 AM CST		
Date Processed:	3/4/2021 10:34:53 AM CST		

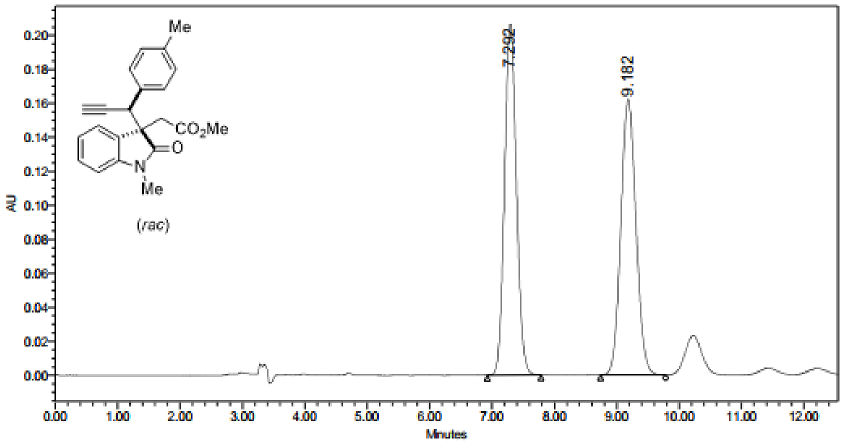

	RT	Area	% Area	Height
1	7.292	2707073	49.92	206422
2	9.182	2715675	50.08	162068

SAMPLE INFORMATION			
Sample Name:	wyh-1-79-12-asy-IG-30%-1ml/min	Acquired By:	System
Sample Type:	Unknown	Sample Set Name:	wyh17912asy
Vial:	2	Acq. Method Set:	30% 1ml
Injection #:	1	Processing Method:	1
Injection Volume:	10.00 ul	Channel Name:	254.0nm
Run Time:	12.0 Minutes	Proc. Chnl. Descr.:	2998 PDA 254.0 nm (2998
Date Acquired:	3/4/2021 10:16:43 AM CST		
Date Processed:	3/4/2021 10:34:27 AM CST		

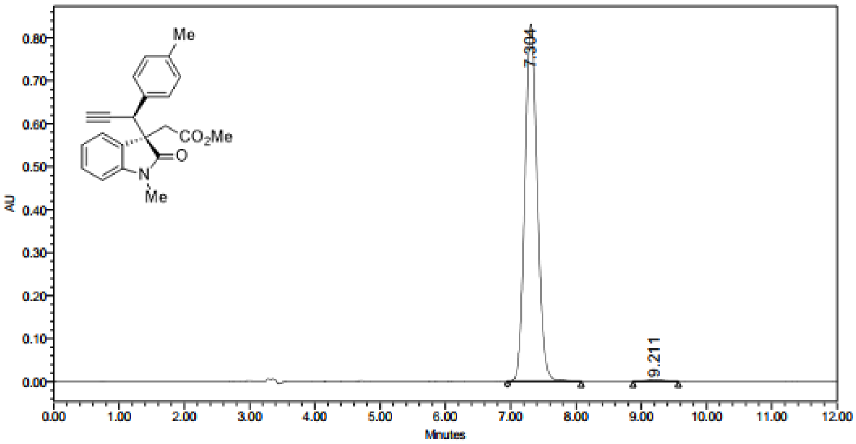

	RT	Area	% Area	Height
1	7.304	11015778	99.47	830903
2	9.211	58365	0.53	3522

Supplementary Fig. 87. HPLC spectra of compound **(*R*,*S*)-3ae**

SAMPLE INFORMATION

Sample Name:	wyh-1-79-6-rac-u-IG-30%-1ml/min	Acquired By:	System
Sample Type:	Unknown	Sample Set Name:	
Vial:	49	Acq. Method Set:	30% 1ml
Injection #:	1	Processing Method:	1
Injection Volume:	10.00 ul	Channel Name:	254.0nm
Run Time:	30.0 Minutes	Proc. Chnl. Descr.:	2998 PDA 254.0 nm (2998
Date Acquired:	2/4/2021 10:41:57 AM CST		
Date Processed:	2/4/2021 11:03:11 AM CST		

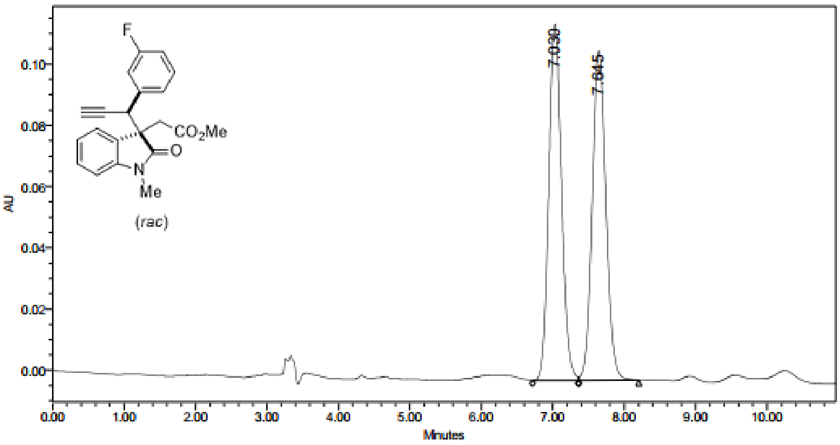

	RT	Area	% Area	Height
1	7.030	1443398	49.98	116353
2	7.645	1444832	50.02	107652

SAMPLE INFORMATION

Sample Name:	wyh-1-79-6-asy-IG-30%-1ml/min	Acquired By:	System
Sample Type:	Unknown	Sample Set Name:	
Vial:	50	Acq. Method Set:	30% 1ml
Injection #:	1	Processing Method:	1
Injection Volume:	10.00 ul	Channel Name:	254.0nm
Run Time:	30.0 Minutes	Proc. Chnl. Descr.:	2998 PDA 254.0 nm (2998
Date Acquired:	2/4/2021 10:53:49 AM CST		
Date Processed:	2/4/2021 11:05:05 AM CST		

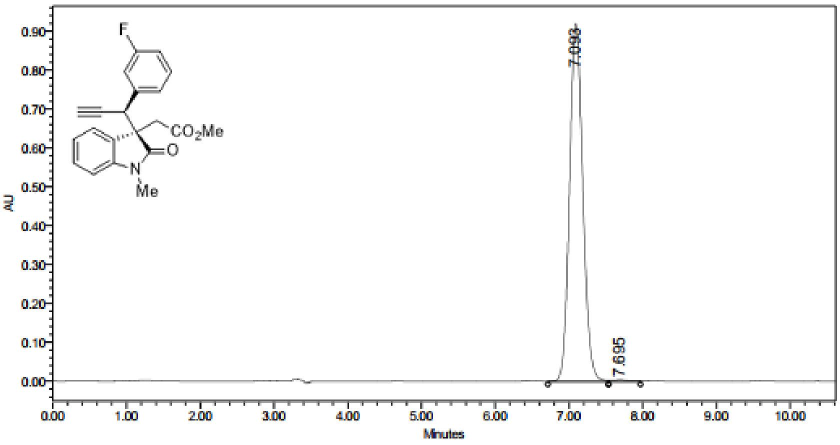

	RT	Area	% Area	Height
1	7.093	11429689	99.50	919256
2	7.695	57547	0.50	3488

Supplementary Fig. 88. HPLC spectra of compound **(*R*,*S*)-3ag**

SAMPLE INFORMATION

Sample Name:	wyh-2-92-2-rac-IG-30%-1ml/min	Acquired By:	System
Sample Type:	Unknown	Sample Set Name:	20211203
Vial:	18	Acq. Method Set:	30% 1ml
Injection #:	1	Processing Method:	1
Injection Volume:	10.00 ul	Channel Name:	254.0nm
Run Time:	18.0 Minutes	Proc. Chnl. Descr.:	2998 PDA 254.0 nm (2998
Date Acquired:	12/3/2021 9:23:30 PM CST		
Date Processed:	12/3/2021 9:46:51 PM CST		

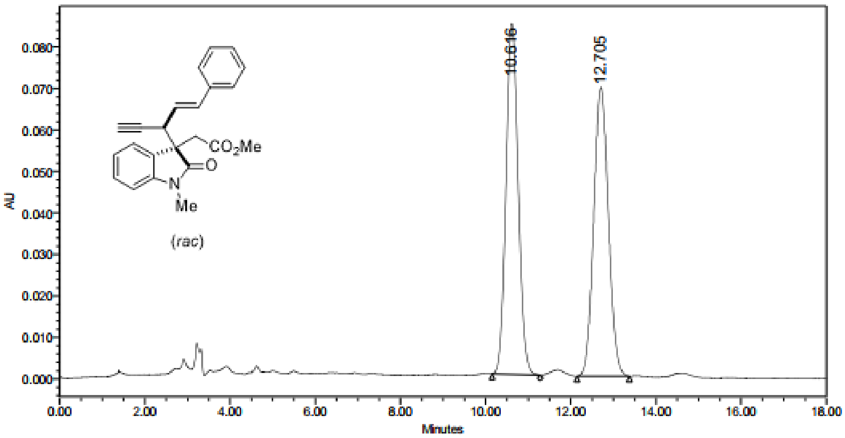

	RT	Area	% Area	Height
1	10.616	1680172	50.19	84449
2	12.705	1667466	49.81	69606

SAMPLE INFORMATION

Sample Name:	wyh-2-87-2-asy-IG-30%-1ml/min	Acquired By:	System
Sample Type:	Unknown	Sample Set Name:	20211203
Vial:	19	Acq. Method Set:	30% 1ml
Injection #:	1	Processing Method:	1
Injection Volume:	10.00 ul	Channel Name:	254.0nm
Run Time:	18.0 Minutes	Proc. Chnl. Descr.:	2998 PDA 254.0 nm (2998
Date Acquired:	12/3/2021 4:57:16 PM CST		
Date Processed:	12/3/2021 9:38:45 PM CST		

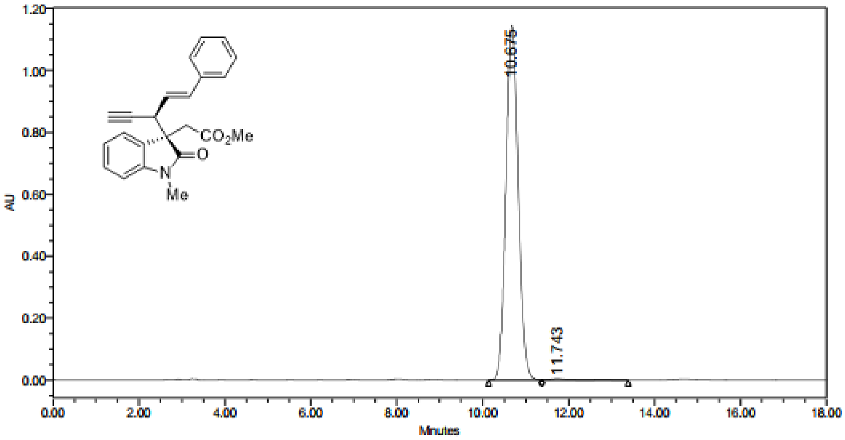

	RT	Area	% Area	Height
1	10.675	22765044	99.45	1145284
2	11.743	125930	0.55	4446

Supplementary Fig. 89. HPLC spectra of compound **(*R*,*R*)-3ao**

SAMPLE INFORMATION

Sample Name:	wyh-2-90-2-rac-IG-30%-1ml/min	Acquired By:	System
Sample Type:	Unknown	Sample Set Name:	wyh20211127
Vial:	11	Acq. Method Set:	30% 1ml
Injection #:	1	Processing Method:	1
Injection Volume:	10.00 ul	Channel Name:	254.0nm
Run Time:	24.0 Minutes	Proc. Chnl. Descr.:	2998 PDA 254.0 nm (2998
Date Acquired:	11/27/2021 8:52:32 PM CST		
Date Processed:	12/3/2021 4:06:04 PM CST		

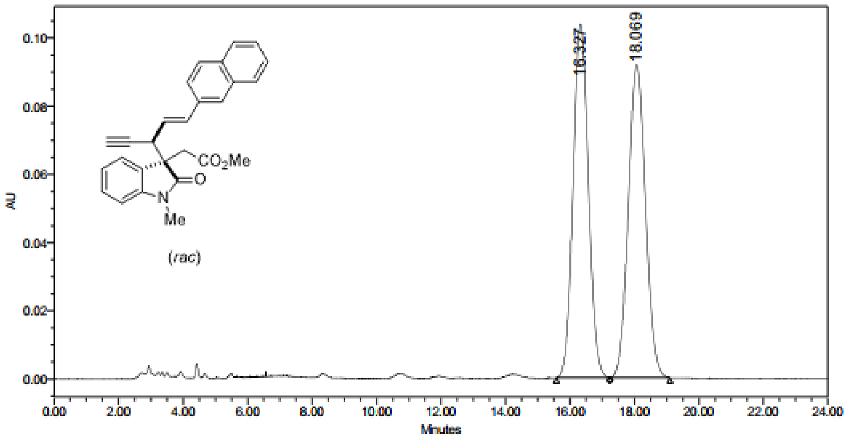

	RT	Area	% Area	Height
1	16.327	3407568	50.01	103664
2	18.069	3406564	49.99	91701

SAMPLE INFORMATION

Sample Name:	wyh-2-90-2-asy-IG-30%-1ml/min	Acquired By:	System
Sample Type:	Unknown	Sample Set Name:	wyh20211127
Vial:	12	Acq. Method Set:	30% 1ml
Injection #:	1	Processing Method:	1
Injection Volume:	10.00 ul	Channel Name:	254.0nm
Run Time:	24.0 Minutes	Proc. Chnl. Descr.:	2998 PDA 254.0 nm (2998
Date Acquired:	11/27/2021 9:17:10 PM CST		
Date Processed:	12/3/2021 4:06:17 PM CST		

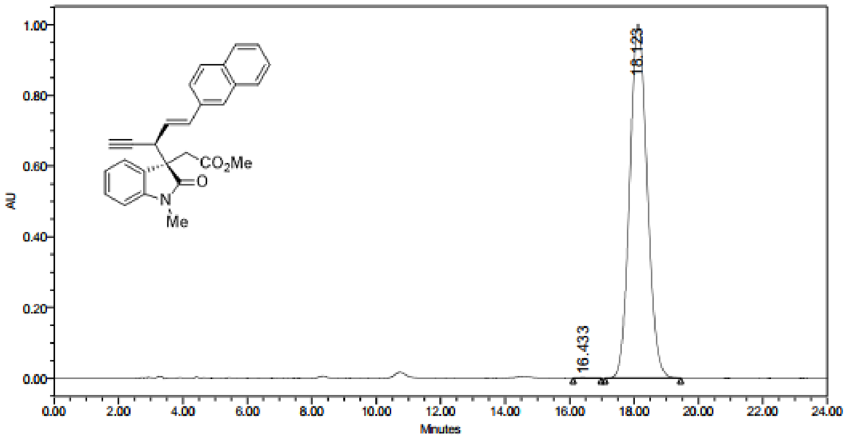

	RT	Area	% Area	Height
1	16.433	19907	0.05	767
2	18.123	37847529	99.95	1001593

Supplementary Fig. 90. HPLC spectra of compound **(*R*,*R*)-3ap**

SAMPLE INFORMATION

Sample Name:	wyh-2-91-1-rac-IG-30%-1ml/min	Acquired By:	System
Sample Type:	Unknown	Sample Set Name:	
Vial:	14	Acq. Method Set:	30% 1ml
Injection #:	2	Processing Method:	1
Injection Volume:	10.00 ul	Channel Name:	254.0nm
Run Time:	60.0 Minutes	Proc. Chnl. Descr.:	2998 PDA 254.0 nm (2998
Date Acquired:	12/3/2021 3:22:42 PM CST		
Date Processed:	12/3/2021 3:56:41 PM CST		

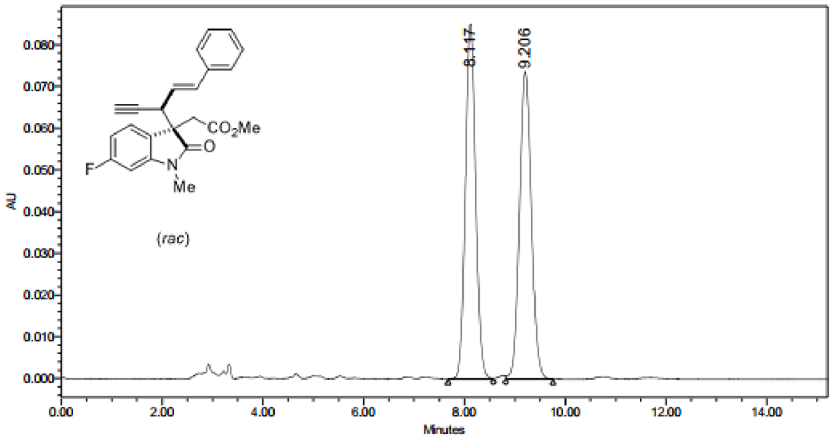

	RT	Area	% Area	Height
1	8.117	1206081	50.03	85093
2	9.206	1204552	49.97	73734

SAMPLE INFORMATION

Sample Name:	wyh-2-91-1-asy-IG-30%-1ml/min	Acquired By:	System
Sample Type:	Unknown	Sample Set Name:	
Vial:	15	Acq. Method Set:	30% 1ml
Injection #:	1	Processing Method:	1
Injection Volume:	10.00 ul	Channel Name:	254.0nm
Run Time:	60.0 Minutes	Proc. Chnl. Descr.:	2998 PDA 254.0 nm (2998
Date Acquired:	12/3/2021 3:38:49 PM CST		
Date Processed:	12/3/2021 3:55:47 PM CST		

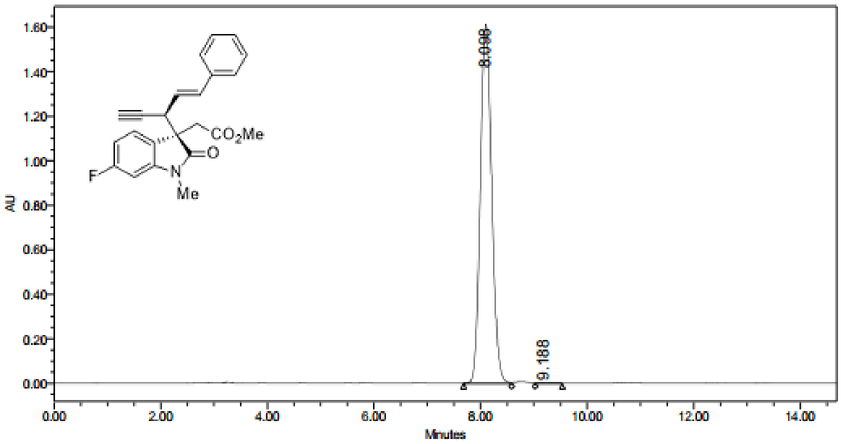

	RT	Area	% Area	Height
1	8.098	23883635	99.84	1612713
2	9.188	39274	0.16	2371

Supplementary Fig. 91. HPLC spectra of compound **(*R*,*R*)-3ko**

SAMPLE INFORMATION

Sample Name:	wyh-2-91-2-rac-IG-30%-1ml/min	Acquired By:	System
Sample Type:	Unknown	Sample Set Name:	
Vial:	16	Acq. Method Set:	30% 1ml
Injection #:	1	Processing Method:	1
Injection Volume:	10.00 ul	Channel Name:	254.0nm
Run Time:	60.0 Minutes	Proc. Chnl. Descr.:	2998 PDA 254.0 nm (2998
Date Acquired:	12/3/2021 3:55:08 PM CST		
Date Processed:	12/3/2021 9:35:22 PM CST		

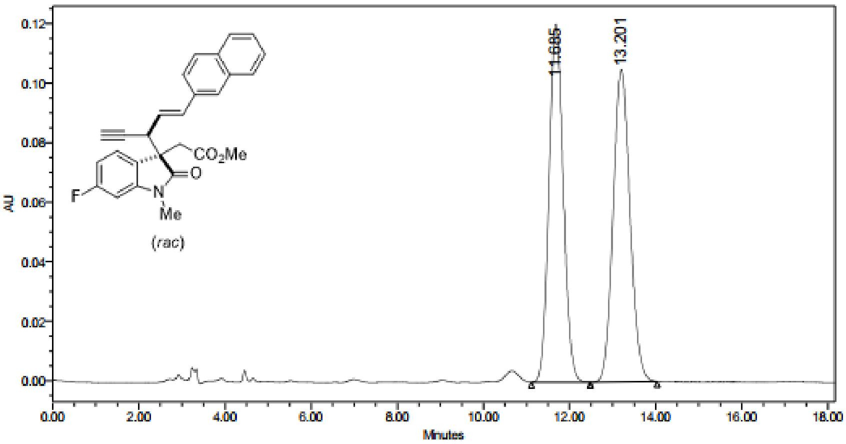

	RT	Area	% Area	Height
1	11.685	2759531	49.22	120278
2	13.201	2846654	50.78	104901

SAMPLE INFORMATION

Sample Name:	wyh-2-91-2-asy-IG-30%-1ml/min	Acquired By:	System
Sample Type:	Unknown	Sample Set Name:	
Vial:	17	Acq. Method Set:	30% 1ml
Injection #:	1	Processing Method:	1
Injection Volume:	10.00 ul	Channel Name:	254.0nm
Run Time:	60.0 Minutes	Proc. Chnl. Descr.:	2998 PDA 254.0 nm (2998
Date Acquired:	12/3/2021 4:14:07 PM CST		
Date Processed:	12/3/2021 9:35:34 PM CST		

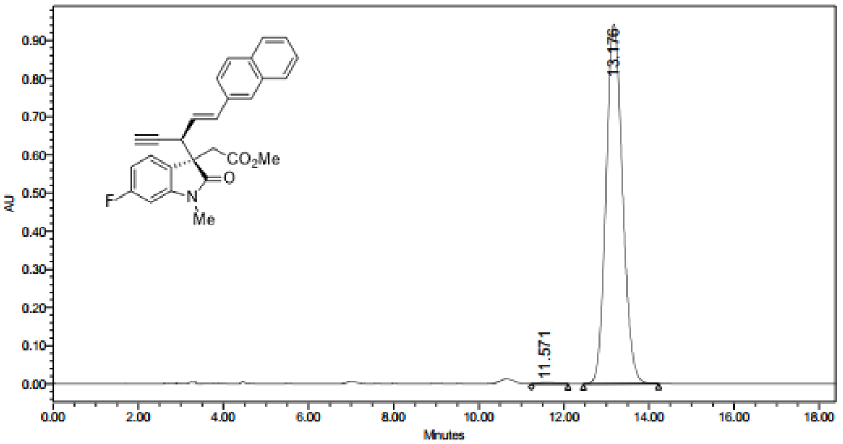

	RT	Area	% Area	Height
1	11.571	71378	0.28	2958
2	13.176	25323224	99.72	942045

Supplementary Fig. 92. HPLC spectra of compound **(*R*,*R*)-3kp**

SAMPLE INFORMATION

Sample Name:	wyh-1-158-rac-IA-30%-1ml/min	Acquired By:	System
Sample Type:	Unknown	Sample Set Name:	wyh158rac
Vial:	22	Acq. Method Set:	30% 1ml
Injection #:	1	Processing Method:	1
Injection Volume:	10.00 ul	Channel Name:	254.0nm
Run Time:	50.0 Minutes	Proc. Chnl. Descr.:	2998 PDA 254.0 nm (2998
Date Acquired:	6/25/2021 4:53:09 PM CST		
Date Processed:	6/27/2021 3:47:20 PM CST		

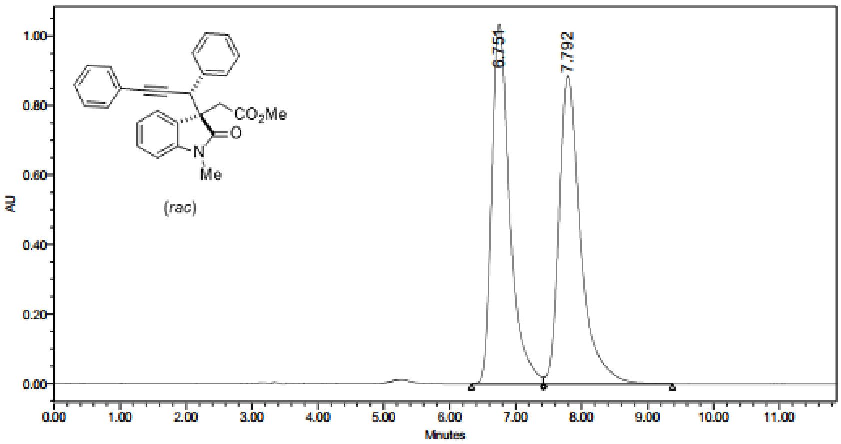

	RT	Area	% Area	Height
1	6.751	19650870	49.55	1033599
2	7.792	20005071	50.45	885851

SAMPLE INFORMATION

Sample Name:	wyh-1-158-asy-IA-30%-1ml/min	Acquired By:	System
Sample Type:	Unknown	Sample Set Name:	111
Vial:	23	Acq. Method Set:	30% 1ml
Injection #:	1	Processing Method:	1
Injection Volume:	10.00 ul	Channel Name:	254.0nm
Run Time:	12.0 Minutes	Proc. Chnl. Descr.:	2998 PDA 254.0 nm (2998
Date Acquired:	6/25/2021 5:06:46 PM CST		
Date Processed:	6/27/2021 3:47:35 PM CST		

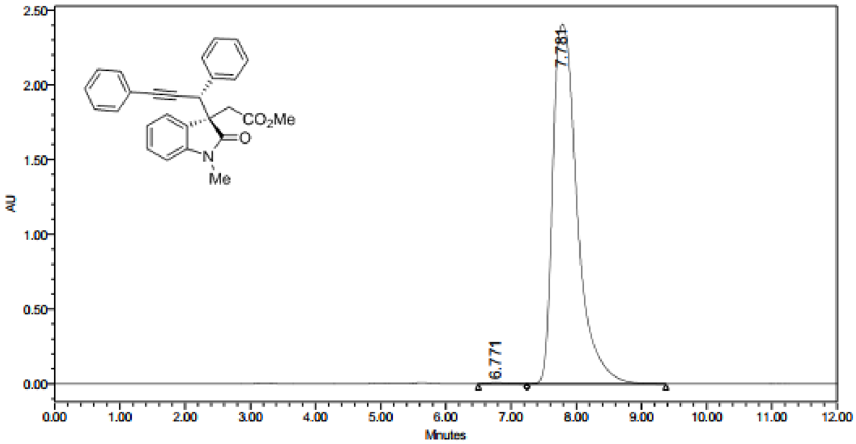

	RT	Area	% Area	Height
1	6.771	123337	0.20	6518
2	7.781	62173725	99.80	2404716

Supplementary Fig. 93. HPLC spectra of compound **5**

SAMPLE INFORMATION

Sample Name:	wyh-1-159-rac-IG-30%-1ml/min	Acquired By:	System
Sample Type:	Unknown	Sample Set Name:	wyh1159rac
Vial:	25	Acq. Method Set:	30% 1ml
Injection #:	1	Processing Method:	1
Injection Volume:	10.00 ul	Channel Name:	254.0nm@1
Run Time:	30.0 Minutes	Proc. Chnl. Descr.:	2998 PDA 254.0 nm (2998
Date Acquired:	6/27/2021 3:16:43 PM CST		
Date Processed:	6/27/2021 3:54:33 PM CST		

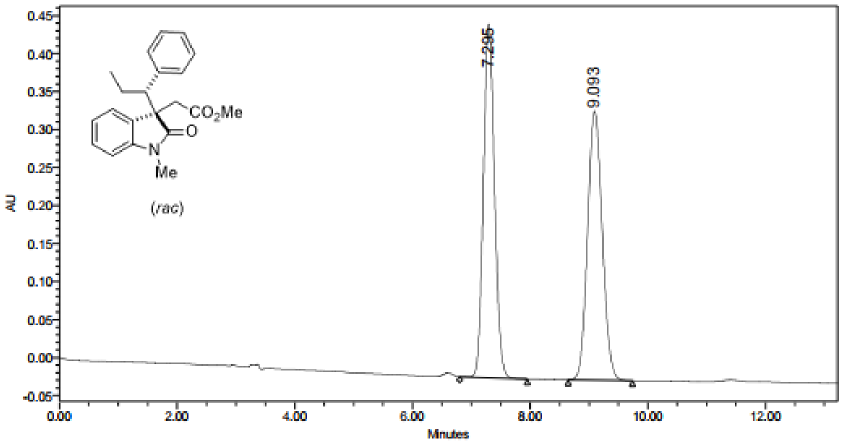

	RT	Area	% Area	Height
1	7.295	6158366	50.09	465846
2	9.093	6136651	49.91	354046

SAMPLE INFORMATION

Sample Name:	wyh-1-159-asy-IG-30%-1ml/min	Acquired By:	System
Sample Type:	Unknown	Sample Set Name:	wyh1159asy
Vial:	26	Acq. Method Set:	30% 1ml
Injection #:	1	Processing Method:	1
Injection Volume:	10.00 ul	Channel Name:	254.0nm
Run Time:	13.0 Minutes	Proc. Chnl. Descr.:	2998 PDA 254.0 nm (2998
Date Acquired:	6/27/2021 3:31:27 PM CST		
Date Processed:	6/27/2021 3:54:56 PM CST		

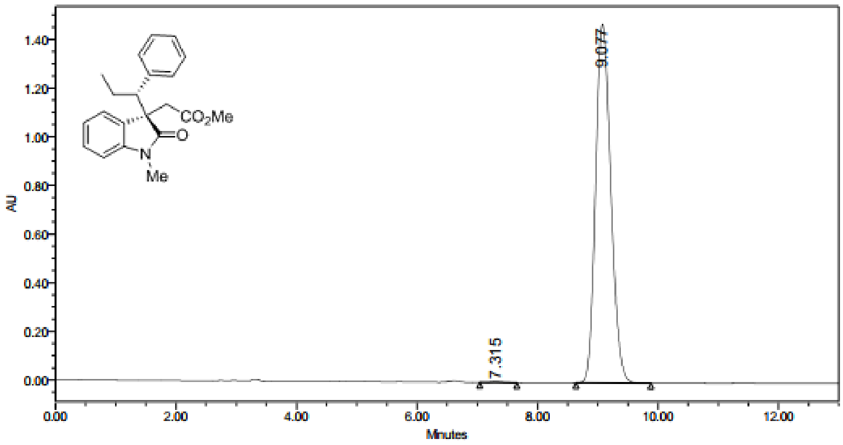

	RT	Area	% Area	Height
1	7.315	49860	0.19	3788
2	9.077	26054486	99.81	1473643

Supplementary Fig. 94. HPLC spectra of compound **6**

SAMPLE INFORMATION

Sample Name:	wyh-1-157-rac-IA-30%-1ml/min	Acquired By:	System
Sample Type:	Unknown	Sample Set Name	wyh1157rac
Vial:	21	Acq. Method Set:	30% 1ml
Injection #:	1	Processing Method	1
Injection Volume:	10.00 ul	Channel Name:	254.0nm
Run Time:	25.0 Minutes	Proc. Chnl. Descr.:	2998 PDA 254.0 nm (2998
Date Acquired:	7/13/2021 9:48:40 PM CST		
Date Processed:	7/14/2021 9:33:13 AM CST		

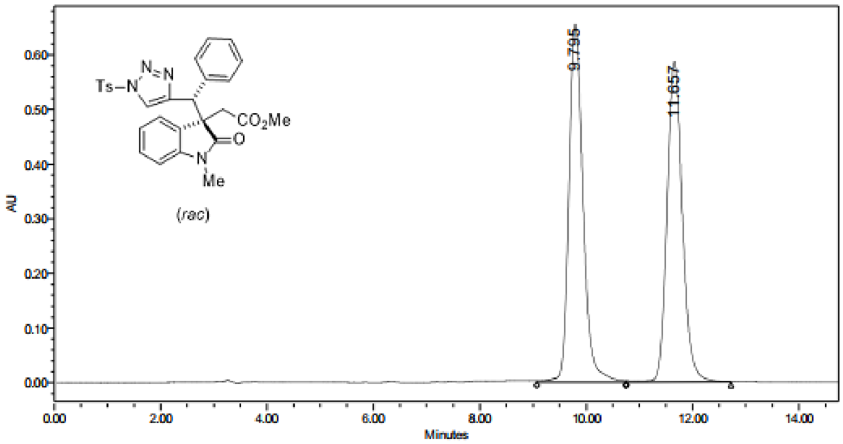

	RT	Area	% Area	Height
1	9.795	12140674	50.79	655366
2	11.657	11764158	49.21	586102

SAMPLE INFORMATION

Sample Name:	wyh-1-157-asy-IA-30%-1ml/min	Acquired By:	System
Sample Type:	Unknown	Sample Set Name:	wyh157asy
Vial:	19	Acq. Method Set:	30% 1ml
Injection #:	1	Processing Method:	1
Injection Volume:	10.00 ul	Channel Name:	230.0nm
Run Time:	16.0 Minutes	Proc. Chnl. Descr.:	2998 PDA 230.0 nm (2998
Date Acquired:	6/25/2021 10:18:48 PM CST		
Date Processed:	7/6/2021 9:50:16 AM CST		

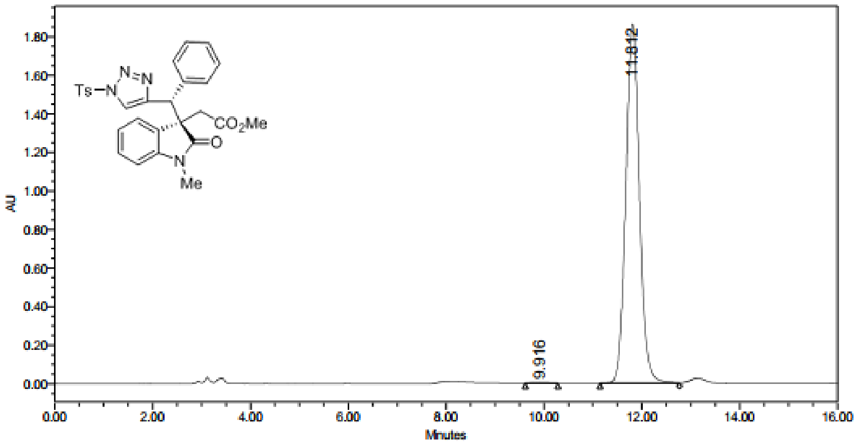

	RT	Area	% Area	Height
1	9.916	39194	0.11	2247
2	11.812	36035727	99.89	1861785

Supplementary Fig. 95. HPLC spectra of compound **7**

SAMPLE INFORMATION

Sample Name:	wyh-1-160-rac-IG-20%-1ml/min	Acquired By:	System
Sample Type:	Unknown	Sample Set Name:	wyh1160rac
Vial:	31	Acq. Method Set:	20% 1ml
Injection #:	1	Processing Method:	1
Injection Volume:	10.00 ul	Channel Name:	254.0nm
Run Time:	20.0 Minutes	Proc. Chnl. Descr.:	2998 PDA 254.0 nm (2998
Date Acquired:	6/30/2021 7:32:35 PM CST		
Date Processed:	7/1/2021 11:25:32 PM CST		

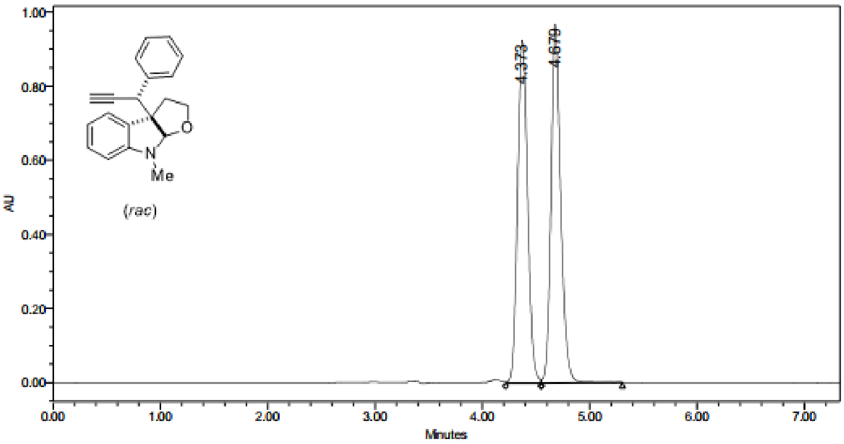

	RT	Area	% Area	Height
1	4.373	6065317	50.01	924829
2	4.679	6063342	49.99	968126

SAMPLE INFORMATION

Sample Name:	wyh-1-160-asy-IG-20%-1ml/min	Acquired By:	System
Sample Type:	Unknown	Sample Set Name:	111
Vial:	32	Acq. Method Set:	20% 1ml
Injection #:	1	Processing Method:	1
Injection Volume:	10.00 ul	Channel Name:	254.0nm
Run Time:	7.0 Minutes	Proc. Chnl. Descr.:	2998 PDA 254.0 nm (2998
Date Acquired:	6/30/2021 7:42:11 PM CST		
Date Processed:	7/1/2021 11:25:53 PM CST		

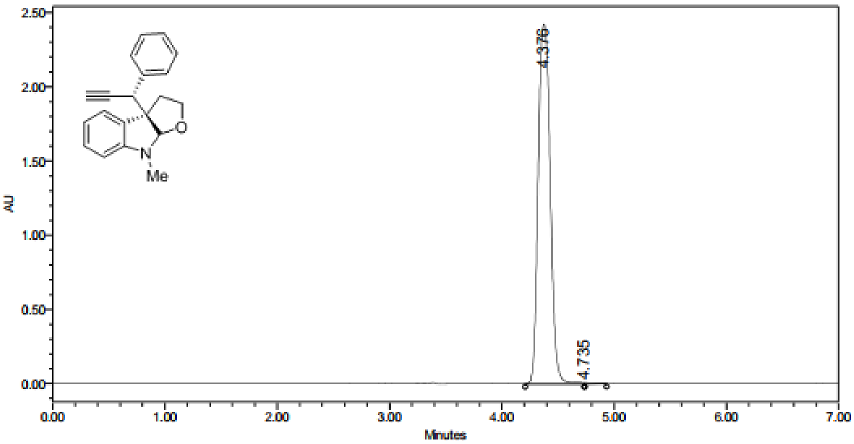

	RT	Area	% Area	Height
1	4.376	18114156	99.83	2421172
2	4.735	31604	0.17	6167

Supplementary Fig. 96. HPLC spectra of compound **8**

SAMPLE INFORMATION

Sample Name:	wyh-2-97-rac-IG-15%-1ml/min	Acquired By:	System
Sample Type:	Unknown	Sample Set Name:	
Vial:	17	Acq. Method Set:	15% 1ml
Injection #:	1	Processing Method:	1
Injection Volume:	10.00 ul	Channel Name:	254.0nm
Run Time:	60.0 Minutes	Proc. Chnl. Descr.:	2998 PDA 254.0 nm (2998
Date Acquired:	12/9/2021 3:36:01 PM CST		
Date Processed:	12/9/2021 4:30:39 PM CST		

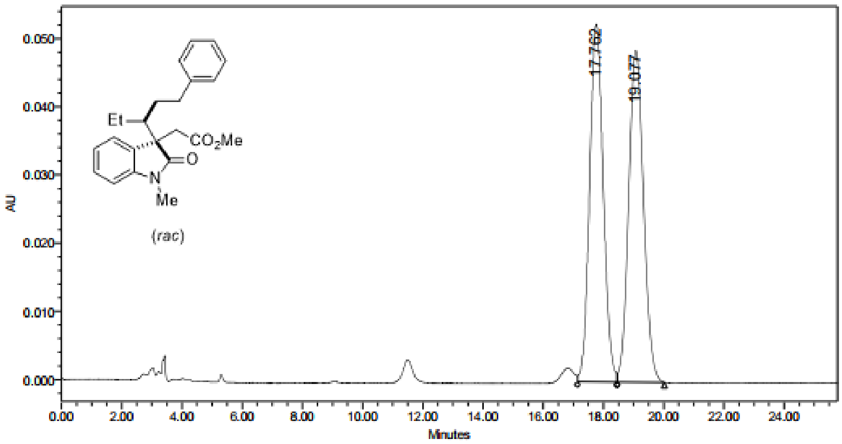

	RT	Area	% Area	Height
1	17.762	1656401	49.90	52435
2	19.077	1663372	50.10	48632

SAMPLE INFORMATION

Sample Name:	wyh-2-97-asy-IG-15%-1ml/min	Acquired By:	System
Sample Type:	Unknown	Sample Set Name:	
Vial:	18	Acq. Method Set:	15% 1ml
Injection #:	1	Processing Method:	1
Injection Volume:	10.00 ul	Channel Name:	254.0nm
Run Time:	60.0 Minutes	Proc. Chnl. Descr.:	2998 PDA 254.0 nm (2998
Date Acquired:	12/9/2021 4:02:37 PM CST		
Date Processed:	12/9/2021 4:32:50 PM CST		

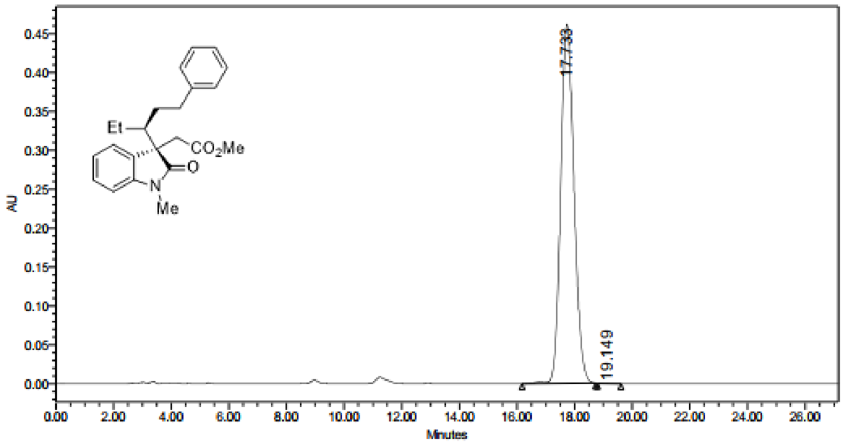

	RT	Area	% Area	Height
1	17.733	14606297	99.98	461161
2	19.149	3149	0.02	150

Supplementary Fig. 97. HPLC spectra of compound **9**

SAMPLE INFORMATION

Sample Name:	wyh-1-139-4-rac-d-IA-15%-1ml/min	Acquired By:	System
Sample Type:	Unknown	Sample Set Name:	
Vial:	20	Acq. Method Set:	15% 1ml
Injection #:	1	Processing Method:	1
Injection Volume:	10.00 ul	Channel Name:	254.0nm
Run Time:	60.0 Minutes	Proc. Chnl. Descr.:	2998 PDA 254.0 nm (2998
Date Acquired:	6/4/2021 10:54:29 AM CST		
Date Processed:	7/6/2021 9:36:51 AM CST		

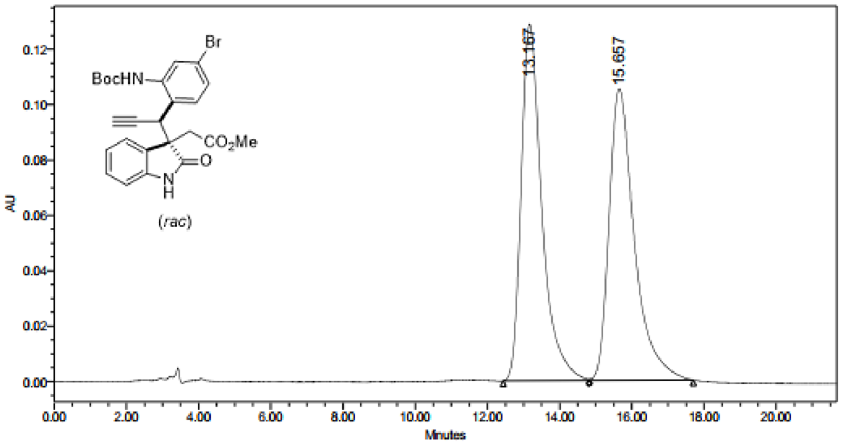

	RT	Area	% Area	Height
1	13.167	5158517	50.20	128896
2	15.657	5117450	49.80	105353

SAMPLE INFORMATION

Sample Name:	wyh-1-big-IA-15%-1ml/min	Acquired By:	System
Sample Type:	Unknown	Sample Set Name:	
Vial:	27	Acq. Method Set:	15% 1ml
Injection #:	1	Processing Method:	1
Injection Volume:	10.00 ul	Channel Name:	254.0nm
Run Time:	20.0 Minutes	Proc. Chnl. Descr.:	2998 PDA 254.0 nm (2998
Date Acquired:	6/17/2021 7:22:08 PM CST		
Date Processed:	7/6/2021 9:36:30 AM CST		

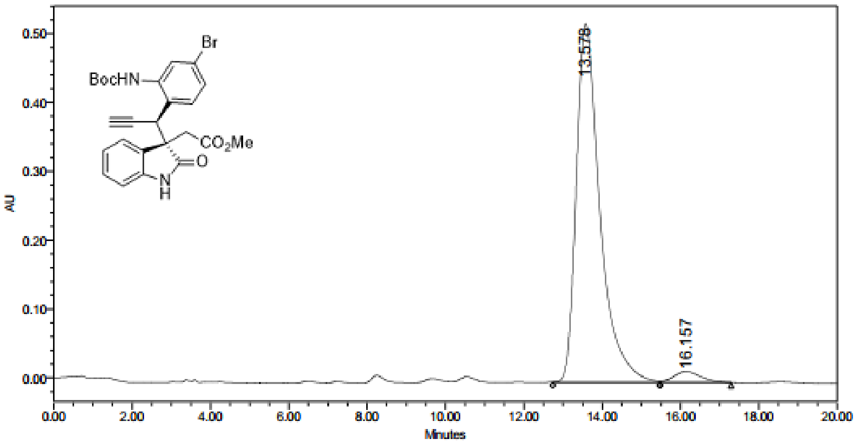

	RT	Area	% Area	Height
1	13.578	21938551	96.68	520679
2	16.157	753318	3.32	15868

Supplementary Fig. 98. HPLC spectra of compound **3nq**

SAMPLE INFORMATION

Sample Name:	wyh-1-OH-rac-IA-30%-1ml/min	Acquired By:	System
Sample Type:	Unknown	Sample Set Name:	
Vial:	32	Acq. Method Set:	30% 1ml
Injection #:	1	Processing Method:	1
Injection Volume:	10.00 ul	Channel Name:	254.0nm
Run Time:	60.0 Minutes	Proc. Chnl. Descr.:	2998 PDA 254.0 nm (2998
Date Acquired:	6/23/2021 2:22:06 PM CST		
Date Processed:	7/6/2021 9:23:24 AM CST		

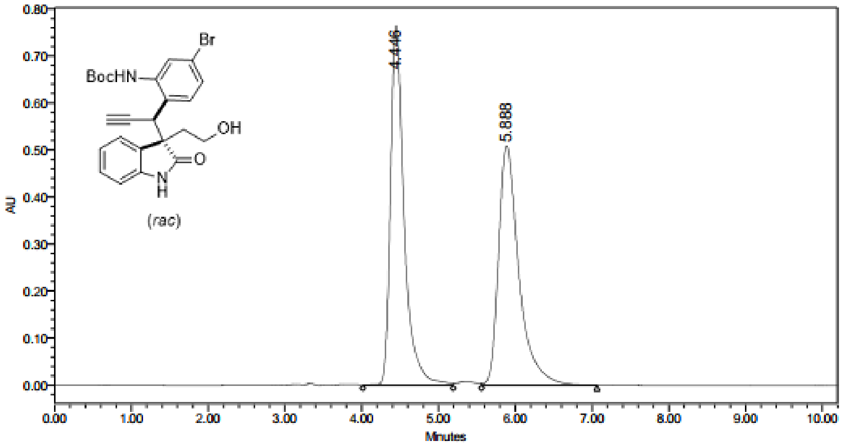

	RT	Area	% Area	Height
1	4.446	9489244	50.42	764717
2	5.888	9331807	49.58	509137

SAMPLE INFORMATION

Sample Name:	wyh-1-OH-asy-IA-30%-1ml/min	Acquired By:	System
Sample Type:	Unknown	Sample Set Name:	
Vial:	33	Acq. Method Set:	30% 1ml
Injection #:	1	Processing Method:	1
Injection Volume:	10.00 ul	Channel Name:	254.0nm
Run Time:	60.0 Minutes	Proc. Chnl. Descr.:	2998 PDA 254.0 nm (2998
Date Acquired:	6/23/2021 2:33:11 PM CST		
Date Processed:	7/6/2021 9:23:12 AM CST		

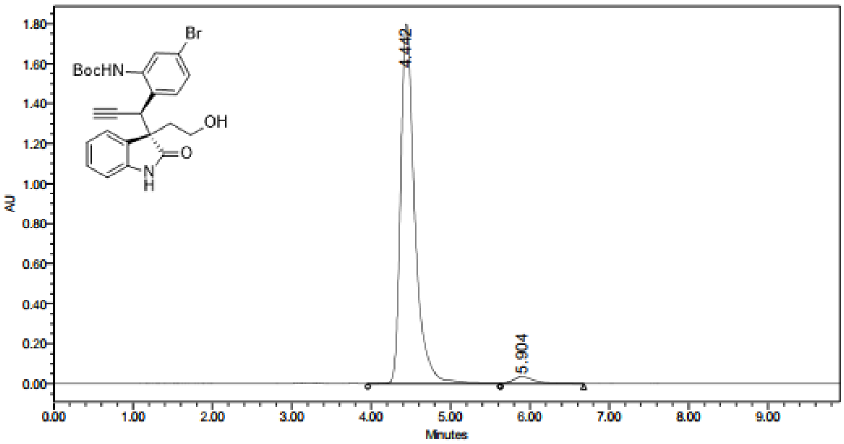

	RT	Area	% Area	Height
1	4.442	22415861	97.23	1797071
2	5.904	638986	2.77	34421

Supplementary Fig. 99. HPLC spectra of compound **10**

SAMPLE INFORMATION

Sample Name:	wyh-1-tp-rac-IA-2%-1ml/min	Acquired By:	System
Sample Type:	Unknown	Sample Set Name:	wyh1tprac
Vial:	5	Acq. Method Set:	2% 1ml
Injection #:	1	Processing Method:	1
Injection Volume:	10.00 ul	Channel Name:	220.0nm
Run Time:	80.0 Minutes	Proc. Chnl. Descr.:	2998 PDA 220.0 nm (2998
Date Acquired:	6/26/2021 2:53:18 PM CST		
Date Processed:	7/14/2021 2:48:50 PM CST		

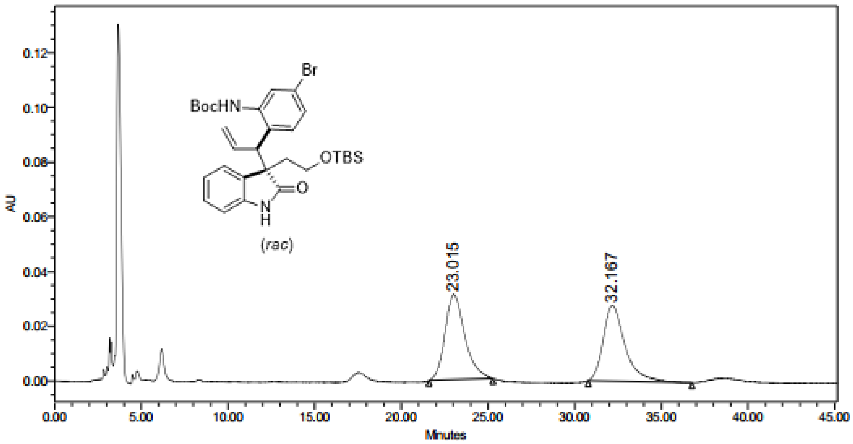

	RT	Area	% Area	Height
1	23.015	2490961	50.61	31199
2	32.167	2431204	49.39	27726

SAMPLE INFORMATION

Sample Name:	wyh-1-tp-asy-IA-2%-1ml/min	Acquired By:	System
Sample Type:	Unknown	Sample Set Name:	wyh1tpasy
Vial:	6	Acq. Method Set:	2% 1ml
Injection #:	1	Processing Method:	1
Injection Volume:	10.00 ul	Channel Name:	220.0nm
Run Time:	45.0 Minutes	Proc. Chnl. Descr.:	2998 PDA 220.0 nm (2998
Date Acquired:	6/26/2021 3:45:24 PM CST		
Date Processed:	7/14/2021 5:12:01 PM CST		

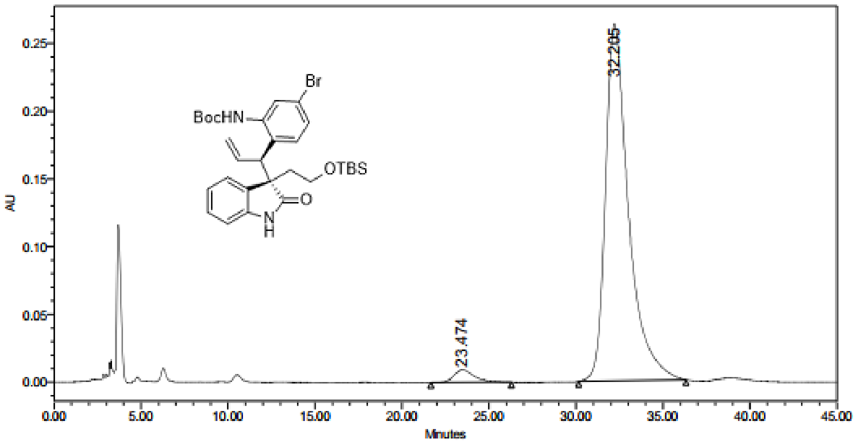

	RT	Area	% Area	Height
1	23.474	762704	3.04	9274
2	32.205	24358883	96.96	263412

Supplementary Fig. 100. HPLC spectra of compound **12**

4. Supplementary References

[1] Liu, R. & Zhang, J. *Chem. Eur. J.* **19**, 7319−7323(2013).

[2] Mukaiyama, T., Ogata, K., Sato, I. & Hayashi, Y. *Chem. Eur. J.* **20**, 13583−13588 (2014).

[3] Nayan, G., Sanatan, N. & Akhila, K. S. *J. Org. Chem.* **76**, 500−511 (2011).

[4] Hattori, G., Matsuzawa, H., Miyake, Y. & Nishibayashi, Y. *Angew. Chem., Int. Ed.* **47**, 3781−3783 (2008).

[5] Kerr, M. S., Read de Alaniz, J. & Rovis, T. *J. Org. Chem.* **70**, 5725−5728 (2005).

[6] Thai, K., Langdon, S. M., Bilodeau, F. & Gravel, M. *Org. Lett.* **15**, 2214−2217 (2013).

[7] Liu, E. & Topczewski, J. *J. Am. Chem. Soc.* **141**, 5135−5138 (2019)
